# Developing ‘high impact’ guideline-based quality indicators for UK primary care: a multi-stage consensus process

**DOI:** 10.1186/s12875-015-0350-6

**Published:** 2015-10-28

**Authors:** Bruno Rushforth, Tim Stokes, Elizabeth Andrews, Thomas A. Willis, Rosemary McEachan, Simon Faulkner, Robbie Foy

**Affiliations:** Foundry Lane Surgery, 3 Street Lane, Leeds, LS8 1BW UK; Department of General Practice and Rural Health, Dunedin School of Medicine, University of Otago, Dunedin, New Zealand; Bradford Institute for Health Research, Bradford, UK; Leeds Institute of Health Sciences, University of Leeds, Leeds, UK; Health and Social Care Information Centre, Leeds, UK; Leeds Institute of Health Sciences, University of Leeds, Leeds, UK

**Keywords:** Primary care, Review criteria, Clinical guidelines, Consensus development

## Abstract

**Background:**

Quality indicators (QIs) are an important tool for improving clinical practice and are increasingly being developed from evidence-based guideline recommendations. We aimed to identify, select and apply guideline recommendations to develop a set of QIs to measure the implementation of evidence-based practice using routinely recorded clinical data in United Kingdom (UK) primary care.

**Methods:**

We reviewed existing national clinical guidelines and QIs and used a four-stage consensus development process to derive a set of ‘high impact’ QIs relevant to primary care based upon explicit prioritisation criteria. We then field tested the QIs using remotely extracted, anonymised patient records from 89 randomly sampled primary care practices in the Yorkshire region of England.

**Results:**

Out of 2365 recommendations and QIs originally reviewed, we derived a set of 18 QIs (5 single, 13 composites – comprising 2–9 individual recommendations) for field testing. QIs predominantly addressed chronic disease management, in particular diabetes, cardiovascular and renal disease, and included both processes and outcomes of care. Field testing proved to be critical for further refinement and final selection.

**Conclusions:**

We have demonstrated a rigorous and transparent methodology to develop a set of high impact, evidence-based QIs for primary care from clinical guideline recommendations. While the development process was successful in developing a limited set of QIs, it remains challenging to derive robust new QIs from clinical guidelines in the absence of established systems for routine, structured recording of clinical care.

**Electronic supplementary material:**

The online version of this article (doi:10.1186/s12875-015-0350-6) contains supplementary material, which is available to authorized users.

## Background

Clinical evidence that can cut avoidable deaths and enhance quality of life does not reliably find its way into everyday patient care. It is widely recognised that the translation of evidence into practice is unpredictable and can be a slow and haphazard process [1]. In the United Kingdom, there are large geographical variations in the level and quality of care in a range of clinical areas, including diabetes, stroke and cancer; their magnitude cannot easily be explained away by population and case mix factors [2]. This gap between evidence and practice is a strategically important problem for policy-makers, healthcare systems and research funders because it limits the health, social and economic impacts of clinical research [3]. Limited resources inevitably mean that quality improvement initiatives cannot focus on all clinical guideline recommendations at once; clinicians need to identify and prioritise those that have the potential for the most positive impact for patients.

The primary care context presents particular implementation challenges not encountered in other settings – given limited practice organisational capacity, increasing complexity of care, and the dispersed and independent nature of practices [4–6]. An international review of quality of care studies from primary care concluded, “In almost all studies reviewed the quality of care did not attain acceptable standards of practice” [7]. A number of initiatives in the UK are used to increase implementation of effective practice in primary care. These include the development and dissemination of evidence-based clinical guidelines by the National Institute for Health and Care Excellence (NICE) [8] and the use of financial incentives to reward adherence to performance indicators set out in the Quality and Outcomes Framework (QOF) [9].

Measuring adherence to recommended practice is a cornerstone of any strategy to improve the quality of care. Measurement is required to identify inappropriate variations in practice, target improvement endeavours, and monitor their impact. In the absence of such data, implementation strategies are best-guess rather than data-driven. The development of quality indicators (QIs) from clinical guidelines offers a way to judge whether recommended practice is being followed and thereby allows the quality of care to be measured [10–12]. Formal consensus methods [13] are generally used both to prioritise those clinical guideline recommendations suitable for QI development and also to develop valid and reliable QIs [12, 14].

However, several challenges and considerations need to be balanced in the development and application of QIs:

- Indicators developed solely by expert panels may be “unoperationalisable, unreliable, too rare to be useful, or too hard to extract reliably.” [15].
- Methods that depend upon manual data extraction can be resource-intensive.
- The utility of routinely collected data drawn from existing schemes, such as QOF, is limited by incomplete coverage of health problems [16].
- Indicators focussing on health care processes should have a strong evidence base showing that the care process leads to improved outcomes, and recognise that there may be a number of intervening steps before an improved outcome is realised (for example, the steps of adding in antihypertensive medication, further monitoring blood tests as required, and further blood pressure review) [17].
- Indicators focusing on processes of care rather than health outcomes may not help overcome therapeutic inertia, i.e. the failure to intensify treatment in patients with an abnormal clinical measurement [18].
- Those focusing on health outcomes are subject to higher ‘noise to signal’ ratios, whereby a range of factors beyond professional practice influence outcomes [19–21].

Those leading and evaluating improvement strategies in primary care also need to consider efficiency. Implementation studies generally focus on one clinical condition. This has advantages, for example so that an intervention to promote better detection of hypertension complements another to improve the treatment of detected hypertension. However, the impact and generalisability of such studies is limited in a number of ways:

- Only a minority of single issue guideline recommendations are relevant to primary care and sufficiently clinically important to justify concerted implementation and provide a high return to investment ratio.
- Many important clinical practice recommendations are not directly amenable to measurement.
- There are risks of encountering ‘ceiling effects’ where adherence to a given recommendation has reached a point beyond which it is difficult to improve practice further.

We recognise that family physicians are generalists who already contend with a large number of QIs. We set out to develop and prioritise indicators that were both acceptable to physicians and likely to have the highest impact on patient care and outcomes [14]. Therefore, as part of a wider research programme (Action to Support Practices Implementing Research Evidence; ASPIRE), we identified and selected a range of what we term ‘high impact’ clinical practice recommendations and developed a limited set of QIs for UK primary care. ASPIRE ultimately aims to develop and undertake a randomised evaluation of the effects of implementation packages targeting high impact recommendations. We focused on evidence-based recommendations with greatest potential for improving quality of care and which could potentially be measured using routinely recorded data.

## Methods

We screened existing UK (NICE) clinical guideline recommendations and UK clinical primary care (QOF) indicators and used a four-stage consensus development process to identify those relevant to primary care based upon: burden of illness; potential for significant patient benefit; scope for improvement upon current levels of adherence; likelihood of cost savings without patient harm; feasibility of measuring adherence; and, the extent to which following a recommendation is directly within the control of individual practice teams or professionals [22]. We then developed QIs based on these recommendations or recommendation composites and undertook field-testing using routinely collected primary care practice data.

### Stage 1: initial screening

We identified candidate recommendations and indicators from three sources: all NICE clinical guidelines published from December 2002 to June 2012; all NICE Quality Standards published from June 2010 to June 2012; and all QOF clinical domain indicators as at June 2012. (From this point on, we will use the term ‘recommendations’ to also include ‘indicators’ whilst acknowledging that indicators are phrased differently given that they are measurement criteria.) One clinical researcher (BR) initially screened titles or summaries of the NICE guidelines and quality standards, excluding those relating exclusively to secondary care or those which had been superseded by a more recent update. From NICE guidelines, we extracted individual recommendations listed in the ‘key priorities for implementation’ from each guideline, together with the full set of recommendations from 16 guidelines judged particularly relevant to primary care (e.g. diagnosis and management of hypertension) (Additional file 1).

Two researchers (BR & RF) independently screened the resulting list of recommendations. We removed those judged irrelevant to primary care (e.g. ‘Decontaminate the skin at the insertion site with chlorhexidine gluconate in 70 % alcohol before inserting a peripheral vascular access device or a peripherally inserted central catheter’ [23]) or not measurable using routine data (e.g. ‘Healthcare professionals should adopt a consulting style that enables the child, young person or adult with epilepsy, and their family and/or carers as appropriate, to participate as partners in all decisions about their healthcare, and take fully into account their race, culture and any specific needs’ [24]). Sets of recommendations that were clearly linked to one another were grouped to form ‘composite’ recommendations (e.g. the nine recommended processes of care for patients with Type 2 diabetes [25]). We included recommendations judged to have potential for significant patient benefit either individually or as part of a composite with others. Disagreements were resolved through discussion.

### Stage 2: online shortlisting by consensus panel

We convened a consensus panel comprising 11 members: five family physicians, including two with responsibilities for commissioning healthcare services and population health; one practice nurse; one practice manager; one consultant clinical advisor from NICE accredited in public health; one health informatics specialist; and two patient representatives, one from a patient support and advocacy group and another with a role in commissioning. Whilst aiming for diversity, we deliberately weighted the panel towards primary care clinicians usually targeted by clinical practice recommendations; a number of judgements required an in-depth, tacit understanding of the day-to-day realities of clinical practice, e.g. knowledge of contraindications and need for monitoring when initiating beta-blockers for heart failure. We opted for 11 participants because a review of consensus development techniques indicated that we would gain relatively little in reliability by exceeding this number [13].

We conducted an online rating process whereby panellists rated each recommendation from Stage 1 according to three criteria: burden of illness (e.g. prevalence, severity, costs); potential for significant patient benefit (e.g. longevity, quality of life, safety of care); and scope for improvement upon current levels of adherence (e.g. from perceived current low levels or high variations). We instructed panellists to rate all recommendations on a 9-point Likert scale (where one is low and nine is high) according to their perceptions of current practice. Although patient representatives were encouraged to rate each recommendation, a ‘don’t know’ option was available if they felt unable to do so for this and subsequent stages. We piloted this process with three family physicians and two lay people beforehand and responded by clarifying instructions and briefing each panellist individually before the rating process. We analysed median scores for each rating using Excel and SPSS (version 19). We ranked recommendations according to the combined scores for burden of illness and patient benefit and also only retained those recommendations rated five or more on the ‘scope for improvement’ criterion. We aimed to apply a cut-off that would result in around 50 recommendations reaching the next stage.

### Stage 3: face-to-face consensus panel meeting

We used a modified RAND process, which is useful for judgements requiring deliberation and discussion [13]. First, panellists independently completed an additional online survey and rated the recommendations resulting from Stage 2 on a 9-point Likert scale according to three criteria: feasibility of measuring adherence (e.g. from clinical data routinely collected for QOF); extent to which following a recommendation is directly within the control of individual practice teams or professionals; and, the likelihood of cost savings without patient harm.

Panellists next attended a facilitated and structured face-to-face meeting. We presented median ratings for each recommendation. We focused discussion on those with maximal discordance, defined as at least three panellists scoring a recommendation 1–3 and at least three scoring it 7–9. Panellists had the opportunity to view summaries of the strength of evidence for each recommendation, clarify aspects of recommendations, and discuss reasons for low or high rankings. Panellists independently rated each recommendation again immediately after discussing each, taking into account panel deliberations and their own and aggregate initial ratings. We aimed to take the 20 top-rated recommendations forward for further development based upon the median ratings from Stage 2 and revised ratings from Stage 3.

### Stage 4: Informal sense-checking

We added this further stage after reviewing the Stage 3 rankings for two reasons. First, we were struck by unexpected rankings which appeared to lack face validity when considered against our criteria. For example, we had doubts about the feasibility of using routinely available data to measure adherence on recommended secondary prevention following myocardial infarction: ‘Advice on physical activity should involve a discussion about current and past activity levels and preferences. The benefit of exercise may be enhanced by tailored advice from a suitably qualified professional’ [26]. Second, we were planning to take selected high impact recommendations forward to develop and evaluate interventions to support their implementation. Hence, we wanted to ensure that selected recommendations were likely to be consistent with local priorities whilst their measurement was unlikely to face ceiling effects given known national and local initiatives.

We therefore identified a convenience sample of four family physician commissioning leads and six academic family physicians with whom we had existing working relationships and who had practical experience of measuring primary care outcomes. We asked them to review the full ranked list of recommendations from Stage 2, select between five and 10 recommendations that they considered would best meet our aims and highlight any they considered problematic to target. We then collated their selections and written comments. The research team drew upon this further feedback in discussions to finalise our selected high impact recommendations.

### Stage 5: Field testing

We randomly sampled and wrote to 114 general practices in West Yorkshire which used the SystmOne™ electronic health record. We first asked practices for consent to remote extraction of anonymised patient data (‘opt in’). Following a period of three to four weeks, we contacted practices again and asked them to inform us if they objected to the data extraction (‘opt out’).

One clinical researcher (BR) then drafted an expanded text for each recommendation, using logical operators (e.g. ‘AND’ and ‘OR’) to link descriptive statements to produce numerators and denominators which would identify whether the element of care occurred or not. Two other researchers (RF & TW) checked and amended these as appropriate prior to testing. A data analyst specialist (SF) took the numerators and denominators for each recommendation and generated search algorithms within SystmOne^TM^. We then undertook an iterative process of refinement of the search algorithms in light of the test data generated and following further input from two family physician advisors. For recommendations that were from the QOF such as: ‘The percentage of patients with diabetes in whom the last blood pressure is ≤ 140/80 mmHg’ [27], we utilised the existing QOF business rules set [28], although the timeframe for compliance for some recommendations was amended e.g. from 15 to 12 months. The QI from the composite recommendation on risky prescribing was constructed by a data analyst specialist (SF) using the specification of numerators and denominators by Dreischulte et al [29].

### Ethical review

The study was approved by National Research Ethics Service Committee Yorkshire and the Humber - Leeds Central (12/YH/0254). The Committee favourably reviewed the study, including the collection of anonymised patient data for the field testing without individual consent.

## Results

### Stage 1

**Fig. 1:**
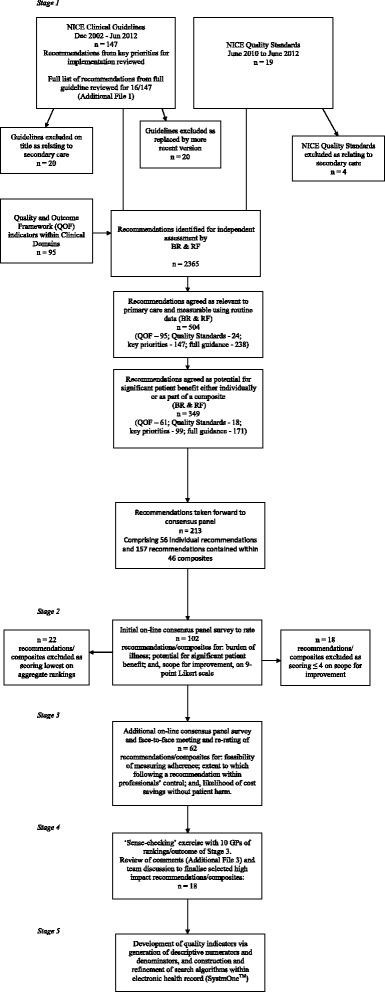
Flowchart of five stage recommendation selection and quality indicator development process

Out of 147 NICE clinical guidelines identified, we excluded 20 relating exclusively to secondary care and 20 which had been superseded by a more recent update (Fig. 1). We extracted all recommendations from 16 guidelines judged particularly relevant to primary care (Additional file 1). We identified 19 NICE Quality Standards, excluding four only relevant to secondary care, and 95 QOF clinical indicators. Together, these sources yielded a total of 2365 recommendations.

Screening of individual recommendations for relevance to primary care, patient benefit, and amenability to measurement yielded a total of 349 recommendations, 148 of which we judged as potentially significantly beneficial to patients and 201 as beneficial as part of a composite group. We then agreed 56 single and 46 composite recommendations respectively (Additional file 2).

### Stage 2

Online panel ratings (Additional file 2) across all 102 recommendations and composites were generally high for patient burden (mean ‘median’ score of 7.6; standard deviation 0.68) and potential for patient benefit (7.8; SD 0.81), with lower scores for scope for improvement (5.00; SD 0.88). We excluded 18 recommendations from further review because they scored four or less on scope for improvement (indicating that the panel perceived adherence to these recommendations to be relatively good). We then took forward 62 recommendations (31 single and 31 composites) based upon the highest aggregate rankings.

### Stage 3

The initial independent panel rating resulted in disagreements (at least three panellists scoring a recommendation 1–3 and at least three scoring it 7–9) for 22 (11.8 %) ratings, with 20 concerning feasibility of measuring adherence and two concerning the extent to which following a recommendation is directly within the control of individual practice teams or professionals. Following the panel meeting, there were disagreements for 12 (6.45 %) ratings. The mean ‘median’ ratings were: 6.8 (SD 1.57) for feasibility of measuring adherence, 7.2 (SD 0.76) for the extent to which following a recommendation is directly within the control of individual practice teams or professionals and 7.3 (SD 0.73) for the likelihood of cost savings without patient harm. Ratings for the 62 recommendations are shown in Additional file 3.

### Stage 4

As we had anticipated, comments from the subsequent ‘sense-checking’ exercise with family physicians mainly concerned perceived likelihoods of ceiling effects, difficulties in measurement or recommendations being outside the immediate control of the primary care team (Additional file 3). We therefore excluded further recommendations following team discussion, e.g.

- ‘The percentage of patients with coronary heart disease (CHD) who are currently treated with a beta-blocker’ [27]. *Judged at high risk of encountering likely ceiling effect in patients who have had myocardial infarctions; there are also credible recommended alternatives in those with angina* [30].
- ‘Cardiac rehabilitation should be equally accessible and relevant to all patients after a myocardial infarction (MI), particularly people from groups that are less likely to access this service’ [26]. *Considered largely outside immediate control of primary care teams.*
- ‘Carry out tests [for ovarian cancer] in primary care if a woman (especially if 50 or over) reports having any of the following symptoms on a persistent or frequent basis - particularly more than 12 times per month: persistent abdominal distension; feeling full and/or loss of appetite; pelvic or abdominal pain; increased urinary urgency and/or frequency’ [31]. *Considered too difficult to measure using routine data.*

We amalgamated two similar recommendations (concerning initiation of insulin in type 2 diabetes) and replaced one recommendation concerning prescribing non-steroid anti-inflammatory drugs (NSAIDs) with a composite recommendation on risky prescribing [29]. By the end of this process, we had derived a list of 18 recommendations (Table 1), 11 of which had been ranked in the top 20 by our panel, which mainly covered chronic disease management and cardiovascular disease.

**Table 1:** Final set of recommendations

Smoking: The percentage of patients in high risk groups whose notes record smoking status and the offer of support and treatment within preceding 15 months [composite].
Chronic Obstructive Pulmonary Disease (COPD): Diagnosis of COPD, through use of spirometry and chest radiograph [composite].
Chronic Kidney Disease (CKD): The percentage of patients on the CKD register with hypertension and proteinuria who are treated with an ACE-inhibitor or angiotensin receptor blocker.
Chronic Kidney Disease (CKD): Measurement of blood pressure**,** urinary protein excretion**,** and lifestyle advice [composite].
Chronic Kidney Disease (CKD): blood pressure and urinary protein excretion targets, and appropriate drug therapy [composite].
Myocardial infarction (MI): All patients who have had an acute MI should be offered specific combination drug treatment.
Chronic heart failure: Measurement of serum natriuretic peptides and referral where appropriate [composite].
Atrial fibrillation (AF): recommendations concerning use of anti-coagulants in AF [composite].
Hypertension: blood pressure targets in those under/over 80 years of age [composite].
Hypertension: lifestyle advice and monitoring of cholesterol and urinary protein excretion [composite].
Type 2 diabetes: 9 annual processes of care i.e. measurement of blood pressure, lipids, renal function, urine albumin-creatinine ratio (ACR), glycaemic control, BMI, smoking status, plus foot and eye checks [composite].
Type 2 diabetes: Integrate dietary advice with a personalised diabetes management plan.
Type 2 diabetes: Cardiovascular risk assessment and subsequent statin therapy where indicated.
Type 2 diabetes: Achievement of target levels for blood pressure, cholesterol and glycaemic control [composite].
Type 2 diabetes: For a person on dual therapy who is markedly hyperglycaemic, consider starting insulin therapy in preference to adding other drugs to control blood glucose.
Diabetes mellitus: The percentage of patients with diabetes in whom the last blood pressure is ≤ 140/80 mmHg.
Non-steroidal anti-inflammatory drugs (NSAIDs): Use of NSAIDs and monitoring of potential side-effects [composite].
Depression in adults: Recommendations concerning severity-appropriate treatment of depression [composite].

### Stage 5

Out of 114 practices initially approached, four opted in and seven refused; a further 15 opted out with our second (‘opt out’) approach. One had closed, one had merged with another in our sample and a third was excluded due to their non-standard patient list. Our final sample comprised 89 (78 %) practices.

**Fig. 2:**
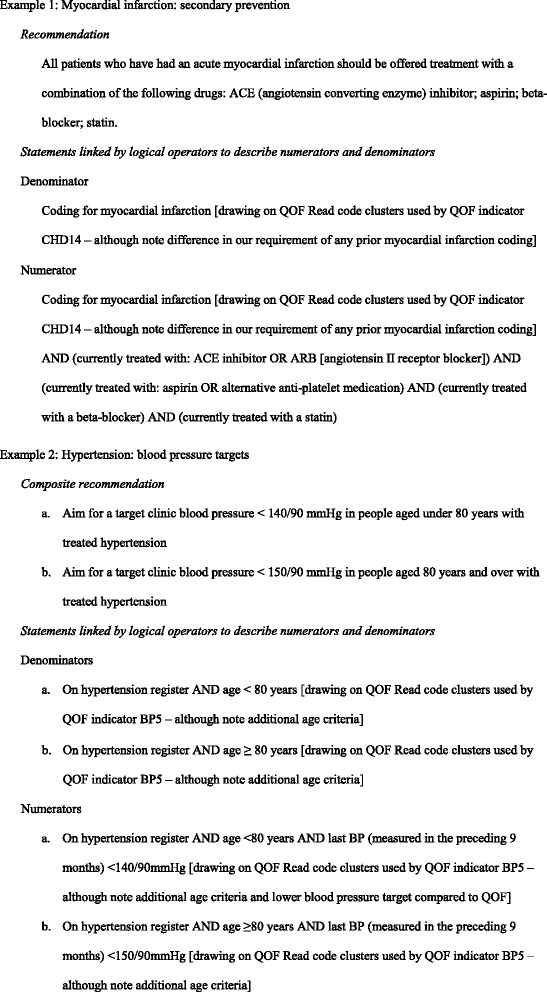
Worked examples of initial stages of quality indicator development process

Two worked examples of the initial stages of the QI development process for recommendations are presented in Fig. 2. The full set of SystmOne™ searches for each of the 18 recommendations is available in Additional file 4.

## Discussion

We developed a set of high impact QIs for primary care, drawn from clinical guideline recommendations, which can be measured using routinely collected data in UK primary care. We included a systematic, four-stage consensus process involving a multidisciplinary panel and field tested selected indicators.

Our final set of 18 QIs was drawn from 2365 recommendations and indicators; even allowing for duplications, our output demonstrates how much work is needed to derive a set of high impact recommendations. Other studies have also attempted to identify high impact or highly relevant clinical guidelines recommendations for primary care from existing national clinical guidelines and have similarly found this to be a labour intensive process with limited ‘yield’ in terms of a final set of recommendations highly relevant to primary care [32]. Our final set of QIs with its focus on chronic disease management, overlaps with existing primary care quality indicator sets [16, 33, 34] which also address chronic conditions such as diabetes and cardiovascular disease. We had intended that our work would lead to the development of new QIs to complement those currently available in national indicator sets for primary care in the UK, such as the QOF. However we found that, in line with recent attempts to develop new QOF indicators [35], our dependence on what can be extracted from routinely collected clinical data means that few new QIs can be developed [36]; the existing data sets are comprehensive in having developed most of the indicators that can be measured in current UK primary care computer systems.

There is an emerging consensus on methods of QI development [12, 14, 37, 38]. Our work included key recommended processes. Although we did not undertake separate systematic reviews of the evidence underpinning each indicator, we drew almost exclusively upon high-quality clinical guidelines [12] and shared summaries of evidence with our panel. We used a four stage consensus process, incorporating a modified RAND process with a multidisciplinary panel [13], to derive a set of QIs relevant to primary care based upon explicit prioritisation criteria. We field tested and subsequently refined our indicator set, an exercise undertaken for less than half of the indicators identified in a systematic review [12].

We draw attention to six limitations of our work, mainly so that others can improve upon our methods. First, our panel only explicitly discussed disagreements on three types of rating criteria (feasible to measure; under control of individual practice teams or professionals; and potential for cost savings without harm), after they had initially screened a larger list on patient burden, potential for patient benefit, and room for improvement. Anticipating the need to prioritise panellists’ time, we focused their discussion on the former criteria that we considered would require higher degrees of judgment. It is worth noting that there was a disagreement level of only 7.5 % in the first survey (23 disagreements from 306 ratings), with all but one focusing on rating ‘room for improvement’. This low level of disagreement suggests that although an initial round of ratings is likely to achieve sufficient consensus, a second round is important to clarify opinion on more difficult ratings. It is also important to decide *a priori* whether increased weighting should be given to certain components which are judged to be of greater importance as weighted criteria may have affected the Stage 3 outcome. Within the current study, feasibility of measurement was a key concern – however, this was given equal weight to all other criteria. A *post-hoc* analysis with ‘feasibility’ double-weighted only made a limited difference to the rankings, but this is an issue which should be considered in future consensus processes of this type.

Second, our process lost a degree of transparency through inclusion of an unstructured and relatively informal Stage 4, whereby we ‘sense-checked’ our panel outputs with a wider group of academic and commissioning family physicians. The need to add this stage somewhat highlights a relative failure of our preceding consensus process to scrutinise the candidate indicators within their limited time and experience. Panels developing indicators, even those including generalists, may tend to over-estimate the feasibility of data collection [36].

Third, our final set of criteria is skewed towards biomarkers (e.g. glycaemic control in diabetes) used for chronic disease monitoring. We recognise the risk of marginalising holistic medical care through focusing attention on what is measurable and what is not necessarily important to patients or physicians [39]. However, as well as including patient representatives in our consensus process, we also sought to maintain a focus on recommendations supported by evidence of benefits for patient and population outcomes (e.g. smoking cessation). We would welcome further work to develop evidence-based indicators of holistic care for which physicians would reliably record data.

Fourth, our approach to developing high impact indicators prioritised those associated with higher population burdens of illness. One critique is that this approach is prejudicial to rare diseases for which appropriate care could make a major difference to individual outcomes [38]. We recognise that we made a trade-off here.

Fifth, we did not directly assess the reliability of data recording. However, many of our measures were derived from data which had either been through reliability checks during piloting or were QOF indicators [40].

Sixth, the detailed operationalization of our indicators is only relevant to UK primary care. Nevertheless, their evidence base and basic structures should be transferable to similar primary care settings.

One key implication of our work is that using clinical guideline recommendations to develop QIs for use in primary care is likely to lead to an indicator set that focuses predominantly on chronic disease management of a range of single important diseases (cardiovascular disease, diabetes, chronic kidney disease). The current absence of clinical guideline recommendations for patients with multi-morbidity [41] means that we have not been able to consider the development of indicators that measure the quality of care for this group of patients.

Clinical evidence and subsequent guidance continue to evolve; Shekelle et al suggest that, as a general rule, guidelines should be re-evaluated for potential updating no less frequently than every three years [42]. We therefore agree with Stelfox and Straus that indicator development should be an ongoing process to reflect important changes [37]. For example, we are presently re-examining our indicators for atrial fibrillation in light of updated guidance, essentially indicating that anticoagulation treatment, after taking bleeding risk into account, has become the expected standard of medical care [43].

Our multi-disciplinary panel included both professional and patient representatives; Kotter et al found that patient participation during QI development is extremely uncommon [12]. We were uncertain as to whether patient representatives would be able to engage in the ratings of complex recommendations. We repeated our analyses on identifying the numbers of disagreements in the stage 3 survey (feasibility, control and cost-saving) with, and without patient representatives. Average median ratings across all criteria did not change with the inclusion of patient representatives, suggesting that patient opinions on these criteria did not markedly differ from those of the professional panellists.

## Conclusion

We have demonstrated that it is feasible to develop a selected set of high impact QIs for primary care. Our development process required considerable filtering of existing guidelines and is highly dependent upon the availability of routinely recorded data. Our methods were also more iterative and required more judgment than we had originally planned, especially considering our additional sense checking stage and refinements following field testing. We will report practice performance against this set of QIs separately. Future work will focus on a subset of these indicators which we will use as outcome measures in a cluster randomised evaluation of strategies to improve professional practice.

## Additional files

Additional file 1: List of NICE clinical guidelines judged particularly relevant to primary care. (DOCX 11 kb) Additional file 2: Results from initial Stage 2 online panel rating of 102 recommendations/composites. (DOCX 76 kb) Additional file 3: Ratings for the 62 recommendations/composites from Stage 3. (DOCX 47 kb) Additional file 4 Folder containing SystmOne™ search algorithms. (ZIP 12.7 mb)

## Abbreviations

QI: Quality indicator
QOF: Quality and Outcomes Framework
NICE: National Institute for Health and Care Excellence
ASPIRE: Action to Support Practices Implementing Research Evidence
ACE: Angiotensin converting enzyme
