# Supplementary material for: Developing ‘high impact’ guideline-based quality indicators for UK primary care: a multi-stage consensus process
Source: BMC Fam Pract. 2015 Oct 28;16:156. doi: 10.1186/s12875-015-0350-6 (PMC4624600; doi:10.1186/s12875-015-0350-6)
Supplement: Additional file 1: — List of NICE clinical guidelines judged particularly relevant to primary care. (DOCX 11 kb) [file 12875_2015_350_MOESM1_ESM.docx]

**Additional File 1.** List of NICE clinical guidelines judged particularly relevant to primary care

| Clinical Guideline 127, August 2011: Hypertension |
| --- |
| Clinical Guideline 126, July 2011: Stable Angina |
| Clinical Guideline 108, August 2010: Chronic Heart Failure |
| Clinical Guideline 101, June 2010: Chronic Obstructed Pulmonary Disease (updated) |
| Clinical Guideline 90, October 2009: Depression in Adults (update) |
| Clinical Guideline 87, May 2009: Type 2 Diabetes – newer agents (partial update of Clinical Guideline 66) |
| Clinical Guideline 66, May 2008: Type 2 Diabetes (partially updated by Clinical Guideline 87) |
| Clinical Guideline 88, May 2009: Low Back Pain |
| Clinical Guideline 67, May 2008: Lipid modification |
| Clinical Guideline 48, May 2007: Myocardial Infarction: secondary prevention |
| Clinical Guideline 73, September 2008: Chronic Kidney Disease |
| Clinical Guideline 68, July 2008: Stroke |
| Clinical Guideline 59, February 2008: Osteoarthritis |
| Clinical Guideline 36, June 2006: Atrial Fibrillation |
| Clinical Guideline 27, June 2005: Referral for suspected cancer |
| Clinical Guideline 17, August 2004: Dyspepsia |
