# Supplementary material for: Developing ‘high impact’ guideline-based quality indicators for UK primary care: a multi-stage consensus process
Source: BMC Fam Pract. 2015 Oct 28;16:156. doi: 10.1186/s12875-015-0350-6 (PMC4624600; doi:10.1186/s12875-015-0350-6)
Supplement: Additional file 2: — Results from initial Stage 2 online panel rating of 102 recommendations/composites. (DOCX 76 kb) [file 12875_2015_350_MOESM2_ESM.docx]

Additional File 2. Results from initial Stage 2 online panel rating of 102 recommendations/composites

| Item | Rating A: Patient burden  (Mean median score) | Rating B: Potential for patient benefit  (Mean median score) | Rating C: Room for improvement in current practice  (Mean median score) | Average ABC | Average AB | RANKING (ALL INDICES) | RANKING (A+B) |
| --- | --- | --- | --- | --- | --- | --- | --- |
| 15. **Alcohol dependence and harmful alcohol use quality standard**  Health and social care staff opportunistically carry out screening and brief interventions for hazardous and harmful drinking as an integral part of practice. | 9.0 | 8.0 | 6.5 | 7.83 | 8.5 | 1 | 1 |
| 48. **Myocardial infarction - secondary prevention**  Advice on physical activity should involve a discussion about current and past activity levels and preferences. The benefit of exercise may be enhanced by tailored advice from a suitably qualified professional. | 8.0 | 9.0 | 6.5 | 7.83 | 8.5 | 1 | 1 |
| 58. **Type II diabetes**  Integrate dietary advice with a personalised diabetes management plan, including other aspects of lifestyle modification, such as increasing physical activity and losing weight | 8.0 | 9.0 | 6.0 | 7.67 | 8.5 | 3 | 1 |
| 14. **Chronic heart failure**  Aspirin (75-150 mg once daily) should be prescribed for patients with the combination of heart failure and atherosclerotic arterial disease (including coronary heart disease). | 8.0 | 9.0 | 5.5 | 7.5 | 8.5 | 4 | 1 |
| 106. **Composites**  **Type 1 Diabetes**  Adults with type 1 diabetes should be assessed for arterial risk at annual intervals. Those found to be at increased risk should be managed through appropriate interventions and regular review. Note should be taken of: microalbuminuria, in particular; the presence of features of the metabolic syndrome; conventional risk factors (family history, abnormal lipid profile, raised blood pressure, smoking). (1 of 2)  Adults with type 1 diabetes should be assessed for early markers and features of eye, kidney, nerve, foot and arterial damage at annual intervals. According to assessed need, they should be offered appropriate interventions and/or referral in order to reduce the progression of such late complications into adverse health outcomes affecting quality of life. (2 of 2) | 8.0 | 9.0 | 5.5 | 7.5 | 8.5 | 4 | 1 |
| 116. **Composites**  **Hypertension -** **MONITORING AND LIFESTYLE**  For all people with hypertension offer to: - test for the presence of protein in the urine by sending a urine sample for estimation of the albumin:creatinine ratio and test for haematuria using a reagent strip; take a blood sample to measure plasma glucose, electrolytes, creatinine, estimated glomerular filtration rate, serum total cholesterol and HDL cholesterol; examine the fundi for the presence of hypertensive retinopathy, arrange for a 12-lead electrocardiograph to be performed. (1 of 7)  Use a formal estimation of cardiovascular risk to discuss prognosis and healthcare options with people with hypertension, both for raised blood pressure and other modifiable risk factors. (2 of 7)  Lifestyle advice should be offered initially and then periodically to people undergoing assessment or treatment for hypertension. (3 of 7)  Ascertain people's diet and exercise patterns because a healthy diet and regular exercise can reduce blood pressure. Offer appropriate guidance and written or audiovisual materials to promote lifestyle changes. (4 of 7)  Ascertain people's alcohol consumption and encourage a reduced intake if they drink excessively, because this can reduce blood pressure and has broader health benefits. (5 of 7)  Offer advice and help to smokers to stop smoking. (6 of 7)  Provide an annual review of care to monitor blood pressure, provide people with support and discuss their lifestyle, symptoms and medication. (7 of 7) | 8.0 | 9.0 | 5.5 | 7.5 | 8.5 | 4 | 1 |
| 98. **Composites**  **Depression in adults - TREATMENT**  For people with persistent subthreshold depressive symptoms or mild to moderate depression, consider offering one or more of the following interventions, guided by the person's preference: individual guided self-help based on the principles of cognitive behavioural therapy (CBT); computerised cognitive behavioural therapy (CCBT)218, a structured group physical activity programme. (1 of 3)  Do not use antidepressants routinely to treat persistent subthreshold depressive symptoms or mild depression because the risk--benefit ratio is poor, but consider them for people with: a past history of moderate or severe depression; or initial presentation of subthreshold depressive symptoms that have been present for a long period (typically at least 2 years); or subthreshold depressive symptoms or mild depression that persist(s) after other interventions. (2 of 3)  For people with moderate or severe depression, provide a combination of antidepressant medication and a high-intensity psychological intervention (CBT or interpersonal therapy [IPT]). (3 of 3) | 8.0 | 8.0 | 6.5 | 7.5 | 8 | 4 | 16 |
| 27. **Stroke**  People who have had a suspected TIA who are at high risk of stroke (that is, with an ABCD2 score of 4 or above) should have: aspirin (300 mg daily) started immediately; specialist assessment and investigation within 24 hours of onset of symptoms; measures for secondary prevention introduced as soon as the diagnosis is confirmed, including discussion of individual risk factors. | 8.0 | 9.0 | 5.0 | 7.33 | 8.5 | 8 | 1 |
| 41. **Type 1 diabetes**  Blood glucose control should be optimised towards attaining DCCT-harmonised HbA1c targets for prevention of microvascular disease (less than 7.5%) and, in those at increased risk, arterial disease (less than or equal to 6.5%) as appropriate, while taking into account: the experiences and preferences of the insulin user, in order to avoid hypoglycaemia; the necessity to seek advice from professionals knowledgeable about the range of available meal-time and basal insulins and about optimal combinations thereof, and their optimal use. | 8.0 | 9.0 | 5.0 | 7.33 | 8.5 | 8 | 1 |
| 42. **Lipid modification**  All people who smoke should be advised to stop, in line with 'Smoking cessation services' (NICE public health guidance 10). | 8.0 | 9.0 | 5.0 | 7.33 | 8.5 | 8 | 1 |
| 46. **Depression in adults**  For people with moderate or severe depression, provide a combination of antidepressant medication and a high-intensity psychological intervention (CBT or IPT). | 8.0 | 9.0 | 5.0 | 7.33 | 8.5 | 8 | 1 |
| 94. **Composites**  **Myocardial infarction - LIFESTYLE**  Patients should be advised to undertake regular physical activity sufficient to increase exercise capacity. (1 of 4)  All patients who smoke should be advised to quit and be offered assistance from a smoking cessation service in line with 'Brief interventions and referral for smoking cessation in primary care and other settings' (NICE public health intervention guidance 1). (2 of 4)  Patients who drink alcohol should be advised to keep weekly consumption within safe limits (no more than 21 units of alcohol per week for men, or 14 units per week for women) and to avoid binge drinking (more than 3 alcoholic drinks in 1-2 hours) (3 of 4)  Patients should be advised to eat a Mediterranean-style diet (more bread, fruit, vegetables and fish; less meat; and replace butter and cheese with products based on vegetable and plant oils). (4 of 4) | 8.0 | 9.0 | 5.0 | 7.33 | 8.5 | 8 | 1 |
| 133. **Composites**  **Ovarian cancer**  Carry out tests in primary care if a woman (especially if 50 or over) reports having any of the following symptoms on a persistent or frequent basis - particularly more than 12 times per month: persistent abdominal distension (women often refer to this as 'bloating'); feeling full (early satiety) and/or loss of appetite; pelvic or abdominal pain; increased urinary urgency and/or frequency. (1 of 6)  Carry out appropriate tests for ovarian cancer in any woman of 50 or over who has experienced symptoms within the last 12 months that suggest irritable bowel syndrome (IBS), because IBS rarely presents for the first time in women of this age. (2 of 6)  Measure serum CA125 in primary care in women with symptoms that suggest ovarian cancer. (3 of 6)  If serum CA125 is 35 IU/ml or greater, arrange an ultrasound scan of the abdomen and pelvis. (4of 6)  Women with raised CA125 have an ultrasound of their abdomen and pelvis within 2 weeks of receiving the CA125 test results. (5 of 6)  Women with normal CA125, or raised CA125 but normal ultrasound, with no confirmed diagnosis but continuing symptoms, are reassessed by their GP within 1 month. (6 of 6) | 7.5 | 9.0 | 5.5 | 7.33 | 8.25 | 8 | 14 |
| 57. **Type II diabetes**  For a person on dual therapy who is markedly hyperglycaemic, consider starting insulin therapy in preference to adding other drugs to control blood glucose unless there is strong justification not to. | 8.0 | 8.0 | 6.0 | 7.33 | 8 | 8 | 16 |
| 69. **Composites**  **Chronic heart failure - IDENTIFICATION AND REFERRAL**  Measure serum natriuretic peptides (B-type natriuretic peptide [BNP] or N-terminal pro-B-type natriuretic peptide [NTproBNP]) in patients with suspected heart failure without previous MI. (1 of 3)  Refer patients with suspected heart failure and a BNP level between 100 and 400pg/ml (29-116 pmol/litre), or an NTproBNP level between 400 and 2000 pg/ml (47-236 pmol/litre) to have transthoracic Doppler 2D echocardiography and specialist assessment within 6 weeks. (2 of 3)  Because very high levels of serum natriuretic peptides carry a poor prognosis, refer patients with suspected heart failure and a BNP level above 400 pg/ml (116 pmol/litre) or an NTproBNP level above 2000 pg/ml (236 pmol/litre) urgently, to have transthoracic Doppler 2D echocardiography and specialist assessment within 2 weeks. (3 of 3) | 8.0 | 8.0 | 6.0 | 7.33 | 8 | 8 | 16 |
| 75. **Composites**  **Chronic kidney disease - TREATMENT AND OUTCOMES OF CARE**  In people with CKD aim to keep the systolic blood pressure below 140 mmHg (target range 120-139 mmHg) and the diastolic blood pressure below 90 mmHg. (1 of 4)  In people with diabetes and CKD or when the ACR is greater than or equal to 70 mg/mmol, or PCR greater than or equal to 100 mg/mmol (approximately equivalent to PCR greater than or equal to 100 mg/mmol, or urinary protein excretion greater than or equal to 1.0 g/24 h) aim to keep the systolic blood pressure below 130 mmHg (target range 120-129 mmHg) and the diastolic blood pressure below 80 mmHg. (2 of 4)  Offer ACE inhibitors/ARBs to non-diabetic people with CKD and hypertension and ACR greater than or equal to 30 mg/mmol (approximately equivalent to PCR greater than or equal to 50 mg/mmol, or urinary protein excretion greater than or equal to 0.5 g/24 h). (3 of 4)  Offer ACE inhibitors/ARBs to non-diabetic people with CKD and ACR 70 mg/mmol or more (approximately equivalent to PCR 100 mg/mmol or more, or urinary protein excretion 1 g/24 h or more), irrespective of the presence of hypertension or cardiovascular disease.(4 of 4) | 8.0 | 8.0 | 6.0 | 7.33 | 8 | 8 | 16 |
| 81. **Composites**  **Chronic obstructive pulmonary disease - DIAGNOSIS**  A diagnosis of COPD should be considered in patients over the age of 35 who have a risk factor (generally smoking) and who present with exertional breathlessness, chronic cough, regular sputum production, frequent winter 'bronchitis' or wheeze. (1 of 3)  The presence of airflow obstruction should be confirmed by performing post-bronchodilator spirometry. All health professionals involved in the care of people with COPD should have access to spirometry and be competent in the interpretation of the results. (2 of 3)  At the time of their initial diagnostic evaluation in addition to spirometry all patients should have: a chest radiograph to exclude other pathologies, a full blood count to identify anaemia or polycythaemia, and body mass index (BMI) calculated. (3 of 3) | 8.0 | 8.0 | 6.0 | 7.33 | 8 | 8 | 16 |
| 84. **Composites**  **Chronic obstructive pulmonary disease - TREATMENT**  Short-acting bronchodilators, as necessary, should be the initial empirical treatment for the relief of breathlessness and exercise limitation. (1 of 8)  In people with stable COPD who remain breathless or have exacerbations despite use of short-acting bronchodilators as required, offer the following as maintenance therapy: if forced expiratory volume in 1 second (FEV1) is greater than or equal to 50% predicted: either long-acting beta2 agonist (LABA) or long-acting muscarinic antagonist (LAMA); if FEV1 < 50% predicted: either LABA with an inhaled corticosteroid (ICS) in a combination inhaler, or LAMA. (2 of 8)  Offer once-daily long-acting muscarinic antagonist (LAMA) in preference to four-times-daily short-acting muscarinic antagonist (SAMA) to people with stable COPD who remain breathless or have exacerbations despite using short-acting bronchodilators as required, and in whom a decision has been made to commence regular maintenance bronchodilator therapy with a muscarinic antagonist. (3 of 8)  In people with stable COPD and an FEV1 greater than or equal to 50% who remain breathless or have exacerbations despite maintenance therapy with a LABA: consider LABA+ICS in a combination inhaler. Consider LAMA in addition to LABA where ICS is declined or not tolerated. (4 of 8)  Consider LABA+ICS in a combination inhaler in addition to LAMA for people with stable COPD who remain breathless or have exacerbations despite maintenance therapy with LAMA irrespective of their FEV1. (5 of 8)  Offer LAMA in addition to LABA + ICS to people with COPD who remain breathless or have exacerbations despite taking LABA + ICS, irrespective of their FEV1. (6 of 8)  Osteoporosis prophylaxis should be considered in patients requiring frequent courses of oral corticosteroids. (7 of 8)  Pneumococcal vaccination and an annual influenza vaccination should be offered to all patients with COPD as recommended by the Chief Medical Officer. (8 of 8) | 8.0 | 8.0 | 6.0 | 7.33 | 8 | 8 | 16 |
| 86. **Composites**  **Chronic obstructive pulmonary disease - MONITORING**  People with COPD have a comprehensive clinical and psychosocial assessment, at least once a year or more frequently if indicated, which includes degree of breathlessness, frequency of exacerbations, validated measures of health status and prognosis, presence of hypoxaemia and comorbidities. (1 of 5)  When patients with very severe COPD are reviewed in primary care, they should be seen at least twice a year, and specific attention should be paid to the issues listed in table 7.7. (Page 350 in guideline) (2 of 5)  One of the primary symptoms of COPD is breathlessness. The Medical Research Council (MRC) dyspnoea scale should be used to grade the breathlessness according to the level of exertion required to elicit it. (3 of 5)  Patients should have their ability to use an inhaler device regularly assessed by a competent healthcare professional and, if necessary, should be re-taught the correct technique. (4 of 5)  BMI should be calculated in patients with COPD: the normal range for BMI is 20 to less than 25; if the BMI is abnormal (high or low), or changing over time, the patient should be referred for dietetic advice if the BMI is low patients should also be given nutritional supplements to increase their total calorific intake and be encouraged to take exercise to augment the effects of nutritional supplementation. (5 of 5) | 8.0 | 8.0 | 6.0 | 7.33 | 8 | 8 | 16 |
| 97. **Composites**  **Depression in adults – THOSE WITH PHYSICAL HEALTH PROBLEMS**  People with moderate depression and a chronic physical health problem receive an appropriate high-intensity psychological intervention. (1 of 3)  People with severe depression and a chronic physical health problem receive a combination of antidepressant medication and individual cognitive behavioural therapy. (2 of 3)  Do not use antidepressants routinely to treat subthreshold depressive symptoms or mild depression in patients with a chronic physical health problem (because the risk--benefit ratio is poor), but consider them for patients with: a past history of moderate or severe depression or − mild depression that complicates the care of the physical health problem; or initial presentation of subthreshold depressive symptoms that have been present for a long period (typically at least 2 years); or subthreshold depressive symptoms or mild depression that persist(s) after other interventions. (3 of 3) | 8.0 | 8.0 | 6.0 | 7.33 | 8 | 8 | 16 |
| 136. **Composites**  **Smoking - COMORBIDITIES**  The percentage of patients with any or any combination of the following conditions: CHD, PAD, stroke or TIA, hypertension, diabetes, COPD, CKD, asthma, schizophrenia, bipolar affective disorder or other psychoses whose notes record smoking status in the preceding 15 months. (1 of 2)  The percentage of patients with any or any combination of the following conditions: CHD, PAD, stroke or TIA, hypertension, diabetes, COPD, CKD, asthma, schizophrenia, bipolar affective disorder or other psychoses who smoke whose notes contain a record of an offer of support and treatment within the preceding 15 months. (2 of 2) | 8.0 | 8.0 | 6.0 | 7.33 | 8 | 8 | 16 |
| 65. **COPD**  Spirometry should be performed in patients who are over 35, current or ex-smokers, and have a chronic cough. | 8.0 | 7.0 | 7.0 | 7.33 | 7.5 | 8 | 46 |
| 49. **Myocardial infarction – secondary prevention**  All patients who smoke and who have expressed a desire to quit should be offered support and advice, and referral to an intensive support service (for example the NHS Stop Smoking Services) in line with 'Brief interventions and referral for smoking cessation in primary care and other settings' (NICE public health intervention guidance 1) (Grade A). If a patient is unable or unwilling to accept a referral they should be offered pharmacotherapy in line with the recommendations in 'Nicotine replacement therapy (NRT) and bupropion for smoking cessation' (NICE technology appraisal guidance 39) (Grade A). | 8.0 | 9.0 | 4.5 | 7.17 | 8.5 | 23 | 1 |
| 103. **Composites**  **Type 2 diabetes - PROCESSES OF CARE**  Measure blood pressure at least annually in a person without previously diagnosed hypertension or renal disease. Offer and reinforce preventive lifestyle advice. (1 of 9)  Measure the individual's HbA1c levels at: 2-6-monthly intervals (tailored to individual needs), until the blood glucose level is stable on unchanging therapy; use a measurement made at an interval of less than 3 months as an indicator of direction of change, rather than as a new steady state; 6-monthly intervals once the blood glucose level and blood glucose lowering therapy are stable. (2 of 9)  Perform full lipid profile (including high-density lipoprotein cholesterol and triglyceride estimations) when assessing cardiovascular risk annually, and before starting lipid-modifying therapy. (3 of 9)  The percentage of patients with diabetes who have a record of micro-albuminuria testing in the preceding 15 months (exception reporting for patients with proteinuria). (4 of 9)  The percentage of patients with diabetes who have a record of estimated glomerular filtration rate (eGFR) or serum creatinine testing in the preceding 15 months. (5 of 9)  [Type 2 Diabetes Foot care] Arrange recall and annual review as part of ongoing care. (6 of 9)  Arrange or perform eye screening at, or around, the time of diagnosis. Arrange repeat of structured eye surveillance annually. (7 of 9)  The percentage of patients with diabetes whose notes record BMI in the preceding 15 months. (8 of 9)  The percentage of patients with any or any combination of the following conditions: CHD, PAD, stroke or TIA, hypertension, diabetes, COPD, CKD, asthma, schizophrenia, bipolar affective disorder or other psychoses whose notes record smoking status in the preceding 15 months. (9 of 9) | 8.0 | 9.0 | 4.5 | 7.17 | 8.5 | 23 | 1 |
| 52. **Composite**  **Atrial fibrillation**  In patients with AF who are either post-stroke, or have had a TIA: warfarin should be administered as the most effective thromboprophylactic agent; aspirin or dipyridamole should not be administered as thromboprophylactic agents unless indicated for the treatment of comorbidities or vascular disease. (1 of 3) | 8.0 | 8.5 | 5.0 | 7.17 | 8.25 | 23 | 14 |
| 60. **Type II diabetes**  When other measures no longer achieve adequate blood glucose control to HbA1c <7.5% or other higher level agreed with the individual, discuss the benefits and risks of insulin therapy. Start insulin therapy if the person agrees. | 8.0 | 8.0 | 5.5 | 7.17 | 8 | 23 | 16 |
| 72. **Composites**  **Chronic heart failure - LIFESTYLE ISSUES, PREVENTATIVE MEASURES AND MONITORING**  Patients should be strongly advised not to smoke. Referral to smoking cessation services should be considered. (1 of 7)  Healthcare professionals should discuss alcohol consumption with the patient and tailor their advice appropriately to the clinical circumstances. (2 of 7)  The diagnosis of depression should be considered in all patients with heart failure. (3 of 7)  Patients with heart failure should be offered an annual vaccination against influenza. (4 of 7)  Patients with heart failure should be offered vaccination against pneumococcal disease (only required once). (5 of 7)  Measure serum urea, creatinine, electrolytes and eGFR at initiation of an ACE inhibitor and after each dose increment. (6 of 7)  Monitor serum urea, electrolytes, creatinine and eGFR for signs of renal impairment or hyperkalaemia in patients with heart failure who are taking an ARB. (7 of 7) | 8.0 | 8.0 | 5.5 | 7.17 | 8 | 23 | 16 |
| 92. **Composites**  **Myocardial infarction - ADJUNCTS**  All patients (regardless of their age) should be given advice about and offered a cardiac rehabilitation programme with an exercise component. (1 of 2)  Cardiac rehabilitation should be equally accessible and relevant to all patients after an MI, particularly people from groups that are less likely to access this service. These include people from black and minority ethnic groups, older people, people from lower socioeconomic groups, women, people from rural communities and people with mental and physical health comorbidities. (2 of 2) | 8.0 | 8.0 | 5.5 | 7.17 | 8 | 23 | 16 |
| 105. **Composites**  **Type 2 diabetes - INTERMEDIATE CLINICAL OUTCOMES OF CARE**  Add medications if lifestyle advice does not reduce blood pressure to below 140/80 mmHg (below 130/80 mmHg if there is kidney, eye or cerebrovascular damage). (1 of 3)  Consider intensifying cholesterol-lowering therapy (with a more effective statin or ezetimibe, in line with NICE guidance), if there is existing or newly diagnosed cardiovascular disease, or if there is an increased albumin excretion rate, to achieve a total cholesterol level below 4.0 mmol/litre (and high-density lipoprotein cholesterol not exceeding 1.4 mmol/litre) or a low-density lipoprotein cholesterol level below 2.0 mmol/litre. (2 of 3)  The percentage of patients with diabetes in whom the last IFCC-HbA1c is 59 mmol/mol or less in the preceding 15 months. (3 of 3) | 8.0 | 8.0 | 5.5 | 7.17 | 8 | 23 | 16 |
| 114. **Composites**  **Hypertension - BLOOD PRESSURE TARGETS**  Aim for a target clinic blood pressure below 140/90 mmHg in people aged under 80 years with treated hypertension. (1 of 2)  Aim for a target clinic blood pressure below 150/90 mmHg in people aged 80 years and over with treated hypertension. (2 of 2) | 8.0 | 8.0 | 5.5 | 7.17 | 8 | 23 | 16 |
| 122. **Composites**  **Lipid modification - CARDIOVASCULAR RISK**  For the primary prevention of CVD in primary care, a systematic strategy should be used to identify people aged 40-74 who are likely to be at high risk. (1 of 3)  People should be prioritised on the basis of an estimate of their CVD risk before a full formal risk assessment. Their CVD risk should be estimated using CVD risk factors already recorded in primary care electronic medical records. (2 of 3)  Risk equations should be used to assess CVD risk. (3 of 3) | 8.0 | 8.0 | 5.5 | 7.17 | 8 | 23 | 16 |
| 13. **Chronic heart failure**  Perform an ECG and consider the following tests to evaluate possible aggravating factors and/or alternative diagnoses: chest X-ray; blood tests; electrolytes, urea and creatinine; eGFR ; thyroid function tests; liver function tests; fasting lipids; fasting glucose; full blood count; urinalysis, peak flow or spirometry | 8.0 | 7.5 | 6.0 | 7.17 | 7.75 | 23 | 45 |
| 66. **COPD**  Pulmonary rehabilitation should be offered to all patients who consider themselves functionally disabled by COPD (usually MRC grade 3 and above). Pulmonary rehabilitation is not suitable for patients who are unable to walk, have unstable angina or who have had a recent myocardial infarction | 8.0 | 7.0 | 6.5 | 7.17 | 7.5 | 23 | 46 |
| 20. **CKD**  Encourage people with CKD to take exercise, achieve a healthy weight and stop smoking. | 8.0 | 8.0 | 5.0 | 7 | 8 | 34 | 16 |
| 23. **CKD**  The percentage of patients on the CKD register with hypertension and proteinuria who are treated with an angiotensin converting enzyme inhibitor (ACE inhibitor) or angiotensin receptor blocker (ARB). | 8.0 | 8.0 | 5.0 | 7 | 8 | 34 | 16 |
| 25. **Secondary prevention of coronary heart disease**  The percentage of patients with coronary heart disease with a record in the preceding 15 months that aspirin, an alternative anti-platelet therapy, or an anti-coagulant is being taken. | 8.0 | 8.0 | 5.0 | 7 | 8 | 34 | 16 |
| 26. **Secondary prevention of coronary heart disease**  The percentage of patients with coronary heart disease who are currently treated with a beta-blocker. | 8.0 | 8.0 | 5.0 | 7 | 8 | 34 | 16 |
| 31. **Dementia**  People with dementia should not be excluded from any services because of their diagnosis, age (whether designated too young or too old) or coexisting learning disabilities. | 8.0 | 8.0 | 5.0 | 7 | 8 | 34 | 16 |
| 37. **Hypertension**  If hypertension is not diagnosed, measure the person's clinic blood pressure at least every 5 years subsequently, and consider measuring it more frequently if the person's clinic blood pressure is close to 140/90 mmHg. | 8.0 | 8.0 | 5.0 | 7 | 8 | 34 | 16 |
| 38. **Hypertension**  The percentage of patients with hypertension in whom the last blood pressure (measured in the preceding 9 months) is 150/90 or less. | 8.0 | 8.0 | 5.0 | 7 | 8 | 34 | 16 |
| 53. **Composite**  **Atrial fibrillation**  In those patients with atrial fibrillation in whom there is a record of a CHADS2 score of 1 (latest in the preceding 15 months), the percentage of patients who are currently treated with anti-coagulation drug therapy or anti-platelet therapy. (2 of 3) | 8.0 | 8.0 | 5.0 | 7 | 8 | 34 | 16 |
| 54. **Composite**  **Atrial fibrillation**  In those patients with atrial fibrillation whose latest record of a CHADS2 score is greater than 1, the percentage of patients who are currently treated with anti-coagulation therapy. (3 of 3) | 8.0 | 8.0 | 5.0 | 7 | 8 | 34 | 16 |
| 62. **Diabetes mellitus**  The percentage of patients with diabetes in whom the last blood pressure is 140/80 or less. | 8.0 | 8.0 | 5.0 | 7 | 8 | 34 | 16 |
| 137. **Composites**  **Smoking - DOCUMENTATION AND ADVICE**  The percentage of patients aged 15 years and over whose notes record smoking status in the preceding 27 months. (1 of 2)  The percentage of patients aged 15 years and over who are recorded as current smokers who have a record of an offer of support and treatment within the preceding 27 months. (2 of 2) | 7.5 | 8.5 | 5.0 | 7 | 8 | 34 | 16 |
| 12. **Chronic heart failure**  All patients with chronic heart failure require monitoring. This monitoring should include: a clinical assessment of functional capacity; fluid status; cardiac rhythm (minimum of examining the pulse); cognitive status and nutritional status; a review of medication, including need for changes and possible side effects; and serum urea, electrolytes, creatinine and eGFR (estimated glomerular filtration rate). | 8.0 | 7.0 | 6.0 | 7 | 7.5 | 34 | 46 |
| 22. **CKD**  People with higher levels of proteinuria, and people with diabetes and microalbuminuria, are enabled to safely maintain their systolic blood pressure within a target range 120-129 mmHg and their diastolic blood pressure below 80 mmHg. | 7.0 | 8.0 | 6.0 | 7 | 7.5 | 34 | 46 |
| 68. **Composites**  **Chronic heart failure - TREATMENT**  People with chronic heart failure due to left ventricular systolic dysfunction are offered angiotensin-converting enzyme inhibitors (or angiotensin II receptor antagonists licensed for heart failure if there are intolerable side effects with angiotensin-converting enzyme inhibitors) and beta-blockers licensed for heart failure, which are gradually increased up to the optimal tolerated or target dose with monitoring after each increase. (1 of 2)  Offer beta-blockers licensed for heart failure to all patients with heart failure due to left ventricular systolic dysfunction, including: - older adults and patients with: peripheral vascular disease; erectile dysfunction; diabetes mellitus; interstitial pulmonary disease, and chronic obstructive pulmonary disease (COPD) without reversibility. (2 of 2) | 7.5 | 7.0 | 6.5 | 7 | 7.25 | 34 | 66 |
| 61. **Type II diabetes**  For a person who is 40 years old or over: - initiate therapy with generic simvastatin (to 40 mg) or a statin of similar efficacy and cost unless the cardiovascular risk from non-hyperglycaemia-related factors is low; if the cardiovascular risk from non-hyperglycaemia-related factors is low, assess cardiovascular risk using the UKPDS risk engine and initiate simvastatin therapy (to 40 mg), or a statin of similar efficacy and cost, if the cardiovascular risk exceeds 20% over 10 years. | 8.0 | 8.0 | 4.5 | 6.83 | 8 | 48 | 16 |
| 113. **Composites**  **Hypertension - INITIATING TREATMENT**  Offer antihypertensive drug treatment to people aged under 80 years with stage 1 hypertension who have one or more of the following: target organ damage; established cardiovascular disease; renal disease; diabetes, a 10-year cardiovascular risk equivalent to 20% or greater. (1 of 3)  Offer antihypertensive drug treatment to people of any age with stage 2 hypertension. (2 of 3)  For people aged under 40 years with stage 1 hypertension and no evidence of target organ damage, cardiovascular disease, renal disease or diabetes, consider seeking specialist evaluation of secondary causes of hypertension and a more detailed assessment of potential target organ damage. This is because 10-year cardiovascular risk assessments can underestimate the lifetime risk of cardiovascular events in these people. (3 of 3) | 8.0 | 8.0 | 4.5 | 6.83 | 8 | 48 | 16 |
| 123. **Composites**  **Lipid modification - SECONDARY PREVENTION**  For secondary prevention, lipid modification therapy should be offered and should not be delayed by management of modifiable risk factors. Blood tests and clinical assessment should be performed, and comordbidities and secondary causes of dyslipidaemia should be treated. Assessment should include: smoking status; alcohol consumption; blood pressure (see 'Hypertension', NICE clinical guideline 34); body mass index or other measure of obesity (see 'Obesity', NICE clinical guideline 43); fasting total cholesterol, LDL cholesterol, HDL cholesterol and triglycerides (if fasting levels are not already available); fasting blood glucose; renal function; liver function (transaminases); thyroid-stimulating hormone (TSH) if dyslipidaemia is present. (1 of 2)  In people taking statins for secondary prevention, consider increasing to simvastatin 80 mg or a drug of similar efficacy and acquisition cost if a total cholesterol of less than 4 mmol/litre or an LDL cholesterol of less than 2 mmol/litre is not attained. Any decision to offer a higher intensity statin should take into account informed preference, comorbidities, multiple drug therapy, and the benefit and risks of treatment. (2 of 2) | 8.0 | 8.0 | 4.5 | 6.83 | 8 | 48 | 16 |
| 11. **Chronic heart failure**  Offer both angiotensin-converting enzyme (ACE) inhibitors and betablockers licensed for heart failure to all patients with heart failure due to left ventricular systolic dysfunction. Use clinical judgement when deciding which drug to start first. | 8.0 | 7.0 | 5.5 | 6.83 | 7.5 | 48 | 46 |
| 21. **CKD**  People with CKD are assessed for cardiovascular risk. | 8.0 | 7.0 | 5.5 | 6.83 | 7.5 | 48 | 46 |
| 96. **Composites**  **Depression in adults - IDENTIFICATION AND DIAGNOSIS**  Be alert to possible depression (particularly in people with a past history of depression, possible somatic symptoms of depression or a chronic physical health problem with associated functional impairment) and consider asking people who may have depression two questions, specifically: During the last month, have you often been bothered by feeling down, depressed or hopeless? During the last month, have you often been bothered by having little interest or pleasure in doing things? If a person answers 'yes' to either of the above questions consider depression and follow the recommendations for assessment. ( 1 of 2)  [Common Mental Health Disorders 5.4.1.3] When assessing a person with a suspected common mental health disorder, consider using: - a diagnostic or problem identification tool or algorithm, for example the Improving Access to Psychological Therapies (IAPT) screening prompts tool20; - a validated measure relevant to the disorder or problem being assessed, for example, the 9-item Patient Health Questionnaire (PHQ-9), the Hospital Anxiety and Depression Scale (HADS) or the 7-item Generalized Anxiety Disorder scale (GAD-7) to inform the assessment and support the evaluation of any intervention. (2 of 2). | 8.0 | 7.0 | 5.5 | 6.83 | 7.5 | 48 | 46 |
| 131. **Composites**  **Opioids in palliative care**  When starting treatment with strong opioids, offer patients with advanced and progressive disease regular oral sustained-release or oral immediate-release morphine (depending on patient preference), with rescue doses of oral immediate-release morphine for breakthrough pain. (1 of 4)  For patients with no renal or hepatic comorbidities, offer a typical total daily starting dose schedule of 20-30 mg of oral morphine (for example, 10-15 mg oral sustained-release morphine twice daily), plus 5 mg oral immediate-release morphine for rescue doses during the titration phase. (2 of 4)  Do not routinely offer transdermal patch formulations as first-line maintenance treatment to patients in whom oral opioids are suitable. (3 of 4)  Prescribe laxative treatment (to be taken regularly at an effective dose) for all patients initiating strong opioids. (4 of 4) | 7.0 | 8.0 | 5.5 | 6.83 | 7.5 | 48 | 46 |
| 78. **Composites**  **Coeliac disease**  Offer serological testing for coeliac disease to children and adults with any of the following conditions: autoimmune thyroid disease; dermatitis herpetiformis; irritable bowel syndrome; type 1 diabetes, or first-degree relatives (parents, siblings or children) with coeliac disease. (1 of 2)  Offer serological testing for coeliac disease to children and adults with any of the following signs and symptoms: - chronic or intermittent diarrhoea; failure to thrive or faltering growth (in children); persistent or unexplained gastrointestinal symptoms including nausea and vomiting; prolonged fatigue ('tired all the time'); recurrent abdominal pain, cramping or distension; sudden or unexpected weight loss; unexplained iron-deficiency anaemia, or other unspecified anaemia. (2 of 2) | 6.0 | 8.0 | 6.5 | 6.83 | 7 | 48 | 68 |
| 8. **Peripheral arterial disease**  The percentage of patients with peripheral arterial disease with a record in the preceding 15 months that aspirin or an alternative anti-platelet is being taken. | 7.0 | 8.0 | 5.0 | 6.67 | 7.5 | 56 | 46 |
| 18. **CKD**  Offer people testing for CKD if they have any of the following risk factors: diabetes; hypertension; cardiovascular disease (ischaemic heart disease, chronic heart failure, peripheral vascular disease and cerebral vascular disease); structural renal tract disease, renal calculi or prostatic hypertrophy; multisystem diseases with potential kidney involvement, for example, systemic lupus erythematosus; family history of stage 5 CKD or hereditary kidney disease; opportunistic detection of haematuria or proteinuria. | 7.0 | 8.0 | 5.0 | 6.67 | 7.5 | 56 | 46 |
| 29. **Irritable bowel syndrome**  All people presenting with possible irritable bowel syndrome (IBS) symptoms should be assessed and clinically examined for the following 'red flag' indicators and should be referred to secondary care for further investigation if any are present: anaemia; inflammatory markers for inflammatory bowel disease; abdominal masses; rectal masses. If there is significant concern that symptoms may suggest ovarian cancer, a pelvic examination should also be considered. | 7.0 | 8.0 | 5.0 | 6.67 | 7.5 | 56 | 46 |
| 30. **Atopic eczema in children**  Healthcare professionals should offer children with atopic eczema a choice of unperfumed emollients to use every day for moisturising, washing and bathing. This should be suited to the child's needs and preferences, and may include a combination of products or one product for all purposes. Leave-on emollients should be prescribed in large quantities (250-500 g weekly) and easily available to use at nursery, pre-school or school. | 7.0 | 8.0 | 5.0 | 6.67 | 7.5 | 56 | 46 |
| 34. **Antenatal and postnatal mental health**  At a woman's first contact with primary care, at her booking visit and postnatally (usually at 4 to 6 weeks and 3 to 4 months), healthcare professionals (including midwives, obstetricians, health visitors and GPs) should ask two questions to identify possible depression. During the past month, have you often been bothered by feeling down, depressed or hopeless? During the past month, have you often been bothered by having little interest or pleasure in doing things? A third question should be considered if the woman answers 'yes' to either of the initial questions; Is this something you feel you need or want help with? | 7.0 | 8.0 | 5.0 | 6.67 | 7.5 | 56 | 46 |
| 35. **Obesity**  Body mass index (BMI) (adjusted for age and gender) is recommended as a practical estimate of overweight in children and young people, but needs to be interpreted with caution because it is not a direct measure of adiposity. | 7.0 | 8.0 | 5.0 | 6.67 | 7.5 | 56 | 46 |
| 43. **Stable angina**  Assess the person's need for lifestyle advice (for example about exercise, stopping smoking, diet and weight control) and psychological support, and offer interventions as necessary. | 7.0 | 8.0 | 5.0 | 6.67 | 7.5 | 56 | 46 |
| 74. **Composites**  **Chronic kidney disease - PROCESSES OF CARE**  The percentage of patients on the CKD register whose notes have a record of blood pressure in the preceding 15 months. (1 of 4).  The percentage of patients on the CKD register whose notes have a record of a urine albumin:creatinine ratio (or protein:creatinine ratio) test in the preceding 15 months. (2 of 4)  Take the following steps to identify progressive CKD: Obtain a minimum of three GFR estimations over a period of not less than 90 days. In people with a new finding of reduced eGFR, repeat the eGFR within 2 weeks to exclude causes of acute deterioration of GFR , for example, acute kidney injury or initiation of ACE inhibitor/ARB therapy. Define progression as a decline in eGFR of > 5 ml/min/1.73 m2 within 1 year, or > 10 ml/min/1.73 m2 within 5 years. Focus particularly on those in whom a decline of GFR continuing at the observed rate would lead to the need for renal replacement therapy within their lifetime by extrapolating the current rate of decline. (3 of 4)  Encourage people with CKD to take exercise, achieve a healthy weight and stop smoking. (4 of 4)  ‘Surprise’ that this had fallen out of the top 50 so we are including it. | 8.0 | 7.0 | 5.0 | 6.67 | 7.5 | 56 | 46 |
| 101. **Composites**  **Depression – CONTINUATION OF TREATMENT**  Support and encourage a person who has benefited from taking an antidepressant to continue medication for at least 6 months after remission of an episode of depression. Discuss with the person that: this greatly reduces the risk of relapse; antidepressants are not associated with addiction. (1 of 2)  Advise people with depression to continue antidepressants for at least 2 years if they are at risk of relapse. Maintain the level of medication at which acute treatment was effective (unless there is good reason to reduce the dose, such as unacceptable adverse effects) if: they have had two or more episodes of depression in the recent past, during which they experienced significant functional impairment; they have other risk factors for relapse such as residual symptoms, multiple previous episodes, or a history of severe or prolonged episodes or of inadequate response; the consequences of relapse are likely to be severe (for example, suicide attempts, loss of functioning, severe life disruption, and inability to work). ( 2 of 2) | 8.0 | 7.0 | 5.0 | 6.67 | 7.5 | 56 | 46 |
| 125. **Composites**  **Lipid modification - STATINS THERAPY**  Statin therapy is recommended as part of the management strategy for the primary prevention of CVD for adults who have a 20% or greater 10-year risk of developing CVD. This level of risk should be estimated using an appropriate risk calculator, or by clinical assessment for people for whom an appropriate risk calculator is not available or appropriate (for example, older people, people with diabetes or people in high-risk ethnic groups). (1 of 6)  People aged 75 or older should be considered at increased risk of CVD, particularly people who smoke or have raised blood pressure. They are likely to benefit from statin treatment. Assessment and treatment should be guided by the benefits and risks of treatment, informed preference and comorbidities that may make treatment inappropriate. (2 of 6)  Before offering lipid modification therapy for primary prevention, all other modifiable CVD risk factors should be considered and their management optimised if possible. Baseline blood tests and clinical assessment should be performed, and comorbidities and secondary causes of dyslipidaemia should be treated. Assessment should include: smoking status; alcohol consumption; blood pressure (see 'Hypertension', NICE clinical guideline 34); body mass index or other measure of obesity (see 'Obesity', NICE clinical guideline 43); fasting total cholesterol, LDL cholesterol, HDL cholesterol and triglycerides (if fasting levels are not already available); fasting blood glucose; renal function; liver function (transaminases); thyroid-stimulating hormone (TSH) if dyslipidaemia is present. (3 of 6)  Treatment for the primary prevention of CVD should be initiated with simvastatin 40 mg. If there are potential drug interactions, or simvastatin 40 mg is contraindicated, a lower dose or alternative preparation such as pravastatin may be chosen. (4 of 6)  Baseline liver enzymes should be measured before starting a statin. Liver function (transaminases) should be measured within 3 months of starting treatment and at 12 months, but not again unless clinically indicated. (5 of 6)  Higher intensity statins should not routinely be offered to people for the primary prevention of CVD. (6 of 6)  Surprise’ that this had fallen out of the top 50 so we are including it. | 8.0 | 7.0 | 5.0 | 6.67 | 7.5 | 56 | 46 |
| 19. **Chronic kidney disease**  At any given stage of CKD, management should not be influenced solely by age. | 8.0 | 7.0 | 4.5 | 6.5 | 7.5 | 66 | 46 |
| 89. **Composites**  **Stable angina - TREATMENT**  Offer people optimal drug treatment for the initial management of stable angina. Optimal drug treatment consists of one or two anti-anginal drugs as necessary plus drugs for secondary prevention of cardiovascular disease. (1 of 4)  Offer either a beta blocker or a calcium channel blocker as first-line treatment for stable angina. Decide which drug to use based on comorbidities, contraindications and the person's preference. (2 of 4)  Consider aspirin 75 mg daily for people with stable angina, taking into account the risk of bleeding and comorbidities. (3 of 4)  Offer a short-acting nitrate for preventing and treating episodes of angina. Advise people with stable angina: how to administer the short-acting nitrate; to use it immediately before any planned exercise or exertion; that side effects such as flushing, headache and light-headedness may occur, to sit down or find something to hold on to if feeling light-headed. (4 of 4)  Surprise’ that this had fallen out of the top 50 so we are including it. | 7.0 | 8.0 | 4.5 | 6.5 | 7.5 | 66 | 46 |
| 108. **Composites**  **Dyspepsia – IDENTIFICATION AND TREATMENT**  Review medications for possible causes of dyspepsia, for example calcium antagonists, nitrates, theophyllines, bisphosphonates, steroids and NSAIDs. In patients requiring referral suspend NSAID use. (1 of 5)  Offer simple lifestyle advice, including healthy eating, weight reduction and smoking cessation. (2 of 5)  Initial therapeutic strategies for dyspepsia are empirical treatment with a proton pump inhibitor (PPI) or testing for and treating H.pylori. There is currently insufficient evidence to guide which should be offered first. A 2-week washout period following PPI use is necessary before testing for H. pylori with a breath test or a stool antigen test. (3 of 5)  Patients testing positive for H. pylori should be offered eradication therapy. (4 of 5)  Offer older patients (over 80 years of age) the same treatment as younger patients, taking account of any comorbidity and their existing use of medication. (5 of 5) | 6.5 | 8.0 | 5.0 | 6.5 | 7.25 | 66 | 66 |
| 17. **CKD**  People with chronic kidney disease (CKD) in the following groups should normally be referred for specialist assessment: stage 4 and 5 CKD (with or without diabetes); higher levels of proteinuria (ACR greater than or equal to 70 mg/mmol, approximately equivalent to PCR greater than or equal to 100 mg/mmol, or urinary protein excretion greater than or equal to 1 g/24 h) unless known to be due to diabetes and already appropriately treated; proteinuria (ACR greater than or equal to 30 mg/mmol, approximately equivalent to PCR greater than or equal to 50 mg/mmol, or urinary protein excretion greater than or equal to 0.5 g/24 h) together with haematuria; rapidly declining eGFR (> 5 ml/min/1.73 m2 in 1 year, or > 10 ml/min/1.73 m2 within 5 years); hypertension that remains poorly controlled despite the use of at least four antihypertensive drugs at therapeutic doses (see 'Hypertension: management of hypertension in adults in primary care' [NICE clinical guideline 34]); people with, or suspected of having, rare or genetic causes of CKD, suspected renal artery stenosis. | 7.0 | 7.0 | 5.5 | 6.5 | 7 | 66 | 68 |
| 6. **Osteoporosis - secondary prevention of fragility fractures**  The percentage of patients aged 75 years and over with a fragility fracture, who are currently treated with an appropriate bone-sparing agent. | 7.0 | 7.0 | 5.0 | 6.33 | 7 | 70 | 68 |
| 45. **Referral for suspected cancer (urological cancers)**  In a male a patient with or without lower urinary tract symptoms and in whom the prostate is normal on DRE but the age-specific PSA is raised or rising, an urgent referral should be made. In those patients whose clinical state is compromised by other comorbidities, a discussion with the patient or carers and/or a specialist in urological cancer may be more appropriate. | 7.0 | 7.0 | 5.0 | 6.33 | 7 | 70 | 68 |
| 51. **Atrial fibrillation**  In patients with permanent Atrial Fibrillation (AF), who need treatment for rate-control: beta-blockers or rate-limiting calcium antagonists should be the preferred initial monotherapy in all patients; digoxin should only be considered as monotherapy in predominantly sedentary patients. | 7.0 | 7.0 | 5.0 | 6.33 | 7 | 70 | 68 |
| 77. **Composites**  **Non-steroidal anti-inflammatory drugs - NSAIDs**  Monitor glomerular filtration rate (GFR) in people prescribed drugs known to be nephrotoxic, such as calcineurin inhibitors and lithium. Check GFR at least annually in people receiving long-term systemic non-steroidal anti-inflammatory drug (NSAID) trreatment. (1 of 2)  In people with CKD the chronic use of NSAIDs may be associated with progression and acute use is associated with a reversible fall in glomerular filtration rate (GFR). Exercise caution when treating people with CKD with NSAIDs over prolonged periods of time. Monitor the effects on GFR, particularly in people with a low baseline GFR and/or in the presence of other risks for progression. (2 of 2)  Surprise’ that this had fallen out of the top 50 so we are including it. | 7.0 | 7.0 | 5.0 | 6.33 | 7 | 70 | 68 |
| 118. **Composites**  **Irritable bowel syndrome (IBS)**  In people who meet the IBS diagnostic criteria, the following tests should be undertaken to exclude other diagnoses: full blood count (FBC); antibody testing for coeliac disease (endomysial antibodies [EMA]or tissue transglutaminase [TTG]); erythrocyte sedimentation rate (ESR) or plasma viscosity, c-reactive protein (CRP) (1 of 2)  The following tests are not necessary to confirm diagnosis in people who meet the IBS diagnostic criteria: ultrasound; faecal ova and parasite test; rigid/flexible sigmoidoscopy; faecal occult blood; colonoscopy; barium enema; hydrogen breath test (for lactose intolerance and bacterial overgrowth), thyroid function test. (2 of 2) | 7.0 | 7.0 | 5.0 | 6.33 | 7 | 70 | 68 |
| 127. **Composites**  **Mental health (severe and enduring)**  The percentage of patients with schizophrenia, bipolar affective disorder and other psychoses who have a record of alcohol consumption in the preceding 15 months. (1 of 7)  The percentage of patients with schizophrenia, bipolar affective disorder and other psychoses who have a record of BMI in the preceding 15 months. (2 of 7)  The percentage of patients with schizophrenia, bipolar affective disorder and other psychoses who have a record of blood pressure in the preceding 15 months. (3 of 7)  The percentage of patients aged 40 years and over with schizophrenia, bipolar affective disorder and other psychoses who have a record of total cholesterol:hdl ratio in the preceding 15 months. (4 of 7)  The percentage of patients aged 40 years and over with schizophrenia, bipolar affective disorder and other psychoses who have a record of blood glucose or HbA1c in the preceding15 months. (5 of 7)  The percentage of women (aged from 25 to 64 in England and Northern Ireland, from 20 to 60 in Scotland and from 20 to 64 in Wales) with schizophrenia, bipolar affective schizophrenia, bipolar affective disorder and other psychoses whose notes record that a cervical screening test has been performed in the preceding 5 years. (6 of 7)  The percentage of patients on the register who have a comprehensive care plan documented in the records agreed between individuals, their family and/or carers as appropriate. (7 of 7) | 7.0 | 7.0 | 5.0 | 6.33 | 7 | 70 | 68 |
| 129. **Composites**  **Lower back pain**  When offering treatment with an oral NSAID/COX-2 (cyclooxygenase 2) inhibitor, the first choice should be either a standard NSAID or a COX-2 inhibitor. In either case, for people over 45 these should be co-prescribed with a PPI (proton pump inhibitor), choosing the one with the lowest acquisition cost. (1 of 2)  Consider offering tricyclic antidepressants if other medications provide insufficient pain relief. Start at a low dosage and increase up to the maximum antidepressant dosage until therapeutic effect is achieved or unacceptable side effects prevent further increase. (2 of 2) | 7.0 | 7.0 | 5.0 | 6.33 | 7 | 70 | 68 |
| 135. **Composites**  **Respiratory tract infections**  A no antibiotic prescribing strategy or a delayed antibiotic prescribing strategy should be agreed for patients with the following conditions: acute otitis media; acute sore throat/acute pharyngitis/acute tonsillitis; common cold; acute rhinosinusitis, acute cough/acute bronchitis. (1 of 3)  An immediate antibiotic prescription and/or further appropriate investigation and management should only be offered to patients (both adults and children) in the following situations: if the patient is systemically very unwell; if the patient has symptoms and signs suggestive of serious illness and/or complications (particularly pneumonia, mastoiditis, peritonsillar abscess,peritonsillar cellulitis, intraorbital and intracranial complications); if the patient is at high risk of serious complications because of pre-existing comorbidity. This includes patients with significant heart, lung, renal, liver or neuromuscular disease, immunosuppression, cystic fibrosis, and young children who were born prematurely; if the patient is older than 65 years with acute cough and two or more of the following criteria, or older than 80 years with acute cough and one or more of the following criteria: hospitalisation in previous year; type 1 or type 2 diabetes; history of congestive heart failure; current use of oral glucocorticoids. For these patients, the no antibiotic prescribing strategy and the delayed antibiotic prescribing strategy should not be considered. (2 of 3)  Depending on clinical assessment of severity, patients in the following subgroups can also be considered for an immediate antibiotic prescribing strategy (in addition to a no antibiotic or a delayed antibiotic prescribing strategy): bilateral acute otitis media in children younger than 2 years; acute otitis media in children with otorrhoea; acute sore throat/acute pharyngitis/acute tonsillitis when three or more Centor criteria are present. (3 of 3) | 7.0 | 6.0 | 6.0 | 6.33 | 6.5 | 70 | 80 |
| 5. **Osteoporosis - secondary prevention of fragility fractures**  The percentage of patients aged between 50 and 74 years, with a fragility fracture, in whom osteoporosis is confirmed on DXA scan, who are currently treated with an appropriate bone-sparing agent. | 7.0 | 7.0 | 4.5 | 6.17 | 7 | 78 | 68 |
| 36. **Bipolar disorder**  People with bipolar disorder should have an annual physical health review, normally in primary care, to ensure that the following are assessed each year: lipid levels, including cholesterol in all patients over 40 even if there is no other indication of risk; plasma glucose levels; weight; smoking status and alcohol use; blood pressure. | 7.0 | 7.0 | 4.5 | 6.17 | 7 | 78 | 68 |
| 79. **Composites**  **Dementia**  The percentage of patients with a new diagnosis of dementia recorded between the preceding 1 April to 31 March with a record of FBC, calcium, glucose, renal and liver function, thyroid function tests, serum vitamin B12 and folate levels recorded 6 months before or after entering on to the register. (1 of 2)  The percentage of patients diagnosed with dementia whose care has been reviewed in the preceding 15 months. (2 of 2).  Surprise’ that this had fallen out of the top 50 so we are including it. | 8.0 | 6.0 | 4.5 | 6.17 | 7 | 78 | 68 |
| 130. **Composites**  **Neuropathic pain (pharmacological management)**  Offer oral amitriptyline or pregabalin as first-line treatment (but see recommendation 1.11 for people with painful diabetic neuropathy). For amitriptyline: start at 10 mg per day, with gradual upward titration to an effective dose or the person's maximum tolerated dose of no higher than 75 mg per day (higher doses could be considered in consultation with a specialist pain service). (1 of 2)  Offer oral amitriptyline or pregabalin as first-line treatment (but see recommendation 1.11 for people with painful diabetic neuropathy). For pregabalin: start at 150 mg per day (divided into two doses; a lower starting dose may be appropriate for some people), with upward titration to an effective dose or the person's maximum tolerated dose of no higher than 600 mg per day (divided into two doses). (2 of 2) | 6.0 | 7.0 | 5.0 | 6 | 6.5 | 81 | 80 |
| 111. **Composites**  **Dyspepsia with peptic ulcer**  For patients using NSAIDs with diagnosed peptic ulcer, stop the use of NSAIDs where possible. Offer full-dose PPI or H2RA therapy for 2 months to these patients and if H. pylori is present, subsequently offer eradication therapy. (1 of 3)  For patients continuing to take NSAIDs after a peptic ulcer has healed, discuss the potential harm from NSAID treatment. Review the need for NSAID use regularly (at least 6 monthly) and offer a trial of use on a limited, 'as required' basis. Consider dose reduction, substitution of an NSAID with paracetamol, use of an alternative analgesic or low dose ibuprofen (1.2g daily). (2 of 3)  In patients at high risk (previous ulceration) and for whom NSAID continuation is necessary, offer gastric protection or consider substitution to a COX-2 selective NSAID. (3 of 3) | 6.0 | 7.0 | 4.5 | 5.83 | 6.5 | 82 | 80 |
| 28. **Antenatal care**  All women should be informed at the booking appointment about the importance for their own and their baby's health of maintaining adequate vitamin D stores during pregnancy and whilst breastfeeding. In order to achieve this, women may choose to take 10 micrograms of vitamin D per day, as found in the Healthy Start multivitamin supplement. Particular care should be taken to enquire as to whether women at greatest risk are following advice to take this daily supplement. These include: women of South Asian, African, Caribbean or Middle Eastern family origin; women who have limited exposure to sunlight, such as women who are predominantly housebound, or usually remain covered when outdoors; women who eat a diet particularly low in vitamin D, such as women who consume no oily fish, eggs, meat, vitamin D-fortified margarine or breakfast cereal; women with a pre-pregnancy body mass index above 30 kg/m2. | 6.0 | 6.0 | 5.0 | 5.67 | 6 | 83 | 83 |
| 7. **Constipation in children and young people**  Treat constipation with laxatives and a combination of: Negotiated and non-punitive behavioural interventions suited to the child or young person's stage of development. These could include scheduled toileting and support to establish a regular bowel habit, maintenance and discussion of a bowel diary, information on constipation, and use of encouragement and rewards systems. Dietary modifications to ensure a balanced diet and sufficient fluids are consumed. | 4.5 | 5.0 | 5.0 | 4.83 | 4.75 | 84 | 84 |
| 82. **Composites**  **Chronic obstructive pulmonary disease – LIFESTYLE**  An up-to-date smoking history, including pack years smoked (number of cigarettes smoked per day, divided by 20, multiplied by the number of years smoked), should be documented for everyone with COPD. (1 of 2)  Encouraging patients with COPD to stop smoking is one of the most important components of their management. All COPD patients still smoking, regardless of age, should be encouraged to stop, and offered help to do so, at every opportunity. (2 of 2) | 8.0 | 9.0 | 4.0 | 7 | 8.5 | Exclude C 4 or below | |
| 44. **Stable angina**  Consider angiotensin-converting enzyme (ACE) inhibitors for people with stable angina and diabetes. Offer or continue ACE inhibitors for other conditions, in line with relevant NICE guidance. | 8.0 | 8.0 | 4.0 | 6.67 | 8 | Exclude C 4 or below | |
| 100. **Composites**  **Depression – MONITORING**  A person with depression started on antidepressants who is considered to present an increased suicide risk or is younger than 30 years (because of the potential increased prevalence of suicidal thoughts in the early stages of antidepressant treatment for this group) should normally be seen after 1 week and frequently thereafter as appropriate until the risk is no longer considered clinically important. (1 of 3)  For people started on antidepressants who are not considered to be at increased risk of suicide, normally see them after 2 weeks. See them regularly thereafter, for example at intervals of 2 to 4 weeks in the first 3 months, and then at longer intervals if response is good. (2 of 3)  People with depression on long-term maintenance treatment should be regularly re-evaluated, with frequency of contact determined by: comorbid conditions; risk factors for relapse, severity and frequency of episodes of depression. (3 of 3) | 8.0 | 8.0 | 4.0 | 6.67 | 8 | Exclude C 4 or below | |
| 120. **Composites**  **Stroke / TIA**  The percentage of patients with a stroke shown to be non-haemorrhagic, or a history of TIA, who have a record that an anti-platelet agent (aspirin, clopidogrel, dipyridamole or a combination), or an anti-coagulant is being taken. (1 of 2)  The percentage of patients with stroke or TIA who have had influenza immunisation in the preceding 1 September to 31 March. (2 of 2) | 8.0 | 8.0 | 4.0 | 6.67 | 8 | Exclude C 4 or below | |
| 119. **Composites**  **Osteoarthritis**  Healthcare professionals should consider offering paracetamol for pain relief in addition to core treatment; regular dosing may be required. Paracetamol and/or topical non-steroidal anti-inflammatory drugs (NSAIDs) should be considered ahead of oral NSAIDs,cyclo-oxygenase 2 (COX-2) inhibitors or opioids. (1 of 2)  When offering treatment with an oral NSAID/COX-2 inhibitor, the first choice should be either a standard NSAID or a COX-2 inhibitor (other than etoricoxib 60 mg). In either case, these should be co-prescribed with a proton pump inhibitor (PPI), choosing the one with the lowest acquisition cost. (2 of 2) | 8.0 | 7.0 | 4.0 | 6.33 | 7.5 | Exclude C 4 or below | |
| 3. **Epilepsy**  All children, young people and adults with epilepsy should have a regular structured review. In children and young people, this review should be carried out at least yearly (but may be between 3 and 12 months by arrangement) by a specialist. In adults, this review should be carried out at least yearly by either a generalist or specialist, depending on how well the epilepsy is controlled and/or the presence of specific lifestyle issues. | 6 | 8 | 4 | 6 | 7 | Exclude C 4 or below | |
| 33. **Antenatal and postnatal mental health**  At a woman's first contact with services in both the antenatal and the postnatal periods, healthcare professionals (including midwives, obstetricians, health visitors and GPs) should ask questions about: past or present severe mental illness including schizophrenia, bipolar disorder, psychosis in the postnatal period and severe depression; previous treatment by a psychiatrist/specialist mental health team including inpatient care; a family history of perinatal mental illness. Other specific predictors, such as poor relationships with her partner, should not be used for the routine prediction of the development of a mental disorder. | 6.0 | 8.0 | 4.0 | 6 | 7 | Exclude C 4 or below | |
| 39. **Long-acting reversible contraception**  Women requiring contraception should be given information about and offered a choice of all methods, including long-acting reversible contraception (LARC) methods. | 7.0 | 7.0 | 4.0 | 6 | 7 | Exclude C 4 or below | |
| 63. **Diabetes mellitus**  The percentage of patients with diabetes with a diagnosis of proteinuria or micro-albuminuria who are treated with ACE inhibitors (or A2 antagonists). | 8.0 | 8.0 | 3.5 | 6.5 | 8 | Exclude C 4 or below | |
| 88. **Composites**  **Stable angina – EQUITY ISSUES**  Do not investigate or treat symptoms of stable angina differently in men and women or in different ethnic groups. (1 of 2)  Do not exclude people with stable angina from treatment based on their age alone. (2 of 2) | 8.0 | 8.0 | 3.5 | 6.5 | 8 | Exclude C 4 or below | |
| 4. **Lung cancer**  Urgent referral for a chest X-ray should be offered when a patient presents with: haemoptysis, or any of the following unexplained or persistent (that is, lasting more than 3 weeks) symptoms or signs: cough, chest/shoulder pain, dyspnoea, weight loss, chest signs, hoarseness, finger clubbing, features suggestive of metastasis from a lung cancer (for example, in brain, bone, liver or skin), cervical/supraclavicular lymphadenopathy. | 7.0 | 8.0 | 3.5 | 6.17 | 7.5 | Exclude C 4 or below | |
| 110. **Composites**  **Dyspepsia – ENDOSCOPY**  Urgent specialist referral for endoscopic investigation (to be seen within 2 weeks) is indicated for patients of any age with dyspepsia when presenting with any of chronic gastrointestinal bleeding, progressive unintentional weight loss, progressive difficulty swallowing, persistent vomiting, iron deficiency anaemia, epigastric mass or suspicious barium meal. (1 of 2)  Routine endoscopic investigation of patients of any age, presenting with dyspepsia and without alarm signs, is not necessary. However, in patients aged 55 years and older with unexplained and persistent recent onset dyspepsia alone, an urgent referral for endoscopy should be made. (2 of 2) | 7.0 | 7.0 | 3.5 | 5.83 | 7 | Exclude C 4 or below | |
| 91. **Composites**  **Myocardial infarction (secondary prevention)**  **Myocardial infarction – TREATMENT**  All patients who have had an acute MI should be offered treatment with a combination of the following drugs: ACE (angiotensin-converting enzyme) inhibitor; beta-blocker; aspirin, statin. (1 of 5)  Treatment with clopidogrel in combination with low-dose aspirin should be continued for 12 months after the most recent acute episode of non-ST-segment-elevation acute coronary syndrome. Thereafter, standard care, including treatment with low-dose aspirin alone, is recommended unless there are other indications to continue dual antiplatelet therapy. (2 of 5)  Beta-blockers should be initiated as soon as possible when the patient is clinically stable and titrated upwards to the maximum tolerated dose. (3 of 5)  For patients after an MI with left ventricular systolic dysfunction, who are being offered treatment with a beta-blocker, clinicians may prefer to consider treatment with a beta-blocker licensed for use in heart failure. (4 of 5)  ACE inhibitor therapy should be initiated at the appropriate dose, and titrated upwards at short intervals (for example every 1 to 2 weeks) until the maximum tolerated or target dose is reached. (5 of 5) | 8.0 | 9.0 | 3.0 | 6.67 | 8.5 | Exclude C 4 or below | |
| 50. **Myocardial infarction - secondary prevention**  All patients who have had an acute MI should be offered treatment with a combination of the following drugs (Grade A): ACE (angiotensin-converting enzyme) inhibitor; aspirin; beta-blocker ; statin. Surprise’ that this had fallen out of the top 50 – including this rather than Q91 composite. | 8.0 | 8.0 | 3.0 | 6.33 | 8 | Exclude C 4 or below | |
| 56. **Type 2 diabetes – newer agents (partial update of CG66)**  When setting a target HbA1c: involve the person in decisions about their individual HbA1c target level, which may be above that of 6.5% set for people with type 2 diabetes in general; encourage the person to maintain their individual target unless the resulting side effects (including hypoglycaemia) or their efforts to achieve this impair their quality of life; offer therapy (lifestyle and medication) to help achieve and maintain the HbA1c target level; inform a person with a higher HbA1c that any reduction in HbA1c towards the agreed target is advantageous to future health; avoid pursuing highly intensive management to levels of less than 6.5%. | 8.0 | 8.0 | 3.0 | 6.33 | 8 | Exclude C 4 or below | |
| 59. **Type II diabetes**  Add a sulfonylurea as second-line therapy when blood glucose control remains, or becomes, inadequate with metformin. | 8.0 | 8.0 | 3.0 | 6.33 | 8 | Exclude C 4 or below | |
| 70. **Composites**  **Chronic heart failure – EQUITY ISSUES**  The principles of pharmacological management of heart failure should be the same for men and women. (1 of 2)  The management of heart failure should be determined by clinical criteria, irrespective of the age of the patient. (2 of 2) | 8.0 | 8.0 | 3.0 | 6.33 | 8 | Exclude C 4 or below | |
| 9. **Low back pain**  Do not offer X-ray of the lumbar spine for the management of non-specific low back pain. | 8.0 | 5.0 | 3.0 | 5.33 | 6.5 | Exclude C 4 or below | |

Note: Shaded rows indicate recommendations excluded following survey 1 due to receiving an average median rating for room for improvement of 4 or lower.
