# Supplementary material for: Developing ‘high impact’ guideline-based quality indicators for UK primary care: a multi-stage consensus process
Source: BMC Fam Pract. 2015 Oct 28;16:156. doi: 10.1186/s12875-015-0350-6 (PMC4624600; doi:10.1186/s12875-015-0350-6)
Supplement: Additional file 3: — Ratings for the 62 recommendations/composites from Stage 3. (DOCX 47 kb) [file 12875_2015_350_MOESM3_ESM.docx]

Additional File 3: Ratings for the 62 recommendations/composites from Stage 3

|  | Final consensus panel scores | | | | | |  |  |  |  |
| --- | --- | --- | --- | --- | --- | --- | --- | --- | --- | --- |
| Recommendation* | A  Patient burden | B  Potential for patient benefit | C  Room for improvement in current practice | D  Feasible to measure | E  Primary care control | F  Potential for cost savings without harm | Mean ABCDEF | Ranked by Mean ABCDEF | Reasons for rejection  (if in top 20 consensus panel rankings) | |
| Chronic heart failure: Prescribe aspirin for patients with the combination of heart failure and atherosclerotic heart disease. | 8.0 | 9.0 | 5.5 | 8.0 | 8.0 | 8.0 | 7.8 | 1 | Concern that aspirin hard to track as often purchased over the counter; not felt to be of significant clinical importance. | |
| Lipid modification: All people who smoke should be advised to stop. | 8.0 | 9.0 | 5.0 | 8.0 | 7.0 | 9.0 | 7.7 | 2 | Felt to be reasonably well adhered to. | |
| [**Smoking:**](https://www.survey.bris.ac.uk/cgi-bin/gen.pl?manifestid=123410&op=edit&itemid=108850127&editrootitemid=108850127) **The percentage of patients in high risk groups whose notes record smoking status and the offer of support and treatment within preceding 15m [composite].** | 8.0 | 8.0 | 6.0 | 8.0 | 8.0 | 8.0 | 7.7 | 2 |  | |
| Secondary prevention of coronary heart disease (CHD): The percentage of patients with CHD with a record in the preceding 15m that anti-platelet or anti-coagulant therapy is being taken. | 8.0 | 8.0 | 5.0 | 9.0 | 8.0 | 8.0 | 7.7 | 2 | Felt to be reasonably well adhered to. | |
| Secondary prevention of CHD: The percentage of patients with CHD who are currently treated with a beta-blocker. | 8.0 | 8.0 | 5.0 | 9.0 | 8.0 | 8.0 | 7.7 | 2 | Evidence for using beta-blockers in CHD conditions other than post-MI is limited; also, concern that comorbidities may affect prescribing decisions. | |
| Hypertension: The percentage of patients with hypertension in whom the last blood pressure is ≤ 150/90mmHg. | 8.0 | 8.0 | 5.0 | 9.0 | 8.0 | 8.0 | 7.7 | 2 | Composite recommendation below examining differential blood pressure targets depending on age taken forward to Stage 5. | |
| Chronic kidney disease (CKD): The percentage of patients on the CKD register with hypertension and proteinuria who are treated with an ACE-inhibitor or angiotensin receptor blocker. | 8.0 | 8.0 | 5.0 | 8.0 | 8.0 | 8.0 | 7.5 | 7 |  | |
| Diabetes mellitus: The percentage of patients with diabetes in whom the last blood pressure is ≤ 140/80mmHg. | 8.0 | 8.0 | 5.0 | 9.0 | 7.0 | 8.0 | 7.5 | 7 |  | |
| [**Smoking: The percentage of patients aged ≥15years**](https://www.survey.bris.ac.uk/cgi-bin/gen.pl?manifestid=123410&op=edit&itemid=108850131&editrootitemid=108850131) **whose notes record smoking status, and if so, an offer of support and treatment within the preceding 27m [composite].** | 7.5 | 8.5 | 5.0 | 9.0 | 7.0 | 8.0 | 7.5 | 7 | Felt to be reasonably well adhered to. | |
| Peripheral arterial disease (PAD): The percentage of patients with PAD with a record in the preceding 15 months that aspirin or alternative anti-platelet is being taken. | 7.0 | 8.0 | 5.0 | 9.0 | 8.0 | 8.0 | 7.5 | 7 | Felt to be reasonably well adhered to. | |
| [**Type 2 diabetes: 9 annual processes of care**](https://www.survey.bris.ac.uk/cgi-bin/gen.pl?manifestid=123410&op=edit&itemid=108848017&editrootitemid=108848017) **i.e. measurement of blood pressure, lipids, renal function, urine ACR, glycaemic control, BMI, smoking status, plus foot and eye checks [composite].** | 8.0 | 9.0 | 4.5 | 8.0 | 8.0 | 7.0 | 7.4 | 11 |  | |
| [**Type 2 diabetes:**](https://www.survey.bris.ac.uk/cgi-bin/gen.pl?manifestid=123410&op=edit&itemid=108848109&editrootitemid=108848109) **achievement of target levels for blood pressure, cholesterol and glycaemic control [composite].** | 8.0 | 8.0 | 5.5 | 8.0 | 8.0 | 7.0 | 7.4 | 11 |  | |
| [**Hypertension:**](https://www.survey.bris.ac.uk/cgi-bin/gen.pl?manifestid=123410&op=edit&itemid=108848761&editrootitemid=108848761) **blood pressure targets in those under/over 80 years of age [composite].** | 8.0 | 8.0 | 5.5 | 8.0 | 8.0 | 7.0 | 7.4 | 11 |  | |
| [**Lipid modification:**](https://www.survey.bris.ac.uk/cgi-bin/gen.pl?manifestid=123410&op=edit&itemid=108849451&editrootitemid=108849451) **Assessment of risk using cardiovascular risk calculators [composite].** | 8.0 | 8.0 | 5.5 | 8.0 | 8.0 | 7.0 | 7.4 | 11 | Felt recommendation may conflict with policy on vascular risk checks. | |
| Chronic Kidney Disease (CKD): People with CKD are assessed for cardiovascular risk. | 8.0^ | 7.0^ | 5.5^ | 8.0^ | 8.0^ | 8.0^ | 7.4 | 11 | Other CKD processes of care felt to be more important and included as below. | |
| Type 2 diabetes: Cardiovascular risk assessment and subsequent statin therapy where indicated. | 8.0 | 8.0 | 4.5 | 8.0 | 8.0 | 8.0 | 7.4 | 11 |  | |
| Type 2 diabetes: Integrate dietary advice with a personalised diabetes management plan. | 8.0 | 9.0 | 6.0 | 6.0 | 7.0 | 8.0 | 7.3 | 17 |  | |
| Chronic obstructive pulmonary disease (COPD): Spirometry should be performed in patients who are over 35 years, current/ex-smokers and have a chronic cough. | 8.0 | 7.0 | 7.0 | 7.0 | 8.0 | 7.0 | 7.3 | 17 | Overlap with included composite COPD recommendation below. | |
| Type 2 diabetes: For a person on dual therapy who is markedly hyperglycaemic, consider starting insulin therapy in preference to adding other drugs to control blood glucose. | 8.0 | 8.0 | 6.0 | 8.0 | 7.0 | 7.0^ | 7.3 | 17 | Subsequently amalgamated with Type 2 diabetes recommendation below advising starting insulin therapy if oral therapy not adequate to achieve glycaemic control. | |
| Chronic Obstructive Pulmonary Disease ([**COPD): D**](https://www.survey.bris.ac.uk/cgi-bin/gen.pl?manifestid=123410&op=edit&itemid=108837267&editrootitemid=108837267)**iagnosis of COPD, through use of spirometry and chest radiograph [composite].** | 8.0 | 8.0 | 6.0 | 7.0 | 8.0 | 7.0 | 7.3 | 17 |  | |
| Chronic Kidney Disease (CKD): People with higher levels of proteinuria are enabled to safely maintain their blood pressure within target range. | 7.0 | 8.0 | 6.0 | 8.0 | 7.0 | 8.0 | 7.3 | 17 | Overlap with included recommendation on the treatment of hypertension in CKD above. | |
| Hypertension: If hypertension not diagnosed, measure clinic blood pressure at least every 5 years; consider more frequently if close to 140/90 mmHg. | 8.0 | 8.0 | 5.0 | 8.0 | 8.0 | 7.0 | 7.3 | 17 | Composite recommendation examining blood pressure targets in those already diagnosed felt to be more important and included as above. | |
| Myocardial infarction (MI): All patients who have had an acute MI should be offered specific combination drug treatment. | 8.0 | 8.0 | 3.0 | 9.0 | 8.0^ | 8.0 | 7.3 | 17 |  | |
| Myocardial infarction (MI): All patients who smoke and desire to quit should be offered support and referral to intensive support service. | 8.0 | 9.0 | 4.5 | 7.0 | 7.0 | 8.0 | 7.3 | 24 |  | |
| [**Chronic heart failure: lifestyle issues, preventative measures and monitoring**](https://www.survey.bris.ac.uk/cgi-bin/gen.pl?manifestid=123410&op=edit&itemid=108833756&editrootitemid=108833756)**. 7 recommendations, including smoking and alcohol advice, vaccinations and renal monitoring [composite].** | 8.0 | 8.0 | 5.5 | 7.0 | 8.0 | 7.0 | 7.3 | 24 |  | |
| Chronic heart failure: Offer both ACE-inhibitors and beta-blockers to all patients with heart failure due to left ventricular systolic dysfunction. | 8.0 | 7.0 | 5.5 | 8.0 | 7.0 | 8.0 | 7.3 | 24 |  | |
| Myocardial infarction (MI): Advice on physical activity should involve discussion of current and past activity levels. | 8.0 | 9.0 | 6.5 | 4.0 | 7.5 | 8.0 | 7.2 | 27 |  | |
| Stroke: People who have had a suspected transient ischemic attack who are at high risk of stroke should have aspirin, specialist assessment, and measures for secondary prevention. | 8.0 | 9.0 | 5.0 | 6.0 | 7.0 | 8.0 | 7.2 | 27 |  | |
| [Chronic Kidney Disease (CKD):](https://www.survey.bris.ac.uk/cgi-bin/gen.pl?manifestid=123410&op=edit&itemid=108834181&editrootitemid=108834181) **blood pressure and urinary protein excretion targets, and appropriate drug therapy [composite].** | 8.0 | 8.0 | 6.0 | 7.0 | 7.0 | 7.0 | 7.2 | 27 |  | |
| [Chronic Obstructive Pulmonary Disease (COPD):](https://www.survey.bris.ac.uk/cgi-bin/gen.pl?manifestid=123410&op=edit&itemid=108837375&editrootitemid=108837375) **Appropriate use of drugs, inhalers and vaccinations [composite].** | 8.0 | 8.0 | 6.0 | 7.0 | 7.0 | 7.0 | 7.2 | 27 |  | |
| Atrial fibrillation (AF): recommendations concerning use of anti-coagulants in AF [composite]. | 8.0 | 8.0 | 5.0 | 8.0 | 7.0 | 7.0 | 7.2 | 27 |  | |
| Type 1 diabetes: Assessment of arterial risk and early markers of eye, kidney, nerve and foot damage [composite]. | 8.0 | 9.0 | 5.5 | 7.0 | 6.0 | 7.0 | 7.1 | 32 |  | |
| Hypertension: lifestyle advice and monitoring of cholesterol and urinary protein excretion [composite]. | 8.0 | 9.0 | 5.5 | 7.0 | 6.0 | 7.0 | 7.1 | 32 |  | |
| [**Chronic heart failure:**](https://www.survey.bris.ac.uk/cgi-bin/gen.pl?manifestid=123410&op=edit&itemid=108832961&editrootitemid=108832961) **Measurement of serum natriuretic peptides and referral where appropriate [composite].** | 8.0 | 8.0 | 6.0 | 6.5^ | 7.0 | 7.0 | 7.1 | 32 |  | |
| Chronic Obstructive Pulmonary Disease (COPD): Pulmonary rehabilitation should be offered to all patients who consider themselves functionally disabled by COPD. | 8.0 | 7.0 | 6.5 | 7.0 | 7.0 | 7.0 | 7.1 | 32 |  | |
| Type 2 diabetes: Start insulin therapy if oral therapy not adequate to achieve glycaemic control. | 8.0 | 8.0 | 5.5 | 7.0 | 7.0 | 7.0 | 7.1 | 32 | NB Subsequently amalgamated with Type 2 diabetes recommendation above concerning initiation of insulin for a patient on dual therapy who is markedly hyperglycaemic. | |
| [**Lipid modification:**](https://www.survey.bris.ac.uk/cgi-bin/gen.pl?manifestid=123410&op=edit&itemid=108849464&editrootitemid=108849464) **Clinical assessment and statin therapy [composite].** | 8.0 | 8.0 | 4.5 | 7.0 | 8.0 | 7.0 | 7.1 | 32 |  | |
| Depression in adults: For people with moderate or severe depression, provide a combination of medication and psychological intervention. | 8.0 | 9.0 | 5.0 | 6.0 | 7.0 | 7.0 | 7.0 | 38 |  | |
| Chronic Kidney Disease (CKD): Offer people testing for CKD if they are high risk. | 7.0 | 8.0 | 5.0 | 7.0 | 8.0 | 7.0 | 7.0 | 38 |  | |
| Stable angina: Assess patient’s need for lifestyle advice and psychological support, and offer interventions as necessary. | 7.0 | 8.0 | 5.0 | 7.0 | 7.0 | 8.0 | 7.0 | 38 |  | |
| Osteoporosis: The percentage of patients aged ≥75years with a fragility fracture, who are treated with an appropriate bone-sparing agent. | 7.0 | 7.0 | 5.0 | 8.0 | 8.0 | 7.0 | 7.0 | 38 |  | |
| Chronic heart failure: Perform an ECG and consider various tests to evaluate possible aggravating factors or alternative diagnoses. | 8.0 | 7.5 | 6.0 | 7.0 | 7.0 | 6.0 | 6.9 | 42 |  | |
| [**Hypertension:**](https://www.survey.bris.ac.uk/cgi-bin/gen.pl?manifestid=123410&op=edit&itemid=108848605&editrootitemid=108848605) **Antihypertensive drug treatment and assessment of potential target organ damage [composite].** | 8.0 | 8.0 | 4.5 | 6.0 | 8.0 | 7.0 | 6.9 | 42 |  | |
| [Myocardial infarction (MI):](https://www.survey.bris.ac.uk/cgi-bin/gen.pl?manifestid=123410&op=edit&itemid=108841142&editrootitemid=108841142) **Recommendations on exercise, smoking, alcohol and dietary advice [composite].** | 8.0 | 9.0 | 5.0 | 5.0 | 6.0 | 8.0 | 6.8 | 44 |  | |
| Ovarian cancer: Use of appropriate assessments and tests with presenting symptoms. | 7.5 | 9.0 | 5.5 | 4.0 | 7.0 | 8.0 | 6.8 | 44 |  | |
| Chronic Kidney Disease (CKD): Encourage people with CKD to take exercise, achieve a healthy weight and stop smoking. | 8.0 | 8.0 | 5.0 | 4.0 | 7.0 | 9.0 | 6.8 | 44 |  | |
| [**Chronic heart failure:**](https://www.survey.bris.ac.uk/cgi-bin/gen.pl?manifestid=123410&op=edit&itemid=108787095&editrootitemid=108787095) **Recommendations about appropriate drug treatment [composite].** | 7.5 | 7.0 | 6.5 | 7.0 | 6.0 | 7.0 | 6.8 | 44 |  | |
| [Chronic Kidney Disease (CKD):](https://www.survey.bris.ac.uk/cgi-bin/gen.pl?manifestid=123410&op=edit&itemid=108834170&editrootitemid=108834170) **Measurement of blood pressure**, **urinary protein excretion**, **and lifestyle advice [composite].** | 8.0 | 7.0 | 5.0 | 7.0 | 7.0 | 7.0 | 6.8 | 44 |  | |
| [**Lipid modification:**](https://www.survey.bris.ac.uk/cgi-bin/gen.pl?manifestid=123410&op=edit&itemid=108849682&editrootitemid=108849682) **Cardiovascular risk assessment, measurement of liver function, and use of appropriate statins [composite].** | 8.0 | 7.0 | 5.0 | 7.0 | 7.0 | 7.0 | 6.8 | 44 |  | |
| [**Opioids in palliative care**](https://www.survey.bris.ac.uk/cgi-bin/gen.pl?manifestid=123410&op=edit&itemid=108850051&editrootitemid=108850051)**: Dosage, use of morphine, and laxatives [composite].** | 7.0 | 8.0 | 5.5 | 6.0 | 7.0 | 7.0 | 6.8 | 50 |  | |
| [**Stable angina:**](https://www.survey.bris.ac.uk/cgi-bin/gen.pl?manifestid=123410&op=edit&itemid=108838054&editrootitemid=108838054) **Recommendations concerning optimal drug treatment [composite].** | 7.0 | 8.0 | 4.5 | 7.0 | 7.0 | 7.0 | 6.8 | 50 |  | |
| Chronic heart failure: Monitoring of heart failure status. | 8.0 | 7.0 | 6.0 | 5.0 | 7.0 | 7.0 | 6.7 | 52 |  | |
| [**Non-steroidal anti-inflammatory drugs**](https://www.survey.bris.ac.uk/cgi-bin/gen.pl?manifestid=123410&op=edit&itemid=108834321&editrootitemid=108834321) **(NSAIDs): Use of NSAIDs and monitoring of potential side-effects [composite].** | 7.0 | 7.0 | 5.0 | 6.0 | 8.0 | 7.0^ | 6.7 | 52 | NB Initially selected, but later replaced by composite high-risk prescribing indicator used in Dreischulte et al [28]. | |
| [**Depression in adults:**](https://www.survey.bris.ac.uk/cgi-bin/gen.pl?manifestid=123410&op=edit&itemid=108842135&editrootitemid=108842135) **Recommendations concerning severity-appropriate treatment of depression [composite].** | 8.0 | 8.0 | 6.5 | 4.0 | 6.0 | 7.0 | 6.6 | 54 |  | |
| [**Myocardial infarction (MI):**](https://www.survey.bris.ac.uk/cgi-bin/gen.pl?manifestid=123410&op=edit&itemid=108838718&editrootitemid=108838718) **Cardiac rehabilitation should be accessible and offered to all patients [composite].** | 8.0 | 8.0 | 5.5 | 6.0 | 5.0 | 7.0 | 6.6 | 54 |  | |
| [**Depression in adults:**](https://www.survey.bris.ac.uk/cgi-bin/gen.pl?manifestid=123410&op=edit&itemid=108841969&editrootitemid=108841969) **Be alert to possible depression and consider using tools to aid diagnosis [composite].** | 8.0 | 7.0 | 5.5 | 5.0 | 8.0 | 6.0 | 6.6 | 54 |  | |
| [Chronic Kidney Disease (CKD):](https://www.survey.bris.ac.uk/cgi-bin/gen.pl?manifestid=123410&op=edit&itemid=108834170&editrootitemid=108834170) Refer for specialist assessment people with CKD at high risk of deterioration. | 7.0 | 7.0 | 5.5 | 6.0 | 7.0 | 7.0 | 6.6 | 54 |  | |
| [**Dementia**](https://www.survey.bris.ac.uk/cgi-bin/gen.pl?manifestid=123410&op=edit&itemid=108836802&editrootitemid=108836802)**: The percentage of patients with dementia whose care has been reviewed in preceding 15months and has had baseline tests [composite].** | 8.0 | 6.0 | 4.5 | 8.0 | 7.0 | 6.0 | 6.6 | 54 |  | |
| [Chronic Obstructive Pulmonary Disease (COPD):](https://www.survey.bris.ac.uk/cgi-bin/gen.pl?manifestid=123410&op=edit&itemid=108837538&editrootitemid=108837538) **Recommendations about lifestyle advice and regular review [composite].** | 8.0 | 8.0 | 6.0 | 4.0 | 7.0 | 6.0 | 6.5 | 59 |  | |
| [**Depression in adults: Recommendations on use of drugs and psychological interventions**](https://www.survey.bris.ac.uk/cgi-bin/gen.pl?manifestid=123410&op=edit&itemid=108841969&editrootitemid=108841969) **[composite].** | 8.0 | 8.0 | 6.0 | 4.0 | 6.0 | 6.0 | 6.3 | 60 |  | |
| [**Coeliac disease**](https://www.survey.bris.ac.uk/cgi-bin/gen.pl?manifestid=123410&op=edit&itemid=108834467&editrootitemid=108834467)**: Offer serological testing for coeliac disease to people with at risk conditions or specified symptoms [composite].** | 6.0 | 8.0 | 6.5 | 3.0 | 7.0 | 7.0^ | 6.3 | 61 |  | |
| Dementia: People with dementia should not be excluded from any services because of their diagnosis, age or coexisting learning difficulties. | 8.0 | 8.0 | 5.0 | 3.0 | 5.0 | 5.0 | 5.7 | 62 |  | |
| **Mean ‘median’ ratings with standard deviation (SD)** | 7.8  (0.43) | 8.0  (0.68) | 5.4  (0.68) | 6.8  (1.57) | 7.2  (0.76) | 7.3  (0.73) | 7.1  (0.40) |  |  | |

*Space prevents full text of each recommendation – illustrative summaries are provided in the table

^One missing value

Shaded rows indicate those recommendations selected to be taken forward to Stage 5
