# Supplementary material for: Developing ‘high impact’ guideline-based quality indicators for UK primary care: a multi-stage consensus process
Source: BMC Fam Pract. 2015 Oct 28;16:156. doi: 10.1186/s12875-015-0350-6 (PMC4624600; doi:10.1186/s12875-015-0350-6)
Supplement: Additional file 4 — Folder containing SystmOne™ search algorithms. (ZIP 12.7 mb) [file 12875_2015_350_MOESM4_ESM.zip › Aspire S1 diagrams tw edired/10D1-10 (DM processes #71).pdf]

———	Mandatory In
- - - -	Optional In
....	Not In

10D1-10. Type 2 Diabetic - Register
ASPIRE Study / 10

Has a Read code of Type II diabetes mellitus (X40J5) or one of its children

- Selecting only the most recent matching code
- Without a more recent Read code in...Read Codes and Children:
  Type I diabetes mellitus (X40J4)

Date of Read code before 01 Apr 2013

Registered before 01 Apr 2013

Where patient is registered at General Practice
