# Supplementary material for: Developing ‘high impact’ guideline-based quality indicators for UK primary care: a multi-stage consensus process
Source: BMC Fam Pract. 2015 Oct 28;16:156. doi: 10.1186/s12875-015-0350-6 (PMC4624600; doi:10.1186/s12875-015-0350-6)
Supplement: Additional file 4 — Folder containing SystmOne™ search algorithms. (ZIP 12.7 mb) [file 12875_2015_350_MOESM4_ESM.zip › Aspire S1 diagrams tw edired/10N10 (DM processes #71).pdf]

Mandatory In
Optional In
Not In

10N10. ALL Numerators 1-9
ASPIRE Study / 10

Registered before 01 Apr 2013
Where patient is registered at General Practice

- IN → **10N9. Type 2 diabetic and Smoking Status recorded**
  ASPIRE Study / 10
  Registered before 01 Apr 2013
  Where patient is registered at General Practice

  - IN → **10D1-10. Type 2 Diabetic - Register**
    ASPIRE Study / 10
    Has a Read code of Type II diabetes mellitus (X40J5) or one of its children
    - Selecting only the most recent matching code
    - Without a more recent Read code in...Read Codes and Children:
      Type I diabetes mellitus (X40J4)

    Date of Read code before 01 Apr 2013
    Registered before 01 Apr 2013
    Where patient is registered at General Practice

  - AND IN → **Smoking Status**
    ASPIRE Study / 10
    Registered before 01 Apr 2013
    Where patient is registered at General Practice

    - IN → **Current Non-Smoker Status**
      ASPIRE Study / 10
      Has a Read code of Non-smoker (Ub0oq) or one of its children
      Date of Read code between 01 Jan 2011 and 31 Mar 2013
      Where patient is registered at General Practice

    - OR IN → **Smoking Status**
      ASPIRE Study / 10
      Has a Read code in the SMOK (Smoking habit codes) QOF cluster
      Show read codes in cluster SMOK.
      Date of Read code between 01 Jan 2012 and 31 Mar 2013
      Where patient is registered at General Practice

- AND IN → **10N8. Type 2 Diabetic and BMI Recorded**
  ASPIRE Study / 10
  Registered before 01 Apr 2013
  Where patient is registered at General Practice

  - IN → **10D1-10. Type 2 Diabetic - Register**
    ASPIRE Study / 10
    Has a Read code of Type II diabetes mellitus (X40J5) or one of its children
    - Selecting only the most recent matching code
    - Without a more recent Read code in...Read Codes and Children:
      Type I diabetes mellitus (X40J4)

    Date of Read code before 01 Apr 2013
    Registered before 01 Apr 2013
    Where patient is registered at General Practice

  - AND IN → **BMI**
    ASPIRE Study / 10
    Has a Read code in the BMI (BMI codes) QOF cluster
    Show read codes in cluster BMI.
    Date of Read code between 01 Jan 2012 and 31 Mar 2013
    Registered before 01 Apr 2013
    Where patient is registered at General Practice

Practice

AND IN

10N7. Type 2 diabetic and Retinal Screening Codes
ASPIRE Study / 10

Registered before 01 Apr 2013
Where patient is registered at General Practice

> IN
>
> **10D1-10. Type 2 Diabetic - Register**
> ASPIRE Study / 10
>
> Has a Read code of Type II diabetes mellitus (X40J5) or one of its children
> - Selecting only the most recent matching code
> - Without a more recent Read code in...Read Codes and Children:
>   Type I diabetes mellitus (X40J4)
>
> Date of Read code before 01 Apr 2013
> Registered before 01 Apr 2013
> Where patient is registered at General Practice
>
> AND IN
>
> **Retinal Screening Codes (last 15 months)**
> ASPIRE Study / 10
>
> Has a Read code in the RET (Retinal screening codes) QOF cluster
> Show read codes in cluster RET.
> Date of Read code between 01 Jan 2012 and 31 Mar 2013
> Where patient is registered at General Practice

AND IN

10N6. Tye 2 Diabetic and foot review / neuropathy coding
ASPIRE Study / 10

Registered before 01 Apr 2013
Where patient is registered at General Practice

> IN
>
> **10D1-10. Type 2 Diabetic - Register**
> ASPIRE Study / 10
>
> Has a Read code of Type II diabetes mellitus (X40J5) or one of its children
> - Selecting only the most recent matching code
> - Without a more recent Read code in...Read Codes and Children:
>   Type I diabetes mellitus (X40J4)
>
> Date of Read code before 01 Apr 2013
> Registered before 01 Apr 2013
> Where patient is registered at General Practice
>
> AND IN
>
> **Neuropathy coding or foot risk clasification**
> ASPIRE Study / 10
>
> Where patient is registered at General Practice
>
> > IN
> >
> > **Foot Risk Classification**
> > ASPIRE Study / 10
> >
> > Has a Read code in the FRC (Foot Risk Classification) QOF cluster
> > Show read codes in cluster FRC.
> > Date of Read code between 01 Jan 2012 and 31 Mar 2013
> > Where patient is registered at General Practice
> >
> > OR IN
> >
> > **Neuropathy testing codes**
> > ASPIRE Study / 10
> >
> > Has a Read code in the NPT (Neuropathy testing codes) QOF cluster
> > Show read codes in cluster NPT.
> > Date of Read code between 01 Jan 2012 and 31 Mar 2013
> > Where patient is registered at General Practice

AND IN

10N5. Type 2 Diabetic and eGFR or serum creatinine level recorded
ASPIRE Study / 10

Registered before 01 Apr 2013

Where patient is registered at General Practice

IN

10D1-10. Type 2 Diabetic - Register
ASPIRE Study / 10

Has a Read code of Type II diabetes mellitus (X40J5) or one of its children
- Selecting only the most recent matching code
- Without a more recent Read code in...Read Codes and Children:
  Type I diabetes mellitus (X40J4)

Date of Read code before 01 Apr 2013
Registered before 01 Apr 2013
Where patient is registered at General Practice

AND IN

eGFR or Serum Creatinine Level recorded
ASPIRE Study / 10

Where patient is registered at General Practice

IN

Serum Creatinine level in the last 15m
ASPIRE Study / 10

Has a Read code in the CRE (Codes for serum creatinine) QOF cluster
Show read codes in cluster CRE.
Date of Read code between 01 Jan 2012 and 31 Mar 2013
Registered before 01 Apr 2013

OR IN

eGFR cluster in the last 15 months
ASPIRE Study / 10

Has numeric reading in the EGFR (Codes for estimated glomerular filtration rate) nGMS cluster
Date of numeric reading between 01 Jan 2012 and 31 Mar 2013
Registered before 01 Apr 2013

AND IN

10N4. Type 2 Diabetics and ACR or PCR or Proteinuria
ASPIRE Study / 10

Registered before 01 Apr 2013
Where patient is registered at General Practice

IN

10D1-10. Type 2 Diabetic - Register
ASPIRE Study / 10

Has a Read code of Type II diabetes mellitus (X40J5) or one of its children
- Selecting only the most recent matching code
- Without a more recent Read code in...Read Codes and Children:
  Type I diabetes mellitus (X40J4)

Date of Read code before 01 Apr 2013
Registered before 01 Apr 2013
Where patient is registered at General Practice

AND IN

ACR or PCR or Proteinuria Coded
ASPIRE Study / 10

Where patient is registered at General Practice

IN

ACR or PCR
ASPIRE Study / 10

Where patient is registered at General Practice

IN

ACR (last 15 months)
ASPIRE Study / 10

Has a Urine albumin/creatinine ratio
Date of numeric reading between 01 Jan 2012 and 31 Mar 2013
Registered before 01 Apr 2013

OR IN

PCR in the last 15 months
ASPIRE Study / 10

Has a Urine porphyrin/creatinine ratio
[illegible]

Date of numeric reading between 01 Jan 2012 and 31 Mar 2013
Where patient is registered at General Practice

OR IN

Proteinuria in the last 15 months
ASPIRE Study / 10

- Has a Read code of Proteinuria (X30Q1) or one of its children
- Date of Read code between 01 Jan 2012 and 31 Mar 2013
- Registered before 01 Apr 2013

AND IN

10N3. Type 2 Diabetics and full lipid profile
ASPIRE Study / 10

- Where patient is registered at General Practice

IN

10D1-10. Type 2 Diabetic - Register
ASPIRE Study / 10

- Has a Read code of Type II diabetes mellitus (X40J5) or one of its children
  - Selecting only the most recent matching code
  - Without a more recent Read code in...Read Codes and Children:
    Type I diabetes mellitus (X40J4)
- Date of Read code before 01 Apr 2013
- Registered before 01 Apr 2013
- Where patient is registered at General Practice

AND IN

CHOL2 in the last 15 months
ASPIRE Study / 10

- Has a Read code in the CHOL2 (Total cholesterol codes with a value) QOF cluster
  Show read codes in cluster CHOL2.
- Date of Read code between 01 Jan 2012 and 31 Mar 2013
- Registered before 01 Apr 2013

AND IN

10N2. Type 2 Diabetics and HaA1c
ASPIRE Study / 10

- Registered before 01 Apr 2013
- Where patient is registered at General Practice

IN

10D1-10. Type 2 Diabetic - Register
ASPIRE Study / 10

- Has a Read code of Type II diabetes mellitus (X40J5) or one of its children
  - Selecting only the most recent matching code
  - Without a more recent Read code in...Read Codes and Children:
    Type I diabetes mellitus (X40J4)
- Date of Read code before 01 Apr 2013
- Registered before 01 Apr 2013
- Where patient is registered at General Practice

AND IN

HbA1c Cluster (last 15M)
ASPIRE Study / 10

- Has numeric reading in the IFCCHBA (IFCC HbA1c codes) nGMS cluster
- Date of numeric reading between 01 Jan 2012 and 31 Mar 2013
- Where patient is registered at General Practice

AND IN

10N1. Type 2 Diabetes and BP recorded
ASPIRE Study / 10

- Registered before 01 Apr 2013
- Where patient is registered at General Practice

IN

10D1-10. Type 2 Diabetic - Register
ASPIRE Study / 10

- Has a Read code of Type II diabetes mellitus (X40J5) or one of its children
  - Selecting only the most recent matching code

- Without a more recent Read code in...Read Codes and Children:
  Type I diabetes mellitus (X40J4)

Date of Read code before 01 Apr 2013
Registered before 01 Apr 2013
Where patient is registered at General Practice

AND IN

BP
ASPIRE Study / 10

Registered before 01 Apr 2013
Where patient is registered at General Practice

IN

DM31 - Achievement: BP exists in the last 15 months
ASPIRE Study / 10

BP Has a BP reading
Date of BP reading between 01 Jan 2012 and 31 Mar 2013
Registered before 01 Apr 2013
Where patient is registered at General Practice

OR IN

DM31 - Achievement: BP Systolic and Diastolic valid in the last 15 months
ASPIRE Study / 10

Registered before 01 Apr 2013
Where patient is registered at General Practice

IN

DM31 - Achievement: BP Systolic exists in the last 15 months
ASPIRE Study / 10

Has numeric reading in the BPSYS (BP systolic codes) nGMS cluster
Date of numeric reading between 01 Jan 2012 and 31 Mar 2013
Registered before 01 Apr 2013
Where patient is registered at General Practice

AND IN

DM31 - Achievement: BP Diastolic exists in the last 15 months
ASPIRE Study / 10

Has numeric reading in the BPDIA (BP diastolic codes) nGMS cluster
Date of numeric reading between 01 Jan 2012 and 31 Mar 2013
Registered before 01 Apr 2013
Where patient is registered at General Practice
