# Supplementary material for: Developing ‘high impact’ guideline-based quality indicators for UK primary care: a multi-stage consensus process
Source: BMC Fam Pract. 2015 Oct 28;16:156. doi: 10.1186/s12875-015-0350-6 (PMC4624600; doi:10.1186/s12875-015-0350-6)
Supplement: Additional file 4 — Folder containing SystmOne™ search algorithms. (ZIP 12.7 mb) [file 12875_2015_350_MOESM4_ESM.zip › Aspire S1 diagrams tw edired/10N2 (DM processes #71).pdf]

——	Mandatory In
- - - -	Optional In
....	Not In

10N2. Type 2 Diabetics and HaA1c
ASPIRE Study / 10

Registered before 01 Apr 2013
Where patient is registered at General Practice

IN

10D1-10. Type 2 Diabetic - Register
ASPIRE Study / 10

Has a Read code of Type II diabetes mellitus (X40J5) or one of its children
- Selecting only the most recent matching code
- Without a more recent Read code in...Read Codes and Children:
Type I diabetes mellitus (X40J4)

Date of Read code before 01 Apr 2013
Registered before 01 Apr 2013
Where patient is registered at General Practice

AND IN

HbA1c Cluster (last 15M)
ASPIRE Study / 10

Has numeric reading in the IFCCHBA (IFCC HbA1c codes) nGMS cluster
Date of numeric reading between 01 Jan 2012 and 31 Mar 2013
Where patient is registered at General Practice
