# Supplementary material for: Developing ‘high impact’ guideline-based quality indicators for UK primary care: a multi-stage consensus process
Source: BMC Fam Pract. 2015 Oct 28;16:156. doi: 10.1186/s12875-015-0350-6 (PMC4624600; doi:10.1186/s12875-015-0350-6)
Supplement: Additional file 4 — Folder containing SystmOne™ search algorithms. (ZIP 12.7 mb) [file 12875_2015_350_MOESM4_ESM.zip › Aspire S1 diagrams tw edired/11N3 (DM outcomes #73).pdf]

——	Mandatory In
- - - -	Optional In
....	Not In

11N3. Type 2 Diabetic and serum cholesterol =<5
ASPIRE Study / 11

Registered before 01 Apr 2013
Where patient is registered at General Practice

IN

11D1-3 + 5. Type 2 Diabetic - Register
ASPIRE Study / 11

Has a Read code of Type II diabetes mellitus (X40J5) or one of its children

- Selecting only the most recent matching code
- Without a more recent Read code in...Read Codes and Children:
Type I diabetes mellitus (X40J4)

Date of Read code before 01 Apr 2013
Registered before 01 Apr 2013
Where patient is registered at General Practice

AND IN

Serum Cholesterol =<5
ASPIRE Study / 11

Has a Serum cholesterol level <= 5.0 mmol/L
Date of numeric reading between 01 Jan 2012 and 31 Mar 2013
Registered before 01 Apr 2013
Where patient is registered at General Practice
