# Supplementary material for: Developing ‘high impact’ guideline-based quality indicators for UK primary care: a multi-stage consensus process
Source: BMC Fam Pract. 2015 Oct 28;16:156. doi: 10.1186/s12875-015-0350-6 (PMC4624600; doi:10.1186/s12875-015-0350-6)
Supplement: Additional file 4 — Folder containing SystmOne™ search algorithms. (ZIP 12.7 mb) [file 12875_2015_350_MOESM4_ESM.zip › Aspire S1 diagrams tw edired/11N4 (DM outcomes #73).pdf]

——	Mandatory In
- - - -	Optional In
....	Not In

11N4. Type 2 Diabetic and Type 2 diabetic with ACR, diabetic retinopathy, Stroke, MI and BP <130/80
ASPIRE Study / 11

Registered before 01 Apr 2013
Where patient is registered at General Practice

IN

11D4. Type 2 Diabetic and either ACR, diabetic retinopathy, TIA or Stroke
ASPIRE Study / 11

Registered before 01 Apr 2013
Where patient is registered at General Practice

IN

11D1-3 + 5. Type 2 Diabetic - Register
ASPIRE Study / 11

Has a Read code of Type II diabetes mellitus (X40J5) or one of its children
- Selecting only the most recent matching code
- Without a more recent Read code in...Read Codes and Children:
  Type I diabetes mellitus (X40J4)

Date of Read code before 01 Apr 2013
Registered before 01 Apr 2013
Where patient is registered at General Practice

AND IN

Either ACR, diabetic retinopathy, Stroke or TIA in the previous 12 months
ASPIRE Study / 11

Registered before 01 Apr 2013
Where patient is registered at General Practice

IN

ACR >30
ASPIRE Study / 11

Most recent Urine albumin/creatinine ratio reading > 30.0 mg/mmol
- Without a more recent Urine albumin/creatinine ratio reading <= 29.0 mg/mmol

Date of numeric reading between 01 Apr 2012 and 31 Mar 2013
Registered before 01 Apr 2013

OR IN

Diabetic Retinopathy
ASPIRE Study / 11

Has a Read code in...Read Codes and Children:
Diabetic retinopathy (F420.)
Excluding Exact Read Codes:
Advanced Artificial Eye Disease (Y0505)
Normal Macula Right eye (Y2187)
Normal Macula Left eye (Y2188)
No Photocoagulation Right eye (Y2189)
No Photocoagulation Left eye (Y2190)
Photocoagulation Right eye (Y2191)
Photocoagulation Left eye (Y2192)
Maculopathy - Refer Camera (Y2196)
Date of Read code between 01 Apr 2012 and 31 Mar 2013
Registered before 01 Apr 2013

OR IN

Retinal Screening Codes
ASPIRE Study / 11

Has a Read code in the RET (Retinal screening codes) QOF cluster
Show read codes in cluster RET.
Date of Read code between 01 Apr 2012 and 31 Mar 2013
Registered before 01 Apr 2013

OR IN

Stroke Register
ASPIRE Study / 11

Has a Read code in the STRT (Stroke or TIA codes) QOF cluster
Show read codes in cluster STRT.

OR IN

TIA Codes
ASPIRE Study / 11

Has a Read code in the TIA (TIA codes)

QOF cluster Show read codes in cluster TIA.

AND IN

BP <130/80
ASPIRE Study / 11

Where patient is registered at General Practice

IN

DM31 - Systolic =<130 in the previous 12 months AND Diastolic =<80
ASPIRE Study / 11

Where patient is registered at General Practice

IN

DM31 - Achievement: BP Diastolic exists in the last 15 months
ASPIRE Study / 11

Has numeric reading in the BPDIA (BP diastolic codes) nGMS cluster < 80.0
Date of numeric reading between 01 Jan 2012 and 31 Mar 2013
Registered before 01 Apr 2013
Where patient is registered at General Practice

AND IN

DM31 - Achievement: BP Systolic =<130 in the last 15 months
ASPIRE Study / 11

Has numeric reading in the BPSYS (BP systolic codes) nGMS cluster < 130.0
Date of numeric reading between 01 Jan 2012 and 31 Mar 2013
Registered before 01 Apr 2013
Where patient is registered at General Practice

OR IN

DM31 - Achievement: BP <131/81 in the last 15 months
ASPIRE Study / 11

BP Has a BP reading < 130 / 80
Date of BP reading between 01 Jan 2012 and 31 Mar 2013
Registered before 01 Apr 2013
Where patient is registered at General Practice
