# Supplementary material for: Developing ‘high impact’ guideline-based quality indicators for UK primary care: a multi-stage consensus process
Source: BMC Fam Pract. 2015 Oct 28;16:156. doi: 10.1186/s12875-015-0350-6 (PMC4624600; doi:10.1186/s12875-015-0350-6)
Supplement: Additional file 4 — Folder containing SystmOne™ search algorithms. (ZIP 12.7 mb) [file 12875_2015_350_MOESM4_ESM.zip › Aspire S1 diagrams tw edired/12D1 (Risky p).pdf]

Mandatory In
Optional In
Not In

12D1. Patients with a Peptic Ulcer recorded before 1.4.13 and who have had either a Low Dose Aspirin or NSAID Rx between 1.2.13 and 31.3.13
ASPIRE Study / 12

Registered before 01 Apr 2013
Where patient is registered at General Practice

IN

Peptic ulcer recorded ever
ASPIRE Study / 12

Has a Read code in...Read Codes and Children:
Peptic ulcer disease (XM0BZ)
Excluding Exact Read Codes:
Anastomotic ulcer (Xa1qA)

- Selecting only the most recent matching code

Date of Read code before 01 Apr 2013
Where patient is registered at General Practice

AND IN

Low Dose Aspirin or BNF 10.1.1 NSAID Rx prescribed
ASPIRE Study / 12

Registered before 01 Apr 2013
Where patient is registered at General Practice

IN

Low Dose Aspirin prescribed between 1.2.13 and 31.3.13
ASPIRE Study / 12

Has an issue of...Drugs:
Aspirin 75mg dispersible tablets
Aspirin 75mg dispersible tablets (A A H Pharmaceuticals Ltd)
Aspirin 75mg dispersible tablets (Actavis UK Ltd)
Aspirin 75mg dispersible tablets (Almus Pharmaceuticals Ltd)
Aspirin 75mg dispersible tablets (Aspar Pharmaceuticals Ltd)
Aspirin 75mg dispersible tablets (IVAX Pharmaceuticals UK Ltd)
Aspirin 75mg dispersible tablets (Kent Pharmaceuticals Ltd)
Aspirin 75mg dispersible tablets (Teva UK Ltd)
Aspirin 75mg dispersible tablets (Thornton & Ross Ltd)
Aspirin 75mg dispersible tablets (Wockhardt UK Ltd)
Aspirin 75mg gastro-resistant tablets
Aspirin 75mg gastro-resistant tablets (A A H Pharmaceuticals Ltd)
Aspirin 75mg gastro-resistant tablets (Actavis UK Ltd)
Aspirin 75mg gastro-resistant tablets (Almus Pharmaceuticals Ltd)
Aspirin 75mg gastro-resistant tablets (C P Pharmaceuticals Ltd)
Aspirin 75mg gastro-resistant tablets (Generics (UK) Ltd)
Aspirin 75mg gastro-resistant tablets (IVAX Pharmaceuticals UK Ltd)
Aspirin 75mg gastro-resistant tablets (Kent Pharmaceuticals Ltd)
Aspirin 75mg gastro-resistant tablets (Sandoz Ltd)
Aspirin 75mg gastro-resistant tablets (Sterwin Medicines)
Aspirin 75mg gastro-resistant tablets (Teva UK Ltd)
Aspirin 75mg gastro-resistant tablets (Wockhardt UK Ltd)
Aspirin 75mg tablets
Aspirin 75mg tablets (A A H Pharmaceuticals Ltd)
ASPIRIN dispersible tablet 75mg [AAH(VANT)]
ASPIRIN dispersible tablet 75mg [GALPHARM]
ASPIRIN dispersible tablet 75mg [LEXON(PH)]
ASPIRIN dispersible tablet 75mg [NUCARE]
ASPIRIN dispersible tablet 75mg [NUMARK]
ASPIRIN dispersible tablet 75mg [RANBAXY]
ASPIRIN dispersible tablet 75mg [SOVEREIGN]

ASPIRIN enteric coated tablets 75mg [GALEN]
Aspirin powder (J M Loveridge Ltd)
ASPIRIN powder [T & R]
ASPIRIN soluble tablet 75mg [CELLTECH]
ASPIRIN soluble tablet 75mg [CO-OPERATI]
ASPIRIN soluble tablet 75mg [CP PHARM]

- Include all drug types

Date of medication between 01 Feb 2013 and 31 Mar 2013

Where patient is registered at General Practice

OR IN

BNF 10.1.1 NSAIDs (excluding cox-2) between 1.2.13 and 31.3.13

ASPIRE Study / 12

Has medication in the 'NSAIDs' Action Group, excluding...Excluded Drugs:
Celecoxib 100mg capsules
Celecoxib 200mg capsules
Celecoxib 400mg capsules
Etoricoxib 120mg tablets
Etoricoxib 30mg tablets
Etoricoxib 60mg tablets
Etoricoxib 90mg tablets
parecoxib (roi) injection 20mg
parecoxib powder for solution for injection 40mg

- Include all drug types

Date of medication between 01 Feb 2013 and 31 Mar 2013

Where patient is registered at General Practice
