# Supplementary material for: Developing ‘high impact’ guideline-based quality indicators for UK primary care: a multi-stage consensus process
Source: BMC Fam Pract. 2015 Oct 28;16:156. doi: 10.1186/s12875-015-0350-6 (PMC4624600; doi:10.1186/s12875-015-0350-6)
Supplement: Additional file 4 — Folder containing SystmOne™ search algorithms. (ZIP 12.7 mb) [file 12875_2015_350_MOESM4_ESM.zip › Aspire S1 diagrams tw edired/12D12 (Risky p).pdf]

———	Mandatory In
- - - -	Optional In
....	Not In

12D12. Any Patient in any of the Denominators 1-8
ASPIRE Study / 12

Registered before 01 Apr 2013
Where patient is registered at General Practice

IN

12D14. HF - Register (DRHF01)
ASPIRE Study / 12

Has a Read code in the DRHF1 (Codes for Heart failure) QOF cluster
Show read codes in cluster DRHF1.
Date of Read code before 01 Apr 2013
Registered before 01 Apr 2013
Where patient is registered at General Practice

OR IN

12D7. Warfarin Rx OR Warfarin Rx read code between 1.1.13 and 31.3.13 and either Low dose Aspirin or Clopidogrel Rx between 1.2.13 and 31.3.13
ASPIRE Study / 12

Registered before 01 Apr 2013
Where patient is registered at General Practice

IN

Warfarin Rx OR Warfarin Rx read code
ASPIRE Study / 12

Registered before 01 Apr 2013
Where patient is registered at General Practice

IN

Warfarin between 1.1.13 and 31.3.13
ASPIRE Study / 12

Has a Read code in the WAR (Warfarin prescription codes) QOF cluster
Show read codes in cluster WAR.
- Selecting only the most recent matching code

Date of Read code between 01 Jan 2013 and 31 Mar 2013
Where patient is registered at General Practice

OR IN

BNF 2.8.2 (oral anti-coagulants) between 1.1.13 and 31.3.13
ASPIRE Study / 12

Has medication in the 'Oral anticoagulants' Action Group
- Include all drug types

Date of medication between 01 Jan 2013 and 31 Mar 2013
Where patient is registered at General Practice

AND IN

Low Dose Aspirin or Clopidogrel Rx prescribed
ASPIRE Study / 12

Registered before 01 Apr 2013
Where patient is registered at General Practice

IN

Low Dose Aspirin prescribed between 1.2.13 and 31.3.13
ASPIRE Study / 12

Has an issue of...Drugs:
Aspirin 75mg dispersible tablets
Aspirin 75mg dispersible tablets (A A H Pharmaceuticals Ltd)
Aspirin 75mg dispersible tablets (Actavis UK Ltd)
Aspirin 75mg dispersible tablets (Almus Pharmaceuticals Ltd)
Aspirin 75mg dispersible tablets (Aspar Pharmaceuticals Ltd)
Aspirin 75mg dispersible tablets (IVAX Pharmaceuticals UK Ltd)
Aspirin 75mg dispersible tablets (Kent Pharmaceuticals Ltd)
Aspirin 75mg dispersible tablets (Teva UK Ltd)
Aspirin 75mg dispersible tablets (Thornton & Ross Ltd)
Aspirin 75mg dispersible tablets (Wockhardt UK Ltd)

UK Ltd)
Aspirin 75mg gastro-resistant tablets
Aspirin 75mg gastro-resistant tablets (A A H Pharmaceuticals Ltd)
Aspirin 75mg gastro-resistant tablets (Actavis UK Ltd)
Aspirin 75mg gastro-resistant tablets (Almus Pharmaceuticals Ltd)
Aspirin 75mg gastro-resistant tablets (C P Pharmaceuticals Ltd)
Aspirin 75mg gastro-resistant tablets (Generics (UK) Ltd)
Aspirin 75mg gastro-resistant tablets (IVAX Pharmaceuticals UK Ltd)
Aspirin 75mg gastro-resistant tablets (Kent Pharmaceuticals Ltd)
Aspirin 75mg gastro-resistant tablets (Sandoz Ltd)
Aspirin 75mg gastro-resistant tablets (Sterwin Medicines)
Aspirin 75mg gastro-resistant tablets (Teva UK Ltd)
Aspirin 75mg gastro-resistant tablets (Wockhardt UK Ltd)
Aspirin 75mg tablets
Aspirin 75mg tablets (A A H Pharmaceuticals Ltd)
ASPIRIN dispersible tablet 75mg [AAH(VANT)]
ASPIRIN dispersible tablet 75mg [GALPHARM]
ASPIRIN dispersible tablet 75mg [LEXON(PH)]
ASPIRIN dispersible tablet 75mg [NUCARE]
ASPIRIN dispersible tablet 75mg [NUMARK]
ASPIRIN dispersible tablet 75mg [RANBAXY]
ASPIRIN dispersible tablet 75mg [SOVEREIGN]
ASPIRIN enteric coated tablets 75mg [GALEN]
Aspirin powder (J M Loveridge Ltd)
ASPIRIN powder [T & R]
ASPIRIN soluble tablet 75mg [CELLTECH]
ASPIRIN soluble tablet 75mg [CO-OPERATI]
ASPIRIN soluble tablet 75mg [CP PHARM]

- Include all drug types

Date of medication between 01 Feb 2013 and 31 Mar 2013

Where patient is registered at General Practice

OR IN

Clopidogrel between 1.2.13 and 31.3.13
ASPIRE Study / 12

Has an issue of...Drugs:
clopidogrel (form not specified)
CLOPIDOGREL (Generic Manuf) (form not specified)
clopidogrel oral liquid 1mg/ml
Clopidogrel 25mg/5ml oral suspension
Clopidogrel 300mg tablets
Clopidogrel 75mg tablets
Clopidogrel 75mg tablets (A A H Pharmaceuticals Ltd)
Clopidogrel 75mg tablets (Actavis UK Ltd)
Clopidogrel 75mg tablets (Almus Pharmaceuticals Ltd)
Clopidogrel 75mg tablets (Aspire Pharma Ltd)
Clopidogrel 75mg tablets (Dexcel-Pharma Ltd)
Clopidogrel 75mg tablets (Dr Reddy's Laboratories (UK) Ltd)
Clopidogrel 75mg tablets (Generics (UK) Ltd)
Clopidogrel 75mg tablets (Teva UK Ltd)
Clopidogrel 75mg tablets (Wockhardt UK Ltd)
Clopidogrel 75mg/5ml oral solution
Clopidogrel 75mg/5ml oral suspension
clopidogrel oral powder
clopidogrel with aspirin (roi) tablets 75mg + 75mg

- Include all drug types

Date of medication between 01 Feb 2013 and 31 Mar 2013

Where patient is registered at General Practice

OR IN

12D1. Patients with a Peptic Ulcer recorded before 1.4.13 and who have had either a Low Dose Aspirin or NSAID Rx between 1.2.13 and 31.3.13

between 1.2.13 and 31.3.13
ASPIRE Study / 12

Registered before 01 Apr 2013
Where patient is registered at General Practice

IN

Peptic ulcer recorded ever
ASPIRE Study / 12

Has a Read code in...Read Codes and Children:
Peptic ulcer disease (XM0BZ)
Excluding Exact Read Codes:
Anastomotic ulcer (Xa1qA)

- Selecting only the most recent matching code

Date of Read code before 01 Apr 2013
Where patient is registered at General Practice

AND IN

Low Dose Aspirin or BNF 10.1.1 NSAID Rx prescribed
ASPIRE Study / 12

Registered before 01 Apr 2013
Where patient is registered at General Practice

IN

Low Dose Aspirin prescribed between 1.2.13 and 31.3.13
ASPIRE Study / 12

Has an issue of...Drugs:
Aspirin 75mg dispersible tablets
Aspirin 75mg dispersible tablets (A A H Pharmaceuticals Ltd)
Aspirin 75mg dispersible tablets (Actavis UK Ltd)
Aspirin 75mg dispersible tablets (Almus Pharmaceuticals Ltd)
Aspirin 75mg dispersible tablets (Aspar Pharmaceuticals Ltd)
Aspirin 75mg dispersible tablets (IVAX Pharmaceuticals UK Ltd)
Aspirin 75mg dispersible tablets (Kent Pharmaceuticals Ltd)
Aspirin 75mg dispersible tablets (Teva UK Ltd)
Aspirin 75mg dispersible tablets (Thornton & Ross Ltd)
Aspirin 75mg dispersible tablets (Wockhardt UK Ltd)
Aspirin 75mg gastro-resistant tablets
Aspirin 75mg gastro-resistant tablets (A A H Pharmaceuticals Ltd)
Aspirin 75mg gastro-resistant tablets (Actavis UK Ltd)
Aspirin 75mg gastro-resistant tablets (Almus Pharmaceuticals Ltd)
Aspirin 75mg gastro-resistant tablets (C P Pharmaceuticals Ltd)
Aspirin 75mg gastro-resistant tablets (Generics (UK) Ltd)
Aspirin 75mg gastro-resistant tablets (IVAX Pharmaceuticals UK Ltd)
Aspirin 75mg gastro-resistant tablets (Kent Pharmaceuticals Ltd)
Aspirin 75mg gastro-resistant tablets (Sandoz Ltd)
Aspirin 75mg gastro-resistant tablets (Sterwin Medicines)
Aspirin 75mg gastro-resistant tablets (Teva UK Ltd)
Aspirin 75mg gastro-resistant tablets (Wockhardt UK Ltd)
Aspirin 75mg tablets
Aspirin 75mg tablets (A A H Pharmaceuticals Ltd)
ASPIRIN dispersible tablet 75mg [AAH(VANT)]
ASPIRIN dispersible tablet 75mg [GALPHARM]
ASPIRIN dispersible tablet 75mg [LEXON(PH)]
ASPIRIN dispersible tablet 75mg [NUCARE]
ASPIRIN dispersible tablet 75mg [NUMARK]
ASPIRIN dispersible tablet 75mg [RANBAXY]
ASPIRIN dispersible tablet 75mg [SOVEREIGN]
ASPIRIN enteric coated tablets 75mg [GALEN]
Aspirin powder (J M Loveridge Ltd)
ASPIRIN powder [T & R]
ASPIRIN soluble tablet 75mg [CELLTECH]
[illegible]

ASPIRIN soluble tablet 75mg [CO-OPERATI]
ASPIRIN soluble tablet 75mg [CP PHARM]

- Include all drug types
- Date of medication between 01 Feb 2013 and 31 Mar 2013
- Where patient is registered at General Practice

OR IN

BNF 10.1.1 NSAIDs (excluding cox-2) between 1.2.13 and 31.3.13
ASPIRE Study / 12

- Has medication in the 'NSAIDs' Action Group, excluding...Excluded Drugs:
  Celecoxib 100mg capsules
  Celecoxib 200mg capsules
  Celecoxib 400mg capsules
  Etoricoxib 120mg tablets
  Etoricoxib 30mg tablets
  Etoricoxib 60mg tablets
  Etoricoxib 90mg tablets
  parecoxib (roi) injection 20mg
  parecoxib powder for solution for injection 40mg
- Include all drug types
- Date of medication between 01 Feb 2013 and 31 Mar 2013
- Where patient is registered at General Practice

OR IN

12D2. Patients who are aged 75 or over at 1.4.12 and who have had a NSAID prescribed between 1.2.13 and 31.3.13
ASPIRE Study / 12

- Registered before 01 Apr 2013
- Where patient is registered at General Practice

IN

Patients aged over 75 as of 1.4.12
ASPIRE Study / 12

- Born before 01 Apr 1937
- Registered before 01 Apr 2013
- Where patient is registered at General Practice

AND IN

BNF 10.1.1 NSAIDs (excluding cox-2) between 1.2.13 and 31.3.13
ASPIRE Study / 12

- Has medication in the 'NSAIDs' Action Group, excluding...Excluded Drugs:
  Celecoxib 100mg capsules
  Celecoxib 200mg capsules
  Celecoxib 400mg capsules
  Etoricoxib 120mg tablets
  Etoricoxib 30mg tablets
  Etoricoxib 60mg tablets
  Etoricoxib 90mg tablets
  parecoxib (roi) injection 20mg
  parecoxib powder for solution for injection 40mg
- Include all drug types
- Date of medication between 01 Feb 2013 and 31 Mar 2013
- Where patient is registered at General Practice

OR IN

12D3. Patients aged over 65 (as of 1.4.12) and prescribed Aspirin between 1.1.13 and 31.3.13 and who have had a NSAID between 1.2.13 and 31.3.13
ASPIRE Study / 12

- Registered before 01 Apr 2013
- Where patient is registered at General Practice

IN

Patients aged over 65 (as of 1.4.12) and prescribed Aspirin between 1.1.13 and 31.3.13
ASPIRE Study / 12

- Registered before 01 Apr 2013
- Where patient is registered at General Practice

IN

Patients aged over 65 as of 1.4.12
ASPIRE Study / 12

- Born before 01 Apr 1947

Where patient is registered at General Practice

AND IN

Aspirin prescribed between 1.1.13 and 31.3.13
ASPIRE Study / 12

Has an issue of...Drugs:
aspirin (form not specified)
ASPIRIN (Generic Manuf) (form not specified)
Aspirin 150mg suppositories
Aspirin 300mg / Paracetamol 200mg dispersible tablets sugar free
Aspirin 300mg dispersible tablets
Aspirin 300mg effervescent tablets sugar free
Aspirin 300mg gastro-resistant tablets
Aspirin 300mg modified-release tablets
Aspirin 300mg orodispersible tablets sugar free
Aspirin 300mg suppositories
Aspirin 300mg tablets
Aspirin 325mg / Caffeine 15mg tablets
Aspirin 325mg / Caffeine 22mg tablets
Aspirin 500mg effervescent tablets sugar free
Aspirin 500mg granules sachets sugar free
Aspirin 600mg / Caffeine 50mg oral powder sachets sugar free
Aspirin 75mg dispersible tablets
Aspirin 75mg gastro-resistant tablets
Aspirin 75mg tablets
aspirin capsules 162.5mg
aspirin chewing gum 227mg
aspirin effervescent tablets 100mg
aspirin effervescent tablets 300mg
aspirin gastro-resistant tablets 600mg
aspirin high dose oral liquid
aspirin low dose oral liquid
aspirin mixture
aspirin modified release capsules 162.5mg
aspirin modified release tablet 100mg
aspirin modified release tablet 324mg
aspirin modified release tablet 500mg
Aspirin powder
aspirin tablets 320mg

- Include all drug types

Date of medication between 01 Jan 2013 and 31 Mar 2013
Where patient is registered at General Practice

AND IN

BNF 10.1.1 NSAIDs (excluding cox-2) between 1.2.13 and 31.3.13
ASPIRE Study / 12

Has medication in the 'NSAIDs' Action Group, excluding...Excluded Drugs:
Celecoxib 100mg capsules
Celecoxib 200mg capsules
Celecoxib 400mg capsules
Etoricoxib 120mg tablets
Etoricoxib 30mg tablets
Etoricoxib 60mg tablets
Etoricoxib 90mg tablets
parecoxib (roi) injection 20mg
parecoxib powder for solution for injection 40mg

- Include all drug types

Date of medication between 01 Feb 2013 and 31 Mar 2013
Where patient is registered at General Practice

OR IN

12D5. Warfarin Rx OR Warfarin Rx read code between 1.1.13 and 31.3.13 and also a NSAID between 1.2.13 and 31.3.13
ASPIRE Study / 12

Registered before 01 Apr 2013
Where patient is registered at General Practice

IN

Warfarin Rx OR Warfarin Rx read code
ASPIRE Study / 12

Registered before 01 Apr 2013
Where patient is registered at General Practice

IN

Warfarin between 1.1.13 and 31.3.13
ASPIRE Study / 12

Has a Read code in the WAR (Warfarin

Has a Read code in the WAR (Warfarin prescription codes) QOF cluster
Show read codes in cluster WAR.

- Selecting only the most recent matching code

Date of Read code between 01 Jan 2013 and 31 Mar 2013
Where patient is registered at General Practice

OR IN

BNF 2.8.2 (oral anti-coagulants) between 1.1.13 and 31.3.13
ASPIRE Study / 12

Has medication in the 'Oral anticoagulants' Action Group

- Include all drug types

Date of medication between 01 Jan 2013 and 31 Mar 2013
Where patient is registered at General Practice

AND IN

BNF 10.1.1 NSAIDs (excluding cox-2) between 1.2.13 and 31.3.13
ASPIRE Study / 12

Has medication in the 'NSAIDs' Action Group, excluding...Excluded Drugs:
Celecoxib 100mg capsules
Celecoxib 200mg capsules
Celecoxib 400mg capsules
Etoricoxib 120mg tablets
Etoricoxib 30mg tablets
Etoricoxib 60mg tablets
Etoricoxib 90mg tablets
parecoxib (roi) injection 20mg
parecoxib powder for solution for injection 40mg

- Include all drug types

Date of medication between 01 Feb 2013 and 31 Mar 2013
Where patient is registered at General Practice

OR IN

12D4. Patients aged over 65 (as of 1.4.12) and prescribed either Low dose Aspirin and Clopidogrel between 1.1.13 and 31.3.13
ASPIRE Study / 12

Registered before 01 Apr 2013
Where patient is registered at General Practice

IN

Patients aged over 65 (as of 1.4.12) and prescribed Aspirin between 1.1.13 and 31.3.13
ASPIRE Study / 12

Registered before 01 Apr 2013
Where patient is registered at General Practice

IN

Patients aged over 65 as of 1.4.12
ASPIRE Study / 12

Born before 01 Apr 1947
Where patient is registered at General Practice

AND IN

Aspirin prescribed between 1.1.13 and 31.3.13
ASPIRE Study / 12

Has an issue of...Drugs:
aspirin (form not specified)
ASPIRIN (Generic Manuf) (form not specified)
Aspirin 150mg suppositories
Aspirin 300mg / Paracetamol 200mg dispersible tablets sugar free
Aspirin 300mg dispersible tablets
Aspirin 300mg effervescent tablets sugar free
Aspirin 300mg gastro-resistant tablets
Aspirin 300mg modified-release tablets
Aspirin 300mg orodispersible tablets sugar free
Aspirin 300mg suppositories
Aspirin 300mg tablets
Aspirin 325mg / Caffeine 15mg tablets
Aspirin 325mg / Caffeine 22mg tablets
Aspirin 500mg effervescent tablets sugar free

Aspirin 500mg granules sachets sugar free
Aspirin 600mg / Caffeine 50mg oral powder sachets sugar free
Aspirin 75mg dispersible tablets
Aspirin 75mg gastro-resistant tablets
Aspirin 75mg tablets
aspirin capsules 162.5mg
aspirin chewing gum 227mg
aspirin effervescent tablets 100mg
aspirin effervescent tablets 300mg
aspirin gastro-resistant tablets 600mg
aspirin high dose oral liquid
aspirin low dose oral liquid
aspirin mixture
aspirin modified release capsules 162.5mg
aspirin modified release tablet 100mg
aspirin modified release tablet 324mg
aspirin modified release tablet 500mg
Aspirin powder
aspirin tablets 320mg

- Include all drug types
- Date of medication between 01 Jan 2013 and 31 Mar 2013
- Where patient is registered at General Practice

AND IN

Clopidogrel between 1.2.13 and 31.3.13
ASPIRE Study / 12

Has an issue of...Drugs:
clopidogrel (form not specified)
CLOPIDOGREL (Generic Manuf) (form not specified)
clopidogrel oral liquid 1mg/ml
Clopidogrel 25mg/5ml oral suspension
Clopidogrel 300mg tablets
Clopidogrel 75mg tablets
Clopidogrel 75mg tablets (A A H Pharmaceuticals Ltd)
Clopidogrel 75mg tablets (Actavis UK Ltd)
Clopidogrel 75mg tablets (Almus Pharmaceuticals Ltd)
Clopidogrel 75mg tablets (Aspire Pharma Ltd)
Clopidogrel 75mg tablets (Dexcel-Pharma Ltd)
Clopidogrel 75mg tablets (Dr Reddy's Laboratories (UK) Ltd)
Clopidogrel 75mg tablets (Generics (UK) Ltd)
Clopidogrel 75mg tablets (Teva UK Ltd)
Clopidogrel 75mg tablets (Wockhardt UK Ltd)
Clopidogrel 75mg/5ml oral solution
Clopidogrel 75mg/5ml oral suspension
clopidogrel oral powder
clopidogrel with aspirin (roi) tablets 75mg + 75mg

- Include all drug types
- Date of medication between 01 Feb 2013 and 31 Mar 2013
- Where patient is registered at General Practice
