# Supplementary material for: Developing ‘high impact’ guideline-based quality indicators for UK primary care: a multi-stage consensus process
Source: BMC Fam Pract. 2015 Oct 28;16:156. doi: 10.1186/s12875-015-0350-6 (PMC4624600; doi:10.1186/s12875-015-0350-6)
Supplement: Additional file 4 — Folder containing SystmOne™ search algorithms. (ZIP 12.7 mb) [file 12875_2015_350_MOESM4_ESM.zip › Aspire S1 diagrams tw edired/12D14 (Risky p).pdf]

———	Mandatory In
- - - -	Optional In
....	Not In

12D14. HF - Register (DRHF01)
ASPIRE Study / 12

Has a Read code in the DRHF1 (Codes for Heart failure) QOF cluster
Show read codes in cluster DRHF1.

Date of Read code before 01 Apr 2013

Registered before 01 Apr 2013

Where patient is registered at General Practice
