# Supplementary material for: Developing ‘high impact’ guideline-based quality indicators for UK primary care: a multi-stage consensus process
Source: BMC Fam Pract. 2015 Oct 28;16:156. doi: 10.1186/s12875-015-0350-6 (PMC4624600; doi:10.1186/s12875-015-0350-6)
Supplement: Additional file 4 — Folder containing SystmOne™ search algorithms. (ZIP 12.7 mb) [file 12875_2015_350_MOESM4_ESM.zip › Aspire S1 diagrams tw edired/12D2 (Risky p).pdf]

———	Mandatory In
- - - -	Optional In
....	Not In

12D2. Patients who are aged 75 or over at 1.4.12 and who have had a NSAID prescribed between 1.2.13 and 31.3.13
ASPIRE Study / 12

Registered before 01 Apr 2013
Where patient is registered at General Practice

IN

Patients aged over 75 as of 1.4.12
ASPIRE Study / 12

Born before 01 Apr 1937
Registered before 01 Apr 2013
Where patient is registered at General Practice

AND IN

BNF 10.1.1 NSAIDs (excluding cox-2) between 1.2.13 and 31.3.13
ASPIRE Study / 12

Has medication in the 'NSAIDs' Action Group, excluding...Excluded Drugs:
Celecoxib 100mg capsules
Celecoxib 200mg capsules
Celecoxib 400mg capsules
Etoricoxib 120mg tablets
Etoricoxib 30mg tablets
Etoricoxib 60mg tablets
Etoricoxib 90mg tablets
parecoxib (roi) injection 20mg
parecoxib powder for solution for injection 40mg

- Include all drug types

Date of medication between 01 Feb 2013 and 31 Mar 2013
Where patient is registered at General Practice
