# Supplementary material for: Developing ‘high impact’ guideline-based quality indicators for UK primary care: a multi-stage consensus process
Source: BMC Fam Pract. 2015 Oct 28;16:156. doi: 10.1186/s12875-015-0350-6 (PMC4624600; doi:10.1186/s12875-015-0350-6)
Supplement: Additional file 4 — Folder containing SystmOne™ search algorithms. (ZIP 12.7 mb) [file 12875_2015_350_MOESM4_ESM.zip › Aspire S1 diagrams tw edired/12D3 (Risky p).pdf]

Mandatory In
Optional In
Not In

12D3. Patients aged over 65 (as of 1.4.12) and prescribed Aspirin between 1.1.13 and 31.3.13 and who have had a NSAID between 1.2.13 and 31.3.13
ASPIRE Study / 12

Registered before 01 Apr 2013
Where patient is registered at General Practice

IN → **Patients aged over 65 (as of 1.4.12) and prescribed Aspirin between 1.1.13 and 31.3.13**
ASPIRE Study / 12

Registered before 01 Apr 2013
Where patient is registered at General Practice

IN → **Patients aged over 65 as of 1.4.12**
ASPIRE Study / 12

Born before 01 Apr 1947
Where patient is registered at General Practice

AND IN → **Aspirin prescribed between 1.1.13 and 31.3.13**
ASPIRE Study / 12

Has an issue of...Drugs:
aspirin (form not specified)
ASPIRIN (Generic Manuf) (form not specified)
Aspirin 150mg suppositories
Aspirin 300mg / Paracetamol 200mg dispersible tablets sugar free
Aspirin 300mg dispersible tablets
Aspirin 300mg effervescent tablets sugar free
Aspirin 300mg gastro-resistant tablets
Aspirin 300mg modified-release tablets
Aspirin 300mg orodispersible tablets sugar free
Aspirin 300mg suppositories
Aspirin 300mg tablets
Aspirin 325mg / Caffeine 15mg tablets
Aspirin 325mg / Caffeine 22mg tablets
Aspirin 500mg effervescent tablets sugar free
Aspirin 500mg granules sachets sugar free
Aspirin 600mg / Caffeine 50mg oral powder sachets sugar free
Aspirin 75mg dispersible tablets
Aspirin 75mg gastro-resistant tablets
Aspirin 75mg tablets
aspirin capsules 162.5mg
aspirin chewing gum 227mg
aspirin effervescent tablets 100mg
aspirin effervescent tablets 300mg
aspirin gastro-resistant tablets 600mg
aspirin high dose oral liquid
aspirin low dose oral liquid
aspirin mixture
aspirin modified release capsules 162.5mg
aspirin modified release tablet 100mg
aspirin modified release tablet 324mg
aspirin modified release tablet 500mg
Aspirin powder
aspirin tablets 320mg

- Include all drug types

Date of medication between 01 Jan 2013 and 31 Mar 2013
Where patient is registered at General Practice

AND IN → **BNF 10.1.1 NSAIDs (excluding cox-2) between 1.2.13 and 31.3.13**
ASPIRE Study / 12

Has medication in the 'NSAIDs' Action Group, excluding...Excluded Drugs:
Celecoxib 100mg capsules
Celecoxib 200mg capsules
Celecoxib 400mg capsules
Etoricoxib 120mg tablets
Etoricoxib 30mg tablets
Etoricoxib 60mg tablets
Etoricoxib 90mg tablets
parecoxib (roi) injection 20mg
parecoxib powder for solution for injection 40mg

- Include all drug types

Date of medication between 01 Feb 201[illegible]

Date of medication between 01 Feb 2013 and 31 Mar 2013

Where patient is registered at General Practice
