# Supplementary material for: Developing ‘high impact’ guideline-based quality indicators for UK primary care: a multi-stage consensus process
Source: BMC Fam Pract. 2015 Oct 28;16:156. doi: 10.1186/s12875-015-0350-6 (PMC4624600; doi:10.1186/s12875-015-0350-6)
Supplement: Additional file 4 — Folder containing SystmOne™ search algorithms. (ZIP 12.7 mb) [file 12875_2015_350_MOESM4_ESM.zip › Aspire S1 diagrams tw edired/12N11 (Risky p).pdf]

——	Mandatory In
- - - -	Optional In
....	Not In

12N11. CKD Register and NSAID between 1.2.13 and 31.3.13
ASPIRE Study / 12

- Registered before 01 Apr 2013
- Where patient is registered at General Practice

IN →

BNF 10.1.1 NSAIDs between 1.2.13 and 31.3.13
ASPIRE Study / 12

- Has medication in the 'NSAIDs' Action Group
  - Include all drug types
- Date of medication between 01 Feb 2013 and 31 Mar 2013
- Where patient is registered at General Practice

AND IN →

12D11. CKD Register
ASPIRE Study / 12

- Has a Read code in the DRCKD1 (Chronic kidney disease codes 3-5) QOF cluster Show read codes in cluster DRCKD1.
  - Selecting only the most recent matching code
  - Without a more recent Read code in the DRCKD2 (Chronic kidney disease codes 1-2) QOF cluster
- Date of Read code before 01 Apr 2013
- Where patient is registered at General Practice
