# Supplementary material for: Developing ‘high impact’ guideline-based quality indicators for UK primary care: a multi-stage consensus process
Source: BMC Fam Pract. 2015 Oct 28;16:156. doi: 10.1186/s12875-015-0350-6 (PMC4624600; doi:10.1186/s12875-015-0350-6)
Supplement: Additional file 4 — Folder containing SystmOne™ search algorithms. (ZIP 12.7 mb) [file 12875_2015_350_MOESM4_ESM.zip › Aspire S1 diagrams tw edired/12N13 (Risky p).pdf]

Legend: —— Mandatory In; - - - - Optional In; .... Not In

- **12N13. Any patient in any of the Numerators 9+11**
  ASPIRE Study / 12
  - Registered before 01 Apr 2013
  - Where patient is registered at General Practice
  - IN → **12N9. Diuretic and ACE/ARB between 1.1.13 and 31.3.13 AND NSAID between 1.2.13 and 31.3.13**
    ASPIRE Study / 12
    - Registered before 01 Apr 2013
    - Where patient is registered at General Practice
    - IN → **BNF 10.1.1 NSAIDs between 1.2.13 and 31.3.13**
      ASPIRE Study / 12
      - Has medication in the 'NSAIDs' Action Group
      - Include all drug types
      - Date of medication between 01 Feb 2013 and 31 Mar 2013
      - Where patient is registered at General Practice
    - AND IN → **12D9. Diureticand ACE/ARB between 1.1.13 and 31.3.13**
      ASPIRE Study / 12
      - IN → **ACE-1 or ARB**
        ASPIRE Study / 12
        - Where patient is registered at General Practice
        - IN → **BNF 2.5.5.1**
          ASPIRE Study / 12
          - Has medication in the 'ACE inhibitors' Action Group
          - Include all drug types
          - Date of medication between 01 Jan 2013 and 31 Mar 2013
          - Where patient is registered at General Practice
        - OR IN → **BNF 2.5.5.2**
          ASPIRE Study / 12
          - Has medication in the 'Angiotensin-II antagonists' Action Group
          - Include all drug types
          - Date of medication between 01 Jan 2013 and 31 Mar 2013
          - Where patient is registered at General Practice
      - AND IN → **BNF Diuretics**
        ASPIRE Study / 12
        - Has medication in the 'Diuretics' Action Group
        - Include all drug types
        - Date of medication between 01 Jan 2013 and 31 Mar 2013
        - Where patient is registered at General Practice
  - OR IN → **12N11. CKD Register and NSAID between 1.2.13 and 31.3.13**
    ASPIRE Study / 12
    - Registered before 01 Apr 2013
    - Where patient is registered at General Practice
    - IN → **BNF 10.1.1 NSAIDs between 1.2.13 and 31.3.13**
      ASPIRE Study / 12
      - Has medication in the 'NSAIDs' Action Group
      - Include all drug types
      - Date of medication between 01 Feb 2013 and 31 Mar 2013
      - Where patient is registered at General Practice

Practice

AND IN

12D11. CKD Register
ASPIRE Study / 12

Has a Read code in the DRCKD1 (Chronic kidney disease codes 3-5) QOF cluster
Show read codes in cluster DRCKD1.

- Selecting only the most recent matching code
- Without a more recent Read code in the DRCKD2 (Chronic kidney disease codes 1-2) QOF cluster

Date of Read code before 01 Apr 2013
Where patient is registered at General Practice
