# Supplementary material for: Developing ‘high impact’ guideline-based quality indicators for UK primary care: a multi-stage consensus process
Source: BMC Fam Pract. 2015 Oct 28;16:156. doi: 10.1186/s12875-015-0350-6 (PMC4624600; doi:10.1186/s12875-015-0350-6)
Supplement: Additional file 4 — Folder containing SystmOne™ search algorithms. (ZIP 12.7 mb) [file 12875_2015_350_MOESM4_ESM.zip › Aspire S1 diagrams tw edired/13N2 (AF #39).pdf]

Mandatory In
Optional In
Not In

13N2. AF and Stroke with Warfarin Rx (read code or Rx OR Contraindication)
ASPIRE Study / 13

Registered before 01 Apr 2013
Where patient is registered at General Practice

IN

- **13D1+2. AF Register and Stroke Register**
  ASPIRE Study / 13

  Registered before 01 Apr 2013
  Where patient is registered at General Practice

  IN

  - **STIA001 - Register**
    ASPIRE Study / 13

    Has a Read code in the STRT (Stroke or TIA codes) QOF cluster
    Show read codes in cluster STRT.
    - Selecting only the earliest matching code

    Date of Read code before 01 Apr 2013

  AND IN

  - **AF001 - Register**
    ASPIRE Study / 13

    Has a Read code in the DRAFIB1 (Atrial fibrillation codes) QOF cluster
    Show read codes in cluster DRAFIB1.
    - Selecting only the most recent matching code
    - Without a more recent Read code in the DRAFIB2 (Atrial fibrillation resolved codes) QOF cluster

    Date of Read code before 01 Apr 2013

AND IN

- **Warfarin Rx or Warfarin contraindication codes**
  ASPIRE Study / 13

  Where patient is registered at General Practice

  IN

  - **Warfarin Rx OR Warfarin Rx read code**
    ASPIRE Study / 13

    Where patient is registered at General Practice

    IN

    - **WAR DAT - Warfarin within last 12 months**
      ASPIRE Study / 13

      Has a Read code in the WAR (Warfarin prescription codes) QOF cluster
      Show read codes in cluster WAR.
      - Selecting only the most recent matching code

      Date of Read code between 01 Apr 2012 and 31 Mar 2013
      Where patient is registered at General Practice

    OR IN

    - **BNF 2.8.2 (oral anti-coagulants) in the last 12 months**
      ASPIRE Study / 13

      Has medication in the 'Oral anticoagulants' Action Group
      - Include all drug types

      Date of medication between 01 Apr 2012 and 31 Mar 2013
      Where patient is registered at General Practice

  OR IN

  - **XWAR & TXWAR - Any Warfarin contraindication**
    ASPIRE Study / 13

    Where patient is registered at General Practice

    IN

    - **Expiring Warfarin contraindication within last 12 months**
      ASPIRE Study / 13

      Has a Read code in the TXWAR (Warfarin contraindications: expiring) QOF cluster
      Show read codes in cluster TXWAR.
      - Selecting only the most recent matching code

Date of Read code between 01 Apr 2012 and 31 Mar 2013

Where patient is registered at General Practice

OR IN

Persisting Warfarin contraindication in the last 12 months

ASPIRE Study / 13

Has a Read code in the XWAR (Warfarin contraindications: persistent) QOF cluster
Show read codes in cluster XWAR.

- Selecting only the most recent matching code

Date of Read code between 01 Apr 2012 and 31 Mar 2013

Where patient is registered at General Practice
