# Supplementary material for: Developing ‘high impact’ guideline-based quality indicators for UK primary care: a multi-stage consensus process
Source: BMC Fam Pract. 2015 Oct 28;16:156. doi: 10.1186/s12875-015-0350-6 (PMC4624600; doi:10.1186/s12875-015-0350-6)
Supplement: Additional file 4 — Folder containing SystmOne™ search algorithms. (ZIP 12.7 mb) [file 12875_2015_350_MOESM4_ESM.zip › Aspire S1 diagrams tw edired/13N4 (AF #39).pdf]

——	Mandatory In
- - - -	Optional In
....	Not In

13N4. AF and CHADs 2 = 1 with Warfarin Rx / Antiplatelet (read code or Rx OR Contraindication)
ASPIRE Study / 13

Registered before 01 Apr 2013
Where patient is registered at General Practice

IN → **Warfarin Rx or Read Code OR Antiplatelet or Contraindications**
ASPIRE Study / 13

Where patient is registered at General Practice

IN → **Warfarin Rx or Warfarin contraindication codes**
ASPIRE Study / 13

Where patient is registered at General Practice

IN → **Warfarin Rx OR Warfarin Rx read code**
ASPIRE Study / 13

Where patient is registered at General Practice

IN → **WAR DAT - Warfarin within last 12 months**
ASPIRE Study / 13

Has a Read code in the WAR (Warfarin prescription codes) QOF cluster
Show read codes in cluster WAR.
- Selecting only the most recent matching code

Date of Read code between 01 Apr 2012 and 31 Mar 2013
Where patient is registered at General Practice

OR IN → **BNF 2.8.2 (oral anti-coagulants) in the last 12 months**
ASPIRE Study / 13

Has medication in the 'Oral anticoagulants' Action Group
- Include all drug types

Date of medication between 01 Apr 2012 and 31 Mar 2013
Where patient is registered at General Practice

OR IN → **XWAR & TXWAR - Any Warfarin contraindication**
ASPIRE Study / 13

Where patient is registered at General Practice

IN → **Expiring Warfarin contraindication within last 12 months**
ASPIRE Study / 13

Has a Read code in the TXWAR (Warfarin contraindications: expiring) QOF cluster
Show read codes in cluster TXWAR.
- Selecting only the most recent matching code

Date of Read code between 01 Apr 2012 and 31 Mar 2013
Where patient is registered at General Practice

OR IN → **Persisting Warfarin contraindication in the last 12 months**
ASPIRE Study / 13

Has a Read code in the XWAR (Warfarin contraindications: persistent) QOF cluster
Show read codes in cluster XWAR.
- Selecting only the most recent matching code

Date of Read code between 01 Apr 2012 and 31 Mar 2013
Where patient is registered at General Practice

OR IN → **BNF 2.9 Rx or Contraindications**
ASPIRE Study / 13

[illegible]

Where patient is registered at General Practice

IN

BNF 2.9 (Antiplatelets) in the last 12 months
ASPIRE Study / 13

- Has medication in the 'Antiplatelet drugs' Action Group
- Include all drug types
- Date of medication between 01 Apr 2012 and 31 Mar 2013
- Where patient is registered at General Practice

OR IN

BNF 2.9 Contraindications
ASPIRE Study / 13

- Registered before 01 Apr 2013
- Where patient is registered at General Practice

IN

TXDIPY in the last 12 m
ASPIRE Study / 13

- Has a Read code in the TXDIPY (Dipyridamole contraindications: expiring) QOF cluster
  Show read codes in cluster TXDIPY.
- Selecting only the most recent matching code
- Date of Read code between 01 Apr 2012 and 31 Mar 2013

AND IN

COMBCLOEXC in the last 12 m
ASPIRE Study / 13

- Has a Read code in the COMBCLOEXC (Combined Clopidogrel contraindication codes) QOF cluster
  Show read codes in cluster COMBCLOEXC.
- Selecting only the most recent matching code
- Date of Read code between 01 Apr 2012 and 31 Mar 2013

AND IN

COMSALEXC in the last 12 m
ASPIRE Study / 13

- Has a Read code in the COMBSALEXC (Combined Salicylate contraindication codes) QOF cluster
  Show read codes in cluster COMBSALEXC.
- Selecting only the most recent matching code
- Date of Read code between 01 Apr 2012 and 31 Mar 2013

AND IN

13D3+4. AF CHADs2 =1
ASPIRE Study / 13

- Registered before 01 Apr 2013
- Where patient is registered at General Practice

IN

AF001 - Register
ASPIRE Study / 13

- Has a Read code in the DRAFIB1 (Atrial fibrillation codes) QOF cluster
  Show read codes in cluster DRAFIB1.
- Selecting only the most recent matching code
- Without a more recent Read code in the DRAFIB2 (Atrial fibrillation resolved codes) QOF cluster
- Date of Read code before 01 Apr 2013

AND IN

1. CHAD2 Score = 1 (with AF)
ASPIRE Study / 13 zjoins

IN

CHAD2 Score of 1
ASPIRE Study / 13 zjoins

- Where patient is registered at General Practice

IN

CHAD2 Score 1 or above - any one of HF, Hyp, Diab or Over 75
ASPIRE Study / 13 zjoins

- Where patient is registered at General

Where patient is registered at General Practice

IN

Diabetes diagnosis
ASPIRE Study / 13 zjoins

Has a Read code in...Exact Read Codes:
[Brittle] and/or [labile diabetes] (66AJ1)
Diabetes mellitus (C10..)
Diabetes mellitus with no mention of complication (C100.)
Diabetes mellitus NOS with no mention of complication (C100z)
Other specified diabetes mellitus with coma (C103y)
Other specified diabetes mellitus with multiple comps (C108y)
Unspecified diabetes mellitus with multiple complications (C108z)
Other specified diabetes mellitus with other spec comps (C10yy)
[X]Other specified diabetes mellitus (Cyu20)
[X]Unspecified diabetes mellitus with renal complications (Cyu23)
[X]Pre-existing diabetes mellitus, unspecified (Lyu29)
Insulin treated Type 2 diabetes mellitus (X40J6)
Diabetes-deafness syndrome maternally transmitted (X40JZ)
Diabetes mellitus, juvenile type, no mention of complication (XE10E)
Diabetes mellitus, adult onset, no mention of complication (XE10F)
Diabetes with other complications (XE12M)
Diabetes mellitus with gangrene (XM1Qx)
Diabetes mellitus due to insulin receptor antibodies (XSETp)
Maternally inherited diabetes mellitus (XaOPt)
Read Codes and Children:
Diabetes mellitus with ophthalmic manifestation (C105.)
Diabetes mellitus with other specified manifestation (C10y.)
Diabetes mellitus with unspecified complication (C10z.)
Neonatal diabetes mellitus (Q441.)
Type I diabetes mellitus (X40J4)
Type II diabetes mellitus (X40J5)
Malnutrition-related diabetes mellitus (X40J7)
Secondary diabetes mellitus (X40JA)
Genetic syndromes of diabetes mellitus (X40JG)
Abnormal metabolic state in diabetes mellitus (X40Ja)
Diabetes mellitus with renal manifestation (XE10G)
Diabetes mellitus with neurological manifestation (XE10H)
Diabetes mellitus with peripheral circulatory disorder (XE10I)
Unstable diabetes (XM1Xk)

Where patient is registered at General Practice

OR IN

Hypertension diagnosis
ASPIRE Study / 13 zjoins

Has a Read code in...Exact Read Codes:
Systolic hypertension (G202.)
Secondary hypertension (G24..)
Hypertension secondary to endocrine disorders (G244.)
Secondary hypertension NOS (G24z.)
Hypertension secondary to drug (G24z1)
[X]Other secondary hypertension (Gyu20)
[X]Hypertension secondary to other renal disorders (Gyu21)
Pre-exist 2ndry hypertens comp preg childbth and puerprum (L1282)
Hypertension (XE0Ub)
Diastolic hypertension (XSDSb)
Labile hypertension (Xa0Cs)
Malignant hypertension (Xa3fQ)
Read Codes and Children:
Hypertensive disease (G2...)
Malignant secondary hypertension (G240.)
Secondary benign hypertension (G241.)
Essential hypertension (XE0Uc)
Renovascular hypertension (Xa0kX)

Where patient is registered at General Practice

OR IN

Over 75

ASPIRE Study / 13 zjoins

Current age > 75 years

Where patient is registered at General Practice

OR IN

Heart Failure diagnosis
ASPIRE Study / 13 zjoins

Has a Read code in...Exact Read Codes:
Heart failure (G58..)
Decompensated cardiac failure (G5802)
Compensated cardiac failure (G5803)
Acute heart failure (G582.)
Heart failure as a complication of care (X202k)
Right ventricular failure (X202l)
Heart failure NOS (XE0V9)
Refractory heart failure (XaEgY)
New York Heart Association classification - class I (XaJ9G)
New York Heart Association classification - class II (XaJ9H)
New York Heart Association classification - class III (XaJ9I)
New York Heart Association classification - class IV (XaJ9J)
Read Codes and Children:
Biventricular failure (XE0V8)
Left ventricular failure (XE2QG)

Where patient is registered at General Practice

NOT IN

CHAD2 Score 2 or above
ASPIRE Study / 13 zjoins

Where patient is registered at General Practice

IN

CHAD2 Score 2 or above - any two of HF, Hyp, Diab or Over 75
ASPIRE Study / 13 zjoins

Where patient is registered at General Practice

IN

Hypertension and Over 75s
ASPIRE Study / 13 zjoins

Where patient is registered at General Practice

IN

Over 75
ASPIRE Study / 13 zjoins

Current age > 75 years

Where patient is registered at General Practice

AND IN

Hypertension diagnosis
ASPIRE Study / 13 zjoins

Has a Read code in...Exact Read Codes:
Systolic hypertension (G202.)
Secondary hypertension (G24..)
Hypertension secondary to endocrine disorders (G244.)
Secondary hypertension NOS (G24z.)
Hypertension secondary to drug (G24z1)
[X]Other secondary hypertension (Gyu20)
[X]Hypertension secondary to other renal disorders (Gyu21)
Pre-exist 2ndry hypertens comp preg childbth and puerprum (L1282)
Hypertension (XE0Ub)
Diastolic hypertension (XSDSb)
Labile hypertension (Xa0Cs)
Malignant hypertension (Xa3fQ)
Read Codes and Children:
Hypertensive disease (G2...)
Malignant secondary hypertension (G240.)
Secondary benign hypertension (G241.)
Essential hypertension (XE0Uc)
Renovascular hypertension (Xa0kX)

Where patient is registered at General Practice

OR IN

Hypertension and Heart Failure
ASPIRE Study / 13 zjoins

Where patient is registered at General Practice

IN

Hypertension diagnosis
ASPIRE Study / 13 zjoins

Has a Read code in...Exact Read Codes:
Systolic hypertension (G202.)
Secondary hypertension (G24..)
Hypertension secondary to endocrine disorders (G244.)
Secondary hypertension NOS (G24z.)
Hypertension secondary to drug (G24z1)
[X]Other secondary hypertension (Gyu20)
[X]Hypertension secondary to other renal disorders (Gyu21)
Pre-exist 2ndry hypertens comp preg childbth and puerprum (L1282)
Hypertension (XE0Ub)
Diastolic hypertension (XSDSb)
Labile hypertension (Xa0Cs)
Malignant hypertension (Xa3fQ)
Read Codes and Children:
Hypertensive disease (G2...)
Malignant secondary hypertension (G240.)
Secondary benign hypertension (G241.)
Essential hypertension (XE0Uc)
Renovascular hypertension (Xa0kX)
Where patient is registered at General Practice

AND IN

Heart Failure diagnosis
ASPIRE Study / 13 zjoins

Has a Read code in...Exact Read Codes:
Heart failure (G58..)
Decompensated cardiac failure (G5802)
Compensated cardiac failure (G5803)
Acute heart failure (G582.)
Heart failure as a complication of care (X202k)
Right ventricular failure (X202l)
Heart failure NOS (XE0V9)
Refractory heart failure (XaEgY)
New York Heart Association classification - class I (XaJ9G)
New York Heart Association classification - class II (XaJ9H)
New York Heart Association classification - class III (XaJ9I)
New York Heart Association classification - class IV (XaJ9J)
Read Codes and Children:
Biventricular failure (XE0V8)
Left ventricular failure (XE2QG)
Where patient is registered at General Practice

OR IN

Heart Failure and Over 75s
ASPIRE Study / 13 zjoins

Where patient is registered at General Practice

IN

Over 75
ASPIRE Study / 13 zjoins

Current age > 75 years
Where patient is registered at General Practice

AND IN

Heart Failure diagnosis
ASPIRE Study / 13 zjoins

Has a Read code in...Exact Read Codes:
Heart failure (G58..)
Decompensated cardiac failure (G5802)
Compensated cardiac failure (G5803)
Acute heart failure (G582.)
Heart failure as a complication of care (X202k)
Right ventricular failure (X202l)
Heart failure NOS (XE0V9)
Refractory heart failure (XaEgY)
New York Heart Association classification - class I (XaJ9G)
New York Heart Association classification - class II (XaJ9H)
New York Heart Association classification - class III (XaJ9I)
New York Heart Association classification - class IV (XaJ9J)
Read Codes and Children:
Biventricular failure (XE0V8)
Left ventricular failure (XE2QG)
Where patient is registered at General Practice

OR IN

Diabetes and Over 75s
ASPIRE Study / 13 zjoins

Where patient is registered at General Practice

IN

Diabetes diagnosis
ASPIRE Study / 13 zjoins

Has a Read code in...Exact Read Codes:
[Brittle] and/or [labile diabetes] (66AJ1)
Diabetes mellitus (C10..)
Diabetes mellitus with no mention of complication (C100.)
Diabetes mellitus NOS with no mention of complication (C100z)
Other specified diabetes mellitus with coma (C103y)
Other specified diabetes mellitus with multiple comps (C108y)
Unspecified diabetes mellitus with multiple complications (C108z)
Other specified diabetes mellitus with other spec comps (C10yy)
[X]Other specified diabetes mellitus (Cyu20)
[X]Unspecified diabetes mellitus with renal complications (Cyu23)
[X]Pre-existing diabetes mellitus, unspecified (Lyu29)
Insulin treated Type 2 diabetes mellitus (X40J6)
Diabetes-deafness syndrome maternally transmitted (X40JZ)
Diabetes mellitus, juvenile type, no mention of complication (XE10E)
Diabetes mellitus, adult onset, no mention of complication (XE10F)
Diabetes with other complications (XE12M)
Diabetes mellitus with gangrene (XM1Qx)
Diabetes mellitus due to insulin receptor antibodies (XSETp)
Maternally inherited diabetes mellitus (XaOPt)
Read Codes and Children:
Diabetes mellitus with ophthalmic manifestation (C105.)
Diabetes mellitus with other specified manifestation (C10y.)
Diabetes mellitus with unspecified complication (C10z.)
Neonatal diabetes mellitus (Q441.)
Type I diabetes mellitus (X40J4)
Type II diabetes mellitus (X40J5)
Malnutrition-related diabetes mellitus (X40J7)
Secondary diabetes mellitus (X40JA)
Genetic syndromes of diabetes mellitus (X40JG)
Abnormal metabolic state in diabetes mellitus (X40Ja)
Diabetes mellitus with renal manifestation (XE10G)
Diabetes mellitus with neurological manifestation (XE10H)
Diabetes mellitus with peripheral circulatory disorder (XE10I)
Unstable diabetes (XM1Xk)

Where patient is registered at General Practice

AND IN

Over 75
ASPIRE Study / 13 zjoins

Current age > 75 years
Where patient is registered at General Practice

OR IN

Diabetes and Hypertension
ASPIRE Study / 13 zjoins

Where patient is registered at General Practice

IN

Diabetes diagnosis
ASPIRE Study / 13 zjoins

Has a Read code in...Exact Read Codes:
[Brittle] and/or [labile diabetes] (66AJ1)
Diabetes mellitus (C10..)
Diabetes mellitus with no mention of complication (C100.)
Diabetes mellitus NOS with no mention of complication (C100z)
Other specified diabetes mellitus with coma (C103y)
Other specified diabetes mellitus with multiple comps (C108y)
Unspecified diabetes mellitus with multiple [illegible]

complications (C108z)
Other specified diabetes mellitus with other spec comps (C10yy)
[X]Other specified diabetes mellitus (Cyu20)
[X]Unspecified diabetes mellitus with renal complications (Cyu23)
[X]Pre-existing diabetes mellitus, unspecified (Lyu29)
Insulin treated Type 2 diabetes mellitus (X40J6)
Diabetes-deafness syndrome maternally transmitted (X40JZ)
Diabetes mellitus, juvenile type, no mention of complication (XE10E)
Diabetes mellitus, adult onset, no mention of complication (XE10F)
Diabetes with other complications (XE12M)
Diabetes mellitus with gangrene (XM1Qx)
Diabetes mellitus due to insulin receptor antibodies (XSETp)
Maternally inherited diabetes mellitus (XaOPt)
Read Codes and Children:
Diabetes mellitus with ophthalmic manifestation (C105.)
Diabetes mellitus with other specified manifestation (C10y.)
Diabetes mellitus with unspecified complication (C10z.)
Neonatal diabetes mellitus (Q441.)
Type I diabetes mellitus (X40J4)
Type II diabetes mellitus (X40J5)
Malnutrition-related diabetes mellitus (X40J7)
Secondary diabetes mellitus (X40JA)
Genetic syndromes of diabetes mellitus (X40JG)
Abnormal metabolic state in diabetes mellitus (X40Ja)
Diabetes mellitus with renal manifestation (XE10G)
Diabetes mellitus with neurological manifestation (XE10H)
Diabetes mellitus with peripheral circulatory disorder (XE10I)
Unstable diabetes (XM1Xk)
Where patient is registered at General Practice

AND IN

Hypertension diagnosis
ASPIRE Study / 13 zjoins

Has a Read code in...Exact Read Codes:
Systolic hypertension (G202.)
Secondary hypertension (G24..)
Hypertension secondary to endocrine disorders (G244.)
Secondary hypertension NOS (G24z.)
Hypertension secondary to drug (G24z1)
[X]Other secondary hypertension (Gyu20)
[X]Hypertension secondary to other renal disorders (Gyu21)
Pre-exist 2ndry hypertens comp preg childbth and puerprum (L1282)
Hypertension (XE0Ub)
Diastolic hypertension (XSDSb)
Labile hypertension (Xa0Cs)
Malignant hypertension (Xa3fQ)
Read Codes and Children:
Hypertensive disease (G2...)
Malignant secondary hypertension (G240.)
Secondary benign hypertension (G241.)
Essential hypertension (XE0Uc)
Renovascular hypertension (Xa0kX)
Where patient is registered at General Practice

OR IN

Diabetes and Heart Failure
ASPIRE Study / 13 zjoins

Where patient is registered at General Practice

IN

Diabetes diagnosis
ASPIRE Study / 13 zjoins

Has a Read code in...Exact Read Codes:
[Brittle] and/or [labile diabetes] (66AJ1)
Diabetes mellitus (C10..)
Diabetes mellitus with no mention of complication (C100.)
Diabetes mellitus NOS with no mention of complication (C100z)
Other specified diabetes mellitus with coma (C103y)
Other specified diabetes mellitus with

multiple comps (C108y)
Unspecified diabetes mellitus with multiple complications (C108z)
Other specified diabetes mellitus with other spec comps (C10yy)
[X]Other specified diabetes mellitus (Cyu20)
[X]Unspecified diabetes mellitus with renal complications (Cyu23)
[X]Pre-existing diabetes mellitus, unspecified (Lyu29)
Insulin treated Type 2 diabetes mellitus (X40J6)
Diabetes-deafness syndrome maternally transmitted (X40JZ)
Diabetes mellitus, juvenile type, no mention of complication (XE10E)
Diabetes mellitus, adult onset, no mention of complication (XE10F)
Diabetes with other complications (XE12M)
Diabetes mellitus with gangrene (XM1Qx)
Diabetes mellitus due to insulin receptor antibodies (XSETp)
Maternally inherited diabetes mellitus (XaOPt)
Read Codes and Children:
Diabetes mellitus with ophthalmic manifestation (C105.)
Diabetes mellitus with other specified manifestation (C10y.)
Diabetes mellitus with unspecified complication (C10z.)
Neonatal diabetes mellitus (Q441.)
Type I diabetes mellitus (X40J4)
Type II diabetes mellitus (X40J5)
Malnutrition-related diabetes mellitus (X40J7)
Secondary diabetes mellitus (X40JA)
Genetic syndromes of diabetes mellitus (X40JG)
Abnormal metabolic state in diabetes mellitus (X40Ja)
Diabetes mellitus with renal manifestation (XE10G)
Diabetes mellitus with neurological manifestation (XE10H)
Diabetes mellitus with peripheral circulatory disorder (XE10I)
Unstable diabetes (XM1Xk)

Where patient is registered at General Practice

AND IN

Heart Failure diagnosis
ASPIRE Study / 13 zjoins

Has a Read code in...Exact Read Codes:
Heart failure (G58..)
Decompensated cardiac failure (G5802)
Compensated cardiac failure (G5803)
Acute heart failure (G582.)
Heart failure as a complication of care (X202k)
Right ventricular failure (X202l)
Heart failure NOS (XE0V9)
Refractory heart failure (XaEgY)
New York Heart Association classification - class I (XaJ9G)
New York Heart Association classification - class II (XaJ9H)
New York Heart Association classification - class III (XaJ9I)
New York Heart Association classification - class IV (XaJ9J)
Read Codes and Children:
Biventricular failure (XE0V8)
Left ventricular failure (XE2QG)

Where patient is registered at General Practice

OR IN

CVA and/or TIA
ASPIRE Study / 13 zjoins

Where patient is registered at General Practice

IN

TIA diagnosis
ASPIRE Study / 13 zjoins

Has a Read code of Transient ischaemic attack (XE0VK) or one of its children

Where patient is registered at General Practice

OR IN

CVA diagnosis
ASPIRE Study / 13 zjoins

[illegible]

Has a Read code of Cerebrovascular accident (X00D1) or one of its children

Where patient is registered at General Practice

AND IN

Atrial Fibrilation diagnosis
ASPIRE Study / 13 zjoins

Has a Read code in the DRAFIB1 (Atrial fibrillation codes) QOF cluster
Show read codes in cluster DRAFIB1.

- Selecting only the most recent matching code
- Without a more recent Read code in the DRAFIB2 (Atrial fibrillation resolved codes) QOF cluster

Where patient is registered at General Practice
