# Supplementary material for: Developing ‘high impact’ guideline-based quality indicators for UK primary care: a multi-stage consensus process
Source: BMC Fam Pract. 2015 Oct 28;16:156. doi: 10.1186/s12875-015-0350-6 (PMC4624600; doi:10.1186/s12875-015-0350-6)
Supplement: Additional file 4 — Folder containing SystmOne™ search algorithms. (ZIP 12.7 mb) [file 12875_2015_350_MOESM4_ESM.zip › Aspire S1 diagrams tw edired/13N6 (AF #39).pdf]

Mandatory In
Optional In
Not In

13N6. AF and CHADs 2 = 2>6 with Warfarin Rx (read code or Rx OR Contraindication)
ASPIRE Study / 13
Registered before 01 Apr 2013
Where patient is registered at General Practice

IN

13D5+6. AF Register and CHADs2 2>6
ASPIRE Study / 13
Registered before 01 Apr 2013
Where patient is registered at General Practice

IN

CHADs2 2>6
ASPIRE Study / 13

IN

2. CHAD2 Score = 2 (with AF)
ASPIRE Study / 13 zjoins
Where patient is registered at General Practice

IN

2h. All CHAD2 = 2 Combinations
ASPIRE Study / 13 zjoins
Where patient is registered at General Practice

IN

2g. DIAB and >75 not in (HF or HYP or CVA/TIA)
ASPIRE Study / 13 zjoins

IN

Double DIAB and >75
ASPIRE Study / 13 zjoins
Where patient is registered at General Practice

IN

Over 75
ASPIRE Study / 13 zjoins
Current age > 75 years
Where patient is registered at General Practice

AND IN

Diabetes diagnosis
ASPIRE Study / 13 zjoins
Has a Read code in...Exact Read Codes:
[Brittle] and/or [labile diabetes] (66AJ1)
Diabetes mellitus (C10..)
Diabetes mellitus with no mention of complication (C100.)
Diabetes mellitus NOS with no mention of complication (C100z)
Other specified diabetes mellitus with coma (C103y)
Other specified diabetes mellitus with multiple comps (C108y)
Unspecified diabetes mellitus with multiple complications (C108z)
Other specified diabetes mellitus with other spec comps (C10yy)
[X]Other specified diabetes mellitus (Cyu20)
[X]Unspecified diabetes mellitus with renal complications (Cyu23)
[X]Pre-existing diabetes mellitus, unspecified (Lyu29)
Insulin treated Type 2 diabetes mellitus (X40J6)
Diabetes-deafness syndrome maternally transmitted (X40JZ)
Diabetes mellitus, juvenile type, no mention of complication (XE10E)
Diabetes mellitus, adult onset, no mention of complication (XE10F)
Diabetes with other complications (XE12M)
Diabetes mellitus with gangrene (XM1Qx)
Diabetes mellitus due to insulin receptor antibodies (XSETp)
Maternally inherited diabetes mellitus (XaOPt)
Read Codes and Children:
Diabetes mellitus with ophthalmic manifestation (C105.)
Diabetes mellitus with other specified manifestation (C10y.)
Diabetes mellitus with unspecified complication (C10z.)
Neonatal diabetes mellitus (Q441.)

Neonatal diabetes mellitus ([illegible])
Type I diabetes mellitus (X40J4)
Type II diabetes mellitus (X40J5)
Malnutrition-related diabetes mellitus (X40J7)
Secondary diabetes mellitus (X40JA)
Genetic syndromes of diabetes mellitus (X40JG)
Abnormal metabolic state in diabetes mellitus (X40Ja)
Diabetes mellitus with renal manifestation (XE10G)
Diabetes mellitus with neurological manifestation (XE10H)
Diabetes mellitus with peripheral circulatory disorder (XE10I)
Unstable diabetes (XM1Xk)

Where patient is registered at General Practice

NOT IN

Triple HF or HYP or CVA/TIA
ASPIRE Study / 13 zjoins

Where patient is registered at General Practice

IN

Heart Failure diagnosis
ASPIRE Study / 13 zjoins

Has a Read code in...Exact Read Codes:
Heart failure (G58..)
Decompensated cardiac failure (G5802)
Compensated cardiac failure (G5803)
Acute heart failure (G582.)
Heart failure as a complication of care (X202k)
Right ventricular failure (X202l)
Heart failure NOS (XE0V9)
Refractory heart failure (XaEgY)
New York Heart Association classification - class I (XaJ9G)
New York Heart Association classification - class II (XaJ9H)
New York Heart Association classification - class III (XaJ9I)
New York Heart Association classification - class IV (XaJ9J)
Read Codes and Children:
Biventricular failure (XE0V8)
Left ventricular failure (XE2QG)

Where patient is registered at General Practice

OR IN

CVA and/or TIA
ASPIRE Study / 13 zjoins

Where patient is registered at General Practice

IN

TIA diagnosis
ASPIRE Study / 13 zjoins

Has a Read code of Transient ischaemic attack (XE0VK) or one of its children

Where patient is registered at General Practice

OR IN

CVA diagnosis
ASPIRE Study / 13 zjoins

Has a Read code of Cerebrovascular accident (X00D1) or one of its children

Where patient is registered at General Practice

OR IN

Hypertension diagnosis
ASPIRE Study / 13 zjoins

Has a Read code in...Exact Read Codes:
Systolic hypertension (G202.)
Secondary hypertension (G24..)
Hypertension secondary to endocrine disorders (G244.)
Secondary hypertension NOS (G24z.)
Hypertension secondary to drug (G24z1)
[X]Other secondary hypertension (Gyu20)
[X]Hypertension secondary to other renal disorders (Gyu21)
Pre-exist 2ndry hypertens comp preg childbth and puerprum (L1282)
Hypertension (XE0Ub)
Diastolic hypertension (XSDSb)
Labile hypertension (Xa0Cs)
Malignant hypertension (Xa3fQ)
Read Codes and Children:
Hypertensive disease (G2...)

Malignant secondary hypertension (G240.)
Secondary benign hypertension (G241.)
Essential hypertension (XE0Uc)
Renovascular hypertension (Xa0kX)
Where patient is registered at General Practice

OR IN

2f. HYP and >75 not in (HF or DIAB or CVA/TIA)
ASPIRE Study / 13 zjoins

IN

Double HYP and >75
ASPIRE Study / 13 zjoins
Where patient is registered at General Practice

IN

Hypertension diagnosis
ASPIRE Study / 13 zjoins
Has a Read code in...Exact Read Codes:
Systolic hypertension (G202.)
Secondary hypertension (G24..)
Hypertension secondary to endocrine disorders (G244.)
Secondary hypertension NOS (G24z.)
Hypertension secondary to drug (G24z1)
[X]Other secondary hypertension (Gyu20)
[X]Hypertension secondary to other renal disorders (Gyu21)
Pre-exist 2ndry hypertens comp preg childbth and puerprum (L1282)
Hypertension (XE0Ub)
Diastolic hypertension (XSDSb)
Labile hypertension (Xa0Cs)
Malignant hypertension (Xa3fQ)
Read Codes and Children:
Hypertensive disease (G2...)
Malignant secondary hypertension (G240.)
Secondary benign hypertension (G241.)
Essential hypertension (XE0Uc)
Renovascular hypertension (Xa0kX)
Where patient is registered at General Practice

AND IN

Over 75
ASPIRE Study / 13 zjoins
Current age > 75 years
Where patient is registered at General Practice

NOT IN

Triple HF or DIAB or CVA/TIA
ASPIRE Study / 13 zjoins
Where patient is registered at General Practice

IN

Heart Failure diagnosis
ASPIRE Study / 13 zjoins
Has a Read code in...Exact Read Codes:
Heart failure (G58..)
Decompensated cardiac failure (G5802)
Compensated cardiac failure (G5803)
Acute heart failure (G582.)
Heart failure as a complication of care (X202k)
Right ventricular failure (X202l)
Heart failure NOS (XE0V9)
Refractory heart failure (XaEgY)
New York Heart Association classification - class I (XaJ9G)
New York Heart Association classification - class II (XaJ9H)
New York Heart Association classification - class III (XaJ9I)
New York Heart Association classification - class IV (XaJ9J)
Read Codes and Children:
Biventricular failure (XE0V8)
Left ventricular failure (XE2QG)
Where patient is registered at General Practice

OR IN

CVA and/or TIA
ASPIRE Study / 13 zjoins
Where patient is registered at General Practice

IN

TIA diagnosis
ASPIRE Study / 13 zjoins
[illegible]

Has a Read code of Transient ischaemic attack (XE0VK) or one of its children
Where patient is registered at General Practice

OR IN

CVA diagnosis
ASPIRE Study / 13 zjoins

Has a Read code of Cerebrovascular accident (X00D1) or one of its children
Where patient is registered at General Practice

OR IN

Diabetes diagnosis
ASPIRE Study / 13 zjoins

Has a Read code in...Exact Read Codes:
[Brittle] and/or [labile diabetes] (66AJ1)
Diabetes mellitus (C10..)
Diabetes mellitus with no mention of complication (C100.)
Diabetes mellitus NOS with no mention of complication (C100z)
Other specified diabetes mellitus with coma (C103y)
Other specified diabetes mellitus with multiple comps (C108y)
Unspecified diabetes mellitus with multiple complications (C108z)
Other specified diabetes mellitus with other spec comps (C10yy)
[X]Other specified diabetes mellitus (Cyu20)
[X]Unspecified diabetes mellitus with renal complications (Cyu23)
[X]Pre-existing diabetes mellitus, unspecified (Lyu29)
Insulin treated Type 2 diabetes mellitus (X40J6)
Diabetes-deafness syndrome maternally transmitted (X40JZ)
Diabetes mellitus, juvenile type, no mention of complication (XE10E)
Diabetes mellitus, adult onset, no mention of complication (XE10F)
Diabetes with other complications (XE12M)
Diabetes mellitus with gangrene (XM1Qx)
Diabetes mellitus due to insulin receptor antibodies (XSETp)
Maternally inherited diabetes mellitus (XaOPt)
Read Codes and Children:
Diabetes mellitus with ophthalmic manifestation (C105.)
Diabetes mellitus with other specified manifestation (C10y.)
Diabetes mellitus with unspecified complication (C10z.)
Neonatal diabetes mellitus (Q441.)
Type I diabetes mellitus (X40J4)
Type II diabetes mellitus (X40J5)
Malnutrition-related diabetes mellitus (X40J7)
Secondary diabetes mellitus (X40JA)
Genetic syndromes of diabetes mellitus (X40JG)
Abnormal metabolic state in diabetes mellitus (X40Ja)
Diabetes mellitus with renal manifestation (XE10G)
Diabetes mellitus with neurological manifestation (XE10H)
Diabetes mellitus with peripheral circulatory disorder (XE10I)
Unstable diabetes (XM1Xk)
Where patient is registered at General Practice

OR IN

2e. HYP and DIAB not in (HF or >75 or CVA/TIA)
ASPIRE Study / 13 zjoins

IN

Double HYP and DIAB
ASPIRE Study / 13 zjoins

Where patient is registered at General Practice

IN

Hypertension diagnosis
ASPIRE Study / 13 zjoins

Has a Read code in...Exact Read Codes:
Systolic hypertension (G202.)
Secondary hypertension (G24..)
Hypertension secondary to endocrine disorders (G244.)

Secondary hypertension NOS (G24z.)
Hypertension secondary to drug (G24z1)
[X]Other secondary hypertension (Gyu20)
[X]Hypertension secondary to other renal disorders (Gyu21)
Pre-exist 2ndry hypertens comp preg childbth and puerprum (L1282)
Hypertension (XE0Ub)
Diastolic hypertension (XSDSb)
Labile hypertension (Xa0Cs)
Malignant hypertension (Xa3fQ)
Read Codes and Children:
Hypertensive disease (G2...)
Malignant secondary hypertension (G240.)
Secondary benign hypertension (G241.)
Essential hypertension (XE0Uc)
Renovascular hypertension (Xa0kX)

Where patient is registered at General Practice

AND IN

Diabetes diagnosis
ASPIRE Study / 13 zjoins

Has a Read code in...Exact Read Codes:
[Brittle] and/or [labile diabetes] (66AJ1)
Diabetes mellitus (C10..)
Diabetes mellitus with no mention of complication (C100.)
Diabetes mellitus NOS with no mention of complication (C100z)
Other specified diabetes mellitus with coma (C103y)
Other specified diabetes mellitus with multiple comps (C108y)
Unspecified diabetes mellitus with multiple complications (C108z)
Other specified diabetes mellitus with other spec comps (C10yy)
[X]Other specified diabetes mellitus (Cyu20)
[X]Unspecified diabetes mellitus with renal complications (Cyu23)
[X]Pre-existing diabetes mellitus, unspecified (Lyu29)
Insulin treated Type 2 diabetes mellitus (X40J6)
Diabetes-deafness syndrome maternally transmitted (X40JZ)
Diabetes mellitus, juvenile type, no mention of complication (XE10E)
Diabetes mellitus, adult onset, no mention of complication (XE10F)
Diabetes with other complications (XE12M)
Diabetes mellitus with gangrene (XM1Qx)
Diabetes mellitus due to insulin receptor antibodies (XSETp)
Maternally inherited diabetes mellitus (XaOPt)
Read Codes and Children:
Diabetes mellitus with ophthalmic manifestation (C105.)
Diabetes mellitus with other specified manifestation (C10y.)
Diabetes mellitus with unspecified complication (C10z.)
Neonatal diabetes mellitus (Q441.)
Type I diabetes mellitus (X40J4)
Type II diabetes mellitus (X40J5)
Malnutrition-related diabetes mellitus (X40J7)
Secondary diabetes mellitus (X40JA)
Genetic syndromes of diabetes mellitus (X40JG)
Abnormal metabolic state in diabetes mellitus (X40Ja)
Diabetes mellitus with renal manifestation (XE10G)
Diabetes mellitus with neurological manifestation (XE10H)
Diabetes mellitus with peripheral circulatory disorder (XE10I)
Unstable diabetes (XM1Xk)

Where patient is registered at General Practice

NOT IN

Triple HF or >75 or CVA/TIA
ASPIRE Study / 13 zjoins

Where patient is registered at General Practice

IN

CVA and/or TIA
ASPIRE Study / 13 zjoins

Where patient is registered at General Practice

IN
TIA diagnosis
ASPIRE Study / 13 zjoins
Has a Read code of Transient ischaemic attack (XE0VK) or one of its children
Where patient is registered at General Practice

OR IN
CVA diagnosis
ASPIRE Study / 13 zjoins
Has a Read code of Cerebrovascular accident (X00D1) or one of its children
Where patient is registered at General Practice

OR IN
Heart Failure diagnosis
ASPIRE Study / 13 zjoins
Has a Read code in...Exact Read Codes:
Heart failure (G58..)
Decompensated cardiac failure (G5802)
Compensated cardiac failure (G5803)
Acute heart failure (G582.)
Heart failure as a complication of care (X202k)
Right ventricular failure (X202l)
Heart failure NOS (XE0V9)
Refractory heart failure (XaEgY)
New York Heart Association classification - class I (XaJ9G)
New York Heart Association classification - class II (XaJ9H)
New York Heart Association classification - class III (XaJ9I)
New York Heart Association classification - class IV (XaJ9J)
Read Codes and Children:
Biventricular failure (XE0V8)
Left ventricular failure (XE2QG)
Where patient is registered at General Practice

OR IN
Over 75
ASPIRE Study / 13 zjoins
Current age > 75 years
Where patient is registered at General Practice

OR IN
2d. HF and >75 not in (HYP or DIAB or CVA/TIA)
ASPIRE Study / 13 zjoins
Where patient is registered at General Practice

IN
Double HF and >75
ASPIRE Study / 13 zjoins
Where patient is registered at General Practice

IN
Heart Failure diagnosis
ASPIRE Study / 13 zjoins
Has a Read code in...Exact Read Codes:
Heart failure (G58..)
Decompensated cardiac failure (G5802)
Compensated cardiac failure (G5803)
Acute heart failure (G582.)
Heart failure as a complication of care (X202k)
Right ventricular failure (X202l)
Heart failure NOS (XE0V9)
Refractory heart failure (XaEgY)
New York Heart Association classification - class I (XaJ9G)
New York Heart Association classification - class II (XaJ9H)
New York Heart Association classification - class III (XaJ9I)
New York Heart Association classification - class IV (XaJ9J)
Read Codes and Children:
Biventricular failure (XE0V8)
Left ventricular failure (XE2QG)
Where patient is registered at General Practice

AND IN
Over 75
ASPIRE Study / 13 zjoins
Current age > 75 years
[illegible]

Where patient is registered at General Practice

NOT IN

Triple HYP or DIAB or CVA/TIA
ASPIRE Study / 13 zjoins

Where patient is registered at General Practice

IN

Hypertension diagnosis
ASPIRE Study / 13 zjoins

Has a Read code in...Exact Read Codes:
Systolic hypertension (G202.)
Secondary hypertension (G24..)
Hypertension secondary to endocrine disorders (G244.)
Secondary hypertension NOS (G24z.)
Hypertension secondary to drug (G24z1)
[X]Other secondary hypertension (Gyu20)
[X]Hypertension secondary to other renal disorders (Gyu21)
Pre-exist 2ndry hypertens comp preg childbth and puerprum (L1282)
Hypertension (XE0Ub)
Diastolic hypertension (XSDSb)
Labile hypertension (Xa0Cs)
Malignant hypertension (Xa3fQ)
Read Codes and Children:
Hypertensive disease (G2...)
Malignant secondary hypertension (G240.)
Secondary benign hypertension (G241.)
Essential hypertension (XE0Uc)
Renovascular hypertension (Xa0kX)

Where patient is registered at General Practice

OR IN

CVA and/or TIA
ASPIRE Study / 13 zjoins

Where patient is registered at General Practice

IN

TIA diagnosis
ASPIRE Study / 13 zjoins

Has a Read code of Transient ischaemic attack (XE0VK) or one of its children

Where patient is registered at General Practice

OR IN

CVA diagnosis
ASPIRE Study / 13 zjoins

Has a Read code of Cerebrovascular accident (X00D1) or one of its children

Where patient is registered at General Practice

OR IN

Diabetes diagnosis
ASPIRE Study / 13 zjoins

Has a Read code in...Exact Read Codes:
[Brittle] and/or [labile diabetes] (66AJ1)
Diabetes mellitus (C10..)
Diabetes mellitus with no mention of complication (C100.)
Diabetes mellitus NOS with no mention of complication (C100z)
Other specified diabetes mellitus with coma (C103y)
Other specified diabetes mellitus with multiple comps (C108y)
Unspecified diabetes mellitus with multiple complications (C108z)
Other specified diabetes mellitus with other spec comps (C10yy)
[X]Other specified diabetes mellitus (Cyu20)
[X]Unspecified diabetes mellitus with renal complications (Cyu23)
[X]Pre-existing diabetes mellitus, unspecified (Lyu29)
Insulin treated Type 2 diabetes mellitus (X40J6)
Diabetes-deafness syndrome maternally transmitted (X40JZ)
Diabetes mellitus, juvenile type, no mention of complication (XE10E)
Diabetes mellitus, adult onset, no mention of complication (XE10F)
Diabetes with other complications (XE12M)
Diabetes mellitus with gangrene (XM1Qx)
Diabetes mellitus due to insulin receptor antibodies (XSETp)
Maternally inherited diabetes mellitus (XaOPt)

[illegible]
Read Codes and Children:
Diabetes mellitus with ophthalmic manifestation (C105.)
Diabetes mellitus with other specified manifestation (C10y.)
Diabetes mellitus with unspecified complication (C10z.)
Neonatal diabetes mellitus (Q441.)
Type I diabetes mellitus (X40J4)
Type II diabetes mellitus (X40J5)
Malnutrition-related diabetes mellitus (X40J7)
Secondary diabetes mellitus (X40JA)
Genetic syndromes of diabetes mellitus (X40JG)
Abnormal metabolic state in diabetes mellitus (X40Ja)
Diabetes mellitus with renal manifestation (XE10G)
Diabetes mellitus with neurological manifestation (XE10H)
Diabetes mellitus with peripheral circulatory disorder (XE10I)
Unstable diabetes (XM1Xk)

Where patient is registered at General Practice

OR IN

2c. HF and DIAB not in (HYP or >75 or CVA/TIA)
ASPIRE Study / 13 zjoins

Where patient is registered at General Practice

IN

Double HF and DIAB
ASPIRE Study / 13 zjoins

Where patient is registered at General Practice

IN

Heart Failure diagnosis
ASPIRE Study / 13 zjoins

Has a Read code in...Exact Read Codes:
Heart failure (G58..)
Decompensated cardiac failure (G5802)
Compensated cardiac failure (G5803)
Acute heart failure (G582.)
Heart failure as a complication of care (X202k)
Right ventricular failure (X202l)
Heart failure NOS (XE0V9)
Refractory heart failure (XaEgY)
New York Heart Association classification - class I (XaJ9G)
New York Heart Association classification - class II (XaJ9H)
New York Heart Association classification - class III (XaJ9I)
New York Heart Association classification - class IV (XaJ9J)
Read Codes and Children:
Biventricular failure (XE0V8)
Left ventricular failure (XE2QG)

Where patient is registered at General Practice

AND IN

Diabetes diagnosis
ASPIRE Study / 13 zjoins

Has a Read code in...Exact Read Codes:
[Brittle] and/or [labile diabetes] (66AJ1)
Diabetes mellitus (C10..)
Diabetes mellitus with no mention of complication (C100.)
Diabetes mellitus NOS with no mention of complication (C100z)
Other specified diabetes mellitus with coma (C103y)
Other specified diabetes mellitus with multiple comps (C108y)
Unspecified diabetes mellitus with multiple complications (C108z)
Other specified diabetes mellitus with other spec comps (C10yy)
[X]Other specified diabetes mellitus (Cyu20)
[X]Unspecified diabetes mellitus with renal complications (Cyu23)
[X]Pre-existing diabetes mellitus, unspecified (Lyu29)
Insulin treated Type 2 diabetes mellitus (X40J6)
Diabetes-deafness syndrome maternally transmitted (X40JZ)
Diabetes mellitus, juvenile type, no mention [illegible]

of complication (XE10E)
Diabetes mellitus, adult onset, no mention of complication (XE10F)
Diabetes with other complications (XE12M)
Diabetes mellitus with gangrene (XM1Qx)
Diabetes mellitus due to insulin receptor antibodies (XSETp)
Maternally inherited diabetes mellitus (XaOPt)
Read Codes and Children:
Diabetes mellitus with ophthalmic manifestation (C105.)
Diabetes mellitus with other specified manifestation (C10y.)
Diabetes mellitus with unspecified complication (C10z.)
Neonatal diabetes mellitus (Q441.)
Type I diabetes mellitus (X40J4)
Type II diabetes mellitus (X40J5)
Malnutrition-related diabetes mellitus (X40J7)
Secondary diabetes mellitus (X40JA)
Genetic syndromes of diabetes mellitus (X40JG)
Abnormal metabolic state in diabetes mellitus (X40Ja)
Diabetes mellitus with renal manifestation (XE10G)
Diabetes mellitus with neurological manifestation (XE10H)
Diabetes mellitus with peripheral circulatory disorder (XE10I)
Unstable diabetes (XM1Xk)
Where patient is registered at General Practice

NOT IN

Triple HYP or >75 or CVA/TIA
ASPIRE Study / 13 zjoins
Where patient is registered at General Practice

IN

CVA and/or TIA
ASPIRE Study / 13 zjoins
Where patient is registered at General Practice

IN

TIA diagnosis
ASPIRE Study / 13 zjoins
Has a Read code of Transient ischaemic attack (XE0VK) or one of its children
Where patient is registered at General Practice

OR IN

CVA diagnosis
ASPIRE Study / 13 zjoins
Has a Read code of Cerebrovascular accident (X00D1) or one of its children
Where patient is registered at General Practice

OR IN

Over 75
ASPIRE Study / 13 zjoins
Current age > 75 years
Where patient is registered at General Practice

OR IN

Hypertension diagnosis
ASPIRE Study / 13 zjoins
Has a Read code in...Exact Read Codes:
Systolic hypertension (G202.)
Secondary hypertension (G24..)
Hypertension secondary to endocrine disorders (G244.)
Secondary hypertension NOS (G24z.)
Hypertension secondary to drug (G24z1)
[X]Other secondary hypertension (Gyu20)
[X]Hypertension secondary to other renal disorders (Gyu21)
Pre-exist 2ndry hypertens comp preg childbth and puerprum (L1282)
Hypertension (XE0Ub)
Diastolic hypertension (XSDSb)
Labile hypertension (Xa0Cs)
Malignant hypertension (Xa3fQ)
Read Codes and Children:
Hypertensive disease (G2...)
Malignant secondary hypertension (G240.)
Secondary benign hypertension (G241.)
Essential hypertension (XE0Uc)
Renovascular hypertension (Xa0kX)

Renovascular hypertension (Xa0kX)
Where patient is registered at General Practice

OR IN

2b. HF and HYP not in (DIAB or >75 or CVA/TIA)
ASPIRE Study / 13 zjoins
Where patient is registered at General Practice

IN

Double HF and HYP
ASPIRE Study / 13 zjoins
Where patient is registered at General Practice

IN

Heart Failure diagnosis
ASPIRE Study / 13 zjoins
Has a Read code in...Exact Read Codes:
Heart failure (G58..)
Decompensated cardiac failure (G5802)
Compensated cardiac failure (G5803)
Acute heart failure (G582.)
Heart failure as a complication of care (X202k)
Right ventricular failure (X202l)
Heart failure NOS (XE0V9)
Refractory heart failure (XaEgY)
New York Heart Association classification - class I (XaJ9G)
New York Heart Association classification - class II (XaJ9H)
New York Heart Association classification - class III (XaJ9I)
New York Heart Association classification - class IV (XaJ9J)
Read Codes and Children:
Biventricular failure (XE0V8)
Left ventricular failure (XE2QG)
Where patient is registered at General Practice

AND IN

Hypertension diagnosis
ASPIRE Study / 13 zjoins
Has a Read code in...Exact Read Codes:
Systolic hypertension (G202.)
Secondary hypertension (G24..)
Hypertension secondary to endocrine disorders (G244.)
Secondary hypertension NOS (G24z.)
Hypertension secondary to drug (G24z1)
[X]Other secondary hypertension (Gyu20)
[X]Hypertension secondary to other renal disorders (Gyu21)
Pre-exist 2ndry hypertens comp preg childbth and puerprum (L1282)
Hypertension (XE0Ub)
Diastolic hypertension (XSDSb)
Labile hypertension (Xa0Cs)
Malignant hypertension (Xa3fQ)
Read Codes and Children:
Hypertensive disease (G2...)
Malignant secondary hypertension (G240.)
Secondary benign hypertension (G241.)
Essential hypertension (XE0Uc)
Renovascular hypertension (Xa0kX)
Where patient is registered at General Practice

NOT IN

Triple DIAB or >75 or CVA/TIA
ASPIRE Study / 13 zjoins
Where patient is registered at General Practice

IN

Diabetes diagnosis
ASPIRE Study / 13 zjoins
Has a Read code in...Exact Read Codes:
[Brittle] and/or [labile diabetes] (66AJ1)
Diabetes mellitus (C10..)
Diabetes mellitus with no mention of complication (C100.)
Diabetes mellitus NOS with no mention of complication (C100z)
Other specified diabetes mellitus with coma (C103y)
Other specified diabetes mellitus with multiple comps (C108y)
Unspecified diabetes mellitus with multiple complications (C108z)
Other specified diabetes mellitus with other spec comps (C10yy)

[X]Other specified diabetes mellitus (Cyu20)
[X]Unspecified diabetes mellitus with renal complications (Cyu23)
[X]Pre-existing diabetes mellitus, unspecified (Lyu29)
Insulin treated Type 2 diabetes mellitus (X40J6)
Diabetes-deafness syndrome maternally transmitted (X40JZ)
Diabetes mellitus, juvenile type, no mention of complication (XE10E)
Diabetes mellitus, adult onset, no mention of complication (XE10F)
Diabetes with other complications (XE12M)
Diabetes mellitus with gangrene (XM1Qx)
Diabetes mellitus due to insulin receptor antibodies (XSETp)
Maternally inherited diabetes mellitus (XaOPt)
Read Codes and Children:
Diabetes mellitus with ophthalmic manifestation (C105.)
Diabetes mellitus with other specified manifestation (C10y.)
Diabetes mellitus with unspecified complication (C10z.)
Neonatal diabetes mellitus (Q441.)
Type I diabetes mellitus (X40J4)
Type II diabetes mellitus (X40J5)
Malnutrition-related diabetes mellitus (X40J7)
Secondary diabetes mellitus (X40JA)
Genetic syndromes of diabetes mellitus (X40JG)
Abnormal metabolic state in diabetes mellitus (X40Ja)
Diabetes mellitus with renal manifestation (XE10G)
Diabetes mellitus with neurological manifestation (XE10H)
Diabetes mellitus with peripheral circulatory disorder (XE10I)
Unstable diabetes (XM1Xk)
Where patient is registered at General Practice

OR IN
Over 75
ASPIRE Study / 13 zjoins
Current age > 75 years
Where patient is registered at General Practice

OR IN
CVA and/or TIA
ASPIRE Study / 13 zjoins
Where patient is registered at General Practice

IN
TIA diagnosis
ASPIRE Study / 13 zjoins
Has a Read code of Transient ischaemic attack (XE0VK) or one of its children
Where patient is registered at General Practice

OR IN
CVA diagnosis
ASPIRE Study / 13 zjoins
Has a Read code of Cerebrovascular accident (X00D1) or one of its children
Where patient is registered at General Practice

OR IN
2a. CVA/TIA not in (HF or HYP or DIAB or >75)
ASPIRE Study / 13 zjoins
Where patient is registered at General Practice

IN
CVA and/or TIA
ASPIRE Study / 13 zjoins
Where patient is registered at General Practice

IN
TIA diagnosis
ASPIRE Study / 13 zjoins
Has a Read code of Transient ischaemic attack (XE0VK) or one of its children
Where patient is registered at General Practice

OR IN

CVA diagnosis
ASPIRE Study / 13 zjoins

Has a Read code of Cerebrovascular accident (X00D1) or one of its children

Where patient is registered at General Practice

NOT IN

Quadruple HF or HYP or DIAB or >75
ASPIRE Study / 13 zjoins

Where patient is registered at General Practice

IN

Heart Failure diagnosis
ASPIRE Study / 13 zjoins

Has a Read code in...Exact Read Codes:
Heart failure (G58..)
Decompensated cardiac failure (G5802)
Compensated cardiac failure (G5803)
Acute heart failure (G582.)
Heart failure as a complication of care (X202k)
Right ventricular failure (X202l)
Heart failure NOS (XE0V9)
Refractory heart failure (XaEgY)
New York Heart Association classification - class I (XaJ9G)
New York Heart Association classification - class II (XaJ9H)
New York Heart Association classification - class III (XaJ9I)
New York Heart Association classification - class IV (XaJ9J)
Read Codes and Children:
Biventricular failure (XE0V8)
Left ventricular failure (XE2QG)

Where patient is registered at General Practice

OR IN

Hypertension diagnosis
ASPIRE Study / 13 zjoins

Has a Read code in...Exact Read Codes:
Systolic hypertension (G202.)
Secondary hypertension (G24..)
Hypertension secondary to endocrine disorders (G244.)
Secondary hypertension NOS (G24z.)
Hypertension secondary to drug (G24z1)
[X]Other secondary hypertension (Gyu20)
[X]Hypertension secondary to other renal disorders (Gyu21)
Pre-exist 2ndry hypertens comp preg childbth and puerprum (L1282)
Hypertension (XE0Ub)
Diastolic hypertension (XSDSb)
Labile hypertension (Xa0Cs)
Malignant hypertension (Xa3fQ)
Read Codes and Children:
Hypertensive disease (G2...)
Malignant secondary hypertension (G240.)
Secondary benign hypertension (G241.)
Essential hypertension (XE0Uc)
Renovascular hypertension (Xa0kX)

Where patient is registered at General Practice

OR IN

Diabetes diagnosis
ASPIRE Study / 13 zjoins

Has a Read code in...Exact Read Codes:
[Brittle] and/or [labile diabetes] (66AJ1)
Diabetes mellitus (C10..)
Diabetes mellitus with no mention of complication (C100.)
Diabetes mellitus NOS with no mention of complication (C100z)
Other specified diabetes mellitus with coma (C103y)
Other specified diabetes mellitus with multiple comps (C108y)
Unspecified diabetes mellitus with multiple complications (C108z)
Other specified diabetes mellitus with other spec comps (C10yy)
[X]Other specified diabetes mellitus (Cyu20)
[X]Unspecified diabetes mellitus with renal complications (Cyu23)
[X]Pre-existing diabetes mellitus, unspecified (Lyu29)
Insulin treated Type 2 diabetes mellitus (X40J6)
[illegible]

Diabetes-deafness syndrome maternally transmitted (X40JZ)
Diabetes mellitus, juvenile type, no mention of complication (XE10E)
Diabetes mellitus, adult onset, no mention of complication (XE10F)
Diabetes with other complications (XE12M)
Diabetes mellitus with gangrene (XM1Qx)
Diabetes mellitus due to insulin receptor antibodies (XSETp)
Maternally inherited diabetes mellitus (XaOPt)
Read Codes and Children:
Diabetes mellitus with ophthalmic manifestation (C105.)
Diabetes mellitus with other specified manifestation (C10y.)
Diabetes mellitus with unspecified complication (C10z.)
Neonatal diabetes mellitus (Q441.)
Type I diabetes mellitus (X40J4)
Type II diabetes mellitus (X40J5)
Malnutrition-related diabetes mellitus (X40J7)
Secondary diabetes mellitus (X40JA)
Genetic syndromes of diabetes mellitus (X40JG)
Abnormal metabolic state in diabetes mellitus (X40Ja)
Diabetes mellitus with renal manifestation (XE10G)
Diabetes mellitus with neurological manifestation (XE10H)
Diabetes mellitus with peripheral circulatory disorder (XE10I)
Unstable diabetes (XM1Xk)
Where patient is registered at General Practice

OR IN → **Over 75**
ASPIRE Study / 13 zjoins
Current age > 75 years
Where patient is registered at General Practice

AND IN → **Atrial Fibrilation diagnosis**
ASPIRE Study / 13 zjoins
Has a Read code in the DRAFIB1 (Atrial fibrillation codes) QOF cluster
Show read codes in cluster DRAFIB1.
- Selecting only the most recent matching code
- Without a more recent Read code in the DRAFIB2 (Atrial fibrillation resolved codes) QOF cluster

Where patient is registered at General Practice

OR IN → **3. CHAD2 Score = 3 (with AF)**
ASPIRE Study / 13 zjoins

IN → **3I. All CHAD2 = 3 Combinations**
ASPIRE Study / 13 zjoins

IN → **3a. CVA/TIA and HF not in (HYP or DIAB or >75)**
ASPIRE Study / 13 zjoins

IN → **Double CVA/TIA and HF**
ASPIRE Study / 13 zjoins
Where patient is registered at General Practice

IN → **CVA and/or TIA**
ASPIRE Study / 13 zjoins
Where patient is registered at General Practice

IN → **TIA diagnosis**
ASPIRE Study / 13 zjoins
Has a Read code of Transient ischaemic attack (XE0VK) or one of its children
Where patient is registered at General Practice

OR IN → **CVA diagnosis**
ASPIRE Study / 13 zjoins

Has a Read code of Cerebrovascular accident (X00D1) or one of its children
Where patient is registered at General Practice

AND IN

Heart Failure diagnosis
ASPIRE Study / 13 zjoins

Has a Read code in...Exact Read Codes:
Heart failure (G58..)
Decompensated cardiac failure (G5802)
Compensated cardiac failure (G5803)
Acute heart failure (G582.)
Heart failure as a complication of care (X202k)
Right ventricular failure (X202l)
Heart failure NOS (XE0V9)
Refractory heart failure (XaEgY)
New York Heart Association classification - class I (XaJ9G)
New York Heart Association classification - class II (XaJ9H)
New York Heart Association classification - class III (XaJ9I)
New York Heart Association classification - class IV (XaJ9J)
Read Codes and Children:
Biventricular failure (XE0V8)
Left ventricular failure (XE2QG)
Where patient is registered at General Practice

NOT IN

Triple HYP or DIAB or >75
ASPIRE Study / 13 zjoins

Where patient is registered at General Practice

IN

Hypertension diagnosis
ASPIRE Study / 13 zjoins

Has a Read code in...Exact Read Codes:
Systolic hypertension (G202.)
Secondary hypertension (G24..)
Hypertension secondary to endocrine disorders (G244.)
Secondary hypertension NOS (G24z.)
Hypertension secondary to drug (G24z1)
[X]Other secondary hypertension (Gyu20)
[X]Hypertension secondary to other renal disorders (Gyu21)
Pre-exist 2ndry hypertens comp preg childbth and puerprum (L1282)
Hypertension (XE0Ub)
Diastolic hypertension (XSDSb)
Labile hypertension (Xa0Cs)
Malignant hypertension (Xa3fQ)
Read Codes and Children:
Hypertensive disease (G2...)
Malignant secondary hypertension (G240.)
Secondary benign hypertension (G241.)
Essential hypertension (XE0Uc)
Renovascular hypertension (Xa0kX)
Where patient is registered at General Practice

OR IN

Diabetes diagnosis
ASPIRE Study / 13 zjoins

Has a Read code in...Exact Read Codes:
[Brittle] and/or [labile diabetes] (66AJ1)
Diabetes mellitus (C10..)
Diabetes mellitus with no mention of complication (C100.)
Diabetes mellitus NOS with no mention of complication (C100z)
Other specified diabetes mellitus with coma (C103y)
Other specified diabetes mellitus with multiple comps (C108y)
Unspecified diabetes mellitus with multiple complications (C108z)
Other specified diabetes mellitus with other spec comps (C10yy)
[X]Other specified diabetes mellitus (Cyu20)
[X]Unspecified diabetes mellitus with renal complications (Cyu23)
[X]Pre-existing diabetes mellitus, unspecified (Lyu29)
Insulin treated Type 2 diabetes mellitus (X40J6)
Diabetes-deafness syndrome maternally transmitted (X40JZ)
Diabetes mellitus, juvenile type, no mention of complication (XE10E)

Diabetes mellitus, adult onset, no mention of complication (XE10F)
Diabetes with other complications (XE12M)
Diabetes mellitus with gangrene (XM1Qx)
Diabetes mellitus due to insulin receptor antibodies (XSETp)
Maternally inherited diabetes mellitus (XaOPt)
Read Codes and Children:
Diabetes mellitus with ophthalmic manifestation (C105.)
Diabetes mellitus with other specified manifestation (C10y.)
Diabetes mellitus with unspecified complication (C10z.)
Neonatal diabetes mellitus (Q441.)
Type I diabetes mellitus (X40J4)
Type II diabetes mellitus (X40J5)
Malnutrition-related diabetes mellitus (X40J7)
Secondary diabetes mellitus (X40JA)
Genetic syndromes of diabetes mellitus (X40JG)
Abnormal metabolic state in diabetes mellitus (X40Ja)
Diabetes mellitus with renal manifestation (XE10G)
Diabetes mellitus with neurological manifestation (XE10H)
Diabetes mellitus with peripheral circulatory disorder (XE10I)
Unstable diabetes (XM1Xk)
Where patient is registered at General Practice

OR IN

Over 75
ASPIRE Study / 13 zjoins

Current age > 75 years
Where patient is registered at General Practice

OR IN

3b. CVA/TIA and HYP not in (HF or DIAB or >75)
ASPIRE Study / 13 zjoins

IN

Double CVA/TIA and HYP
ASPIRE Study / 13 zjoins

Where patient is registered at General Practice

IN

Hypertension diagnosis
ASPIRE Study / 13 zjoins

Has a Read code in...Exact Read Codes:
Systolic hypertension (G202.)
Secondary hypertension (G24..)
Hypertension secondary to endocrine disorders (G244.)
Secondary hypertension NOS (G24z.)
Hypertension secondary to drug (G24z1)
[X]Other secondary hypertension (Gyu20)
[X]Hypertension secondary to other renal disorders (Gyu21)
Pre-exist 2ndry hypertens comp preg childbth and puerprum (L1282)
Hypertension (XE0Ub)
Diastolic hypertension (XSDSb)
Labile hypertension (Xa0Cs)
Malignant hypertension (Xa3fQ)
Read Codes and Children:
Hypertensive disease (G2...)
Malignant secondary hypertension (G240.)
Secondary benign hypertension (G241.)
Essential hypertension (XE0Uc)
Renovascular hypertension (Xa0kX)
Where patient is registered at General Practice

AND IN

CVA and/or TIA
ASPIRE Study / 13 zjoins

Where patient is registered at General Practice

IN

TIA diagnosis
ASPIRE Study / 13 zjoins

Has a Read code of Transient ischaemic attack (XE0VK) or one of its children
Where patient is registered at General Practice

OR IN

CVA diagnosis
ASPIRE Study / 13 zjoins

Has a Read code of Cerebrovascular accident (X00D1) or one of its children

Where patient is registered at General Practice

NOT IN

Triple HF or DIAB or >75
ASPIRE Study / 13 zjoins

Where patient is registered at General Practice

IN

Diabetes diagnosis
ASPIRE Study / 13 zjoins

Has a Read code in...Exact Read Codes:
[Brittle] and/or [labile diabetes] (66AJ1)
Diabetes mellitus (C10..)
Diabetes mellitus with no mention of complication (C100.)
Diabetes mellitus NOS with no mention of complication (C100z)
Other specified diabetes mellitus with coma (C103y)
Other specified diabetes mellitus with multiple comps (C108y)
Unspecified diabetes mellitus with multiple complications (C108z)
Other specified diabetes mellitus with other spec comps (C10yy)
[X]Other specified diabetes mellitus (Cyu20)
[X]Unspecified diabetes mellitus with renal complications (Cyu23)
[X]Pre-existing diabetes mellitus, unspecified (Lyu29)
Insulin treated Type 2 diabetes mellitus (X40J6)
Diabetes-deafness syndrome maternally transmitted (X40JZ)
Diabetes mellitus, juvenile type, no mention of complication (XE10E)
Diabetes mellitus, adult onset, no mention of complication (XE10F)
Diabetes with other complications (XE12M)
Diabetes mellitus with gangrene (XM1Qx)
Diabetes mellitus due to insulin receptor antibodies (XSETp)
Maternally inherited diabetes mellitus (XaOPt)
Read Codes and Children:
Diabetes mellitus with ophthalmic manifestation (C105.)
Diabetes mellitus with other specified manifestation (C10y.)
Diabetes mellitus with unspecified complication (C10z.)
Neonatal diabetes mellitus (Q441.)
Type I diabetes mellitus (X40J4)
Type II diabetes mellitus (X40J5)
Malnutrition-related diabetes mellitus (X40J7)
Secondary diabetes mellitus (X40JA)
Genetic syndromes of diabetes mellitus (X40JG)
Abnormal metabolic state in diabetes mellitus (X40Ja)
Diabetes mellitus with renal manifestation (XE10G)
Diabetes mellitus with neurological manifestation (XE10H)
Diabetes mellitus with peripheral circulatory disorder (XE10I)
Unstable diabetes (XM1Xk)

Where patient is registered at General Practice

OR IN

Over 75
ASPIRE Study / 13 zjoins

Current age > 75 years

Where patient is registered at General Practice

OR IN

Heart Failure diagnosis
ASPIRE Study / 13 zjoins

Has a Read code in...Exact Read Codes:
Heart failure (G58..)
Decompensated cardiac failure (G5802)
Compensated cardiac failure (G5803)
Acute heart failure (G582.)
Heart failure as a complication of care (X202k)
Right ventricular failure (X202l)

Heart failure NOS (XE0V9)
Refractory heart failure (XaEgY)
New York Heart Association classification - class I (XaJ9G)
New York Heart Association classification - class II (XaJ9H)
New York Heart Association classification - class III (XaJ9I)
New York Heart Association classification - class IV (XaJ9J)
Read Codes and Children:
Biventricular failure (XE0V8)
Left ventricular failure (XE2QG)
Where patient is registered at General Practice

OR IN

3c. CVA/TIA and DIAB not in (HF or HYP or >75)
ASPIRE Study / 13 zjoins

IN

Double CVA/TIA and DIAB
ASPIRE Study / 13 zjoins

Where patient is registered at General Practice

IN

Diabetes diagnosis
ASPIRE Study / 13 zjoins

Has a Read code in...Exact Read Codes:
[Brittle] and/or [labile diabetes] (66AJ1)
Diabetes mellitus (C10..)
Diabetes mellitus with no mention of complication (C100.)
Diabetes mellitus NOS with no mention of complication (C100z)
Other specified diabetes mellitus with coma (C103y)
Other specified diabetes mellitus with multiple comps (C108y)
Unspecified diabetes mellitus with multiple complications (C108z)
Other specified diabetes mellitus with other spec comps (C10yy)
[X]Other specified diabetes mellitus (Cyu20)
[X]Unspecified diabetes mellitus with renal complications (Cyu23)
[X]Pre-existing diabetes mellitus, unspecified (Lyu29)
Insulin treated Type 2 diabetes mellitus (X40J6)
Diabetes-deafness syndrome maternally transmitted (X40JZ)
Diabetes mellitus, juvenile type, no mention of complication (XE10E)
Diabetes mellitus, adult onset, no mention of complication (XE10F)
Diabetes with other complications (XE12M)
Diabetes mellitus with gangrene (XM1Qx)
Diabetes mellitus due to insulin receptor antibodies (XSETp)
Maternally inherited diabetes mellitus (XaOPt)
Read Codes and Children:
Diabetes mellitus with ophthalmic manifestation (C105.)
Diabetes mellitus with other specified manifestation (C10y.)
Diabetes mellitus with unspecified complication (C10z.)
Neonatal diabetes mellitus (Q441.)
Type I diabetes mellitus (X40J4)
Type II diabetes mellitus (X40J5)
Malnutrition-related diabetes mellitus (X40J7)
Secondary diabetes mellitus (X40JA)
Genetic syndromes of diabetes mellitus (X40JG)
Abnormal metabolic state in diabetes mellitus (X40Ja)
Diabetes mellitus with renal manifestation (XE10G)
Diabetes mellitus with neurological manifestation (XE10H)
Diabetes mellitus with peripheral circulatory disorder (XE10I)
Unstable diabetes (XM1Xk)
Where patient is registered at General Practice

AND IN

CVA and/or TIA
ASPIRE Study / 13 zjoins

Where patient is registered at General Practice

IN

TIA diagnosis
ASPIRE Study / 13 zjoins

Has a Read code of Transient ischaemic attack (XE0VK) or one of its children
Where patient is registered at General Practice

OR IN

CVA diagnosis
ASPIRE Study / 13 zjoins

Has a Read code of Cerebrovascular accident (X00D1) or one of its children
Where patient is registered at General Practice

NOT IN

Triple HF or HYP or >75
ASPIRE Study / 13 zjoins

Where patient is registered at General Practice

IN

Heart Failure diagnosis
ASPIRE Study / 13 zjoins

Has a Read code in...Exact Read Codes:
Heart failure (G58..)
Decompensated cardiac failure (G5802)
Compensated cardiac failure (G5803)
Acute heart failure (G582.)
Heart failure as a complication of care (X202k)
Right ventricular failure (X202l)
Heart failure NOS (XE0V9)
Refractory heart failure (XaEgY)
New York Heart Association classification - class I (XaJ9G)
New York Heart Association classification - class II (XaJ9H)
New York Heart Association classification - class III (XaJ9I)
New York Heart Association classification - class IV (XaJ9J)
Read Codes and Children:
Biventricular failure (XE0V8)
Left ventricular failure (XE2QG)
Where patient is registered at General Practice

OR IN

Over 75
ASPIRE Study / 13 zjoins

Current age > 75 years
Where patient is registered at General Practice

OR IN

Hypertension diagnosis
ASPIRE Study / 13 zjoins

Has a Read code in...Exact Read Codes:
Systolic hypertension (G202.)
Secondary hypertension (G24..)
Hypertension secondary to endocrine disorders (G244.)
Secondary hypertension NOS (G24z.)
Hypertension secondary to drug (G24z1)
[X]Other secondary hypertension (Gyu20)
[X]Hypertension secondary to other renal disorders (Gyu21)
Pre-exist 2ndry hypertens comp preg childbth and puerprum (L1282)
Hypertension (XE0Ub)
Diastolic hypertension (XSDSb)
Labile hypertension (Xa0Cs)
Malignant hypertension (Xa3fQ)
Read Codes and Children:
Hypertensive disease (G2...)
Malignant secondary hypertension (G240.)
Secondary benign hypertension (G241.)
Essential hypertension (XE0Uc)
Renovascular hypertension (Xa0kX)
Where patient is registered at General Practice

OR IN

3d. CVA/TIA and >75 not in (HF or HYP or DIAB)
ASPIRE Study / 13 zjoins

IN

Double CVA/TIA and >75
ASPIRE Study / 13 zjoins

Where patient is registered at General Practice

IN → **Over 75**
ASPIRE Study / 13 zjoins
- Current age > 75 years
- Where patient is registered at General Practice

AND IN → **CVA and/or TIA**
ASPIRE Study / 13 zjoins
- Where patient is registered at General Practice

IN → **TIA diagnosis**
ASPIRE Study / 13 zjoins
- Has a Read code of Transient ischaemic attack (XE0VK) or one of its children
- Where patient is registered at General Practice

OR IN → **CVA diagnosis**
ASPIRE Study / 13 zjoins
- Has a Read code of Cerebrovascular accident (X00D1) or one of its children
- Where patient is registered at General Practice

NOT IN → **Triple HF or HYP or DIAB**
ASPIRE Study / 13 zjoins
- Where patient is registered at General Practice

IN → **Heart Failure diagnosis**
ASPIRE Study / 13 zjoins
- Has a Read code in...Exact Read Codes:
  Heart failure (G58..)
  Decompensated cardiac failure (G5802)
  Compensated cardiac failure (G5803)
  Acute heart failure (G582.)
  Heart failure as a complication of care (X202k)
  Right ventricular failure (X202l)
  Heart failure NOS (XE0V9)
  Refractory heart failure (XaEgY)
  New York Heart Association classification - class I (XaJ9G)
  New York Heart Association classification - class II (XaJ9H)
  New York Heart Association classification - class III (XaJ9I)
  New York Heart Association classification - class IV (XaJ9J)
  Read Codes and Children:
  Biventricular failure (XE0V8)
  Left ventricular failure (XE2QG)
- Where patient is registered at General Practice

OR IN → **Hypertension diagnosis**
ASPIRE Study / 13 zjoins
- Has a Read code in...Exact Read Codes:
  Systolic hypertension (G202.)
  Secondary hypertension (G24..)
  Hypertension secondary to endocrine disorders (G244.)
  Secondary hypertension NOS (G24z.)
  Hypertension secondary to drug (G24z1)
  [X]Other secondary hypertension (Gyu20)
  [X]Hypertension secondary to other renal disorders (Gyu21)
  Pre-exist 2ndry hypertens comp preg childbth and puerprum (L1282)
  Hypertension (XE0Ub)
  Diastolic hypertension (XSDSb)
  Labile hypertension (Xa0Cs)
  Malignant hypertension (Xa3fQ)
  Read Codes and Children:
  Hypertensive disease (G2...)
  Malignant secondary hypertension (G240.)
  Secondary benign hypertension (G241.)
  Essential hypertension (XE0Uc)
  Renovascular hypertension (Xa0kX)
- Where patient is registered at General Practice

OR IN → **Diabetes diagnosis**
ASPIRE Study / 13 zjoins
- Has a Read code in...Exact Read Codes:

[Brittle] and/or [labile diabetes] (66AJ1)
Diabetes mellitus (C10..)
Diabetes mellitus with no mention of complication (C100.)
Diabetes mellitus NOS with no mention of complication (C100z)
Other specified diabetes mellitus with coma (C103y)
Other specified diabetes mellitus with multiple comps (C108y)
Unspecified diabetes mellitus with multiple complications (C108z)
Other specified diabetes mellitus with other spec comps (C10yy)
[X]Other specified diabetes mellitus (Cyu20)
[X]Unspecified diabetes mellitus with renal complications (Cyu23)
[X]Pre-existing diabetes mellitus, unspecified (Lyu29)
Insulin treated Type 2 diabetes mellitus (X40J6)
Diabetes-deafness syndrome maternally transmitted (X40JZ)
Diabetes mellitus, juvenile type, no mention of complication (XE10E)
Diabetes mellitus, adult onset, no mention of complication (XE10F)
Diabetes with other complications (XE12M)
Diabetes mellitus with gangrene (XM1Qx)
Diabetes mellitus due to insulin receptor antibodies (XSETp)
Maternally inherited diabetes mellitus (XaOPt)
Read Codes and Children:
Diabetes mellitus with ophthalmic manifestation (C105.)
Diabetes mellitus with other specified manifestation (C10y.)
Diabetes mellitus with unspecified complication (C10z.)
Neonatal diabetes mellitus (Q441.)
Type I diabetes mellitus (X40J4)
Type II diabetes mellitus (X40J5)
Malnutrition-related diabetes mellitus (X40J7)
Secondary diabetes mellitus (X40JA)
Genetic syndromes of diabetes mellitus (X40JG)
Abnormal metabolic state in diabetes mellitus (X40Ja)
Diabetes mellitus with renal manifestation (XE10G)
Diabetes mellitus with neurological manifestation (XE10H)
Diabetes mellitus with peripheral circulatory disorder (XE10I)
Unstable diabetes (XM1Xk)

Where patient is registered at General Practice

OR IN

3e. HF and HYP and DIAB not in (CVA/TIA or >75)
ASPIRE Study / 13 zjoins

IN

Triple HF and HYP and DIAB
ASPIRE Study / 13 zjoins

Where patient is registered at General Practice

IN

Heart Failure diagnosis
ASPIRE Study / 13 zjoins

Has a Read code in...Exact Read Codes:
Heart failure (G58..)
Decompensated cardiac failure (G5802)
Compensated cardiac failure (G5803)
Acute heart failure (G582.)
Heart failure as a complication of care (X202k)
Right ventricular failure (X202l)
Heart failure NOS (XE0V9)
Refractory heart failure (XaEgY)
New York Heart Association classification - class I (XaJ9G)
New York Heart Association classification - class II (XaJ9H)
New York Heart Association classification - class III (XaJ9I)
New York Heart Association classification - class IV (XaJ9J)
Read Codes and Children:
Biventricular failure (XE0V8)
Left ventricular failure (XE2QG)

Where patient is registered at General

Where patient is registered at General Practice

AND IN

Hypertension diagnosis
ASPIRE Study / 13 zjoins

Has a Read code in...Exact Read Codes:
Systolic hypertension (G202.)
Secondary hypertension (G24..)
Hypertension secondary to endocrine disorders (G244.)
Secondary hypertension NOS (G24z.)
Hypertension secondary to drug (G24z1)
[X]Other secondary hypertension (Gyu20)
[X]Hypertension secondary to other renal disorders (Gyu21)
Pre-exist 2ndry hypertens comp preg childbth and puerprum (L1282)
Hypertension (XE0Ub)
Diastolic hypertension (XSDSb)
Labile hypertension (Xa0Cs)
Malignant hypertension (Xa3fQ)
Read Codes and Children:
Hypertensive disease (G2...)
Malignant secondary hypertension (G240.)
Secondary benign hypertension (G241.)
Essential hypertension (XE0Uc)
Renovascular hypertension (Xa0kX)

Where patient is registered at General Practice

AND IN

Diabetes diagnosis
ASPIRE Study / 13 zjoins

Has a Read code in...Exact Read Codes:
[Brittle] and/or [labile diabetes] (66AJ1)
Diabetes mellitus (C10..)
Diabetes mellitus with no mention of complication (C100.)
Diabetes mellitus NOS with no mention of complication (C100z)
Other specified diabetes mellitus with coma (C103y)
Other specified diabetes mellitus with multiple comps (C108y)
Unspecified diabetes mellitus with multiple complications (C108z)
Other specified diabetes mellitus with other spec comps (C10yy)
[X]Other specified diabetes mellitus (Cyu20)
[X]Unspecified diabetes mellitus with renal complications (Cyu23)
[X]Pre-existing diabetes mellitus, unspecified (Lyu29)
Insulin treated Type 2 diabetes mellitus (X40J6)
Diabetes-deafness syndrome maternally transmitted (X40JZ)
Diabetes mellitus, juvenile type, no mention of complication (XE10E)
Diabetes mellitus, adult onset, no mention of complication (XE10F)
Diabetes with other complications (XE12M)
Diabetes mellitus with gangrene (XM1Qx)
Diabetes mellitus due to insulin receptor antibodies (XSETp)
Maternally inherited diabetes mellitus (XaOPt)
Read Codes and Children:
Diabetes mellitus with ophthalmic manifestation (C105.)
Diabetes mellitus with other specified manifestation (C10y.)
Diabetes mellitus with unspecified complication (C10z.)
Neonatal diabetes mellitus (Q441.)
Type I diabetes mellitus (X40J4)
Type II diabetes mellitus (X40J5)
Malnutrition-related diabetes mellitus (X40J7)
Secondary diabetes mellitus (X40JA)
Genetic syndromes of diabetes mellitus (X40JG)
Abnormal metabolic state in diabetes mellitus (X40Ja)
Diabetes mellitus with renal manifestation (XE10G)
Diabetes mellitus with neurological manifestation (XE10H)
Diabetes mellitus with peripheral circulatory disorder (XE10I)
Unstable diabetes (XM1Xk)

Where patient is registered at General Practice

NOT IN

Double CVA/TIA or >75

ASPIRE Study / 13 zjoins
Where patient is registered at General Practice

IN → **Over 75**
ASPIRE Study / 13 zjoins
Current age > 75 years
Where patient is registered at General Practice

OR IN → **CVA and/or TIA**
ASPIRE Study / 13 zjoins
Where patient is registered at General Practice

IN → **TIA diagnosis**
ASPIRE Study / 13 zjoins
Has a Read code of Transient ischaemic attack (XE0VK) or one of its children
Where patient is registered at General Practice

OR IN → **CVA diagnosis**
ASPIRE Study / 13 zjoins
Has a Read code of Cerebrovascular accident (X00D1) or one of its children
Where patient is registered at General Practice

OR IN → **3f. HF and DIAB and >75 not in (CVA/TIA or HYP)**
ASPIRE Study / 13 zjoins

IN → **Triple HF and DIAB and >75**
ASPIRE Study / 13 zjoins
Where patient is registered at General Practice

IN → **Diabetes diagnosis**
ASPIRE Study / 13 zjoins
Has a Read code in...Exact Read Codes:
[Brittle] and/or [labile diabetes] (66AJ1)
Diabetes mellitus (C10..)
Diabetes mellitus with no mention of complication (C100.)
Diabetes mellitus NOS with no mention of complication (C100z)
Other specified diabetes mellitus with coma (C103y)
Other specified diabetes mellitus with multiple comps (C108y)
Unspecified diabetes mellitus with multiple complications (C108z)
Other specified diabetes mellitus with other spec comps (C10yy)
[X]Other specified diabetes mellitus (Cyu20)
[X]Unspecified diabetes mellitus with renal complications (Cyu23)
[X]Pre-existing diabetes mellitus, unspecified (Lyu29)
Insulin treated Type 2 diabetes mellitus (X40J6)
Diabetes-deafness syndrome maternally transmitted (X40JZ)
Diabetes mellitus, juvenile type, no mention of complication (XE10E)
Diabetes mellitus, adult onset, no mention of complication (XE10F)
Diabetes with other complications (XE12M)
Diabetes mellitus with gangrene (XM1Qx)
Diabetes mellitus due to insulin receptor antibodies (XSETp)
Maternally inherited diabetes mellitus (XaOPt)
Read Codes and Children:
Diabetes mellitus with ophthalmic manifestation (C105.)
Diabetes mellitus with other specified manifestation (C10y.)
Diabetes mellitus with unspecified complication (C10z.)
Neonatal diabetes mellitus (Q441.)
Type I diabetes mellitus (X40J4)
Type II diabetes mellitus (X40J5)
Malnutrition-related diabetes mellitus (X40J7)
Secondary diabetes mellitus (X40JA)
Genetic syndromes of diabetes mellitus

(X40JG)
Abnormal metabolic state in diabetes mellitus (X40Ja)
Diabetes mellitus with renal manifestation (XE10G)
Diabetes mellitus with neurological manifestation (XE10H)
Diabetes mellitus with peripheral circulatory disorder (XE10I)
Unstable diabetes (XM1Xk)
Where patient is registered at General Practice

AND IN

Over 75
ASPIRE Study / 13 zjoins
Current age > 75 years
Where patient is registered at General Practice

AND IN

Heart Failure diagnosis
ASPIRE Study / 13 zjoins
Has a Read code in...Exact Read Codes:
Heart failure (G58..)
Decompensated cardiac failure (G5802)
Compensated cardiac failure (G5803)
Acute heart failure (G582.)
Heart failure as a complication of care (X202k)
Right ventricular failure (X202l)
Heart failure NOS (XE0V9)
Refractory heart failure (XaEgY)
New York Heart Association classification - class I (XaJ9G)
New York Heart Association classification - class II (XaJ9H)
New York Heart Association classification - class III (XaJ9I)
New York Heart Association classification - class IV (XaJ9J)
Read Codes and Children:
Biventricular failure (XE0V8)
Left ventricular failure (XE2QG)
Where patient is registered at General Practice

NOT IN

Double CVA/TIA or HYP
ASPIRE Study / 13 zjoins
Where patient is registered at General Practice

IN

Hypertension diagnosis
ASPIRE Study / 13 zjoins
Has a Read code in...Exact Read Codes:
Systolic hypertension (G202.)
Secondary hypertension (G24..)
Hypertension secondary to endocrine disorders (G244.)
Secondary hypertension NOS (G24z.)
Hypertension secondary to drug (G24z1)
[X]Other secondary hypertension (Gyu20)
[X]Hypertension secondary to other renal disorders (Gyu21)
Pre-exist 2ndry hypertens comp preg childbth and puerprum (L1282)
Hypertension (XE0Ub)
Diastolic hypertension (XSDSb)
Labile hypertension (Xa0Cs)
Malignant hypertension (Xa3fQ)
Read Codes and Children:
Hypertensive disease (G2...)
Malignant secondary hypertension (G240.)
Secondary benign hypertension (G241.)
Essential hypertension (XE0Uc)
Renovascular hypertension (Xa0kX)
Where patient is registered at General Practice

OR IN

CVA and/or TIA
ASPIRE Study / 13 zjoins
Where patient is registered at General Practice

IN

TIA diagnosis
ASPIRE Study / 13 zjoins
Has a Read code of Transient ischaemic attack (XE0VK) or one of its children
Where patient is registered at General Practice

OR IN

CVA diagnosis
ASPIRE Study / 13 zjoins

Has a Read code of Cerebrovascular accident (X00D1) or one of its children

Where patient is registered at General Practice

OR IN

3g. HF and HYP and >75 not in (CVA/TIA or DIAB)
ASPIRE Study / 13 zjoins

IN

Triple HF and HYP and >75
ASPIRE Study / 13 zjoins

Where patient is registered at General Practice

IN

Heart Failure diagnosis
ASPIRE Study / 13 zjoins

Has a Read code in...Exact Read Codes:
Heart failure (G58..)
Decompensated cardiac failure (G5802)
Compensated cardiac failure (G5803)
Acute heart failure (G582.)
Heart failure as a complication of care (X202k)
Right ventricular failure (X202l)
Heart failure NOS (XE0V9)
Refractory heart failure (XaEgY)
New York Heart Association classification - class I (XaJ9G)
New York Heart Association classification - class II (XaJ9H)
New York Heart Association classification - class III (XaJ9I)
New York Heart Association classification - class IV (XaJ9J)
Read Codes and Children:
Biventricular failure (XE0V8)
Left ventricular failure (XE2QG)

Where patient is registered at General Practice

AND IN

Over 75
ASPIRE Study / 13 zjoins

Current age > 75 years

Where patient is registered at General Practice

AND IN

Hypertension diagnosis
ASPIRE Study / 13 zjoins

Has a Read code in...Exact Read Codes:
Systolic hypertension (G202.)
Secondary hypertension (G24..)
Hypertension secondary to endocrine disorders (G244.)
Secondary hypertension NOS (G24z.)
Hypertension secondary to drug (G24z1)
[X]Other secondary hypertension (Gyu20)
[X]Hypertension secondary to other renal disorders (Gyu21)
Pre-exist 2ndry hypertens comp preg childbth and puerprum (L1282)
Hypertension (XE0Ub)
Diastolic hypertension (XSDSb)
Labile hypertension (Xa0Cs)
Malignant hypertension (Xa3fQ)
Read Codes and Children:
Hypertensive disease (G2...)
Malignant secondary hypertension (G240.)
Secondary benign hypertension (G241.)
Essential hypertension (XE0Uc)
Renovascular hypertension (Xa0kX)

Where patient is registered at General Practice

NOT IN

Double CVA/TIA or DIAB
ASPIRE Study / 13 zjoins

Where patient is registered at General Practice

IN

Diabetes diagnosis
ASPIRE Study / 13 zjoins

Has a Read code in...Exact Read Codes:
[Brittle] and/or [labile diabetes] (66AJ1)
Diabetes mellitus (C10..)
Diabetes mellitus with no mention of complication (C100.)
[illegible]

Diabetes mellitus NOS with no mention of complication (C100z)
Other specified diabetes mellitus with coma (C103y)
Other specified diabetes mellitus with multiple comps (C108y)
Unspecified diabetes mellitus with multiple complications (C108z)
Other specified diabetes mellitus with other spec comps (C10yy)
[X]Other specified diabetes mellitus (Cyu20)
[X]Unspecified diabetes mellitus with renal complications (Cyu23)
[X]Pre-existing diabetes mellitus, unspecified (Lyu29)
Insulin treated Type 2 diabetes mellitus (X40J6)
Diabetes-deafness syndrome maternally transmitted (X40JZ)
Diabetes mellitus, juvenile type, no mention of complication (XE10E)
Diabetes mellitus, adult onset, no mention of complication (XE10F)
Diabetes with other complications (XE12M)
Diabetes mellitus with gangrene (XM1Qx)
Diabetes mellitus due to insulin receptor antibodies (XSETp)
Maternally inherited diabetes mellitus (XaOPt)
Read Codes and Children:
Diabetes mellitus with ophthalmic manifestation (C105.)
Diabetes mellitus with other specified manifestation (C10y.)
Diabetes mellitus with unspecified complication (C10z.)
Neonatal diabetes mellitus (Q441.)
Type I diabetes mellitus (X40J4)
Type II diabetes mellitus (X40J5)
Malnutrition-related diabetes mellitus (X40J7)
Secondary diabetes mellitus (X40JA)
Genetic syndromes of diabetes mellitus (X40JG)
Abnormal metabolic state in diabetes mellitus (X40Ja)
Diabetes mellitus with renal manifestation (XE10G)
Diabetes mellitus with neurological manifestation (XE10H)
Diabetes mellitus with peripheral circulatory disorder (XE10I)
Unstable diabetes (XM1Xk)

Where patient is registered at General Practice

OR IN

CVA and/or TIA
ASPIRE Study / 13 zjoins

Where patient is registered at General Practice

IN

TIA diagnosis
ASPIRE Study / 13 zjoins

Has a Read code of Transient ischaemic attack (XE0VK) or one of its children
Where patient is registered at General Practice

OR IN

CVA diagnosis
ASPIRE Study / 13 zjoins

Has a Read code of Cerebrovascular accident (X00D1) or one of its children
Where patient is registered at General Practice

OR IN

3h. HYP and DIAB and >75 not in (CVA/TIA or HF)
ASPIRE Study / 13 zjoins

IN

Triple HYP and DIAB and >75
ASPIRE Study / 13 zjoins

Where patient is registered at General Practice

IN

Hypertension diagnosis
ASPIRE Study / 13 zjoins

Has a Read code in...Exact Read Codes:
Systolic hypertension (G202.)
Secondary hypertension (G24..)
Hypertension secondary to endocrine

disorders (G244.)
Secondary hypertension NOS (G24z.)
Hypertension secondary to drug (G24z1)
[X]Other secondary hypertension (Gyu20)
[X]Hypertension secondary to other renal disorders (Gyu21)
Pre-exist 2ndry hypertens comp preg childbth and puerprum (L1282)
Hypertension (XE0Ub)
Diastolic hypertension (XSDSb)
Labile hypertension (Xa0Cs)
Malignant hypertension (Xa3fQ)
Read Codes and Children:
Hypertensive disease (G2...)
Malignant secondary hypertension (G240.)
Secondary benign hypertension (G241.)
Essential hypertension (XE0Uc)
Renovascular hypertension (Xa0kX)
Where patient is registered at General Practice

AND IN

Diabetes diagnosis
ASPIRE Study / 13 zjoins

Has a Read code in...Exact Read Codes:
[Brittle] and/or [labile diabetes] (66AJ1)
Diabetes mellitus (C10..)
Diabetes mellitus with no mention of complication (C100.)
Diabetes mellitus NOS with no mention of complication (C100z)
Other specified diabetes mellitus with coma (C103y)
Other specified diabetes mellitus with multiple comps (C108y)
Unspecified diabetes mellitus with multiple complications (C108z)
Other specified diabetes mellitus with other spec comps (C10yy)
[X]Other specified diabetes mellitus (Cyu20)
[X]Unspecified diabetes mellitus with renal complications (Cyu23)
[X]Pre-existing diabetes mellitus, unspecified (Lyu29)
Insulin treated Type 2 diabetes mellitus (X40J6)
Diabetes-deafness syndrome maternally transmitted (X40JZ)
Diabetes mellitus, juvenile type, no mention of complication (XE10E)
Diabetes mellitus, adult onset, no mention of complication (XE10F)
Diabetes with other complications (XE12M)
Diabetes mellitus with gangrene (XM1Qx)
Diabetes mellitus due to insulin receptor antibodies (XSETp)
Maternally inherited diabetes mellitus (XaOPt)
Read Codes and Children:
Diabetes mellitus with ophthalmic manifestation (C105.)
Diabetes mellitus with other specified manifestation (C10y.)
Diabetes mellitus with unspecified complication (C10z.)
Neonatal diabetes mellitus (Q441.)
Type I diabetes mellitus (X40J4)
Type II diabetes mellitus (X40J5)
Malnutrition-related diabetes mellitus (X40J7)
Secondary diabetes mellitus (X40JA)
Genetic syndromes of diabetes mellitus (X40JG)
Abnormal metabolic state in diabetes mellitus (X40Ja)
Diabetes mellitus with renal manifestation (XE10G)
Diabetes mellitus with neurological manifestation (XE10H)
Diabetes mellitus with peripheral circulatory disorder (XE10I)
Unstable diabetes (XM1Xk)
Where patient is registered at General Practice

AND IN

Over 75
ASPIRE Study / 13 zjoins

Current age > 75 years
Where patient is registered at General Practice

NOT IN

Double CVA/TIA or HF
ASPIRE Study / 13 zjoins

Where patient is registered at General Practice

IN

CVA and/or TIA
ASPIRE Study / 13 zjoins

Where patient is registered at General Practice

IN

TIA diagnosis
ASPIRE Study / 13 zjoins

Has a Read code of Transient ischaemic attack (XE0VK) or one of its children

Where patient is registered at General Practice

OR IN

CVA diagnosis
ASPIRE Study / 13 zjoins

Has a Read code of Cerebrovascular accident (X00D1) or one of its children

Where patient is registered at General Practice

OR IN

Heart Failure diagnosis
ASPIRE Study / 13 zjoins

Has a Read code in...Exact Read Codes:
Heart failure (G58..)
Decompensated cardiac failure (G5802)
Compensated cardiac failure (G5803)
Acute heart failure (G582.)
Heart failure as a complication of care (X202k)
Right ventricular failure (X202l)
Heart failure NOS (XE0V9)
Refractory heart failure (XaEgY)
New York Heart Association classification - class I (XaJ9G)
New York Heart Association classification - class II (XaJ9H)
New York Heart Association classification - class III (XaJ9I)
New York Heart Association classification - class IV (XaJ9J)
Read Codes and Children:
Biventricular failure (XE0V8)
Left ventricular failure (XE2QG)

Where patient is registered at General Practice

AND IN

Atrial Fibrilation diagnosis
ASPIRE Study / 13 zjoins

Has a Read code in the DRAFIB1 (Atrial fibrillation codes) QOF cluster
Show read codes in cluster DRAFIB1.

- Selecting only the most recent matching code
- Without a more recent Read code in the DRAFIB2 (Atrial fibrillation resolved codes) QOF cluster

Where patient is registered at General Practice

OR IN

4. CHAD2 Score = 4 (with AF)
ASPIRE Study / 13 zjoins

IN

4h. All CHAD2 = 4 Combinations
ASPIRE Study / 13 zjoins

IN

4a. CVA/TIA and HF and HYP not in (DIAB or >75)
ASPIRE Study / 13 zjoins

IN

Triple HF and HYP and CVA/TIA
ASPIRE Study / 13 zjoins

Where patient is registered at General Practice

IN

Heart Failure diagnosis
ASPIRE Study / 13 zjoins

Has a Read code in...Exact Read Codes:
Heart failure (G58..)
Decompensated cardiac failure (G5802)
Compensated cardiac failure (G5803)
Acute heart failure (G582.)
Heart failure as a complication of care (X202k)
Right ventricular failure (X202l)

Right ventricular failure ([illegible])
Heart failure NOS (XE0V9)
Refractory heart failure (XaEgY)
New York Heart Association classification - class I (XaJ9G)
New York Heart Association classification - class II (XaJ9H)
New York Heart Association classification - class III (XaJ9I)
New York Heart Association classification - class IV (XaJ9J)
Read Codes and Children:
Biventricular failure (XE0V8)
Left ventricular failure (XE2QG)

Where patient is registered at General Practice

AND IN

CVA and/or TIA
ASPIRE Study / 13 zjoins

Where patient is registered at General Practice

IN

TIA diagnosis
ASPIRE Study / 13 zjoins

Has a Read code of Transient ischaemic attack (XE0VK) or one of its children
Where patient is registered at General Practice

OR IN

CVA diagnosis
ASPIRE Study / 13 zjoins

Has a Read code of Cerebrovascular accident (X00D1) or one of its children
Where patient is registered at General Practice

AND IN

Hypertension diagnosis
ASPIRE Study / 13 zjoins

Has a Read code in...Exact Read Codes:
Systolic hypertension (G202.)
Secondary hypertension (G24..)
Hypertension secondary to endocrine disorders (G244.)
Secondary hypertension NOS (G24z.)
Hypertension secondary to drug (G24z1)
[X]Other secondary hypertension (Gyu20)
[X]Hypertension secondary to other renal disorders (Gyu21)
Pre-exist 2ndry hypertens comp preg childbth and puerprum (L1282)
Hypertension (XE0Ub)
Diastolic hypertension (XSDSb)
Labile hypertension (Xa0Cs)
Malignant hypertension (Xa3fQ)
Read Codes and Children:
Hypertensive disease (G2...)
Malignant secondary hypertension (G240.)
Secondary benign hypertension (G241.)
Essential hypertension (XE0Uc)
Renovascular hypertension (Xa0kX)

Where patient is registered at General Practice

NOT IN

Double DIAB or >75
ASPIRE Study / 13 zjoins

Where patient is registered at General Practice

IN

Over 75
ASPIRE Study / 13 zjoins

Current age > 75 years
Where patient is registered at General Practice

OR IN

Diabetes diagnosis
ASPIRE Study / 13 zjoins

Has a Read code in...Exact Read Codes:
[Brittle] and/or [labile diabetes] (66AJ1)
Diabetes mellitus (C10..)
Diabetes mellitus with no mention of complication (C100.)
Diabetes mellitus NOS with no mention of complication (C100z)
Other specified diabetes mellitus with coma (C103y)
Other specified diabetes mellitus with multiple comps (C108y)
Unspecified diabetes mellitus with multiple complications (C108z)

[illegible]
Other specified diabetes mellitus with other spec comps (C10yy)
[X]Other specified diabetes mellitus (Cyu20)
[X]Unspecified diabetes mellitus with renal complications (Cyu23)
[X]Pre-existing diabetes mellitus, unspecified (Lyu29)
Insulin treated Type 2 diabetes mellitus (X40J6)
Diabetes-deafness syndrome maternally transmitted (X40JZ)
Diabetes mellitus, juvenile type, no mention of complication (XE10E)
Diabetes mellitus, adult onset, no mention of complication (XE10F)
Diabetes with other complications (XE12M)
Diabetes mellitus with gangrene (XM1Qx)
Diabetes mellitus due to insulin receptor antibodies (XSETp)
Maternally inherited diabetes mellitus (XaOPt)
Read Codes and Children:
Diabetes mellitus with ophthalmic manifestation (C105.)
Diabetes mellitus with other specified manifestation (C10y.)
Diabetes mellitus with unspecified complication (C10z.)
Neonatal diabetes mellitus (Q441.)
Type I diabetes mellitus (X40J4)
Type II diabetes mellitus (X40J5)
Malnutrition-related diabetes mellitus (X40J7)
Secondary diabetes mellitus (X40JA)
Genetic syndromes of diabetes mellitus (X40JG)
Abnormal metabolic state in diabetes mellitus (X40Ja)
Diabetes mellitus with renal manifestation (XE10G)
Diabetes mellitus with neurological manifestation (XE10H)
Diabetes mellitus with peripheral circulatory disorder (XE10I)
Unstable diabetes (XM1Xk)

Where patient is registered at General Practice

OR IN

4b. CVA/TIA and HF and DIAB not in (HYP or >75)
ASPIRE Study / 13 zjoins

IN

Triple HF and DIAB and CVA/TIA
ASPIRE Study / 13 zjoins

Where patient is registered at General Practice

IN

Heart Failure diagnosis
ASPIRE Study / 13 zjoins

Has a Read code in...Exact Read Codes:
Heart failure (G58..)
Decompensated cardiac failure (G5802)
Compensated cardiac failure (G5803)
Acute heart failure (G582.)
Heart failure as a complication of care (X202k)
Right ventricular failure (X202l)
Heart failure NOS (XE0V9)
Refractory heart failure (XaEgY)
New York Heart Association classification - class I (XaJ9G)
New York Heart Association classification - class II (XaJ9H)
New York Heart Association classification - class III (XaJ9I)
New York Heart Association classification - class IV (XaJ9J)
Read Codes and Children:
Biventricular failure (XE0V8)
Left ventricular failure (XE2QG)

Where patient is registered at General Practice

AND IN

CVA and/or TIA
ASPIRE Study / 13 zjoins

Where patient is registered at General Practice

IN

TIA diagnosis
ASPIRE Study / 13 zjoins

Has a Read code of Transient ischaemic [illegible]

Has a Read code of Transient ischaemic attack (XE0VK) or one of its children
Where patient is registered at General Practice

OR IN

CVA diagnosis
ASPIRE Study / 13 zjoins

Has a Read code of Cerebrovascular accident (X00D1) or one of its children
Where patient is registered at General Practice

AND IN

Diabetes diagnosis
ASPIRE Study / 13 zjoins

Has a Read code in...Exact Read Codes:
[Brittle] and/or [labile diabetes] (66AJ1)
Diabetes mellitus (C10..)
Diabetes mellitus with no mention of complication (C100.)
Diabetes mellitus NOS with no mention of complication (C100z)
Other specified diabetes mellitus with coma (C103y)
Other specified diabetes mellitus with multiple comps (C108y)
Unspecified diabetes mellitus with multiple complications (C108z)
Other specified diabetes mellitus with other spec comps (C10yy)
[X]Other specified diabetes mellitus (Cyu20)
[X]Unspecified diabetes mellitus with renal complications (Cyu23)
[X]Pre-existing diabetes mellitus, unspecified (Lyu29)
Insulin treated Type 2 diabetes mellitus (X40J6)
Diabetes-deafness syndrome maternally transmitted (X40JZ)
Diabetes mellitus, juvenile type, no mention of complication (XE10E)
Diabetes mellitus, adult onset, no mention of complication (XE10F)
Diabetes with other complications (XE12M)
Diabetes mellitus with gangrene (XM1Qx)
Diabetes mellitus due to insulin receptor antibodies (XSETp)
Maternally inherited diabetes mellitus (XaOPt)
Read Codes and Children:
Diabetes mellitus with ophthalmic manifestation (C105.)
Diabetes mellitus with other specified manifestation (C10y.)
Diabetes mellitus with unspecified complication (C10z.)
Neonatal diabetes mellitus (Q441.)
Type I diabetes mellitus (X40J4)
Type II diabetes mellitus (X40J5)
Malnutrition-related diabetes mellitus (X40J7)
Secondary diabetes mellitus (X40JA)
Genetic syndromes of diabetes mellitus (X40JG)
Abnormal metabolic state in diabetes mellitus (X40Ja)
Diabetes mellitus with renal manifestation (XE10G)
Diabetes mellitus with neurological manifestation (XE10H)
Diabetes mellitus with peripheral circulatory disorder (XE10I)
Unstable diabetes (XM1Xk)
Where patient is registered at General Practice

NOT IN

Double HYP or >75
ASPIRE Study / 13 zjoins

Where patient is registered at General Practice

IN

Hypertension diagnosis
ASPIRE Study / 13 zjoins

Has a Read code in...Exact Read Codes:
Systolic hypertension (G202.)
Secondary hypertension (G24..)
Hypertension secondary to endocrine disorders (G244.)
Secondary hypertension NOS (G24z.)
Hypertension secondary to drug (G24z1)
[X]Other secondary hypertension (Gyu20)
[X]Hypertension secondary to other renal disorders (Gyu21)

Pre-exist 2ndry hypertens comp preg childbth and puerprum (L1282)
Hypertension (XE0Ub)
Diastolic hypertension (XSDSb)
Labile hypertension (Xa0Cs)
Malignant hypertension (Xa3fQ)
Read Codes and Children:
Hypertensive disease (G2...)
Malignant secondary hypertension (G240.)
Secondary benign hypertension (G241.)
Essential hypertension (XE0Uc)
Renovascular hypertension (Xa0kX)
Where patient is registered at General Practice

OR IN → **Over 75**
ASPIRE Study / 13 zjoins
Current age > 75 years
Where patient is registered at General Practice

OR IN → **4c. CVA/TIA and HF and >75 not in (HYP or DIAB)**
ASPIRE Study / 13 zjoins

IN → **Triple HF and >75 and CVA/TIA**
ASPIRE Study / 13 zjoins
Where patient is registered at General Practice

IN → **CVA and/or TIA**
ASPIRE Study / 13 zjoins
Where patient is registered at General Practice

IN → **TIA diagnosis**
ASPIRE Study / 13 zjoins
Has a Read code of Transient ischaemic attack (XE0VK) or one of its children
Where patient is registered at General Practice

OR IN → **CVA diagnosis**
ASPIRE Study / 13 zjoins
Has a Read code of Cerebrovascular accident (X00D1) or one of its children
Where patient is registered at General Practice

AND IN → **Heart Failure diagnosis**
ASPIRE Study / 13 zjoins
Has a Read code in...Exact Read Codes:
Heart failure (G58..)
Decompensated cardiac failure (G5802)
Compensated cardiac failure (G5803)
Acute heart failure (G582.)
Heart failure as a complication of care (X202k)
Right ventricular failure (X202l)
Heart failure NOS (XE0V9)
Refractory heart failure (XaEgY)
New York Heart Association classification - class I (XaJ9G)
New York Heart Association classification - class II (XaJ9H)
New York Heart Association classification - class III (XaJ9I)
New York Heart Association classification - class IV (XaJ9J)
Read Codes and Children:
Biventricular failure (XE0V8)
Left ventricular failure (XE2QG)
Where patient is registered at General Practice

AND IN → **Over 75**
ASPIRE Study / 13 zjoins
Current age > 75 years
Where patient is registered at General Practice

NOT IN → **Double HYP or DIAB**
ASPIRE Study / 13 zjoins
Where patient is registered at General Practice

IN

Hypertension diagnosis
ASPIRE Study / 13 zjoins

Has a Read code in...Exact Read Codes:
Systolic hypertension (G202.)
Secondary hypertension (G24..)
Hypertension secondary to endocrine disorders (G244.)
Secondary hypertension NOS (G24z.)
Hypertension secondary to drug (G24z1)
[X]Other secondary hypertension (Gyu20)
[X]Hypertension secondary to other renal disorders (Gyu21)
Pre-exist 2ndry hypertens comp preg childbth and puerprum (L1282)
Hypertension (XE0Ub)
Diastolic hypertension (XSDSb)
Labile hypertension (Xa0Cs)
Malignant hypertension (Xa3fQ)
Read Codes and Children:
Hypertensive disease (G2...)
Malignant secondary hypertension (G240.)
Secondary benign hypertension (G241.)
Essential hypertension (XE0Uc)
Renovascular hypertension (Xa0kX)

Where patient is registered at General Practice

OR IN

Diabetes diagnosis
ASPIRE Study / 13 zjoins

Has a Read code in...Exact Read Codes:
[Brittle] and/or [labile diabetes] (66AJ1)
Diabetes mellitus (C10..)
Diabetes mellitus with no mention of complication (C100.)
Diabetes mellitus NOS with no mention of complication (C100z)
Other specified diabetes mellitus with coma (C103y)
Other specified diabetes mellitus with multiple comps (C108y)
Unspecified diabetes mellitus with multiple complications (C108z)
Other specified diabetes mellitus with other spec comps (C10yy)
[X]Other specified diabetes mellitus (Cyu20)
[X]Unspecified diabetes mellitus with renal complications (Cyu23)
[X]Pre-existing diabetes mellitus, unspecified (Lyu29)
Insulin treated Type 2 diabetes mellitus (X40J6)
Diabetes-deafness syndrome maternally transmitted (X40JZ)
Diabetes mellitus, juvenile type, no mention of complication (XE10E)
Diabetes mellitus, adult onset, no mention of complication (XE10F)
Diabetes with other complications (XE12M)
Diabetes mellitus with gangrene (XM1Qx)
Diabetes mellitus due to insulin receptor antibodies (XSETp)
Maternally inherited diabetes mellitus (XaOPt)
Read Codes and Children:
Diabetes mellitus with ophthalmic manifestation (C105.)
Diabetes mellitus with other specified manifestation (C10y.)
Diabetes mellitus with unspecified complication (C10z.)
Neonatal diabetes mellitus (Q441.)
Type I diabetes mellitus (X40J4)
Type II diabetes mellitus (X40J5)
Malnutrition-related diabetes mellitus (X40J7)
Secondary diabetes mellitus (X40JA)
Genetic syndromes of diabetes mellitus (X40JG)
Abnormal metabolic state in diabetes mellitus (X40Ja)
Diabetes mellitus with renal manifestation (XE10G)
Diabetes mellitus with neurological manifestation (XE10H)
Diabetes mellitus with peripheral circulatory disorder (XE10I)
Unstable diabetes (XM1Xk)

Where patient is registered at General Practice

OR IN

4d. CVA/TIA and HYP and DIAB not in (HF or >75)
ASPIRE Study / 13 zjoins

IN

Triple HYP and DIAB and CVA/TIA
ASPIRE Study / 13 zjoins

Where patient is registered at General Practice

IN

Hypertension diagnosis
ASPIRE Study / 13 zjoins

Has a Read code in...Exact Read Codes:
Systolic hypertension (G202.)
Secondary hypertension (G24..)
Hypertension secondary to endocrine disorders (G244.)
Secondary hypertension NOS (G24z.)
Hypertension secondary to drug (G24z1)
[X]Other secondary hypertension (Gyu20)
[X]Hypertension secondary to other renal disorders (Gyu21)
Pre-exist 2ndry hypertens comp preg childbth and puerprum (L1282)
Hypertension (XE0Ub)
Diastolic hypertension (XSDSb)
Labile hypertension (Xa0Cs)
Malignant hypertension (Xa3fQ)
Read Codes and Children:
Hypertensive disease (G2...)
Malignant secondary hypertension (G240.)
Secondary benign hypertension (G241.)
Essential hypertension (XE0Uc)
Renovascular hypertension (Xa0kX)

Where patient is registered at General Practice

AND IN

CVA and/or TIA
ASPIRE Study / 13 zjoins

Where patient is registered at General Practice

IN

TIA diagnosis
ASPIRE Study / 13 zjoins

Has a Read code of Transient ischaemic attack (XE0VK) or one of its children

Where patient is registered at General Practice

OR IN

CVA diagnosis
ASPIRE Study / 13 zjoins

Has a Read code of Cerebrovascular accident (X00D1) or one of its children

Where patient is registered at General Practice

AND IN

Diabetes diagnosis
ASPIRE Study / 13 zjoins

Has a Read code in...Exact Read Codes:
[Brittle] and/or [labile diabetes] (66AJ1)
Diabetes mellitus (C10..)
Diabetes mellitus with no mention of complication (C100.)
Diabetes mellitus NOS with no mention of complication (C100z)
Other specified diabetes mellitus with coma (C103y)
Other specified diabetes mellitus with multiple comps (C108y)
Unspecified diabetes mellitus with multiple complications (C108z)
Other specified diabetes mellitus with other spec comps (C10yy)
[X]Other specified diabetes mellitus (Cyu20)
[X]Unspecified diabetes mellitus with renal complications (Cyu23)
[X]Pre-existing diabetes mellitus, unspecified (Lyu29)
Insulin treated Type 2 diabetes mellitus (X40J6)
Diabetes-deafness syndrome maternally transmitted (X40JZ)
Diabetes mellitus, juvenile type, no mention of complication (XE10E)
Diabetes mellitus, adult onset, no mention of complication (XE10F)
Diabetes with other complications (XE12M)
Diabetes mellitus with gangrene (XM1Qx)
Diabetes mellitus due to insulin receptor antibodies (XSETp)
Maternally inherited diabetes mellitus (XaOPt)
Read Codes and Children:
[illegible]

Diabetes mellitus with ophthalmic manifestation (C105.)
Diabetes mellitus with other specified manifestation (C10y.)
Diabetes mellitus with unspecified complication (C10z.)
Neonatal diabetes mellitus (Q441.)
Type I diabetes mellitus (X40J4)
Type II diabetes mellitus (X40J5)
Malnutrition-related diabetes mellitus (X40J7)
Secondary diabetes mellitus (X40JA)
Genetic syndromes of diabetes mellitus (X40JG)
Abnormal metabolic state in diabetes mellitus (X40Ja)
Diabetes mellitus with renal manifestation (XE10G)
Diabetes mellitus with neurological manifestation (XE10H)
Diabetes mellitus with peripheral circulatory disorder (XE10I)
Unstable diabetes (XM1Xk)
Where patient is registered at General Practice

NOT IN

Double HF or >75
ASPIRE Study / 13 zjoins
Where patient is registered at General Practice

IN

Heart Failure diagnosis
ASPIRE Study / 13 zjoins
Has a Read code in...Exact Read Codes:
Heart failure (G58..)
Decompensated cardiac failure (G5802)
Compensated cardiac failure (G5803)
Acute heart failure (G582.)
Heart failure as a complication of care (X202k)
Right ventricular failure (X202l)
Heart failure NOS (XE0V9)
Refractory heart failure (XaEgY)
New York Heart Association classification - class I (XaJ9G)
New York Heart Association classification - class II (XaJ9H)
New York Heart Association classification - class III (XaJ9I)
New York Heart Association classification - class IV (XaJ9J)
Read Codes and Children:
Biventricular failure (XE0V8)
Left ventricular failure (XE2QG)
Where patient is registered at General Practice

OR IN

Over 75
ASPIRE Study / 13 zjoins
Current age > 75 years
Where patient is registered at General Practice

OR IN

4e. CVA/TIA and HYP and >75 not in (HF or DIAB)
ASPIRE Study / 13 zjoins

IN

Triple HYP and >75 and CVA/TIA
ASPIRE Study / 13 zjoins
Where patient is registered at General Practice

IN

CVA and/or TIA
ASPIRE Study / 13 zjoins
Where patient is registered at General Practice

IN

TIA diagnosis
ASPIRE Study / 13 zjoins
Has a Read code of Transient ischaemic attack (XE0VK) or one of its children
Where patient is registered at General Practice

OR IN

CVA diagnosis
ASPIRE Study / 13 zjoins
Has a Read code of Cerebrovascular accident (X00D1) or one of its children

accident (X00D1) or one of its children
Where patient is registered at General Practice

AND IN

Over 75
ASPIRE Study / 13 zjoins

Current age > 75 years
Where patient is registered at General Practice

AND IN

Hypertension diagnosis
ASPIRE Study / 13 zjoins

Has a Read code in...Exact Read Codes:
Systolic hypertension (G202.)
Secondary hypertension (G24..)
Hypertension secondary to endocrine disorders (G244.)
Secondary hypertension NOS (G24z.)
Hypertension secondary to drug (G24z1)
[X]Other secondary hypertension (Gyu20)
[X]Hypertension secondary to other renal disorders (Gyu21)
Pre-exist 2ndry hypertens comp preg childbth and puerprum (L1282)
Hypertension (XE0Ub)
Diastolic hypertension (XSDSb)
Labile hypertension (Xa0Cs)
Malignant hypertension (Xa3fQ)
Read Codes and Children:
Hypertensive disease (G2...)
Malignant secondary hypertension (G240.)
Secondary benign hypertension (G241.)
Essential hypertension (XE0Uc)
Renovascular hypertension (Xa0kX)
Where patient is registered at General Practice

NOT IN

Double HF or DIAB
ASPIRE Study / 13 zjoins

Where patient is registered at General Practice

IN

Heart Failure diagnosis
ASPIRE Study / 13 zjoins

Has a Read code in...Exact Read Codes:
Heart failure (G58..)
Decompensated cardiac failure (G5802)
Compensated cardiac failure (G5803)
Acute heart failure (G582.)
Heart failure as a complication of care (X202k)
Right ventricular failure (X202l)
Heart failure NOS (XE0V9)
Refractory heart failure (XaEgY)
New York Heart Association classification - class I (XaJ9G)
New York Heart Association classification - class II (XaJ9H)
New York Heart Association classification - class III (XaJ9I)
New York Heart Association classification - class IV (XaJ9J)
Read Codes and Children:
Biventricular failure (XE0V8)
Left ventricular failure (XE2QG)
Where patient is registered at General Practice

OR IN

Diabetes diagnosis
ASPIRE Study / 13 zjoins

Has a Read code in...Exact Read Codes:
[Brittle] and/or [labile diabetes] (66AJ1)
Diabetes mellitus (C10..)
Diabetes mellitus with no mention of complication (C100.)
Diabetes mellitus NOS with no mention of complication (C100z)
Other specified diabetes mellitus with coma (C103y)
Other specified diabetes mellitus with multiple comps (C108y)
Unspecified diabetes mellitus with multiple complications (C108z)
Other specified diabetes mellitus with other spec comps (C10yy)
[X]Other specified diabetes mellitus (Cyu20)
[X]Unspecified diabetes mellitus with renal complications (Cyu23)
[X]Pre-existing diabetes mellitus, unspecified (Lyu29)
Insulin treated Type 2 diabetes mellitus

Insulin treated Type 2 diabetes mellitus (X40J6)
Diabetes-deafness syndrome maternally transmitted (X40JZ)
Diabetes mellitus, juvenile type, no mention of complication (XE10E)
Diabetes mellitus, adult onset, no mention of complication (XE10F)
Diabetes with other complications (XE12M)
Diabetes mellitus with gangrene (XM1Qx)
Diabetes mellitus due to insulin receptor antibodies (XSETp)
Maternally inherited diabetes mellitus (XaOPt)
Read Codes and Children:
Diabetes mellitus with ophthalmic manifestation (C105.)
Diabetes mellitus with other specified manifestation (C10y.)
Diabetes mellitus with unspecified complication (C10z.)
Neonatal diabetes mellitus (Q441.)
Type I diabetes mellitus (X40J4)
Type II diabetes mellitus (X40J5)
Malnutrition-related diabetes mellitus (X40J7)
Secondary diabetes mellitus (X40JA)
Genetic syndromes of diabetes mellitus (X40JG)
Abnormal metabolic state in diabetes mellitus (X40Ja)
Diabetes mellitus with renal manifestation (XE10G)
Diabetes mellitus with neurological manifestation (XE10H)
Diabetes mellitus with peripheral circulatory disorder (XE10I)
Unstable diabetes (XM1Xk)

Where patient is registered at General Practice

OR IN

4f. CVA/TIA and DIAB and >75 not in (HF or HYP)
ASPIRE Study / 13 zjoins

IN

Triple DIAB and >75 and CVA/TIA
ASPIRE Study / 13 zjoins

Where patient is registered at General Practice

IN

Diabetes diagnosis
ASPIRE Study / 13 zjoins

Has a Read code in...Exact Read Codes:
[Brittle] and/or [labile diabetes] (66AJ1)
Diabetes mellitus (C10..)
Diabetes mellitus with no mention of complication (C100.)
Diabetes mellitus NOS with no mention of complication (C100z)
Other specified diabetes mellitus with coma (C103y)
Other specified diabetes mellitus with multiple comps (C108y)
Unspecified diabetes mellitus with multiple complications (C108z)
Other specified diabetes mellitus with other spec comps (C10yy)
[X]Other specified diabetes mellitus (Cyu20)
[X]Unspecified diabetes mellitus with renal complications (Cyu23)
[X]Pre-existing diabetes mellitus, unspecified (Lyu29)
Insulin treated Type 2 diabetes mellitus (X40J6)
Diabetes-deafness syndrome maternally transmitted (X40JZ)
Diabetes mellitus, juvenile type, no mention of complication (XE10E)
Diabetes mellitus, adult onset, no mention of complication (XE10F)
Diabetes with other complications (XE12M)
Diabetes mellitus with gangrene (XM1Qx)
Diabetes mellitus due to insulin receptor antibodies (XSETp)
Maternally inherited diabetes mellitus (XaOPt)
Read Codes and Children:
Diabetes mellitus with ophthalmic manifestation (C105.)
Diabetes mellitus with other specified manifestation (C10y.)
Diabetes mellitus with unspecified complication (C10z.)
Neonatal diabetes mellitus (Q441.)

[illegible]
Type I diabetes mellitus (X40J4)
Type II diabetes mellitus (X40J5)
Malnutrition-related diabetes mellitus (X40J7)
Secondary diabetes mellitus (X40JA)
Genetic syndromes of diabetes mellitus (X40JG)
Abnormal metabolic state in diabetes mellitus (X40Ja)
Diabetes mellitus with renal manifestation (XE10G)
Diabetes mellitus with neurological manifestation (XE10H)
Diabetes mellitus with peripheral circulatory disorder (XE10I)
Unstable diabetes (XM1Xk)
Where patient is registered at General Practice

AND IN

Over 75
ASPIRE Study / 13 zjoins

Current age > 75 years
Where patient is registered at General Practice

AND IN

CVA and/or TIA
ASPIRE Study / 13 zjoins

Where patient is registered at General Practice

IN

TIA diagnosis
ASPIRE Study / 13 zjoins

Has a Read code of Transient ischaemic attack (XE0VK) or one of its children
Where patient is registered at General Practice

OR IN

CVA diagnosis
ASPIRE Study / 13 zjoins

Has a Read code of Cerebrovascular accident (X00D1) or one of its children
Where patient is registered at General Practice

NOT IN

Double HF or HYP
ASPIRE Study / 13 zjoins

Where patient is registered at General Practice

IN

Heart Failure diagnosis
ASPIRE Study / 13 zjoins

Has a Read code in...Exact Read Codes:
Heart failure (G58..)
Decompensated cardiac failure (G5802)
Compensated cardiac failure (G5803)
Acute heart failure (G582.)
Heart failure as a complication of care (X202k)
Right ventricular failure (X202l)
Heart failure NOS (XE0V9)
Refractory heart failure (XaEgY)
New York Heart Association classification - class I (XaJ9G)
New York Heart Association classification - class II (XaJ9H)
New York Heart Association classification - class III (XaJ9I)
New York Heart Association classification - class IV (XaJ9J)
Read Codes and Children:
Biventricular failure (XE0V8)
Left ventricular failure (XE2QG)
Where patient is registered at General Practice

OR IN

Hypertension diagnosis
ASPIRE Study / 13 zjoins

Has a Read code in...Exact Read Codes:
Systolic hypertension (G202.)
Secondary hypertension (G24..)
Hypertension secondary to endocrine disorders (G244.)
Secondary hypertension NOS (G24z.)
Hypertension secondary to drug (G24z1)
[X]Other secondary hypertension (Gyu20)
[X]Hypertension secondary to other renal disorders (Gyu21)
Pre-exist 2ndry hypertens comp preg

[illegible]
childbth and puerprum (L1282)
Hypertension (XE0Ub)
Diastolic hypertension (XSDSb)
Labile hypertension (Xa0Cs)
Malignant hypertension (Xa3fQ)
Read Codes and Children:
Hypertensive disease (G2...)
Malignant secondary hypertension (G240.)
Secondary benign hypertension (G241.)
Essential hypertension (XE0Uc)
Renovascular hypertension (Xa0kX)
Where patient is registered at General Practice

OR IN

4g. HF and HYP and DIAB and >75 not in (CVA/TIA)
ASPIRE Study / 13 zjoins

IN

Quadruple HF and HYP and DIAB and >75
ASPIRE Study / 13 zjoins

Where patient is registered at General Practice

IN

Heart Failure diagnosis
ASPIRE Study / 13 zjoins

Has a Read code in...Exact Read Codes:
Heart failure (G58..)
Decompensated cardiac failure (G5802)
Compensated cardiac failure (G5803)
Acute heart failure (G582.)
Heart failure as a complication of care (X202k)
Right ventricular failure (X202l)
Heart failure NOS (XE0V9)
Refractory heart failure (XaEgY)
New York Heart Association classification - class I (XaJ9G)
New York Heart Association classification - class II (XaJ9H)
New York Heart Association classification - class III (XaJ9I)
New York Heart Association classification - class IV (XaJ9J)
Read Codes and Children:
Biventricular failure (XE0V8)
Left ventricular failure (XE2QG)
Where patient is registered at General Practice

AND IN

Hypertension diagnosis
ASPIRE Study / 13 zjoins

Has a Read code in...Exact Read Codes:
Systolic hypertension (G202.)
Secondary hypertension (G24..)
Hypertension secondary to endocrine disorders (G244.)
Secondary hypertension NOS (G24z.)
Hypertension secondary to drug (G24z1)
[X]Other secondary hypertension (Gyu20)
[X]Hypertension secondary to other renal disorders (Gyu21)
Pre-exist 2ndry hypertens comp preg childbth and puerprum (L1282)
Hypertension (XE0Ub)
Diastolic hypertension (XSDSb)
Labile hypertension (Xa0Cs)
Malignant hypertension (Xa3fQ)
Read Codes and Children:
Hypertensive disease (G2...)
Malignant secondary hypertension (G240.)
Secondary benign hypertension (G241.)
Essential hypertension (XE0Uc)
Renovascular hypertension (Xa0kX)
Where patient is registered at General Practice

AND IN

Diabetes diagnosis
ASPIRE Study / 13 zjoins

Has a Read code in...Exact Read Codes:
[Brittle] and/or [labile diabetes] (66AJ1)
Diabetes mellitus (C10..)
Diabetes mellitus with no mention of complication (C100.)
Diabetes mellitus NOS with no mention of complication (C100z)
Other specified diabetes mellitus with coma (C103y)
Other specified diabetes mellitus with multiple comps (C108y)
Unspecified diabetes mellitus with multiple
[illegible]

complications (C108z)
Other specified diabetes mellitus with other spec comps (C10yy)
[X]Other specified diabetes mellitus (Cyu20)
[X]Unspecified diabetes mellitus with renal complications (Cyu23)
[X]Pre-existing diabetes mellitus, unspecified (Lyu29)
Insulin treated Type 2 diabetes mellitus (X40J6)
Diabetes-deafness syndrome maternally transmitted (X40JZ)
Diabetes mellitus, juvenile type, no mention of complication (XE10E)
Diabetes mellitus, adult onset, no mention of complication (XE10F)
Diabetes with other complications (XE12M)
Diabetes mellitus with gangrene (XM1Qx)
Diabetes mellitus due to insulin receptor antibodies (XSETp)
Maternally inherited diabetes mellitus (XaOPt)
Read Codes and Children:
Diabetes mellitus with ophthalmic manifestation (C105.)
Diabetes mellitus with other specified manifestation (C10y.)
Diabetes mellitus with unspecified complication (C10z.)
Neonatal diabetes mellitus (Q441.)
Type I diabetes mellitus (X40J4)
Type II diabetes mellitus (X40J5)
Malnutrition-related diabetes mellitus (X40J7)
Secondary diabetes mellitus (X40JA)
Genetic syndromes of diabetes mellitus (X40JG)
Abnormal metabolic state in diabetes mellitus (X40Ja)
Diabetes mellitus with renal manifestation (XE10G)
Diabetes mellitus with neurological manifestation (XE10H)
Diabetes mellitus with peripheral circulatory disorder (XE10I)
Unstable diabetes (XM1Xk)

Where patient is registered at General Practice

AND IN

Over 75
ASPIRE Study / 13 zjoins

Current age > 75 years
Where patient is registered at General Practice

NOT IN

CVA and/or TIA
ASPIRE Study / 13 zjoins

Where patient is registered at General Practice

IN

TIA diagnosis
ASPIRE Study / 13 zjoins

Has a Read code of Transient ischaemic attack (XE0VK) or one of its children
Where patient is registered at General Practice

OR IN

CVA diagnosis
ASPIRE Study / 13 zjoins

Has a Read code of Cerebrovascular accident (X00D1) or one of its children
Where patient is registered at General Practice

AND IN

Atrial Fibrilation diagnosis
ASPIRE Study / 13 zjoins

Has a Read code in the DRAFIB1 (Atrial fibrillation codes) QOF cluster
Show read codes in cluster DRAFIB1.

- Selecting only the most recent matching code
- Without a more recent Read code in the DRAFIB2 (Atrial fibrillation resolved codes) QOF cluster

Where patient is registered at General Practice

OR IN

5. CHAD2 Score = 5 (with AF)
ASPIRE Study / 13 zjoins

IN

5e. All CHAD2 = 5 Combinations
ASPIRE Study / 13 zjoins

IN

5a. CVA/TIA and HF and HYP and DIAB not in >75
ASPIRE Study / 13 zjoins

IN

Quadruple CVA/TIA and HF and HYP and DIAB
ASPIRE Study / 13 zjoins

Where patient is registered at General Practice

IN

Heart Failure diagnosis
ASPIRE Study / 13 zjoins

Has a Read code in...Exact Read Codes:
Heart failure (G58..)
Decompensated cardiac failure (G5802)
Compensated cardiac failure (G5803)
Acute heart failure (G582.)
Heart failure as a complication of care (X202k)
Right ventricular failure (X202l)
Heart failure NOS (XE0V9)
Refractory heart failure (XaEgY)
New York Heart Association classification - class I (XaJ9G)
New York Heart Association classification - class II (XaJ9H)
New York Heart Association classification - class III (XaJ9I)
New York Heart Association classification - class IV (XaJ9J)
Read Codes and Children:
Biventricular failure (XE0V8)
Left ventricular failure (XE2QG)

Where patient is registered at General Practice

AND IN

Hypertension diagnosis
ASPIRE Study / 13 zjoins

Has a Read code in...Exact Read Codes:
Systolic hypertension (G202.)
Secondary hypertension (G24..)
Hypertension secondary to endocrine disorders (G244.)
Secondary hypertension NOS (G24z.)
Hypertension secondary to drug (G24z1)
[X]Other secondary hypertension (Gyu20)
[X]Hypertension secondary to other renal disorders (Gyu21)
Pre-exist 2ndry hypertens comp preg childbth and puerprum (L1282)
Hypertension (XE0Ub)
Diastolic hypertension (XSDSb)
Labile hypertension (Xa0Cs)
Malignant hypertension (Xa3fQ)
Read Codes and Children:
Hypertensive disease (G2...)
Malignant secondary hypertension (G240.)
Secondary benign hypertension (G241.)
Essential hypertension (XE0Uc)
Renovascular hypertension (Xa0kX)

Where patient is registered at General Practice

AND IN

Diabetes diagnosis
ASPIRE Study / 13 zjoins

Has a Read code in...Exact Read Codes:
[Brittle] and/or [labile diabetes] (66AJ1)
Diabetes mellitus (C10..)
Diabetes mellitus with no mention of complication (C100.)
Diabetes mellitus NOS with no mention of complication (C100z)
Other specified diabetes mellitus with coma (C103y)
Other specified diabetes mellitus with multiple comps (C108y)
Unspecified diabetes mellitus with multiple complications (C108z)
Other specified diabetes mellitus with other spec comps (C10yy)
[X]Other specified diabetes mellitus (Cyu20)
[X]Unspecified diabetes mellitus with renal complications (Cyu23)
[X]Pre-existing diabetes mellitus, unspecified (Lyu29)
Insulin treated Type 2 diabetes mellitus

(X40J6)
Diabetes-deafness syndrome maternally transmitted (X40JZ)
Diabetes mellitus, juvenile type, no mention of complication (XE10E)
Diabetes mellitus, adult onset, no mention of complication (XE10F)
Diabetes with other complications (XE12M)
Diabetes mellitus with gangrene (XM1Qx)
Diabetes mellitus due to insulin receptor antibodies (XSETp)
Maternally inherited diabetes mellitus (XaOPt)
Read Codes and Children:
Diabetes mellitus with ophthalmic manifestation (C105.)
Diabetes mellitus with other specified manifestation (C10y.)
Diabetes mellitus with unspecified complication (C10z.)
Neonatal diabetes mellitus (Q441.)
Type I diabetes mellitus (X40J4)
Type II diabetes mellitus (X40J5)
Malnutrition-related diabetes mellitus (X40J7)
Secondary diabetes mellitus (X40JA)
Genetic syndromes of diabetes mellitus (X40JG)
Abnormal metabolic state in diabetes mellitus (X40Ja)
Diabetes mellitus with renal manifestation (XE10G)
Diabetes mellitus with neurological manifestation (XE10H)
Diabetes mellitus with peripheral circulatory disorder (XE10I)
Unstable diabetes (XM1Xk)

Where patient is registered at General Practice

AND IN → **CVA and/or TIA**
ASPIRE Study / 13 zjoins

Where patient is registered at General Practice

IN → **TIA diagnosis**
ASPIRE Study / 13 zjoins

Has a Read code of Transient ischaemic attack (XE0VK) or one of its children
Where patient is registered at General Practice

OR IN → **CVA diagnosis**
ASPIRE Study / 13 zjoins

Has a Read code of Cerebrovascular accident (X00D1) or one of its children
Where patient is registered at General Practice

NOT IN → **Over 75**
ASPIRE Study / 13 zjoins

Current age > 75 years
Where patient is registered at General Practice

OR IN → **5b. CVA/TIA and HF and DIAB and >75 not in HYP**
ASPIRE Study / 13 zjoins

IN → **Quadruple CVA/TIA and HF and DIAB and >75**
ASPIRE Study / 13 zjoins

Where patient is registered at General Practice

IN → **Heart Failure diagnosis**
ASPIRE Study / 13 zjoins

Has a Read code in...Exact Read Codes:
Heart failure (G58..)
Decompensated cardiac failure (G5802)
Compensated cardiac failure (G5803)
Acute heart failure (G582.)
Heart failure as a complication of care (X202k)
Right ventricular failure (X202l)
Heart failure NOS (XE0V9)
Refractory heart failure (XaEgY)
New York Heart Association classification - class I (XaJ9G)

class I (XaJ9G)
New York Heart Association classification - class II (XaJ9H)
New York Heart Association classification - class III (XaJ9I)
New York Heart Association classification - class IV (XaJ9J)
Read Codes and Children:
Biventricular failure (XE0V8)
Left ventricular failure (XE2QG)

Where patient is registered at General Practice

AND IN

CVA and/or TIA
ASPIRE Study / 13 zjoins

Where patient is registered at General Practice

IN

TIA diagnosis
ASPIRE Study / 13 zjoins

Has a Read code of Transient ischaemic attack (XE0VK) or one of its children

Where patient is registered at General Practice

OR IN

CVA diagnosis
ASPIRE Study / 13 zjoins

Has a Read code of Cerebrovascular accident (X00D1) or one of its children

Where patient is registered at General Practice

AND IN

Diabetes diagnosis
ASPIRE Study / 13 zjoins

Has a Read code in...Exact Read Codes:
[Brittle] and/or [labile diabetes] (66AJ1)
Diabetes mellitus (C10..)
Diabetes mellitus with no mention of complication (C100.)
Diabetes mellitus NOS with no mention of complication (C100z)
Other specified diabetes mellitus with coma (C103y)
Other specified diabetes mellitus with multiple comps (C108y)
Unspecified diabetes mellitus with multiple complications (C108z)
Other specified diabetes mellitus with other spec comps (C10yy)
[X]Other specified diabetes mellitus (Cyu20)
[X]Unspecified diabetes mellitus with renal complications (Cyu23)
[X]Pre-existing diabetes mellitus, unspecified (Lyu29)
Insulin treated Type 2 diabetes mellitus (X40J6)
Diabetes-deafness syndrome maternally transmitted (X40JZ)
Diabetes mellitus, juvenile type, no mention of complication (XE10E)
Diabetes mellitus, adult onset, no mention of complication (XE10F)
Diabetes with other complications (XE12M)
Diabetes mellitus with gangrene (XM1Qx)
Diabetes mellitus due to insulin receptor antibodies (XSETp)
Maternally inherited diabetes mellitus (XaOPt)
Read Codes and Children:
Diabetes mellitus with ophthalmic manifestation (C105.)
Diabetes mellitus with other specified manifestation (C10y.)
Diabetes mellitus with unspecified complication (C10z.)
Neonatal diabetes mellitus (Q441.)
Type I diabetes mellitus (X40J4)
Type II diabetes mellitus (X40J5)
Malnutrition-related diabetes mellitus (X40J7)
Secondary diabetes mellitus (X40JA)
Genetic syndromes of diabetes mellitus (X40JG)
Abnormal metabolic state in diabetes mellitus (X40Ja)
Diabetes mellitus with renal manifestation (XE10G)
Diabetes mellitus with neurological manifestation (XE10H)
Diabetes mellitus with peripheral circulatory disorder (XE10I)
Unstable diabetes (XM1Xk)

Where patient is registered at General Practice

AND IN

Over 75
ASPIRE Study / 13 zjoins

Current age > 75 years
Where patient is registered at General Practice

NOT IN

Hypertension diagnosis
ASPIRE Study / 13 zjoins

Has a Read code in...Exact Read Codes:
Systolic hypertension (G202.)
Secondary hypertension (G24..)
Hypertension secondary to endocrine disorders (G244.)
Secondary hypertension NOS (G24z.)
Hypertension secondary to drug (G24z1)
[X]Other secondary hypertension (Gyu20)
[X]Hypertension secondary to other renal disorders (Gyu21)
Pre-exist 2ndry hypertens comp preg childbth and puerprum (L1282)
Hypertension (XE0Ub)
Diastolic hypertension (XSDSb)
Labile hypertension (Xa0Cs)
Malignant hypertension (Xa3fQ)
Read Codes and Children:
Hypertensive disease (G2...)
Malignant secondary hypertension (G240.)
Secondary benign hypertension (G241.)
Essential hypertension (XE0Uc)
Renovascular hypertension (Xa0kX)
Where patient is registered at General Practice

OR IN

5c. CVA/TIA and HF and HYP and >75 not in DIAB
ASPIRE Study / 13 zjoins

IN

Quadruple CVA/TIA and HF and HYP and >75
ASPIRE Study / 13 zjoins

Where patient is registered at General Practice

IN

Heart Failure diagnosis
ASPIRE Study / 13 zjoins

Has a Read code in...Exact Read Codes:
Heart failure (G58..)
Decompensated cardiac failure (G5802)
Compensated cardiac failure (G5803)
Acute heart failure (G582.)
Heart failure as a complication of care (X202k)
Right ventricular failure (X202l)
Heart failure NOS (XE0V9)
Refractory heart failure (XaEgY)
New York Heart Association classification - class I (XaJ9G)
New York Heart Association classification - class II (XaJ9H)
New York Heart Association classification - class III (XaJ9I)
New York Heart Association classification - class IV (XaJ9J)
Read Codes and Children:
Biventricular failure (XE0V8)
Left ventricular failure (XE2QG)
Where patient is registered at General Practice

AND IN

CVA and/or TIA
ASPIRE Study / 13 zjoins

Where patient is registered at General Practice

IN

TIA diagnosis
ASPIRE Study / 13 zjoins

Has a Read code of Transient ischaemic attack (XE0VK) or one of its children
Where patient is registered at General Practice

OR IN

CVA diagnosis
ASPIRE Study / 13 zjoins

Has a Read code of Cerebrovascular

accident (X00D1) or one of its children
Where patient is registered at General Practice

AND IN

Over 75
ASPIRE Study / 13 zjoins

Current age > 75 years
Where patient is registered at General Practice

AND IN

Hypertension diagnosis
ASPIRE Study / 13 zjoins

Has a Read code in...Exact Read Codes:
Systolic hypertension (G202.)
Secondary hypertension (G24..)
Hypertension secondary to endocrine disorders (G244.)
Secondary hypertension NOS (G24z.)
Hypertension secondary to drug (G24z1)
[X]Other secondary hypertension (Gyu20)
[X]Hypertension secondary to other renal disorders (Gyu21)
Pre-exist 2ndry hypertens comp preg childbth and puerprum (L1282)
Hypertension (XE0Ub)
Diastolic hypertension (XSDSb)
Labile hypertension (Xa0Cs)
Malignant hypertension (Xa3fQ)
Read Codes and Children:
Hypertensive disease (G2...)
Malignant secondary hypertension (G240.)
Secondary benign hypertension (G241.)
Essential hypertension (XE0Uc)
Renovascular hypertension (Xa0kX)
Where patient is registered at General Practice

NOT IN

Diabetes diagnosis
ASPIRE Study / 13 zjoins

Has a Read code in...Exact Read Codes:
[Brittle] and/or [labile diabetes] (66AJ1)
Diabetes mellitus (C10..)
Diabetes mellitus with no mention of complication (C100.)
Diabetes mellitus NOS with no mention of complication (C100z)
Other specified diabetes mellitus with coma (C103y)
Other specified diabetes mellitus with multiple comps (C108y)
Unspecified diabetes mellitus with multiple complications (C108z)
Other specified diabetes mellitus with other spec comps (C10yy)
[X]Other specified diabetes mellitus (Cyu20)
[X]Unspecified diabetes mellitus with renal complications (Cyu23)
[X]Pre-existing diabetes mellitus, unspecified (Lyu29)
Insulin treated Type 2 diabetes mellitus (X40J6)
Diabetes-deafness syndrome maternally transmitted (X40JZ)
Diabetes mellitus, juvenile type, no mention of complication (XE10E)
Diabetes mellitus, adult onset, no mention of complication (XE10F)
Diabetes with other complications (XE12M)
Diabetes mellitus with gangrene (XM1Qx)
Diabetes mellitus due to insulin receptor antibodies (XSETp)
Maternally inherited diabetes mellitus (XaOPt)
Read Codes and Children:
Diabetes mellitus with ophthalmic manifestation (C105.)
Diabetes mellitus with other specified manifestation (C10y.)
Diabetes mellitus with unspecified complication (C10z.)
Neonatal diabetes mellitus (Q441.)
Type I diabetes mellitus (X40J4)
Type II diabetes mellitus (X40J5)
Malnutrition-related diabetes mellitus (X40J7)
Secondary diabetes mellitus (X40JA)
Genetic syndromes of diabetes mellitus (X40JG)
Abnormal metabolic state in diabetes mellitus (X40Ja)
Diabetes mellitus with renal manifestation (XE10G)
Diabetes mellitus with neurological

Diabetes mellitus with neurological manifestation (XE10H)
Diabetes mellitus with peripheral circulatory disorder (XE10I)
Unstable diabetes (XM1Xk)
Where patient is registered at General Practice

OR IN

5d. CVA/TIA and HYP and DIAB and >75 not in HF
ASPIRE Study / 13 zjoins

IN

Quadruple CVA/TIA and HYP and DIAB and >75
ASPIRE Study / 13 zjoins
Where patient is registered at General Practice

IN

Hypertension diagnosis
ASPIRE Study / 13 zjoins
Has a Read code in...Exact Read Codes:
Systolic hypertension (G202.)
Secondary hypertension (G24..)
Hypertension secondary to endocrine disorders (G244.)
Secondary hypertension NOS (G24z.)
Hypertension secondary to drug (G24z1)
[X]Other secondary hypertension (Gyu20)
[X]Hypertension secondary to other renal disorders (Gyu21)
Pre-exist 2ndry hypertens comp preg childbth and puerprum (L1282)
Hypertension (XE0Ub)
Diastolic hypertension (XSDSb)
Labile hypertension (Xa0Cs)
Malignant hypertension (Xa3fQ)
Read Codes and Children:
Hypertensive disease (G2...)
Malignant secondary hypertension (G240.)
Secondary benign hypertension (G241.)
Essential hypertension (XE0Uc)
Renovascular hypertension (Xa0kX)
Where patient is registered at General Practice

AND IN

CVA and/or TIA
ASPIRE Study / 13 zjoins
Where patient is registered at General Practice

IN

TIA diagnosis
ASPIRE Study / 13 zjoins
Has a Read code of Transient ischaemic attack (XE0VK) or one of its children
Where patient is registered at General Practice

OR IN

CVA diagnosis
ASPIRE Study / 13 zjoins
Has a Read code of Cerebrovascular accident (X00D1) or one of its children
Where patient is registered at General Practice

AND IN

Over 75
ASPIRE Study / 13 zjoins
Current age > 75 years
Where patient is registered at General Practice

AND IN

Diabetes diagnosis
ASPIRE Study / 13 zjoins
Has a Read code in...Exact Read Codes:
[Brittle] and/or [labile diabetes] (66AJ1)
Diabetes mellitus (C10..)
Diabetes mellitus with no mention of complication (C100.)
Diabetes mellitus NOS with no mention of complication (C100z)
Other specified diabetes mellitus with coma (C103y)
Other specified diabetes mellitus with multiple comps (C108y)
Unspecified diabetes mellitus with multiple complications (C108z)
Other specified diabetes mellitus with other spec comps (C10yy)
[X]Other specified diabetes mellitus (Cyu20)

[X]Other specified diabetes mellitus ([illegible])
[X]Unspecified diabetes mellitus with renal complications (Cyu23)
[X]Pre-existing diabetes mellitus, unspecified (Lyu29)
Insulin treated Type 2 diabetes mellitus (X40J6)
Diabetes-deafness syndrome maternally transmitted (X40JZ)
Diabetes mellitus, juvenile type, no mention of complication (XE10E)
Diabetes mellitus, adult onset, no mention of complication (XE10F)
Diabetes with other complications (XE12M)
Diabetes mellitus with gangrene (XM1Qx)
Diabetes mellitus due to insulin receptor antibodies (XSETp)
Maternally inherited diabetes mellitus (XaOPt)
Read Codes and Children:
Diabetes mellitus with ophthalmic manifestation (C105.)
Diabetes mellitus with other specified manifestation (C10y.)
Diabetes mellitus with unspecified complication (C10z.)
Neonatal diabetes mellitus (Q441.)
Type I diabetes mellitus (X40J4)
Type II diabetes mellitus (X40J5)
Malnutrition-related diabetes mellitus (X40J7)
Secondary diabetes mellitus (X40JA)
Genetic syndromes of diabetes mellitus (X40JG)
Abnormal metabolic state in diabetes mellitus (X40Ja)
Diabetes mellitus with renal manifestation (XE10G)
Diabetes mellitus with neurological manifestation (XE10H)
Diabetes mellitus with peripheral circulatory disorder (XE10I)
Unstable diabetes (XM1Xk)

Where patient is registered at General Practice

NOT IN

Heart Failure diagnosis
ASPIRE Study / 13 zjoins

Has a Read code in...Exact Read Codes:
Heart failure (G58..)
Decompensated cardiac failure (G5802)
Compensated cardiac failure (G5803)
Acute heart failure (G582.)
Heart failure as a complication of care (X202k)
Right ventricular failure (X202l)
Heart failure NOS (XE0V9)
Refractory heart failure (XaEgY)
New York Heart Association classification - class I (XaJ9G)
New York Heart Association classification - class II (XaJ9H)
New York Heart Association classification - class III (XaJ9I)
New York Heart Association classification - class IV (XaJ9J)
Read Codes and Children:
Biventricular failure (XE0V8)
Left ventricular failure (XE2QG)

Where patient is registered at General Practice

AND IN

Atrial Fibrilation diagnosis
ASPIRE Study / 13 zjoins

Has a Read code in the DRAFIB1 (Atrial fibrillation codes) QOF cluster
Show read codes in cluster DRAFIB1.

- Selecting only the most recent matching code
- Without a more recent Read code in the DRAFIB2 (Atrial fibrillation resolved codes) QOF cluster

Where patient is registered at General Practice

OR IN

6. CHAD2 Score = 6 (with AF)
ASPIRE Study / 13 zjoins

IN

6a. CVA/TIA and HF and HYP and DIAB and >75
ASPIRE Study / 13 zjoins

IN

CVA and/or TIA
ASPIRE Study / 13 zjoins

Where patient is registered at General Practice

IN

TIA diagnosis
ASPIRE Study / 13 zjoins

Has a Read code of Transient ischaemic attack (XE0VK) or one of its children

Where patient is registered at General Practice

OR IN

CVA diagnosis
ASPIRE Study / 13 zjoins

Has a Read code of Cerebrovascular accident (X00D1) or one of its children

Where patient is registered at General Practice

AND IN

Heart Failure diagnosis
ASPIRE Study / 13 zjoins

Has a Read code in...Exact Read Codes:
Heart failure (G58..)
Decompensated cardiac failure (G5802)
Compensated cardiac failure (G5803)
Acute heart failure (G582.)
Heart failure as a complication of care (X202k)
Right ventricular failure (X202l)
Heart failure NOS (XE0V9)
Refractory heart failure (XaEgY)
New York Heart Association classification - class I (XaJ9G)
New York Heart Association classification - class II (XaJ9H)
New York Heart Association classification - class III (XaJ9I)
New York Heart Association classification - class IV (XaJ9J)
Read Codes and Children:
Biventricular failure (XE0V8)
Left ventricular failure (XE2QG)

Where patient is registered at General Practice

AND IN

Hypertension diagnosis
ASPIRE Study / 13 zjoins

Has a Read code in...Exact Read Codes:
Systolic hypertension (G202.)
Secondary hypertension (G24..)
Hypertension secondary to endocrine disorders (G244.)
Secondary hypertension NOS (G24z.)
Hypertension secondary to drug (G24z1)
[X]Other secondary hypertension (Gyu20)
[X]Hypertension secondary to other renal disorders (Gyu21)
Pre-exist 2ndry hypertens comp preg childbth and puerprum (L1282)
Hypertension (XE0Ub)
Diastolic hypertension (XSDSb)
Labile hypertension (Xa0Cs)
Malignant hypertension (Xa3fQ)
Read Codes and Children:
Hypertensive disease (G2...)
Malignant secondary hypertension (G240.)
Secondary benign hypertension (G241.)
Essential hypertension (XE0Uc)
Renovascular hypertension (Xa0kX)

Where patient is registered at General Practice

AND IN

Diabetes diagnosis
ASPIRE Study / 13 zjoins

Has a Read code in...Exact Read Codes:
[Brittle] and/or [labile diabetes] (66AJ1)
Diabetes mellitus (C10..)
Diabetes mellitus with no mention of complication (C100.)
Diabetes mellitus NOS with no mention of complication (C100z)
Other specified diabetes mellitus with coma (C103y)
Other specified diabetes mellitus with multiple comps (C108y)
Unspecified diabetes mellitus with multiple complications (C108z)
Other specified diabetes mellitus with other spec comps (C10yy)
[illegible]

[X]Other specified diabetes mellitus (Cyu20)
[X]Unspecified diabetes mellitus with renal complications (Cyu23)
[X]Pre-existing diabetes mellitus, unspecified (Lyu29)
Insulin treated Type 2 diabetes mellitus (X40J6)
Diabetes-deafness syndrome maternally transmitted (X40JZ)
Diabetes mellitus, juvenile type, no mention of complication (XE10E)
Diabetes mellitus, adult onset, no mention of complication (XE10F)
Diabetes with other complications (XE12M)
Diabetes mellitus with gangrene (XM1Qx)
Diabetes mellitus due to insulin receptor antibodies (XSETp)
Maternally inherited diabetes mellitus (XaOPt)
Read Codes and Children:
Diabetes mellitus with ophthalmic manifestation (C105.)
Diabetes mellitus with other specified manifestation (C10y.)
Diabetes mellitus with unspecified complication (C10z.)
Neonatal diabetes mellitus (Q441.)
Type I diabetes mellitus (X40J4)
Type II diabetes mellitus (X40J5)
Malnutrition-related diabetes mellitus (X40J7)
Secondary diabetes mellitus (X40JA)
Genetic syndromes of diabetes mellitus (X40JG)
Abnormal metabolic state in diabetes mellitus (X40Ja)
Diabetes mellitus with renal manifestation (XE10G)
Diabetes mellitus with neurological manifestation (XE10H)
Diabetes mellitus with peripheral circulatory disorder (XE10I)
Unstable diabetes (XM1Xk)
Where patient is registered at General Practice

AND IN

Over 75
ASPIRE Study / 13 zjoins

Current age > 75 years
Where patient is registered at General Practice

AND IN

Atrial Fibrilation diagnosis
ASPIRE Study / 13 zjoins

Has a Read code in the DRAFIB1 (Atrial fibrillation codes) QOF cluster
Show read codes in cluster DRAFIB1.
- Selecting only the most recent matching code
- Without a more recent Read code in the DRAFIB2 (Atrial fibrillation resolved codes) QOF cluster

Where patient is registered at General Practice

AND IN

AF001 - Register
ASPIRE Study / 13

Has a Read code in the DRAFIB1 (Atrial fibrillation codes) QOF cluster
Show read codes in cluster DRAFIB1.
- Selecting only the most recent matching code
- Without a more recent Read code in the DRAFIB2 (Atrial fibrillation resolved codes) QOF cluster

Date of Read code before 01 Apr 2013

AND IN

Warfarin or Antiplatelet Medication
ASPIRE Study / 13

Where patient is registered at General Practice

IN

Warfarin Rx OR Warfarin Rx read code
ASPIRE Study / 13

Where patient is registered at General Practice

IN

WAR DAT - Warfarin within last 12 months

ASPIRE Study / 13

Has a Read code in the WAR (Warfarin prescription codes) QOF cluster
Show read codes in cluster WAR.

- Selecting only the most recent matching code

Date of Read code between 01 Apr 2012 and 31 Mar 2013

Where patient is registered at General Practice

OR IN

BNF 2.8.2 (oral anti-coagulants) in the last 12 months
ASPIRE Study / 13

Has medication in the 'Oral anticoagulants' Action Group

- Include all drug types

Date of medication between 01 Apr 2012 and 31 Mar 2013

Where patient is registered at General Practice

OR IN

BNF 2.9 (Antiplatelets) in the last 12 months
ASPIRE Study / 13

Has medication in the 'Antiplatelet drugs' Action Group

- Include all drug types

Date of medication between 01 Apr 2012 and 31 Mar 2013

Where patient is registered at General Practice
