# Supplementary material for: Developing ‘high impact’ guideline-based quality indicators for UK primary care: a multi-stage consensus process
Source: BMC Fam Pract. 2015 Oct 28;16:156. doi: 10.1186/s12875-015-0350-6 (PMC4624600; doi:10.1186/s12875-015-0350-6)
Supplement: Additional file 4 — Folder containing SystmOne™ search algorithms. (ZIP 12.7 mb) [file 12875_2015_350_MOESM4_ESM.zip › Aspire S1 diagrams tw edired/14D2 (CHF #42).pdf]

——	Mandatory In
- - - -	Optional In
....	Not In

14D2. BNP 100-400 or NTproBNP400-2000
ASPIRE Study / 14

- Date of any event between 01 Apr 2012 and 31 Mar 2013
- Registered before 01 Apr 2013
- Where patient is registered at General Practice

IN →

NTproBNP 400-2000pg/ml
ASPIRE Study / 14

- Most recent N terminal pro-brain natriuretic peptide level reading between 400 and 2000 pg/mL
- Date of numeric reading between 01 Apr 2012 and 31 Mar 2013
- Where patient is registered at General Practice

OR IN →

BNP 100-400 pmol/L
ASPIRE Study / 14

- Most recent Plasma pro-brain natriuretic peptide level reading between 100 and 400 pg/mL
- Date of numeric reading between 01 Apr 2012 and 31 Mar 2013
- Where patient is registered at General Practice
