# Supplementary material for: Developing ‘high impact’ guideline-based quality indicators for UK primary care: a multi-stage consensus process
Source: BMC Fam Pract. 2015 Oct 28;16:156. doi: 10.1186/s12875-015-0350-6 (PMC4624600; doi:10.1186/s12875-015-0350-6)
Supplement: Additional file 4 — Folder containing SystmOne™ search algorithms. (ZIP 12.7 mb) [file 12875_2015_350_MOESM4_ESM.zip › Aspire S1 diagrams tw edired/14D3 (CHF #42).pdf]

——	Mandatory In
- - - -	Optional In
....	Not In

14D3. BNP =>400 or NTproBNP =>2000
ASPIRE Study / 14

Date of any event between 01 Apr 2012 and 31 Mar 2013
Registered before 01 Apr 2013
Where patient is registered at General Practice

IN

NTproBNP >2000
ASPIRE Study / 14

Most recent N terminal pro-brain natriuretic peptide level reading >= 2000.0 pg/mL
Date of numeric reading between 01 Apr 2012 and 31 Mar 2013
Where patient is registered at General Practice

OR IN

BNP >400
ASPIRE Study / 14

Most recent Plasma pro-brain natriuretic peptide level reading >= 400.0 pg/mL
Date of numeric reading between 01 Apr 2012 and 31 Mar 2013
Where patient is registered at General Practice
