# Supplementary material for: Developing ‘high impact’ guideline-based quality indicators for UK primary care: a multi-stage consensus process
Source: BMC Fam Pract. 2015 Oct 28;16:156. doi: 10.1186/s12875-015-0350-6 (PMC4624600; doi:10.1186/s12875-015-0350-6)
Supplement: Additional file 4 — Folder containing SystmOne™ search algorithms. (ZIP 12.7 mb) [file 12875_2015_350_MOESM4_ESM.zip › Aspire S1 diagrams tw edired/15D3 (DM #33).pdf]

——	Mandatory In
----	Optional In
....	Not In

15D3. Type 2 Diabetes and HbA1c =>76 and prescribed 2 medication groups
ASPIRE Study / 15

Registered before 01 Apr 2013
Where patient is registered at General Practice

IN

HbA1c (=>76)
ASPIRE Study / 15

Registered before 01 Apr 2013

IN

HbA1c Cluster - XaBLm (=>76)
ASPIRE Study / 15

Most recent HbA1 reading >= 76.0 mmol/mol
Date of numeric reading between 01 Apr 2012 and 31 Mar 2013
Registered before 01 Apr 2013

OR IN

HbA1c Cluster - XaPbt (=>76)
ASPIRE Study / 15

Most recent Haemoglobin A1c level - IFCC standardised reading >= 76.0 mmol/mol
Date of numeric reading between 01 Apr 2012 and 31 Mar 2013
Registered before 01 Apr 2013

AND IN

Any 2 of the medications
ASPIRE Study / 15

IN (>=-1 joins)

Group H drugs
ASPIRE Study / 15

Has an issue of...Drugs:
dapagliflozin (form not specified)
Dapagliflozin 10mg tablets
Dapagliflozin 5mg tablets

- Include all drug types

Date of medication between 01 Apr 2012 and 31 Mar 2013
Registered before 01 Apr 2013

IN (>=-1 joins)

Group G drugs
ASPIRE Study / 15

Has an issue of...Drugs:
exenatide (form not specified)
Exenatide 10micrograms/0.04ml solution for injection 2.4ml pre-filled disposable devices
Exenatide 2mg powder and solvent for suspension for injection vials
Exenatide 5micrograms/0.02ml solution for injection 1.2ml pre-filled disposable devices
liraglutide (form not specified)
Liraglutide 6mg/ml solution for injection 3ml pre-filled disposable devices
lixisenatide (form not specified)
Lixisenatide 10micrograms/0.2ml solution for injection 3ml pre-filled disposable devices
Lixisenatide 10micrograms/0.2ml solution for injection 3ml pre-filled disposable devices and Lixisenatide 20micrograms/0.2ml solution for injection 3ml pre-filled disposable devices
Lixisenatide 20micrograms/0.2ml solution for injection 3ml pre-filled disposable devices

- Include all drug types

Date of medication between 01 Apr 2012 and 31 Mar 2013
Registered before 01 Apr 2013

IN (>=-1 joins)

Group F drugs
ASPIRE Study / 15

Has an issue of...Drugs:
linagliptin (form not specified)
Linagliptin 2.5mg / Metformin 1g tablets
Linagliptin 2.5mg / Metformin 850mg tablets
Linagliptin 5mg tablets
linagliptin with metformin (form not specified)
saxagliptin (form not specified)
Saxagliptin 2.5mg / Metformin 1g tablets
Saxagliptin 2.5mg / Metformin 850mg tablets
Saxagliptin 2.5mg tablets
Saxagliptin 5mg tablets
saxagliptin with metformin (form not

specified)
sitagliptin (form not specified)
Sitagliptin 100mg tablets
Sitagliptin 25mg tablets
Sitagliptin 50mg tablets
sitagliptin with metformin (form not specified)
sitagliptin with metformin (roi) tablets 50mg + 850mg
vildagliptin (form not specified)
Vildagliptin 50mg / Metformin 1g tablets
Vildagliptin 50mg / Metformin 850mg tablets
Vildagliptin 50mg tablets
vildagliptin with metformin (form not specified)

- Include all drug types

Date of medication between 01 Apr 2012 and 31 Mar 2013

Registered before 01 Apr 2013

IN (>=-1 joins)

Group E drugs
ASPIRE Study / 15

Has an issue of...Drugs:
nateglinide (form not specified)
Nateglinide 120mg tablets
Nateglinide 180mg tablets
Nateglinide 60mg tablets
repaglinide (form not specified)
REPAGLINIDE (Generic Manuf) (form not specified)
Repaglinide 1mg tablets
Repaglinide 1mg tablets (Actavis UK Ltd)
Repaglinide 1mg tablets (Aspire Pharma Ltd)
Repaglinide 1mg tablets (Teva UK Ltd)
Repaglinide 2mg tablets
Repaglinide 2mg tablets (Actavis UK Ltd)
Repaglinide 2mg tablets (Aspire Pharma Ltd)
Repaglinide 2mg tablets (Teva UK Ltd)
Repaglinide 500microgram tablets
Repaglinide 500microgram tablets (Actavis UK Ltd)
Repaglinide 500microgram tablets (Aspire Pharma Ltd)
Repaglinide 500microgram tablets (Teva UK Ltd)

- Include all drug types

Date of medication between 01 Apr 2012 and 31 Mar 2013

Registered before 01 Apr 2013

IN (>=-1 joins)

Group D drugs
ASPIRE Study / 15

Has an issue of...Drugs:
pioglitazone (form not specified)
PIOGLITAZONE (Generic Manuf) (form not specified)
Pioglitazone 15mg / Metformin 850mg tablets
Pioglitazone 15mg tablets
Pioglitazone 15mg tablets (A A H Pharmaceuticals Ltd)
Pioglitazone 15mg tablets (Actavis UK Ltd)
Pioglitazone 15mg tablets (Dr Reddy's Laboratories (UK) Ltd)
Pioglitazone 15mg tablets (Teva UK Ltd)
Pioglitazone 30mg tablets
Pioglitazone 30mg tablets (A A H Pharmaceuticals Ltd)
Pioglitazone 30mg tablets (Actavis UK Ltd)
Pioglitazone 30mg tablets (Dr Reddy's Laboratories (UK) Ltd)
Pioglitazone 30mg tablets (Teva UK Ltd)
Pioglitazone 45mg tablets
Pioglitazone 45mg tablets (A A H Pharmaceuticals Ltd)
Pioglitazone 45mg tablets (Actavis UK Ltd)
Pioglitazone 45mg tablets (Dr Reddy's Laboratories (UK) Ltd)
Pioglitazone 45mg tablets (Teva UK Ltd)
pioglitazone oral liquid

- Include all drug types

Date of medication between 01 Apr 2012 and 31 Mar 2013

Registered before 01 Apr 2013

IN (>=-1 joins)

Group C drugs
ASPIRE Study / 15

Has an issue of...Drugs:
acarbose (form not specified)

ACARBOSE (Generic Manuf) (form not specified)
Acarbose 100mg tablets
Acarbose 100mg tablets (A A H Pharmaceuticals Ltd)
Acarbose 100mg tablets (Actavis UK Ltd)
Acarbose 50mg tablets
Acarbose 50mg tablets (A A H Pharmaceuticals Ltd)
Acarbose 50mg tablets (Actavis UK Ltd)

- Include all drug types

Date of medication between 01 Apr 2012 and 31 Mar 2013

Registered before 01 Apr 2013

IN (>=-1 joins)

Group B drugs
ASPIRE Study / 15

Has an issue of...Drugs:
metformin (form not specified)
METFORMIN (Generic Manuf) (form not specified)
metformin (roi) tablets 1000mg
Metformin 1g modified-release tablets
Metformin 1g oral powder sachets sugar free
Metformin 500mg modified-release tablets
Metformin 500mg oral powder sachets sugar free
Metformin 500mg tablets
Metformin 500mg tablets (A A H Pharmaceuticals Ltd)
Metformin 500mg tablets (Actavis UK Ltd)
Metformin 500mg tablets (Almus Pharmaceuticals Ltd)
Metformin 500mg tablets (Generics (UK) Ltd)
Metformin 500mg tablets (IVAX Pharmaceuticals UK Ltd)
Metformin 500mg tablets (Kent Pharmaceuticals Ltd)
Metformin 500mg tablets (Ranbaxy (UK) Ltd)
Metformin 500mg tablets (Sandoz Ltd)
Metformin 500mg tablets (Teva UK Ltd)
Metformin 500mg tablets (Tillomed Laboratories Ltd)
Metformin 500mg tablets (Wockhardt UK Ltd)
Metformin 500mg tablets (Zentiva)
Metformin 500mg/5ml oral solution sugar free
Metformin 500mg/5ml oral solution sugar free (A A H Pharmaceuticals Ltd)
Metformin 500mg/5ml oral solution sugar free (Actavis UK Ltd)
Metformin 500mg/5ml oral solution sugar free (Almus Pharmaceuticals Ltd)
Metformin 500mg/5ml oral solution sugar free (Rosemont Pharmaceuticals Ltd)
Metformin 750mg modified-release tablets
Metformin 850mg capsules
Metformin 850mg tablets
Metformin 850mg tablets (A A H Pharmaceuticals Ltd)
Metformin 850mg tablets (Actavis UK Ltd)
Metformin 850mg tablets (Almus Pharmaceuticals Ltd)
Metformin 850mg tablets (Generics (UK) Ltd)
Metformin 850mg tablets (IVAX Pharmaceuticals UK Ltd)
Metformin 850mg tablets (Kent Pharmaceuticals Ltd)
Metformin 850mg tablets (Ranbaxy (UK) Ltd)
Metformin 850mg tablets (Sandoz Ltd)
Metformin 850mg tablets (Teva UK Ltd)
Metformin 850mg tablets (Tillomed Laboratories Ltd)
Metformin 850mg tablets (Wockhardt UK Ltd)
Metformin 850mg tablets (Zentiva)
metformin oral liquid
metformin oral suspension 100mg/ml
METFORMIN oral suspension 100mg/ml [ROSEMONT]
METFORMIN tablets 500mg [BERK]
METFORMIN tablets 500mg [CELLTECH]
METFORMIN tablets 500mg [LAGAP]
METFORMIN tablets 500mg [M&A PHARM]
METFORMIN tablets 500mg [NEOLAB]
METFORMIN tablets 500mg [RATIOPHARM]
METFORMIN tablets 500mg [SOVEREIGN]
METFORMIN tablets 850mg [CELLTECH]

METFORMIN tablets 850mg [CELLTECH]
METFORMIN tablets 850mg [LAGAP]
METFORMIN tablets 850mg [M&A PHARM]
METFORMIN tablets 850mg [NEOLAB]
METFORMIN tablets 850mg [RATIOPHARM]
METFORMIN tablets 850mg [SOVEREIGN]
metformin with pioglitazone tablets 850mg + 15mg

- Include all drug types

Date of medication between 01 Apr 2012 and 31 Mar 2013

Registered before 01 Apr 2013

IN (>=-1 joins)

Group A drugs
ASPIRE Study / 15

Has an issue of...Drugs:
glibenclamide (form not specified)
GLIBENCLAMIDE (Generic Manuf) (form not specified)
Glibenclamide 2.5mg tablets
Glibenclamide 2.5mg tablets (A A H Pharmaceuticals Ltd)
Glibenclamide 2.5mg tablets (Actavis UK Ltd)
Glibenclamide 2.5mg tablets (Almus Pharmaceuticals Ltd)
Glibenclamide 2.5mg tablets (Generics (UK) Ltd)
Glibenclamide 2.5mg tablets (IVAX Pharmaceuticals UK Ltd)
Glibenclamide 2.5mg tablets (Kent Pharmaceuticals Ltd)
Glibenclamide 2.5mg tablets (Teva UK Ltd)
Glibenclamide 2.5mg tablets (Wockhardt UK Ltd)
Glibenclamide 5mg tablets
Glibenclamide 5mg tablets (A A H Pharmaceuticals Ltd)
Glibenclamide 5mg tablets (Actavis UK Ltd)
Glibenclamide 5mg tablets (Almus Pharmaceuticals Ltd)
Glibenclamide 5mg tablets (Generics (UK) Ltd)
Glibenclamide 5mg tablets (IVAX Pharmaceuticals UK Ltd)
Glibenclamide 5mg tablets (Kent Pharmaceuticals Ltd)
Glibenclamide 5mg tablets (Teva UK Ltd)
Glibenclamide 5mg tablets (Wockhardt UK Ltd)
Glibenclamide 5mg/5ml oral suspension
glibenclamide oral liquid
GLIBENCLAMIDE tablets 2.5mg [BERK]
GLIBENCLAMIDE tablets 2.5mg [CELLTECH]
GLIBENCLAMIDE tablets 2.5mg [LAGAP]
GLIBENCLAMIDE tablets 5mg [BERK]
GLIBENCLAMIDE tablets 5mg [CELLTECH]
GLIBENCLAMIDE tablets 5mg [LAGAP]
GLIBENCLAMIDE tablets 5mg [NUMARK]
gliclazide (form not specified)
GLICLAZIDE (Generic Manuf) (form not specified)
gliclazide (roi) modified release tablet 60mg
Gliclazide 30mg modified-release tablets
Gliclazide 30mg modified-release tablets (A A H Pharmaceuticals Ltd)
Gliclazide 40mg tablets
Gliclazide 40mg/5ml oral suspension
Gliclazide 60mg modified-release tablets
Gliclazide 80mg tablets
Gliclazide 80mg tablets (A A H Pharmaceuticals Ltd)
Gliclazide 80mg tablets (Actavis UK Ltd)
Gliclazide 80mg tablets (Almus Pharmaceuticals Ltd)
Gliclazide 80mg tablets (Generics (UK) Ltd)
Gliclazide 80mg tablets (Genus Pharmaceuticals Ltd)
Gliclazide 80mg tablets (IVAX Pharmaceuticals UK Ltd)
Gliclazide 80mg tablets (Kent Pharmaceuticals Ltd)
Gliclazide 80mg tablets (Milpharm Ltd)
Gliclazide 80mg tablets (PLIVA Pharma Ltd)
Gliclazide 80mg tablets (Sandoz Ltd)
Gliclazide 80mg tablets (Sovereign Medical Ltd)
Gliclazide 80mg tablets (Teva UK Ltd)
Gliclazide 80mg tablets (Wockhardt UK Ltd)
Gliclazide 80mg/5ml oral suspension
Gliclazide 30mg modified-release tablets (Actavis UK Ltd)
gliclazide oral liquid

GLICLAZIDE tablets 80mg [ICE]
GLICLAZIDE tablets 80mg [MERCK-GEN]
GLICLAZIDE tablets 80mg [NEOLAB]
GLICLAZIDE tablets 80mg [STERWIN]
glimepiride (form not specified)
GLIMEPIRIDE (Generic Manuf) (form not specified)
Glimepiride 1mg tablets
Glimepiride 1mg tablets (A A H Pharmaceuticals Ltd)
Glimepiride 1mg tablets (Actavis UK Ltd)
Glimepiride 1mg tablets (Niche Generics Ltd)
Glimepiride 1mg tablets (PLIVA Pharma Ltd)
Glimepiride 1mg tablets (Sandoz Ltd)
Glimepiride 1mg tablets (Teva UK Ltd)
Glimepiride 1mg tablets (Zentiva)
Glimepiride 2mg tablets
Glimepiride 2mg tablets (A A H Pharmaceuticals Ltd)
Glimepiride 2mg tablets (Actavis UK Ltd)
Glimepiride 2mg tablets (Niche Generics Ltd)
Glimepiride 2mg tablets (PLIVA Pharma Ltd)
Glimepiride 2mg tablets (Sandoz Ltd)
Glimepiride 2mg tablets (Teva UK Ltd)
Glimepiride 2mg tablets (Zentiva)
Glimepiride 3mg tablets
Glimepiride 3mg tablets (A A H Pharmaceuticals Ltd)
Glimepiride 3mg tablets (Actavis UK Ltd)
Glimepiride 3mg tablets (Niche Generics Ltd)
Glimepiride 3mg tablets (PLIVA Pharma Ltd)
Glimepiride 3mg tablets (Sandoz Ltd)
Glimepiride 3mg tablets (Teva UK Ltd)
Glimepiride 3mg tablets (Zentiva)
Glimepiride 4mg tablets
Glimepiride 4mg tablets (A A H Pharmaceuticals Ltd)
Glimepiride 4mg tablets (Actavis UK Ltd)
Glimepiride 4mg tablets (Niche Generics Ltd)
Glimepiride 4mg tablets (Sandoz Ltd)
Glimepiride 4mg tablets (Teva UK Ltd)
Glimepiride 4mg tablets (Zentiva)
glipizide (form not specified)
GLIPIZIDE (Generic Manuf) (form not specified)
Glipizide 2.5mg tablets
Glipizide 5mg tablets
Glipizide 5mg tablets (A A H Pharmaceuticals Ltd)
Glipizide 5mg tablets (Actavis UK Ltd)
Glipizide 5mg tablets (Almus Pharmaceuticals Ltd)
Glipizide 5mg tablets (Generics (UK) Ltd)
Glipizide 5mg tablets (IVAX Pharmaceuticals UK Ltd)
Glipizide 5mg tablets (Pfizer Ltd)
Glipizide 5mg tablets (Sandoz Ltd)
Glipizide 5mg tablets (Teva UK Ltd)
tolbutamide (form not specified)
TOLBUTAMIDE (Generic Manuf) (form not specified)
Tolbutamide 500mg tablets
Tolbutamide 500mg tablets (A A H Pharmaceuticals Ltd)
Tolbutamide 500mg tablets (Actavis UK Ltd)
Tolbutamide 500mg tablets (Almus Pharmaceuticals Ltd)
Tolbutamide 500mg tablets (Generics (UK) Ltd)
Tolbutamide 500mg tablets (Kent Pharmaceuticals Ltd)
Tolbutamide 500mg tablets (Sovereign Medical Ltd)
Tolbutamide 500mg tablets (Teva UK Ltd)
tolbutamide injection 50mg/ml
tolbutamide oral liquid
TOLBUTAMIDE tablets 500mg [CELLTECH]
TOLBUTAMIDE tablets 500mg [CP PHARM]

- Include all drug types
- Date of medication between 01 Apr 2012 and 31 Mar 2013
- Registered before 01 Apr 2013

AND IN

15D1. Type 2 Diabetic - Register
ASPIRE Study / 15

- Has a Read code of Type II diabetes mellitus (X40J5) or one of its children
- Selecting only the most recent matching code
- Without a more recent Read code in...Read
