# Supplementary material for: Developing ‘high impact’ guideline-based quality indicators for UK primary care: a multi-stage consensus process
Source: BMC Fam Pract. 2015 Oct 28;16:156. doi: 10.1186/s12875-015-0350-6 (PMC4624600; doi:10.1186/s12875-015-0350-6)
Supplement: Additional file 4 — Folder containing SystmOne™ search algorithms. (ZIP 12.7 mb) [file 12875_2015_350_MOESM4_ESM.zip › Aspire S1 diagrams tw edired/16N3 (DM #36).pdf]

——	Mandatory In
- - - -	Optional In
....	Not In

16N3. Type 2 Diabetic and Total Cholesterol =<4 in the previous 15 months
ASPIRE Study / 16

Registered before 01 Apr 2013
Where patient is registered at General Practice

IN

CHOL2 =<4 in the last 15m
ASPIRE Study / 16

Has numeric reading in the CHOL2 (Total cholesterol codes with a value) nGMS cluster <= 4.0
Date of numeric reading between 01 Jan 2012 and 31 Mar 2013
Registered before 01 Apr 2013

AND IN

16D1+3. Type 2 Diabetic - Register (=>40 yrs old)
ASPIRE Study / 16

Has a Read code of Type II diabetes mellitus (X40J5) or one of its children
- Selecting only the most recent matching code
- Without a more recent Read code in...Read Codes and Children:
  Type I diabetes mellitus (X40J4)

Date of Read code before 01 Apr 2013
Born before 01 Apr 1972
Registered before 01 Apr 2013
Where patient is registered at General Practice
