# Supplementary material for: Developing ‘high impact’ guideline-based quality indicators for UK primary care: a multi-stage consensus process
Source: BMC Fam Pract. 2015 Oct 28;16:156. doi: 10.1186/s12875-015-0350-6 (PMC4624600; doi:10.1186/s12875-015-0350-6)
Supplement: Additional file 4 — Folder containing SystmOne™ search algorithms. (ZIP 12.7 mb) [file 12875_2015_350_MOESM4_ESM.zip › Aspire S1 diagrams tw edired/17N2 (Smoking #53).pdf]

Mandatory In
Optional In
Not In

17N2. Patients on ONE or more of CHD, PAD, Stroke, TIA, HTN, Diab, COPD, CKD, MH and a Current Smoker
ASPIRE Study / 17

Registered before 01 Apr 2013
Where patient is registered at General Practice

IN

17D1+2. Patients on ONE or more of CHD, PAD, Stroke, TIA, HTN, Diab, COPD, CKD, MH
ASPIRE Study / 17

Registered before 01 Apr 2013
Where patient is registered at General Practice

IN

IHD QoF Cluster
ASPIRE Study / 17

Has a Read code in the IHD (Ischaemic heart disease codes) QOF cluster
Show read codes in cluster IHD.
- Selecting only the most recent matching code

Date of Read code before 01 Apr 2013
Registered before 01 Apr 2013
Where patient is registered at General Practice

OR IN

AST001 - Register
ASPIRE Study / 17

Has a Read code in the DRAST1 (Asthma diagnosis codes) QOF cluster
Show read codes in cluster DRAST1.
- Selecting only the most recent matching code
- Without a more recent Read code in the DRAST2 (Codes for asthma resolved) QOF cluster

Date of Read code before 01 Apr 2013
Registered before 01 Apr 2013
Where patient is registered at General Practice

IN

AST001 - Asthma drugs in last 12m
ASPIRE Study / 17

Has a Read code in the DRAST3 (Asthma-related drug treatment codes) QOF cluster Show read codes in cluster DRAST3.
- Selecting only the most recent matching code

Date of Read code between 01 Apr 2012 and 31 Mar 2013
Where patient is registered at General Practice

OR IN

CKD001 - Register
ASPIRE Study / 17

Has a Read code in the DRCKD1 (Chronic kidney disease codes 3-5) QOF cluster
Show read codes in cluster DRCKD1.
- Selecting only the most recent matching code
- Selecting only new episodes
- Without a more recent Read code in the DRCKD2 (Chronic kidney disease codes 1-2) QOF cluster

Registered before 01 Apr 2013
Where patient is registered at General Practice

OR IN

COPD001 - Register
ASPIRE Study / 17

Has a Read code in the DRCOPD1 (COPD diagnosis) QOF cluster
Show read codes in cluster DRCOPD1.
- Selecting only the earliest matching code

Registered before 01 Apr 2013
Where patient is registered at General Practice

OR IN

STIA001 - Register
ASPIRE Study / 17

Has a Read code in the STRT (Stroke or [illegible]

Has a Read code in the STRT (Stroke or TIA codes) QOF cluster
Show read codes in cluster STRT.
- Selecting only the earliest matching code

Registered before 01 Apr 2013
Where patient is registered at General Practice

OR IN

PAD001 - Register
ASPIRE Study / 17

Has a Read code in the PAD (PAD diagnostic codes) QOF cluster
Show read codes in cluster PAD.
- Selecting only the earliest matching code

Registered before 01 Apr 2013
Where patient is registered at General Practice

OR IN

MH001 - Register
ASPIRE Study / 17

Registered before 01 Apr 2013
Where patient is registered at General Practice

IN

MH - Generic - Psychosis, schizophrenia or bipolar disorder
ASPIRE Study / 17

Has a Read code in the DRMH1 (Psychosis, schizophrenia + bipolar affective disease codes) QOF cluster
Show read codes in cluster DRMH1.
- Selecting only the earliest matching code

Date of Read code before 01 Apr 2013

OR IN

MH001 - On lithium within last 6 months
ASPIRE Study / 17

Has a Read code in the DRMH2 (Mental health register codes) QOF cluster
Show read codes in cluster DRMH2.
- Selecting only the most recent matching code
- Without a more recent Read code in the DRMH3 (Code for removed from mental health register) QOF cluster

Date of Read code between 01 Oct 2012 and 01 Apr 2013

OR IN

DM001 - Register
ASPIRE Study / 17

Has a Read code in the DRDM1 (Diagnostic codes for diabetes mellitus) QOF cluster
Show read codes in cluster DRDM1.
- Selecting only the most recent matching code
- Without a more recent Read code in the DRDM2 (Codes for diabetes resolved) QOF cluster

Date of Read code before 01 Apr 2013
Current age > 17 years
Registered before 01 Apr 2013
Where patient is registered at General Practice

AND IN

Current Smoker by cluster
ASPIRE Study / 17

Has a Read code in the CSMOK (Current smoker codes) QOF cluster
Show read codes in cluster CSMOK.
- Selecting only the most recent matching code
- Without a more recent Read code in the SMOK (Smoking habit codes) QOF cluster

Registered before 01 Apr 2013
Where patient is registered at General Practice
