# Supplementary material for: Developing ‘high impact’ guideline-based quality indicators for UK primary care: a multi-stage consensus process
Source: BMC Fam Pract. 2015 Oct 28;16:156. doi: 10.1186/s12875-015-0350-6 (PMC4624600; doi:10.1186/s12875-015-0350-6)
Supplement: Additional file 4 — Folder containing SystmOne™ search algorithms. (ZIP 12.7 mb) [file 12875_2015_350_MOESM4_ESM.zip › Aspire S1 diagrams tw edired/18N1 (COPD #55).pdf]

——	Mandatory In
- - - -	Optional In
....	Not In

18N1. >35 yrs old and current smoker and any of the following (Wheeze, shortness of breath, chronic cough, sputum or bronchitis) Excluding COPD patients before 31.3.12 and BMI, Chest E-ray, FBC and Post-Bronchodilator spirometry recorded
ASPIRE Study / 18

Registered before 01 Apr 2013
Where patient is registered at General Practice

IN → **BMI, Chest E-ray, FBC and Post-Bronchodilator spirometry recorded**
ASPIRE Study / 18

Where patient is registered at General Practice

IN → **FEV1 or Post-Bronchodilator Spirometry**
ASPIRE Study / 18

Where patient is registered at General Practice

IN → **Post Bronchodilator spirometry**
ASPIRE Study / 18

Has a Read code of Post bronchodilator spirometry (XaXeg)
Date of Read code between 31 Mar 2012 and 01 Apr 2013
Registered before 01 Apr 2013
Where patient is registered at General Practice

OR IN → **Codes for FEV1**
ASPIRE Study / 18

Has a Read code in the FEV1 (Codes for FEV1) QOF cluster
Show read codes in cluster FEV1.
Date of Read code between 31 Mar 2012 and 01 Apr 2013
Registered before 01 Apr 2013
Where patient is registered at General Practice

AND IN → **Full Blood Count**
ASPIRE Study / 18

Has a FBC
Date of numeric reading between 01 Apr 2012 and 31 Mar 2013
Registered before 01 Apr 2013

AND IN → **Chest X-ray**
ASPIRE Study / 18

Has a Read code in...Read Codes and Children:
Chest X-ray (XE2az)
Excluding Exact Read Codes:
Ribs X-ray (5264.)
Sternum X-ray (5265.)
Sternoclavicular joint X-ray (5266.)
Date of Read code between 01 Apr 2012 and 31 Mar 2013
Registered before 01 Apr 2013
Where patient is registered at General Practice

AND IN → **BMI**
ASPIRE Study / 18

Has a BMI
Date of numeric reading between 01 Apr 2012 and 31 Mar 2013
Registered before 01 Apr 2013

AND IN → **18D1. >35 yrs old and current smoker and any of the following (Wheeze, shortness of breath, chronic cough, sputum or bronchitis) Excluding COPD patients before 31.3.12**
ASPIRE Study / 18

Born before 01 Apr 1977
Registered before 01 Apr 2013
Where patient is registered at General Practice

Practice

NOT IN

COPD Register before 31 3 12
ASPIRE Study / 18

Has a Read code in the DRCOPD1 (COPD diagnosis) QOF cluster
Show read codes in cluster DRCOPD1.
- Selecting only the earliest matching code

Date of Read code before 31 Mar 2012
Registered before 01 Apr 2013
Where patient is registered at General Practice

AND IN

Current smoker and 2 or more cough codes OR wheese, shortness of breath, chronic cough, sputum or bronchitis coding
ASPIRE Study / 18

Where patient is registered at General Practice

IN

Current Smoker and 2 or more cough codes
ASPIRE Study / 18

Registered before 01 Apr 2013

IN

Current Smoker
ASPIRE Study / 18

Has a Read code of Smoker (137R.) or one of its children
- Selecting only the most recent matching code
- Without a more recent Read code in...Read Codes and Children:
  Ex-smoker (Ub1na)

Date of Read code before 01 Apr 2013
Registered before 01 Apr 2013
Where patient is registered at General Practice

AND IN

At least 2 cough coding in 12/13 qof year
ASPIRE Study / 18

IN (>=-1 joins)

Cough or any children Jan - March 13
ASPIRE Study / 18

Has a Read code of Cough (XE0qn) or one of its children
Date of Read code between 01 Jan 2013 and 31 Mar 2013
Registered before 01 Apr 2013
Where patient is registered at General Practice

IN (>=-1 joins)

Cough or any children Oct - Dec 12
ASPIRE Study / 18

Has a Read code of Cough (XE0qn) or one of its children
Date of Read code between 01 Oct 2012 and 31 Dec 2012
Registered before 01 Apr 2013
Where patient is registered at General Practice

IN (>=-1 joins)

Cough or any children July - September 12
ASPIRE Study / 18

Has a Read code of Cough (XE0qn) or one of its children
Date of Read code between 01 Jul 2012 and 30 Sep 2012
Registered before 01 Apr 2013
Where patient is registered at General Practice

IN (>=-1 joins)

Cough or any children march - june 12
ASPIRE Study / 18

Has a Read code of Cough (XE0qn) or one of its children
Date of Read code between 31 Mar 2012 and 30 Jun 2012
Registered before 01 Apr 2013
Where patient is registered at General Practice

OR IN

Current smoker and Wheeze, shortness of breath, chronic cough, sputum or bronchitis coding
ASPIRE Study / 18

Where patient is registered at General Practice

IN

wheeze, shortness of breath, chronic cough, sputum or bronchitis coding
ASPIRE Study / 18

Has a Read code in...Exact Read Codes:
Chronic cough (171A.)
[D]Shortness of breath (R0608)
Copious sputum (X76I6)
Moderate sputum (X76I7)
Scanty sputum (X76I8)
Read Codes and Children:
Wheeze - rhonchi (X76If)
Blood in sputum - haemoptysis (XE0qp)
Wheezing (XE0qs)
Sputum appearance (Xa0Yg)
[D]Breathlessness (XaBMU)
Bronchitis (XaDtP)
Excluding Exact Read Codes:
Other whooping cough NOS (A33yz)
Whooping cough NOS (A33z.)
[X]Whooping cough due to other Bordetella species (Ayu39)
[X]Whooping cough, unspecified (Ayu3A)
Whooping cough (XE0Qw)

Date of Read code between 31 Mar 2012 and 01 Apr 2013

Registered before 01 Apr 2013

Where patient is registered at General Practice

AND IN

Current Smoker
ASPIRE Study / 18

Has a Read code of Smoker (137R.) or one of its children

- Selecting only the most recent matching code
- Without a more recent Read code in...Read Codes and Children:
Ex-smoker (Ub1na)

Date of Read code before 01 Apr 2013

Registered before 01 Apr 2013

Where patient is registered at General Practice
