# Supplementary material for: Developing ‘high impact’ guideline-based quality indicators for UK primary care: a multi-stage consensus process
Source: BMC Fam Pract. 2015 Oct 28;16:156. doi: 10.1186/s12875-015-0350-6 (PMC4624600; doi:10.1186/s12875-015-0350-6)
Supplement: Additional file 4 — Folder containing SystmOne™ search algorithms. (ZIP 12.7 mb) [file 12875_2015_350_MOESM4_ESM.zip › Aspire S1 diagrams tw edired/2N2 (CKD #17).pdf]

——	Mandatory In
- - - -	Optional In
....	Not In

2N2. Denominator and either ACE-1 or ARB or Contraindication recorded
ASPIRE Study / 2

Registered before 01 Apr 2013
Where patient is registered at General Practice

IN

CKD register and Hypertension and Proteinuria recorded and ACE-e or ARB or Contraindication recorded
ASPIRE Study / 2

Where patient is registered at General Practice

IN

ACE or ARB - All Contraindications
ASPIRE Study / 2

Where patient is registered at General Practice

IN

CKD05 - All Contraindication Expiring last 15 months or Persisting
ASPIRE Study / 2

Where patient is registered at General Practice

IN

CKD05 - Has an AII Antagonist Contraindication Expiring code CKD05 between 1 1 12 and 31 3 13
ASPIRE Study / 2

Has a Read code in the TXAII (AII antagonist contraindications: expiring) QOF cluster Show read codes in cluster TXAII.
- Selecting only the most recent matching code

Date of Read code between 01 Jan 2012 and 31 Mar 2013
Where patient is registered at General Practice

OR IN

CKD05 - Has an AII Antagonist Contraindication Persisting Code CKD05 between 1 1 12 and 31 3 13
ASPIRE Study / 2

Has a Read code in the XAII (AII antagonist contraindications: persisting) QOF cluster Show read codes in cluster XAII.
- Selecting only the most recent matching code

Date of Read code between 01 Jan 2012 and 31 Mar 2013
Where patient is registered at General Practice

OR IN

CKD05 - Ace Contraindication Expiring last 15 months or Persisting
ASPIRE Study / 2

Where patient is registered at General Practice

IN

CKD05 - Has an Ace Contraindication Expiring code between 1 1 12 and 31 3 13
ASPIRE Study / 2

Has a Read code in the TXACE (Ace inhibitor contraindications; expiring) QOF cluster Show read codes in cluster TXACE.
- Selecting only the most recent matching code

Date of Read code between 01 Jan 2012 and 31 Mar 2013
Where patient is registered at General Practice

OR IN

CKD05 - Has an Ace Contraindication persistent code CKD05 between 1 1 12 and 31 3 13
ASPIRE Study / 2

Has a Read code in the XACE (Ace inhibitor contraindications: persistent) QOF cluster Show read codes in cluster XACE.
- Selecting only the most recent matching code

Date of Read code between 01 Jan 2012 and 31 Mar 2013
Where patient is registered at General [illegible]

Where patient is registered at General Practice

- AND IN → **2D1 + 2D2. CKD Register and Hypertension and Proteinuria recorded**
  ASPIRE Study / 2
  Registered before 01 Apr 2013
  Where patient is registered at General Practice
  - IN → **Proteinuria for CKD recorded**
    ASPIRE Study / 2
    Has a Read code in the CKDPRT (Proteinuria for CKD) QOF cluster
    Show read codes in cluster CKDPRT.
    - Selecting only the most recent matching code

    Date of Read code before 01 Apr 2013
    Where patient is registered at General Practice
  - AND IN → **Hypertension Register**
    ASPIRE Study / 2
    Has a Read code in the DRHYP1 (Hypertension diagnosis codes) QOF cluster
    Show read codes in cluster DRHYP1.
    - Selecting only the most recent matching code
    - Without a more recent Read code in the DRHYP2 (Codes for hypertension resolved) QOF cluster

    Date of Read code before 01 Apr 2013
    Where patient is registered at General Practice
  - AND IN → **CKD01 Register**
    ASPIRE Study / 2
    Has a Read code in the DRCKD1 (Chronic kidney disease codes 3-5) QOF cluster
    Show read codes in cluster DRCKD1.
    - Selecting only the most recent matching code
    - Without a more recent Read code in the DRCKD2 (Chronic kidney disease codes 1-2) QOF cluster

    Date of Read code before 01 Apr 2013
    Where patient is registered at General Practice
- OR IN → **2N1. CKD and Hypertension and Proteinuria and ACE-1 or ARB**
  ASPIRE Study / 2
  Registered before 01 Apr 2013
  Where patient is registered at General Practice
  - IN → **ACE-1 or ARB**
    ASPIRE Study / 2
    Where patient is registered at General Practice
    - IN → **BNF 2.5.5.1**
      ASPIRE Study / 2
      Has medication in the 'ACE inhibitors' Action Group
      - Include all drug types

      Date of medication between 01 Apr 2012 and 31 Mar 2013
      Where patient is registered at General Practice
    - OR IN → **BNF 2.5.5.2**
      ASPIRE Study / 2
      Has medication in the 'Angiotensin-II antagonists' Action Group
      - Include all drug types

      Date of medication between 01 Apr 2012 and 31 Mar 2013
      Where patient is registered at General Practice
  - AND IN → **2D1 + 2D2. CKD Register and Hypertension and Proteinuria recorded**
    ASPIRE Study / 2

Registered before 01 Apr 2013
Where patient is registered at General Practice

IN

Proteinuria for CKD recorded
ASPIRE Study / 2

Has a Read code in the CKDPRT (Proteinuria for CKD) QOF cluster
Show read codes in cluster CKDPRT.

- Selecting only the most recent matching code

Date of Read code before 01 Apr 2013
Where patient is registered at General Practice

AND IN

Hypertension Register
ASPIRE Study / 2

Has a Read code in the DRHYP1 (Hypertension diagnosis codes) QOF cluster
Show read codes in cluster DRHYP1.

- Selecting only the most recent matching code
- Without a more recent Read code in the DRHYP2 (Codes for hypertension resolved) QOF cluster

Date of Read code before 01 Apr 2013
Where patient is registered at General Practice

AND IN

CKD01 Register
ASPIRE Study / 2

Has a Read code in the DRCKD1 (Chronic kidney disease codes 3-5) QOF cluster
Show read codes in cluster DRCKD1.

- Selecting only the most recent matching code
- Without a more recent Read code in the DRCKD2 (Chronic kidney disease codes 1-2) QOF cluster

Date of Read code before 01 Apr 2013
Where patient is registered at General Practice
