# Supplementary material for: Developing ‘high impact’ guideline-based quality indicators for UK primary care: a multi-stage consensus process
Source: BMC Fam Pract. 2015 Oct 28;16:156. doi: 10.1186/s12875-015-0350-6 (PMC4624600; doi:10.1186/s12875-015-0350-6)
Supplement: Additional file 4 — Folder containing SystmOne™ search algorithms. (ZIP 12.7 mb) [file 12875_2015_350_MOESM4_ESM.zip › Aspire S1 diagrams tw edired/3D6 (Diabetes #34).pdf]

——	Mandatory In
- - - -	Optional In
....	Not In

3D6. Diabetics with a BMI =>30 in the last 15 months excluding Smokers
ASPIRE Study / 3

Registered before 01 Apr 2013
Where patient is registered at General Practice

IN

3N1. Diabetics with a BMI =>30 in the last 15 months
ASPIRE Study / 3

Registered before 01 Apr 2013
Where patient is registered at General Practice

IN

BMI in the last 15 months =>30
ASPIRE Study / 3

Has a BMI >= 30.0 Kg/m²
Date of numeric reading between 01 Jan 2012 and 31 Mar 2013
Where patient is registered at General Practice

AND IN

3D1 + 3D4. Type 2 Diabetic - Register
ASPIRE Study / 3

Has a Read code of Type II diabetes mellitus (X40J5) or one of its children

- Selecting only the most recent matching code
- Without a more recent Read code in...Read Codes and Children:
  Type I diabetes mellitus (X40J4)

Date of Read code before 01 Apr 2013
Registered before 01 Apr 2013
Where patient is registered at General Practice

NOT IN

Current Smoker
ASPIRE Study / 3

Has a Read code in...Exact Read Codes:
(Trivial smoker - < 1 cig/day) or (occasional smoker) (1372.)
Light cigarette smoker (1-9 cigs/day) (1373.)
Moderate cigarette smoker (10-19 cigs/day) (1374.)
Heavy cigarette smoker (20-39 cigs/day) (1375.)
Very heavy cigarette smoker (40+ cigs/day) (1376.)
Keeps trying to stop smoking (137C.)
Admitted tobacco cons untrue ? (137D.)
Trying to give up smoking (137G.)
Pipe smoker (137H.)
Cigar smoker (137J.)
Rolls own cigarettes (137M.)
Smoker (& cigarette) (137P.)
Smoking: [started] or [restarted] (137Q.)
Smoker (137R.)
Cigar consumption (Ub1tJ)
Pipe tobacco consumption (Ub1tK)
Occasional cigarette smoker (Ub1tR)
Light cigarette smoker (Ub1tS)
Moderate cigarette smoker (Ub1tT)
Heavy cigarette smoker (Ub1tU)
Very heavy cigarette smoker (Ub1tV)
Chain smoker (Ub1tW)
Trivial cigarette smoker (less than one cigarette/day) (XE0oi)
Cigarette smoker (XE0oq)
Smoking started (XE0or)
Smoking restarted (XaBSp)
Smoking reduced (XaIIu)
Thinking about stopping smoking (XaIkW)
Ready to stop smoking (XaIkX)
Not interested in stopping smoking (XaIkY)
Reason for restarting smoking (XaItg)
Minutes from waking to first tobacco consumption (XaJX2)
Wants to stop smoking (XaLQh)
Failed attempt to stop smoking (XaWNE)
Waterpipe tobacco consumption (XaZIE)
Read Codes and Children:
Smoker (137R.)

- Selecting only the most recent matching code
- Without a more recent Read code in...Read Codes and Children:
  Non-smoker (Ub0oq)

Date of Read code before 01 Apr 2013

Date of Read code before 01 Apr 2013
Where patient is registered at General Practice
