# Supplementary material for: Developing ‘high impact’ guideline-based quality indicators for UK primary care: a multi-stage consensus process
Source: BMC Fam Pract. 2015 Oct 28;16:156. doi: 10.1186/s12875-015-0350-6 (PMC4624600; doi:10.1186/s12875-015-0350-6)
Supplement: Additional file 4 — Folder containing SystmOne™ search algorithms. (ZIP 12.7 mb) [file 12875_2015_350_MOESM4_ESM.zip › Aspire S1 diagrams tw edired/3N2 (Diabetes #34).pdf]

——	Mandatory In
- - - -	Optional In
....	Not In

3N2. Diabetics and Current Smoker with Other information in the last 15months
ASPIRE Study / 3

Registered before 01 Apr 2013
Where patient is registered at General Practice

IN

3D2. Type 2 Diabetic and Current Smoker
ASPIRE Study / 3

Registered before 01 Apr 2013
Where patient is registered at General Practice

IN

3D1 + 3D4. Type 2 Diabetic - Register
ASPIRE Study / 3

Has a Read code of Type II diabetes mellitus (X40J5) or one of its children
- Selecting only the most recent matching code
- Without a more recent Read code in...Read Codes and Children:
Type I diabetes mellitus (X40J4)

Date of Read code before 01 Apr 2013
Registered before 01 Apr 2013
Where patient is registered at General Practice

AND IN

Current Smoker
ASPIRE Study / 3

Has a Read code in...Exact Read Codes:
(Trivial smoker - < 1 cig/day) or (occasional smoker) (1372.)
Light cigarette smoker (1-9 cigs/day) (1373.)
Moderate cigarette smoker (10-19 cigs/day) (1374.)
Heavy cigarette smoker (20-39 cigs/day) (1375.)
Very heavy cigarette smoker (40+ cigs/day) (1376.)
Keeps trying to stop smoking (137C.)
Admitted tobacco cons untrue ? (137D.)
Trying to give up smoking (137G.)
Pipe smoker (137H.)
Cigar smoker (137J.)
Rolls own cigarettes (137M.)
Smoker (& cigarette) (137P.)
Smoking: [started] or [restarted] (137Q.)
Smoker (137R.)
Cigar consumption (Ub1tJ)
Pipe tobacco consumption (Ub1tK)
Occasional cigarette smoker (Ub1tR)
Light cigarette smoker (Ub1tS)
Moderate cigarette smoker (Ub1tT)
Heavy cigarette smoker (Ub1tU)
Very heavy cigarette smoker (Ub1tV)
Chain smoker (Ub1tW)
Trivial cigarette smoker (less than one cigarette/day) (XE0oi)
Cigarette smoker (XE0oq)
Smoking started (XE0or)
Smoking restarted (XaBSp)
Smoking reduced (XaIIu)
Thinking about stopping smoking (XaIkW)
Ready to stop smoking (XaIkX)
Not interested in stopping smoking (XaIkY)
Reason for restarting smoking (XaItg)
Minutes from waking to first tobacco consumption (XaJX2)
Wants to stop smoking (XaLQh)
Failed attempt to stop smoking (XaWNE)
Waterpipe tobacco consumption (XaZIE)
Read Codes and Children:
Smoker (137R.)
- Selecting only the most recent matching code
- Without a more recent Read code in...Read Codes and Children:
Non-smoker (Ub0oq)

Date of Read code before 01 Apr 2013
Where patient is registered at General Practice

AND IN

Alcohol consumption, smoking support, ref to dietetics or advice on food intake or Ref to exercise or advice on exercies
ASPIRE Study / 3

Where patient is registered at General Practice

Practice

IN

Either Referral to Dietetics or Advice on diet / food intake
ASPIRE Study / 3

Where patient is registered at General Practice

IN

Referral to Dietetics
ASPIRE Study / 3

Has a Read code in...Exact Read Codes:
Referral by hospital-based dietitian (XaAbi)
Referral to dietetics service (XaAdX)
Referral to hospital-based dietetics service (XaAdZ)
Referral to community-based dietitian (XaAhZ)
Referral to hospital-based dietitian (XaAha)
Referral to dietitian (XaBSz)
Referral to dietician declined (XaIla)
Education : Referral to dietician (Y0366)
Referred to dietician (Y2292)

- Selecting only the most recent matching code

Date of Read code between 01 Jan 2012 and 31 Mar 2013

Where patient is registered at General Practice

OR IN

Dietary intake advice
ASPIRE Study / 3

Has a Read code in...Read Codes and Children:
Dietary advice (8CA4.)
Advice to change dietary intake (Ub1md)
Advice to change nutrient intake (Ub1u1)
Advice to change fat intake (Ub1u4)
Dietary advice for weight loss (Xa2jQ)
Dietary advice for disorder (Xa9yq)
Advice to carer regarding child's diet (XaJIv)
Excluding Exact Read Codes:
Infant feeding advice (67A1.)
Dietary regime (8B5..)
Dietary regime NOS (8B5Z.)
Patient advised about gluten-free diet (8CA42)
Dietary advice for breast feeding (Ub01U)
Dietary advice for gestational diabetes (Xa2hB)
Dietary advice for failure to thrive (Xa2jP)
Dietary advice for weight gain (Xa2jT)
Advice to change baby milk intake (Xa3GR)
Dietary advice for coeliac disease (Xa4Na)
Folic acid advice - pre-pregnancy (XaEDd)
Advice about weaning (XaEFl)
Advice about fluid intake (XaEFm)
Advice to carer regarding child's diet (XaJIv)
Child feeding advice (XaNxh)
Folic acid advice in first trimester of pregnancy (XaPgS)

- Selecting only the most recent matching code

Date of Read code between 01 Jan 2012 and 31 Mar 2013

Where patient is registered at General Practice

OR IN

Either Referral to Exercise therapy or Advice on Exercise
ASPIRE Study / 3

Where patient is registered at General Practice

IN

Referral to Exercise Therapy
ASPIRE Study / 3

Has a Read code in...Exact Read Codes:
Health education - exercise (6798.)
Referred for exercise programme (XaKRq)
Declined referral to physical exercise programme (XaL1X)
Referral to weight management service offered (XaXR5)
Referral to weight management special interest GP (XaZKi)
Read Codes and Children:
Referral for exercise therapy (XaIPu)
Refer to weight management programme (XaJSu)

- Selecting only the most recent matching code

[illegible]

Date of Read code between 01 Jan 2012 and 31 Mar 2013

Where patient is registered at General Practice

OR IN

Exercise advice
ASPIRE Study / 3

Has a Read code in...Exact Read Codes:
Lifestyle advice regarding exercise (XaJIt)
Education : Exercise (Y0305)
Read Codes and Children:
Advice about exercise (Xa9zF)
Advice to undertake functional activity (Xa9zR)
Excluding Exact Read Codes:
Pelvic floor exercise advice given (XaNg2)

- Selecting only the most recent matching code

Date of Read code between 01 Jan 2012 and 31 Mar 2013

Where patient is registered at General Practice

OR IN

Alcohol Consumption codes
ASPIRE Study / 3

Has a Read code in the ALC (Alcohol consumption codes) QOF cluster
Show read codes in cluster ALC.

- Selecting only the most recent matching code

Date of Read code between 01 Jan 2012 and 31 Mar 2013

Where patient is registered at General Practice

OR IN

Support and referral to Stop Smoking Service
ASPIRE Study / 3

Has a Read code in...Exact Read Codes:
Smoking cessation advice (Ua1Nz)
Advice on effects of smoking on health (Ua1O0)
Referral to stop-smoking clinic (XaFw9)
Referral to smoking cessation advisor (XaItC)
Seen by smoking cessation advisor (XaIye)
Lifestyle advice regarding smoking (XaJIs)
Referral to NHS stop smoking service (XaQT5)
Smoking cessation programme declined (XaREz)
Smoking cessation advice declined (XaRFh)
Practice based smoking cessation programme start date (XaW0h)
Consent given for follow-up by smoking cessation team (XaX5W)
Declined consent for follow-up by smoking cessation team (XaX5X)

- Selecting only the most recent matching code

Date of Read code between 01 Jan 2012 and 31 Mar 2013

Where patient is registered at General Practice
