# Supplementary material for: Developing ‘high impact’ guideline-based quality indicators for UK primary care: a multi-stage consensus process
Source: BMC Fam Pract. 2015 Oct 28;16:156. doi: 10.1186/s12875-015-0350-6 (PMC4624600; doi:10.1186/s12875-015-0350-6)
Supplement: Additional file 4 — Folder containing SystmOne™ search algorithms. (ZIP 12.7 mb) [file 12875_2015_350_MOESM4_ESM.zip › Aspire S1 diagrams tw edired/4D1 4D1 (MI #31).pdf]

——	Mandatory In
- - - -	Optional In
....	Not In

4D1 + 4D2. Patients with an MI before 1.4.13
ASPIRE Study / 4

Has a Read code in the MI (Myocardial infarction codes) QOF cluster
Show read codes in cluster MI.

- Selecting only the most recent matching code

Date of Read code before 01 Apr 2013
Registered before 01 Apr 2013
Where patient is registered at General Practice
