# Supplementary material for: Developing ‘high impact’ guideline-based quality indicators for UK primary care: a multi-stage consensus process
Source: BMC Fam Pract. 2015 Oct 28;16:156. doi: 10.1186/s12875-015-0350-6 (PMC4624600; doi:10.1186/s12875-015-0350-6)
Supplement: Additional file 4 — Folder containing SystmOne™ search algorithms. (ZIP 12.7 mb) [file 12875_2015_350_MOESM4_ESM.zip › Aspire S1 diagrams tw edired/4N1 (MI #31).pdf]

——	Mandatory In
- - - -	Optional In
....	Not In

4N1. Patients with an MI and Drug combination in the previous 12 months
ASPIRE Study / 4

Registered before 01 Apr 2013
Where patient is registered at General Practice

IN → **4D1 + 4D2. Patients with an MI before 1.4.13**
ASPIRE Study / 4

- Has a Read code in the MI (Myocardial infarction codes) QOF cluster
  Show read codes in cluster MI.
- Selecting only the most recent matching code
- Date of Read code before 01 Apr 2013
- Registered before 01 Apr 2013
- Where patient is registered at General Practice

AND IN → **Drug Combination**
ASPIRE Study / 4

Where patient is registered at General Practice

IN → **BNF 2.4 (BB)**
ASPIRE Study / 4

- Has medication in the 'Beta-blockers' Action Group
- Include all drug types
- Date of medication between 01 Apr 2012 and 31 Mar 2013
- Where patient is registered at General Practice

AND IN → **BNF 2.12.4 (Statins)**
ASPIRE Study / 4

- Has medication in the 'Statins' Action Group
- Include all drug types
- Date of medication between 01 Apr 2012 and 31 Mar 2013
- Where patient is registered at General Practice

AND IN → **Aspirin or Anti-Platelets**
ASPIRE Study / 4

Where patient is registered at General Practice

IN (optional) → **Aspirin**
ASPIRE Study / 4

Has an issue of...Drugs:
aspirin (form not specified)
ASPIRIN (Generic Manuf) (form not specified)
Aspirin 150mg suppositories
Aspirin 150mg suppositories (Martindale Pharmaceuticals Ltd)
Aspirin 300mg / Paracetamol 200mg dispersible tablets sugar free
Aspirin 300mg dispersible tablets
Aspirin 300mg dispersible tablets (A A H Pharmaceuticals Ltd)
Aspirin 300mg dispersible tablets (Actavis UK Ltd)
Aspirin 300mg dispersible tablets (Almus Pharmaceuticals Ltd)
Aspirin 300mg dispersible tablets (Aspar Pharmaceuticals Ltd)
Aspirin 300mg dispersible tablets (Kent Pharmaceuticals Ltd)
Aspirin 300mg effervescent tablets sugar free
Aspirin 300mg gastro-resistant tablets
Aspirin 300mg gastro-resistant tablets (A A H Pharmaceuticals Ltd)
Aspirin 300mg gastro-resistant tablets (Focus Pharmaceuticals Ltd)
Aspirin 300mg gastro-resistant tablets (Generics (UK) Ltd)
Aspirin 300mg gastro-resistant tablets (Sandoz Ltd)
Aspirin 300mg gastro-resistant tablets (Teva UK Ltd)
Aspirin 300mg modified-release tablets
Aspirin 300mg orodispersible tablets sugar

[illegible]
free
Aspirin 300mg suppositories
Aspirin 300mg suppositories (Martindale Pharmaceuticals Ltd)
Aspirin 300mg tablets
Aspirin 300mg tablets (A A H Pharmaceuticals Ltd)
Aspirin 300mg tablets (Actavis UK Ltd)
Aspirin 300mg tablets (Almus Pharmaceuticals Ltd)
Aspirin 300mg tablets (Aspar Pharmaceuticals Ltd)
Aspirin 300mg tablets (OBG Pharmaceuticals Ltd)
Aspirin 300mg tablets (Sigma Pharmaceuticals Plc)
Aspirin 300mg tablets (Vantage)
Aspirin 300mg tablets (Wockhardt UK Ltd)
Aspirin 325mg / Caffeine 15mg tablets
Aspirin 325mg / Caffeine 22mg tablets
Aspirin 500mg effervescent tablets sugar free
Aspirin 500mg granules sachets sugar free
Aspirin 600mg / Caffeine 50mg oral powder sachets sugar free
Aspirin 75mg dispersible tablets
Aspirin 75mg dispersible tablets (A A H Pharmaceuticals Ltd)
Aspirin 75mg dispersible tablets (Actavis UK Ltd)
Aspirin 75mg dispersible tablets (Almus Pharmaceuticals Ltd)
Aspirin 75mg dispersible tablets (Aspar Pharmaceuticals Ltd)
Aspirin 75mg dispersible tablets (IVAX Pharmaceuticals UK Ltd)
Aspirin 75mg dispersible tablets (Kent Pharmaceuticals Ltd)
Aspirin 75mg dispersible tablets (Teva UK Ltd)
Aspirin 75mg dispersible tablets (Thornton & Ross Ltd)
Aspirin 75mg dispersible tablets (Wockhardt UK Ltd)
Aspirin 75mg gastro-resistant tablets
Aspirin 75mg gastro-resistant tablets (A A H Pharmaceuticals Ltd)
Aspirin 75mg gastro-resistant tablets (Actavis UK Ltd)
Aspirin 75mg gastro-resistant tablets (Almus Pharmaceuticals Ltd)
Aspirin 75mg gastro-resistant tablets (C P Pharmaceuticals Ltd)
Aspirin 75mg gastro-resistant tablets (Generics (UK) Ltd)
Aspirin 75mg gastro-resistant tablets (IVAX Pharmaceuticals UK Ltd)
Aspirin 75mg gastro-resistant tablets (Kent Pharmaceuticals Ltd)
Aspirin 75mg gastro-resistant tablets (Sandoz Ltd)
Aspirin 75mg gastro-resistant tablets (Sterwin Medicines)
Aspirin 75mg gastro-resistant tablets (Teva UK Ltd)
Aspirin 75mg gastro-resistant tablets (Wockhardt UK Ltd)
Aspirin 75mg tablets
Aspirin 75mg tablets (A A H Pharmaceuticals Ltd)
ASPIRIN caplets 300mg [WOCKHARDT]
aspirin capsules 162.5mg
aspirin chewing gum 227mg
ASPIRIN dispersible tablet 300mg [AAH(VANT)]
ASPIRIN dispersible tablet 300mg [CP PHARM]
ASPIRIN dispersible tablet 300mg [FAMILY H]
ASPIRIN dispersible tablet 300mg [GALPHARM]
ASPIRIN dispersible tablet 300mg [M&A PHARM]
ASPIRIN dispersible tablet 300mg [NUCARE]
ASPIRIN dispersible tablet 300mg [NUMARK]
ASPIRIN dispersible tablet 300mg [RUSCO]
ASPIRIN dispersible tablet 300mg [T & R]
ASPIRIN dispersible tablet 300mg [TEVA]
ASPIRIN dispersible tablet 75mg [AAH(VANT)]
ASPIRIN dispersible tablet 75mg [GALPHARM]
ASPIRIN dispersible tablet 75mg [LEXON(PH)]

[illegible]
ASPIRIN dispersible tablet 75mg [NUCARE]
ASPIRIN dispersible tablet 75mg [NUMARK]
ASPIRIN dispersible tablet 75mg [RANBAXY]
ASPIRIN dispersible tablet 75mg [SOVEREIGN]
ASPIRIN EC tablets 300mg [AAH(VANT)]
aspirin effervescent tablets 100mg
aspirin effervescent tablets 300mg
ASPIRIN enteric coated tablets 300mg [GALEN]
ASPIRIN enteric coated tablets 300mg [PINEWOOD]
ASPIRIN enteric coated tablets 75mg [GALEN]
aspirin gastro-resistant tablets 600mg
aspirin high dose oral liquid
aspirin low dose oral liquid
aspirin mixture
aspirin modified release capsules 162.5mg
aspirin modified release tablet 100mg
aspirin modified release tablet 324mg
aspirin modified release tablet 500mg
Aspirin powder
Aspirin powder (J M Loveridge Ltd)
ASPIRIN powder [T & R]
ASPIRIN soluble tablet 300mg [CELLTECH]
ASPIRIN soluble tablet 300mg [RANBAXY]
ASPIRIN soluble tablet 75mg [CELLTECH]
ASPIRIN soluble tablet 75mg [CO-OPERATI]
ASPIRIN soluble tablet 75mg [CP PHARM]
ASPIRIN suppository 150mg [DISTRIPHAR]
ASPIRIN suppository 300mg [DISTRIPHAR]
ASPIRIN tablets 300mg [BAYER]
ASPIRIN tablets 300mg [CELLTECH]
ASPIRIN tablets 300mg [CO-OPERATI]
ASPIRIN tablets 300mg [FAMILY H]
ASPIRIN tablets 300mg [GALPHARM]
ASPIRIN tablets 300mg [M&A PHARM]
ASPIRIN tablets 300mg [NUCARE]
ASPIRIN tablets 300mg [NUMARK]
ASPIRIN tablets 300mg [RANBAXY]
ASPIRIN tablets 300mg [T & R]
ASPIRIN tablets 300mg [TEVA]
aspirin tablets 320mg
aspirin with aloxiprin and caffeine capsules
aspirin with aloxiprin and caffeine powder
aspirin with caffeine capsules 500mg + 32mg
aspirin with caffeine soluble tablet
aspirin with calcium carbonate soluble tablet 500mg
aspirin with chlorphenamine, phenylephrine and caffeine tablets 325mg
aspirin with codeine dispersible tablet 400mg + 8mg
ASPIRIN WITH CODEINE dispersible tablet [ACTAVIS]
aspirin with codeine phosphate and caffeine dispersible tablet
aspirin with codeine phosphate and caffeine tablets
aspirin with codeine soluble tablet 500mg + 8mg
aspirin with codeine tablets 400mg + 8mg
ASPIRIN WITH CODEINE tablets 400mg + 8mg [AAH(VANT)]
ASPIRIN WITH CODEINE tablets [ACTAVIS]
ASPIRIN WITH CODEINE tablets [APS]
ASPIRIN WITH CODEINE tablets [CELLTECH]
ASPIRIN WITH CODEINE tablets [CP PHARM]
ASPIRIN WITH CODEINE tablets [FAMILY H]
aspirin with cyclizine effervescent tablet 500mg+25mg
aspirin with dipyridamole modified release capsules 25mg + 200mg
aspirin with glycine chewable tablet 300mg + 150mg
aspirin with glycine dissolving tablets 300mg + 133mg
aspirin with glycine tablets
aspirin with isosorbide mononitrate modified release tablet 150mg + 60mg
aspirin with isosorbide mononitrate modified release tablet 75mg + 60mg
aspirin with methocarbamol tablets
aspirin with papaveretum dispersible tablet 500mg + 7.71mg
aspirin with sodium bicarbonate and citric acid effervescent tablet
co-codaprin (codeine and aspirin) tablets

[illegible]
8mg+400mg
Co-codaprin 8mg/400mg dispersible tablets
- Include all drug types
- Date of medication between 01 Apr 2012 and 31 Mar 2013
- Where patient is registered at General Practice

OR IN

BNF 2.9 (Antiplatlets)
ASPIRE Study / 4

- Has medication in the 'Antiplatelet drugs' Action Group
- Include all drug types
- Date of medication between 01 Apr 2012 and 31 Mar 2013
- Where patient is registered at General Practice

AND IN

ACE-1 or ARB
ASPIRE Study / 4

- Where patient is registered at General Practice

IN

BNF 2.5.5.2
ASPIRE Study / 4

- Has medication in the 'Angiotensin-II antagonists' Action Group
- Include all drug types
- Date of medication between 01 Apr 2012 and 31 Mar 2013
- Where patient is registered at General Practice

OR IN

BNF 2.5.5.1
ASPIRE Study / 4

- Has medication in the 'ACE inhibitors' Action Group
- Include all drug types
- Date of medication between 01 Apr 2012 and 31 Mar 2013
- Where patient is registered at General Practice
