# Supplementary material for: Developing ‘high impact’ guideline-based quality indicators for UK primary care: a multi-stage consensus process
Source: BMC Fam Pract. 2015 Oct 28;16:156. doi: 10.1186/s12875-015-0350-6 (PMC4624600; doi:10.1186/s12875-015-0350-6)
Supplement: Additional file 4 — Folder containing SystmOne™ search algorithms. (ZIP 12.7 mb) [file 12875_2015_350_MOESM4_ESM.zip › Aspire S1 diagrams tw edired/5D5 (HTN targets #77).pdf]

——	Mandatory In
- - - -	Optional In
....	Not In

5D5. Hypertension Reg (<80yrs old)
ASPIRE Study / 5

Born after 30 Mar 1933
Registered before 01 Apr 2013
Where patient is registered at General Practice

IN →

HYP001 - Register
ASPIRE Study / 5

Has a Read code in the DRHYP1 (Hypertension diagnosis codes) QOF cluster
Show read codes in cluster DRHYP1.

- Selecting only the most recent matching code
- Without a more recent Read code in the DRHYP2 (Codes for hypertension resolved) QOF cluster

Date of Read code before 01 Apr 2013
Registered before 01 Apr 2013
