# Supplementary material for: Developing ‘high impact’ guideline-based quality indicators for UK primary care: a multi-stage consensus process
Source: BMC Fam Pract. 2015 Oct 28;16:156. doi: 10.1186/s12875-015-0350-6 (PMC4624600; doi:10.1186/s12875-015-0350-6)
Supplement: Additional file 4 — Folder containing SystmOne™ search algorithms. (ZIP 12.7 mb) [file 12875_2015_350_MOESM4_ESM.zip › Aspire S1 diagrams tw edired/5N7 (HTN targets #77).pdf]

——	Mandatory In
- - - -	Optional In
....	Not In

5N7. Numerators N5 and N6
ASPIRE Study / 5
- Registered before 01 Apr 2014
- Where patient is registered at General Practice

IN - - -> **5N6. Hypertension Reg (=>80 years old) with BP Less than 151/91 in the last 12 months**
ASPIRE Study / 5
- Registered before 01 Apr 2013
- Where patient is registered at General Practice

IN ——> **BP Less than 150/90 in the last 12 months**
ASPIRE Study / 5
- Registered before 01 Apr 2013
- Where patient is registered at General Practice

IN - - -> **DM31 - Achievement: BP 150/90 or less in the last 12 months**
ASPIRE Study / 5
- BP Has a BP reading < 150 / 90
- Date of BP reading between 01 Apr 2012 and 31 Mar 2013
- Where patient is registered at General Practice

OR IN - - -> **DM31 - Achievement: BP Systolic and Diastolic valid in the last 12 months**
ASPIRE Study / 5
- Where patient is registered at General Practice

IN ——> **DM31 - Achievement: BP Diastolic 90 or less in the last 12 months**
ASPIRE Study / 5
- Most recent numeric reading in the BPDIA (BP diastolic codes) nGMS cluster < 90.0
- Date of numeric reading between 01 Apr 2012 and 31 Mar 2013
- Where patient is registered at General Practice

AND IN ——> **DM31 - Achievement: BP Systolic 150 or less in the last 12 months**
ASPIRE Study / 5
- Has numeric reading in the BPSYS (BP systolic codes) nGMS cluster < 150.0
- Date of numeric reading between 01 Apr 2012 and 31 Mar 2013
- Where patient is registered at General Practice

AND IN ——> **5D6. Hypertension Register (=>80 years old)**
ASPIRE Study / 5
- Born before 31 Mar 1933
- Registered before 01 Apr 2013
- Where patient is registered at General Practice

IN ——> **HYP001 - Register**
ASPIRE Study / 5
- Has a Read code in the DRHYP1 (Hypertension diagnosis codes) QOF cluster
  Show read codes in cluster DRHYP1.
  - Selecting only the most recent matching code
  - Without a more recent Read code in the DRHYP2 (Codes for hypertension resolved) QOF cluster
- Date of Read code before 01 Apr 2013
- Registered before 01 Apr 2013

OR IN - - -> **5N5. Hypertension Reg (<80yrs old) with BP Less than 140/90 in the last 12 months**
ASPIRE Study / 5
- Registered before 01 Apr 2013

[illegible]
Where patient is registered at General Practice

IN → **5D5. Hypertension Reg (<80yrs old)**
ASPIRE Study / 5

- Born after 30 Mar 1933
- Registered before 01 Apr 2013
- Where patient is registered at General Practice

  IN → **HYP001 - Register**
  ASPIRE Study / 5

  - Has a Read code in the DRHYP1 (Hypertension diagnosis codes) QOF cluster Show read codes in cluster DRHYP1.
    - Selecting only the most recent matching code
    - Without a more recent Read code in the DRHYP2 (Codes for hypertension resolved) QOF cluster
  - Date of Read code before 01 Apr 2013
  - Registered before 01 Apr 2013

AND IN → **BP Less than 140/90 in the last 12 months**
ASPIRE Study / 5

- Registered before 01 Apr 2013
- Where patient is registered at General Practice

  IN → **DM31 - Achievement: BP Systolic and Diastolic valid in the last 12 months**
  ASPIRE Study / 5

  - Where patient is registered at General Practice

    IN → **DM31 - Achievement: BP Diastolic 90 or less in the last 12 months**
    ASPIRE Study / 5

    - Has numeric reading in the BPDIA (BP diastolic codes) nGMS cluster < 90.0
    - Date of numeric reading between 01 Apr 2012 and 31 Mar 2013
    - Where patient is registered at General Practice

    AND IN → **DM31 - Achievement: BP Systolic 140 or less in the last 12 months**
    ASPIRE Study / 5

    - Has numeric reading in the BPSYS (BP systolic codes) nGMS cluster < 140.0
    - Date of numeric reading between 01 Apr 2012 and 31 Mar 2013
    - Where patient is registered at General Practice

  OR IN → **DM31 - Achievement: BP 140/90 or less in the last 12 months**
  ASPIRE Study / 5

  - Has a BP reading < 140 / 90
  - Date of BP reading between 01 Apr 2012 and 31 Mar 2013
  - Where patient is registered at General Practice
