# Supplementary material for: Developing ‘high impact’ guideline-based quality indicators for UK primary care: a multi-stage consensus process
Source: BMC Fam Pract. 2015 Oct 28;16:156. doi: 10.1186/s12875-015-0350-6 (PMC4624600; doi:10.1186/s12875-015-0350-6)
Supplement: Additional file 4 — Folder containing SystmOne™ search algorithms. (ZIP 12.7 mb) [file 12875_2015_350_MOESM4_ESM.zip › Aspire S1 diagrams tw edired/6N3 (CKD #46).pdf]

Mandatory In / Optional In / Not In

6N3. CKD Register excluding smokers and Other coding in last 12 months
ASPIRE Study / 6

Registered before 01 Apr 2013
Where patient is registered at General Practice

- IN → **6D3. CKD register excluding current smoker**
  ASPIRE Study / 6

  Registered before 01 Apr 2013
  Where patient is registered at General Practice

  - IN → **6D1 + 6D2 + 6D5. CKD01 Register**
    ASPIRE Study / 6

    Has a Read code in the DRCKD1 (Chronic kidney disease codes 3-5) QOF cluster
    Show read codes in cluster DRCKD1.
    - Selecting only the most recent matching code
    - Without a more recent Read code in the DRCKD2 (Chronic kidney disease codes 1-2) QOF cluster

    Date of Read code before 01 Apr 2013
    Registered before 01 Apr 2013
    Where patient is registered at General Practice

  - NOT IN → **Current Smoker**
    ASPIRE Study / 6

    Has a Read code in...Read Codes and Children:
    Tobacco smoking behaviour (Ub0oo)
    Excluding Exact Read Codes:
    Tobacco smoking behaviour (Ub0oo)
    Smoking Target Notes (Y0018)
    Excluding Read Codes Branches:
    Non-smoker (Ub0oq)
    Smoking cessation milestones (XaIQi)
    - Selecting only the most recent matching code

    Date of Read code before 01 Apr 2013
    Where patient is registered at General Practice

- AND IN → **BMI=<30 with lifestyle advice OR referral to exercise therapy or advice on exercise**
  ASPIRE Study / 6

  Where patient is registered at General Practice

  - IN → **Either BMI <30 or Lifestyle advice**
    ASPIRE Study / 6

    Where patient is registered at General Practice

    - IN → **Lifestyle counselling**
      ASPIRE Study / 6

      Has a Read code in...Exact Read Codes:
      Lifestyle counselling (XaEFY)
      Lifestyle advice regarding diet (XaQaU)
      Date of Read code between 01 Apr 2012 and 31 Mar 2013
      Where patient is registered at General Practice

    - OR IN → **BMI <30**
      ASPIRE Study / 6

      Has a BMI < 30.0 Kg/m²
      Date of numeric reading before 01 Apr 2013

  - OR IN → **Either Referral to Exercise therapy or Advice on Exercise**
    ASPIRE Study / 6

    Where patient is registered at General Practice

    - IN → **Exercise advice**
      ASPIRE Study / 6

      Has a Read code in...Exact Read Codes:
      Lifestyle advice regarding exercise (XaJIt)
      [illegible]

Education : Exercise (Y0305)
Read Codes and Children:
Advice about exercise (Xa9zF)
Advice to undertake functional activity (Xa9zR)
Excluding Exact Read Codes:
Pelvic floor exercise advice given (XaNg2)

- Selecting only the most recent matching code

Date of Read code between 01 Apr 2012 and 31 Mar 2013

Where patient is registered at General Practice

OR IN

Referral to Exercise Therapy
ASPIRE Study / 6

Has a Read code in...Exact Read Codes:
Health education - exercise (6798.)
Referred for exercise programme (XaKRq)
Declined referral to physical exercise programme (XaL1X)
Referral to weight management service offered (XaXR5)
Referral to weight management special interest GP (XaZKi)
Read Codes and Children:
Referral for exercise therapy (XaIPu)
Refer to weight management programme (XaJSu)

- Selecting only the most recent matching code

Date of Read code between 01 Apr 2012 and 31 Mar 2013

Where patient is registered at General Practice
