# Supplementary material for: Developing ‘high impact’ guideline-based quality indicators for UK primary care: a multi-stage consensus process
Source: BMC Fam Pract. 2015 Oct 28;16:156. doi: 10.1186/s12875-015-0350-6 (PMC4624600; doi:10.1186/s12875-015-0350-6)
Supplement: Additional file 4 — Folder containing SystmOne™ search algorithms. (ZIP 12.7 mb) [file 12875_2015_350_MOESM4_ESM.zip › Aspire S1 diagrams tw edired/6N5 (CKD #46).pdf]

Mandatory In
Optional In
Not In

6N5. CKD Reg and BP AND acr/pcr AND Lifestyle advice
ASPIRE Study / 6

Registered before 01 Apr 2013
Where patient is registered at General Practice

IN

6N1. CKD Register and BP recorded in previous 15 months
ASPIRE Study / 6

Registered before 01 Apr 2013
Where patient is registered at General Practice

IN

BP Exists in the last 15 months
ASPIRE Study / 6

Where patient is registered at General Practice

IN

BP Systolic and Diastolic valid in the last 15 months
ASPIRE Study / 6

Where patient is registered at General Practice

IN

BP Diastolic =>30 in the last 15 months
ASPIRE Study / 6

Has numeric reading in the BPDIA (BP diastolic codes) nGMS cluster >= 30.0
Date of numeric reading between 01 Jan 2012 and 31 Mar 2013
Where patient is registered at General Practice

AND IN

BP Systolic =>30 in the last 15 months
ASPIRE Study / 6

Has numeric reading in the BPSYS (BP systolic codes) nGMS cluster >= 30.0
Date of numeric reading between 01 Jan 2012 and 31 Mar 2013
Where patient is registered at General Practice

OR IN

BP exists (>30/30) in the last 15 months
ASPIRE Study / 6

BP Has a BP reading > 30 / 30
Date of BP reading between 01 Jan 2012 and 31 Mar 2013
Where patient is registered at General Practice

AND IN

6D1 + 6D2 + 6D5. CKD01 Register
ASPIRE Study / 6

Has a Read code in the DRCKD1 (Chronic kidney disease codes 3-5) QOF cluster Show read codes in cluster DRCKD1.

- Selecting only the most recent matching code
- Without a more recent Read code in the DRCKD2 (Chronic kidney disease codes 1-2) QOF cluster

Date of Read code before 01 Apr 2013
Registered before 01 Apr 2013
Where patient is registered at General Practice

AND IN

6N2. CKD Register and ACR or PCR recorded in previous 15 months
ASPIRE Study / 6

Registered before 01 Apr 2013
Where patient is registered at General Practice

IN

ACR or PCR recorded in the last 15 months
ASPIRE Study / 6

Has numeric reading in the APCR (Codes for Albumin:Creatinine & Protein:Creatinine Ratio for CKD) nGMS cluster
Date of numeric reading between 01 Jan 2012 and 31 Mar 2013
Where patient is registered at General

[illegible]
Practice

AND IN

6D1 + 6D2 + 6D5. CKD01 Register
ASPIRE Study / 6

Has a Read code in the DRCKD1 (Chronic kidney disease codes 3-5) QOF cluster
Show read codes in cluster DRCKD1.

- Selecting only the most recent matching code
- Without a more recent Read code in the DRCKD2 (Chronic kidney disease codes 1-2) QOF cluster

Date of Read code before 01 Apr 2013
Registered before 01 Apr 2013
Where patient is registered at General Practice

AND IN

6N3. CKD Register excluding smokers and Other coding in last 12 months
ASPIRE Study / 6

Registered before 01 Apr 2013
Where patient is registered at General Practice

IN

6D3. CKD register excluding current smoker
ASPIRE Study / 6

Registered before 01 Apr 2013
Where patient is registered at General Practice

IN

6D1 + 6D2 + 6D5. CKD01 Register
ASPIRE Study / 6

Has a Read code in the DRCKD1 (Chronic kidney disease codes 3-5) QOF cluster
Show read codes in cluster DRCKD1.

- Selecting only the most recent matching code
- Without a more recent Read code in the DRCKD2 (Chronic kidney disease codes 1-2) QOF cluster

Date of Read code before 01 Apr 2013
Registered before 01 Apr 2013
Where patient is registered at General Practice

NOT IN

Current Smoker
ASPIRE Study / 6

Has a Read code in...Read Codes and Children:
Tobacco smoking behaviour (Ub0oo)
Excluding Exact Read Codes:
Tobacco smoking behaviour (Ub0oo)
Smoking Target Notes (Y0018)
Excluding Read Codes Branches:
Non-smoker (Ub0oq)
Smoking cessation milestones (XaIQi)

- Selecting only the most recent matching code

Date of Read code before 01 Apr 2013
Where patient is registered at General Practice

AND IN

BMI=<30 with lifestyle advice OR referral to exercise therapy or advice on exercise
ASPIRE Study / 6

Where patient is registered at General Practice

IN

Either BMI <30 or Lifestyle advice
ASPIRE Study / 6

Where patient is registered at General Practice

IN

Lifestyle counselling
ASPIRE Study / 6

Has a Read code in...Exact Read Codes:
Lifestyle counselling (XaEFY)
Lifestyle advice regarding diet (XaQaU)
Date of Read code between 01 Apr 2012 and 31 Mar 2013
Where patient is registered at General Practice

OR IN → **BMI <30**
ASPIRE Study / 6

Has a BMI < 30.0 Kg/m²
Date of numeric reading before 01 Apr 2013

OR IN → **Either Referral to Exercise therapy or Advice on Exercise**
ASPIRE Study / 6

Where patient is registered at General Practice

IN → **Exercise advice**
ASPIRE Study / 6

Has a Read code in...Exact Read Codes:
Lifestyle advice regarding exercise (XaJIt)
Education : Exercise (Y0305)
Read Codes and Children:
Advice about exercise (Xa9zF)
Advice to undertake functional activity (Xa9zR)
Excluding Exact Read Codes:
Pelvic floor exercise advice given (XaNg2)

- Selecting only the most recent matching code

Date of Read code between 01 Apr 2012 and 31 Mar 2013
Where patient is registered at General Practice

OR IN → **Referral to Exercise Therapy**
ASPIRE Study / 6

Has a Read code in...Exact Read Codes:
Health education - exercise (6798.)
Referred for exercise programme (XaKRq)
Declined referral to physical exercise programme (XaL1X)
Referral to weight management service offered (XaXR5)
Referral to weight management special interest GP (XaZKi)
Read Codes and Children:
Referral for exercise therapy (XaIPu)
Refer to weight management programme (XaJSu)

- Selecting only the most recent matching code

Date of Read code between 01 Apr 2012 and 31 Mar 2013
Where patient is registered at General Practice
