# Supplementary material for: Developing ‘high impact’ guideline-based quality indicators for UK primary care: a multi-stage consensus process
Source: BMC Fam Pract. 2015 Oct 28;16:156. doi: 10.1186/s12875-015-0350-6 (PMC4624600; doi:10.1186/s12875-015-0350-6)
Supplement: Additional file 4 — Folder containing SystmOne™ search algorithms. (ZIP 12.7 mb) [file 12875_2015_350_MOESM4_ESM.zip › Aspire S1 diagrams tw edired/7N2 (CKD #47).pdf]

——	Mandatory In
- - - -	Optional In
....	Not In

7N2. CKD Register and Diabetes register or ACR =>70 or PCR =>100 or Urinry protein excretion =>1 and BP < 130/80
ASPIRE Study / 7

Registered before 01 Apr 2013
Where patient is registered at General Practice

- IN → **7D2. CKD Register and Diabetes register or ACR =>70 or PCR =>100 or Urinry protein excretion =>1**
  ASPIRE Study / 7

  Registered before 01 Apr 2013
  Where patient is registered at General Practice

  - IN → **7D1. CKD01 Register**
    ASPIRE Study / 7

    Has a Read code in the DRCKD1 (Chronic kidney disease codes 3-5) QOF cluster Show read codes in cluster DRCKD1.
    - Selecting only the most recent matching code
    - Without a more recent Read code in the DRCKD2 (Chronic kidney disease codes 1-2) QOF cluster

    Date of Read code before 01 Apr 2013
    Registered before 01 Apr 2013
    Where patient is registered at General Practice

  - AND IN → **Diabetes register or ACR =>70 OR PCR =>100 OR Urinary protein =>1**
    ASPIRE Study / 7

    Where patient is registered at General Practice

    - IN → **ACR =>70**
      ASPIRE Study / 7

      Most recent Urine albumin/creatinine ratio reading >= 70.0 mg/mmol
      - Without a more recent Urine albumin/creatinine ratio reading < 70.0 mg/mmol

      Date of numeric reading before 01 Apr 2013
      Where patient is registered at General Practice

    - OR IN → **Diabetes Register**
      ASPIRE Study / 7

      Has a Read code in the DRDM1 (Diagnostic codes for diabetes mellitus) QOF cluster Show read codes in cluster DRDM1.
      - Selecting only the most recent matching code
      - Without a more recent Read code in the DRDM2 (Codes for diabetes resolved) QOF cluster

      Date of Read code before 01 Apr 2013
      Current age > 17 years
      Where patient is registered at General Practice

    - OR IN → **PCR =>100**
      ASPIRE Study / 7

      Most recent Urine porphyrin/creatinine ratio reading >= 100.0 nmol/mmol Creatinine
      - Without a more recent Urine porphyrin/creatinine ratio reading < 100.0 nmol/mmol Creatinine

      Date of numeric reading before 01 Apr 2013
      Where patient is registered at General Practice

    - OR IN → **Urine protein level =>1**
      ASPIRE Study / 7

      Most recent Urine protein level reading >= 1.0 g/L
      - Without a more recent Urine protein level reading < 1.0 g/L

      Date of numeric reading before 01 Apr 2013
      Where patient is registered at General Practice

Practice

- AND IN → **BP <130/80**
  ASPIRE Study / 7
  - Where patient is registered at General Practice
  - IN → **DM31 - Systolic =<130 in the previous 12 months AND Diastolic =<80**
    ASPIRE Study / 7
    - Where patient is registered at General Practice
    - IN → **DM31 - Achievement: BP Diastolic exists in the last 15 months**
      ASPIRE Study / 7
      - Has numeric reading in the BPDIA (BP diastolic codes) nGMS cluster < 80.0
      - Date of numeric reading between 01 Jan 2012 and 31 Mar 2013
      - Registered before 01 Apr 2013
      - Where patient is registered at General Practice
    - AND IN → **DM31 - Achievement: BP Systolic <130 in the last 15 months**
      ASPIRE Study / 7
      - Has numeric reading in the BPSYS (BP systolic codes) nGMS cluster < 130.0
      - Date of numeric reading between 01 Jan 2012 and 31 Mar 2013
      - Registered before 01 Apr 2013
      - Where patient is registered at General Practice
  - AND IN → **DM31 - Achievement: BP <130/80 in the last 15 months**
    ASPIRE Study / 7
    - Has a BP reading < 130 / 80
    - Date of BP reading between 01 Jan 2012 and 31 Mar 2013
    - Registered before 01 Apr 2013
    - Where patient is registered at General Practice
