# Supplementary material for: Developing ‘high impact’ guideline-based quality indicators for UK primary care: a multi-stage consensus process
Source: BMC Fam Pract. 2015 Oct 28;16:156. doi: 10.1186/s12875-015-0350-6 (PMC4624600; doi:10.1186/s12875-015-0350-6)
Supplement: Additional file 4 — Folder containing SystmOne™ search algorithms. (ZIP 12.7 mb) [file 12875_2015_350_MOESM4_ESM.zip › Aspire S1 diagrams tw edired/7N3 (CKD #47).pdf]

Mandatory In
Optional In
Not In

7N3. CKD and Hypertension Register and ACR =>30 or PCT =>50 or urinary protein =>0.5 and either ACE or ARB-1
ASPIRE Study / 7

- Registered before 01 Apr 2013
- Where patient is registered at General Practice

IN → **ACE-1 or ARB**
ASPIRE Study / 7

- Where patient is registered at General Practice

IN → **BNF 2.5.5.2 (Angiotensin 2)**
ASPIRE Study / 7

- Has medication in the 'Angiotensin-II antagonists' Action Group
- Include all drug types
- Date of medication between 01 Apr 2012 and 31 Mar 2013
- Where patient is registered at General Practice

OR IN → **BNF 2.5.5.1**
ASPIRE Study / 7

- Has medication in the 'ACE inhibitors' Action Group
- Include all drug types
- Date of medication between 01 Apr 2012 and 31 Mar 2013
- Where patient is registered at General Practice

AND IN → **7D3 + 7D4. CKD and Hypertension Register and ACR =>30 or PCT =>50 or urinary protein =>0.5 (Excluding Diabetic Reg)**
ASPIRE Study / 7

- Registered before 01 Apr 2013
- Where patient is registered at General Practice

IN → **CKD and Hypertension Register and ACR =>30 or PCT =>50 or urinary protein =>0.5**
ASPIRE Study / 7

- Registered before 01 Apr 2013
- Where patient is registered at General Practice

IN → **ACR =>30 OR PCR =>50 or Urinary protein excretion =>0.5**
ASPIRE Study / 7

- Where patient is registered at General Practice

IN → **ACR =>30**
ASPIRE Study / 7

- Most recent Urine albumin/creatinine ratio reading >= 30.0 mg/mmol
- Without a more recent Urine albumin/creatinine ratio reading < 30.0 mg/mmol
- Date of numeric reading before 01 Apr 2013
- Where patient is registered at General Practice

OR IN → **PCR =>50**
ASPIRE Study / 7

- Most recent Urine porphyrin/creatinine ratio reading >= 50.0 nmol/mmol Creatinine
- Without a more recent Urine porphyrin/creatinine ratio reading < 50.0 nmol/mmol Creatinine
- Date of numeric reading before 01 Apr 2013
- Where patient is registered at General Practice

OR IN → **Urine protein level =>0.5**
ASPIRE Study / 7

- Most recent Urine protein level reading >= 0.5 g/L

- Without a more recent Urine protein level reading < 0.5 g/L
- Date of numeric reading before 01 Apr 2013
- Where patient is registered at General Practice

AND IN

CKD and Hypertension Register
ASPIRE Study / 7

Where patient is registered at General Practice

IN

7D1. CKD01 Register
ASPIRE Study / 7

Has a Read code in the DRCKD1 (Chronic kidney disease codes 3-5) QOF cluster
Show read codes in cluster DRCKD1.
- Selecting only the most recent matching code
- Without a more recent Read code in the DRCKD2 (Chronic kidney disease codes 1-2) QOF cluster

Date of Read code before 01 Apr 2013
Registered before 01 Apr 2013
Where patient is registered at General Practice

AND IN

Hypertension Register
ASPIRE Study / 7

Has a Read code in the DRHYP1 (Hypertension diagnosis codes) QOF cluster
Show read codes in cluster DRHYP1.
- Selecting only the most recent matching code
- Without a more recent Read code in the DRHYP2 (Codes for hypertension resolved) QOF cluster

Date of Read code before 01 Apr 2013
Where patient is registered at General Practice

NOT IN

Diabetes Register
ASPIRE Study / 7

Has a Read code in the DRDM1 (Diagnostic codes for diabetes mellitus) QOF cluster
Show read codes in cluster DRDM1.
- Selecting only the most recent matching code
- Without a more recent Read code in the DRDM2 (Codes for diabetes resolved) QOF cluster

Date of Read code before 01 Apr 2013
Current age > 17 years
Where patient is registered at General Practice
