# Supplementary material for: Developing ‘high impact’ guideline-based quality indicators for UK primary care: a multi-stage consensus process
Source: BMC Fam Pract. 2015 Oct 28;16:156. doi: 10.1186/s12875-015-0350-6 (PMC4624600; doi:10.1186/s12875-015-0350-6)
Supplement: Additional file 4 — Folder containing SystmOne™ search algorithms. (ZIP 12.7 mb) [file 12875_2015_350_MOESM4_ESM.zip › Aspire S1 diagrams tw edired/7N5 (CKD #47).pdf]

———	Mandatory In
- - - -	Optional In
....	Not In

7N5. CKD Register and ACR =>70 or PCR =>100 or Urinary protein excretion =>1 and either ACE or ARB-1
ASPIRE Study / 7

Registered before 01 Apr 2013
Where patient is registered at General Practice

IN → **ACE-1 or ARB**
ASPIRE Study / 7

Where patient is registered at General Practice

IN → **BNF 2.5.5.2 (Angiotensin 2)**
ASPIRE Study / 7

Has medication in the 'Angiotensin-II antagonists' Action Group
- Include all drug types

Date of medication between 01 Apr 2012 and 31 Mar 2013
Where patient is registered at General Practice

OR IN → **BNF 2.5.5.1**
ASPIRE Study / 7

Has medication in the 'ACE inhibitors' Action Group
- Include all drug types

Date of medication between 01 Apr 2012 and 31 Mar 2013
Where patient is registered at General Practice

AND IN → **7D5 + 7D6. CKD Register and ACR =>70 or PCR =>100 or Urinary protein excretion =>1 (Excluding Diabetes Reg)**
ASPIRE Study / 7

Registered before 01 Apr 2013
Where patient is registered at General Practice

IN → **CKD Register and ACR =>70 or PCR =>100 or Urinary protein excretion =>1**
ASPIRE Study / 7

Registered before 01 Apr 2013
Where patient is registered at General Practice

IN → **7D1. CKD01 Register**
ASPIRE Study / 7

Has a Read code in the DRCKD1 (Chronic kidney disease codes 3-5) QOF cluster Show read codes in cluster DRCKD1.
- Selecting only the most recent matching code
- Without a more recent Read code in the DRCKD2 (Chronic kidney disease codes 1-2) QOF cluster

Date of Read code before 01 Apr 2013
Registered before 01 Apr 2013
Where patient is registered at General Practice

AND IN → **ACR =>70 OR PCR =>100 OR Urinary protein =>1**
ASPIRE Study / 7

Where patient is registered at General Practice

IN → **ACR =>70**
ASPIRE Study / 7

Most recent Urine albumin/creatinine ratio reading >= 70.0 mg/mmol
- Without a more recent Urine albumin/creatinine ratio reading < 70.0 mg/mmol

Date of numeric reading before 01 Apr 2013
Where patient is registered at General Practice

OR IN → **PCR =>100**
ASPIRE Study / 7

Most recent Urine porphyrin/creatinine ratio reading >= 100.0 nmol/mmol Creatinine

- Without a more recent Urine porphyrin/creatinine ratio reading < 100.0 nmol/mmol Creatinine

Date of numeric reading before 01 Apr 2013

Where patient is registered at General Practice

OR IN

Urine protein level =>1
ASPIRE Study / 7

Most recent Urine protein level reading >= 1.0 g/L

- Without a more recent Urine protein level reading < 1.0 g/L

Date of numeric reading before 01 Apr 2013

Where patient is registered at General Practice

NOT IN

Diabetes Register
ASPIRE Study / 7

Has a Read code in the DRDM1 (Diagnostic codes for diabetes mellitus) QOF cluster
Show read codes in cluster DRDM1.

- Selecting only the most recent matching code
- Without a more recent Read code in the DRDM2 (Codes for diabetes resolved) QOF cluster

Date of Read code before 01 Apr 2013

Current age > 17 years

Where patient is registered at General Practice
