# Supplementary material for: Developing ‘high impact’ guideline-based quality indicators for UK primary care: a multi-stage consensus process
Source: BMC Fam Pract. 2015 Oct 28;16:156. doi: 10.1186/s12875-015-0350-6 (PMC4624600; doi:10.1186/s12875-015-0350-6)
Supplement: Additional file 4 — Folder containing SystmOne™ search algorithms. (ZIP 12.7 mb) [file 12875_2015_350_MOESM4_ESM.zip › Aspire S1 diagrams tw edired/8D2 (Depression #69).pdf]

Mandatory In
Optional In
Not In

8D2. PHQ9 between 1-9 OR Mild depression
ASPIRE Study / 8

Registered before 01 Apr 2013
Where patient is registered at General Practice

IN → **Mild or Depressed mood**
ASPIRE Study / 8

- Has a Read code in...Exact Read Codes:
  Depressed mood (XE0re)
  Mild depression (XaCIs)
- Selecting only the most recent matching code
- Date of Read code between 01 Apr 2012 and 31 Mar 2013
- Where patient is registered at General Practice

OR IN → **PHQ9 between 1-9 - excluding =>10**
ASPIRE Study / 8

- Most recent Patient health questionnaire (PHQ-9) score reading between 1 and 9
- Without a more recent Patient health questionnaire (PHQ-9) score reading >= 10.0
- Date of numeric reading between 01 Apr 2012 and 31 Mar 2013
- Where patient is registered at General Practice
