# Supplementary material for: Developing ‘high impact’ guideline-based quality indicators for UK primary care: a multi-stage consensus process
Source: BMC Fam Pract. 2015 Oct 28;16:156. doi: 10.1186/s12875-015-0350-6 (PMC4624600; doi:10.1186/s12875-015-0350-6)
Supplement: Additional file 4 — Folder containing SystmOne™ search algorithms. (ZIP 12.7 mb) [file 12875_2015_350_MOESM4_ESM.zip › Aspire S1 diagrams tw edired/8N1 (Depression #69).pdf]

——	Mandatory In
- - - -	Optional In
....	Not In

8N1. PHQ9 between 1-14 OR mild / moderate depression and Referral to Mental Health Services
ASPIRE Study / 8

Registered before 01 Apr 2013
Where patient is registered at General Practice

IN

Referral to Mental Health Service
ASPIRE Study / 8

Has a Read code in...Exact Read Codes:
Referral to mental health counselling service (XaAem)
Referral for mental health counselling (XaAen)
Referral to mental health counsellor (XaAfJ)
Referral to counsellor (XaBT1)
Referral to mental health crisis team (XaIkg)
Specialist mental health assessment (XaK6K)
Seen by primary care graduate mental health worker (XaL0t)
Referral to primary care mental health gateway worker (XaLFL)
Seen by primary care mental health gateway worker (XaM7s)
Seen in mental health clinic (XaONq)
Read Codes and Children:
Referral to mental health team (XaIPw)
Referral for cognitive behavioural therapy (XaR5D)
Date of Read code between 01 Apr 2012 and 31 Mar 2013
Where patient is registered at General Practice

AND IN

8D1. PHQ9 between 1-14 OR Mild, moderate depression
ASPIRE Study / 8

Registered before 01 Apr 2013
Where patient is registered at General Practice

IN

Mild or Moderate or Depressed mood
ASPIRE Study / 8

Has a Read code in...Exact Read Codes:
Depressed mood (XE0re)
Mild depression (XaCIs)
Moderate depression (XaCIt)
- Selecting only the most recent matching code

Date of Read code between 01 Apr 2012 and 31 Mar 2013
Where patient is registered at General Practice

OR IN

PHQ9 between 1-14 - excluding =>15
ASPIRE Study / 8

Most recent Patient health questionnaire (PHQ-9) score reading between 1 and 14
- Without a more recent Patient health questionnaire (PHQ-9) score reading >= 15.0

Date of numeric reading between 01 Apr 2012 and 31 Mar 2013
Where patient is registered at General Practice
