# Supplementary material for: Developing ‘high impact’ guideline-based quality indicators for UK primary care: a multi-stage consensus process
Source: BMC Fam Pract. 2015 Oct 28;16:156. doi: 10.1186/s12875-015-0350-6 (PMC4624600; doi:10.1186/s12875-015-0350-6)
Supplement: Additional file 4 — Folder containing SystmOne™ search algorithms. (ZIP 12.7 mb) [file 12875_2015_350_MOESM4_ESM.zip › Aspire S1 diagrams tw edired/8N2 (Depression #69).pdf]

Mandatory In
Optional In
Not In

8N2. PHQ9 between 1-9 OR mild depression with BNF chapter 4.3 (excluding amitriptyline)
ASPIRE Study / 8

Registered before 01 Apr 2013
Where patient is registered at General Practice

- IN → **8D2. PHQ9 between 1-9 OR Mild depression**
  ASPIRE Study / 8

  Registered before 01 Apr 2013
  Where patient is registered at General Practice

  - IN → **Mild or Depressed mood**
    ASPIRE Study / 8

    Has a Read code in...Exact Read Codes:
    Depressed mood (XE0re)
    Mild depression (XaCIs)
    - Selecting only the most recent matching code

    Date of Read code between 01 Apr 2012 and 31 Mar 2013
    Where patient is registered at General Practice

  - OR IN → **PHQ9 between 1-9 - excluding =>10**
    ASPIRE Study / 8

    Most recent Patient health questionnaire (PHQ-9) score reading between 1 and 9
    - Without a more recent Patient health questionnaire (PHQ-9) score reading >= 10.0

    Date of numeric reading between 01 Apr 2012 and 31 Mar 2013
    Where patient is registered at General Practice

- AND IN → **BNF Chapter 4.3 (Excluding Amitriptyline)**
  ASPIRE Study / 8

  Where patient is registered at General Practice

  - NOT IN → **Amitriptyline**
    ASPIRE Study / 8

    Has an issue of...Drugs:
    AMITRIPTYLINE (Generic Manuf) (form not specified)
    Amitriptyline 10mg tablets
    Amitriptyline 10mg tablets (A A H Pharmaceuticals Ltd)
    Amitriptyline 10mg tablets (Actavis UK Ltd)
    Amitriptyline 10mg tablets (Almus Pharmaceuticals Ltd)
    Amitriptyline 10mg tablets (IVAX Pharmaceuticals UK Ltd)
    Amitriptyline 10mg tablets (Kent Pharmaceuticals Ltd)
    Amitriptyline 10mg tablets (Teva UK Ltd)
    Amitriptyline 10mg tablets (Wockhardt UK Ltd)
    Amitriptyline 25mg tablets
    Amitriptyline 25mg tablets (A A H Pharmaceuticals Ltd)
    Amitriptyline 25mg tablets (Actavis UK Ltd)
    Amitriptyline 25mg tablets (Almus Pharmaceuticals Ltd)
    Amitriptyline 25mg tablets (IVAX Pharmaceuticals UK Ltd)
    Amitriptyline 25mg tablets (Kent Pharmaceuticals Ltd)
    Amitriptyline 25mg tablets (Ranbaxy (UK) Ltd)
    Amitriptyline 25mg tablets (Sandoz Ltd)
    Amitriptyline 25mg tablets (Teva UK Ltd)
    Amitriptyline 25mg tablets (Wockhardt UK Ltd)
    Amitriptyline 25mg/5ml oral solution sugar free
    Amitriptyline 25mg/5ml oral solution sugar free (A A H Pharmaceuticals Ltd)
    Amitriptyline 25mg/5ml oral solution sugar free (Rosemont Pharmaceuticals Ltd)
    Amitriptyline 25mg/5ml oral solution sugar free (Wockhardt UK Ltd)
    Amitriptyline 50mg tablets
    Amitriptyline 50mg tablets (A A H

Amitriptyline 50mg tablets (A A H Pharmaceuticals Ltd)
Amitriptyline 50mg tablets (Actavis UK Ltd)
Amitriptyline 50mg tablets (Almus Pharmaceuticals Ltd)
Amitriptyline 50mg tablets (IVAX Pharmaceuticals UK Ltd)
Amitriptyline 50mg tablets (Kent Pharmaceuticals Ltd)
Amitriptyline 50mg tablets (Teva UK Ltd)
Amitriptyline 50mg tablets (Wockhardt UK Ltd)
Amitriptyline 50mg/5ml oral solution sugar free
Amitriptyline 50mg/5ml oral solution sugar free (A A H Pharmaceuticals Ltd)
Amitriptyline 50mg/5ml oral solution sugar free (Rosemont Pharmaceuticals Ltd)
Amitriptyline 50mg/5ml oral solution sugar free (Wockhardt UK Ltd)
amitriptyline hydrochloride (form not specified)
amitriptyline hydrochloride oral solution sugar-free 10mg/5ml
AMITRIPTYLINE oral solution sugar-free 10mg/5ml [ROSEMONT]
AMITRIPTYLINE tablets 10mg [BERK]
AMITRIPTYLINE tablets 10mg [CELLTECH]
AMITRIPTYLINE tablets 10mg [NUMARK]
AMITRIPTYLINE tablets 10mg [SUSSEX]
AMITRIPTYLINE tablets 25mg [BERK]
AMITRIPTYLINE tablets 25mg [CELLTECH]
AMITRIPTYLINE tablets 25mg [CROSS-PHAR]
AMITRIPTYLINE tablets 25mg [REGENT]
AMITRIPTYLINE tablets 25mg [SCD]
AMITRIPTYLINE tablets 25mg [SUSSEX]
AMITRIPTYLINE tablets 50mg [BERK]
AMITRIPTYLINE tablets 50mg [CELLTECH]

- Include all drug types
- Date of medication between 01 Apr 2012 and 31 Mar 2013
- Where patient is registered at General Practice

AND IN

BNF Chapter 4.3
ASPIRE Study / 8

- Has medication in the 'Antidepressants' Action Group
- Include all drug types
- Date of medication between 01 Apr 2012 and 31 Mar 2013
- Where patient is registered at General Practice
