# Supplementary material for: Developing ‘high impact’ guideline-based quality indicators for UK primary care: a multi-stage consensus process
Source: BMC Fam Pract. 2015 Oct 28;16:156. doi: 10.1186/s12875-015-0350-6 (PMC4624600; doi:10.1186/s12875-015-0350-6)
Supplement: Additional file 4 — Folder containing SystmOne™ search algorithms. (ZIP 12.7 mb) [file 12875_2015_350_MOESM4_ESM.zip › Aspire S1 diagrams tw edired/8N3 (Depression #69).pdf]

Mandatory In
Optional In
Not In

8N3. PHQ9 between 10-27 OR Moderate / depressed moor or sever depression AND BNF 4.3 (excluding Amitriptyine) and Referral to Mental Health
ASPIRE Study / 8

Registered before 01 Apr 2013
Where patient is registered at General Practice

IN

8D3. PHQ9 between 10-27 OR Moderate or Severe depression
ASPIRE Study / 8

Registered before 01 Apr 2013
Where patient is registered at General Practice

IN

Moderate or Severe depression
ASPIRE Study / 8

Has a Read code in...Exact Read Codes:
Major depressive disorder (XSEGJ)
Moderate major depression (XSGol)
Moderate depression (XaClt)
Severe depression (XaClu)

- Selecting only the most recent matching code

Date of Read code between 01 Apr 2012 and 31 Mar 2013
Where patient is registered at General Practice

OR IN

PHQ9 between 10-27 - excluding =<9
ASPIRE Study / 8

Most recent Patient health questionnaire (PHQ-9) score reading between 10 and 27

- Without a more recent Patient health questionnaire (PHQ-9) score reading <= 9.0

Date of numeric reading between 01 Apr 2012 and 31 Mar 2013
Where patient is registered at General Practice

AND IN

BNF 4.3 (Excluding Amitriptyline) and Referral to Mental Health
ASPIRE Study / 8

Where patient is registered at General Practice

IN

BNF Chapter 4.3 (Excluding Amitriptyline)
ASPIRE Study / 8

Where patient is registered at General Practice

NOT IN

Amitriptyline
ASPIRE Study / 8

Has an issue of...Drugs:
AMITRIPTYLINE (Generic Manuf) (form not specified)
Amitriptyline 10mg tablets
Amitriptyline 10mg tablets (A A H Pharmaceuticals Ltd)
Amitriptyline 10mg tablets (Actavis UK Ltd)
Amitriptyline 10mg tablets (Almus Pharmaceuticals Ltd)
Amitriptyline 10mg tablets (IVAX Pharmaceuticals UK Ltd)
Amitriptyline 10mg tablets (Kent Pharmaceuticals Ltd)
Amitriptyline 10mg tablets (Teva UK Ltd)
Amitriptyline 10mg tablets (Wockhardt UK Ltd)
Amitriptyline 25mg tablets
Amitriptyline 25mg tablets (A A H Pharmaceuticals Ltd)
Amitriptyline 25mg tablets (Actavis UK Ltd)
Amitriptyline 25mg tablets (Almus Pharmaceuticals Ltd)
Amitriptyline 25mg tablets (IVAX Pharmaceuticals UK Ltd)
Amitriptyline 25mg tablets (Kent Pharmaceuticals Ltd)
Amitriptyline 25mg tablets (Ranbaxy (UK) Ltd)
Amitriptyline 25mg tablets (Sandoz Ltd)
Amitriptyline 25mg tablets (Teva UK Ltd)
Amitriptyline 25mg tablets (Wockhardt UK Ltd)
[illegible]

Amitriptyline 25mg/5ml oral solution sugar free
Amitriptyline 25mg/5ml oral solution sugar free (A A H Pharmaceuticals Ltd)
Amitriptyline 25mg/5ml oral solution sugar free (Rosemont Pharmaceuticals Ltd)
Amitriptyline 25mg/5ml oral solution sugar free (Wockhardt UK Ltd)
Amitriptyline 50mg tablets
Amitriptyline 50mg tablets (A A H Pharmaceuticals Ltd)
Amitriptyline 50mg tablets (Actavis UK Ltd)
Amitriptyline 50mg tablets (Almus Pharmaceuticals Ltd)
Amitriptyline 50mg tablets (IVAX Pharmaceuticals UK Ltd)
Amitriptyline 50mg tablets (Kent Pharmaceuticals Ltd)
Amitriptyline 50mg tablets (Teva UK Ltd)
Amitriptyline 50mg tablets (Wockhardt UK Ltd)
Amitriptyline 50mg/5ml oral solution sugar free
Amitriptyline 50mg/5ml oral solution sugar free (A A H Pharmaceuticals Ltd)
Amitriptyline 50mg/5ml oral solution sugar free (Rosemont Pharmaceuticals Ltd)
Amitriptyline 50mg/5ml oral solution sugar free (Wockhardt UK Ltd)
amitriptyline hydrochloride (form not specified)
amitriptyline hydrochloride oral solution sugar-free 10mg/5ml
AMITRIPTYLINE oral solution sugar-free 10mg/5ml [ROSEMONT]
AMITRIPTYLINE tablets 10mg [BERK]
AMITRIPTYLINE tablets 10mg [CELLTECH]
AMITRIPTYLINE tablets 10mg [NUMARK]
AMITRIPTYLINE tablets 10mg [SUSSEX]
AMITRIPTYLINE tablets 25mg [BERK]
AMITRIPTYLINE tablets 25mg [CELLTECH]
AMITRIPTYLINE tablets 25mg [CROSS-PHAR]
AMITRIPTYLINE tablets 25mg [REGENT]
AMITRIPTYLINE tablets 25mg [SCD]
AMITRIPTYLINE tablets 25mg [SUSSEX]
AMITRIPTYLINE tablets 50mg [BERK]
AMITRIPTYLINE tablets 50mg [CELLTECH]

- Include all drug types
- Date of medication between 01 Apr 2012 and 31 Mar 2013
- Where patient is registered at General Practice

AND IN

BNF Chapter 4.3
ASPIRE Study / 8

- Has medication in the 'Antidepressants' Action Group
- Include all drug types
- Date of medication between 01 Apr 2012 and 31 Mar 2013
- Where patient is registered at General Practice

AND IN

Referral to Mental Health Service
ASPIRE Study / 8

Has a Read code in...Exact Read Codes:
Referral to mental health counselling service (XaAem)
Referral for mental health counselling (XaAen)
Referral to mental health counsellor (XaAfJ)
Referral to counsellor (XaBT1)
Referral to mental health crisis team (XaIkg)
Specialist mental health assessment (XaK6K)
Seen by primary care graduate mental health worker (XaL0t)
Referral to primary care mental health gateway worker (XaLFL)
Seen by primary care mental health gateway worker (XaM7s)
Seen in mental health clinic (XaONq)
Read Codes and Children:
Referral to mental health team (XaIPw)
Referral for cognitive behavioural therapy (XaR5D)

- Date of Read code between 01 Apr 2012 and 31 Mar 2013
- Where patient is registered at General Practice
