# Supplementary material for: Developing ‘high impact’ guideline-based quality indicators for UK primary care: a multi-stage consensus process
Source: BMC Fam Pract. 2015 Oct 28;16:156. doi: 10.1186/s12875-015-0350-6 (PMC4624600; doi:10.1186/s12875-015-0350-6)
Supplement: Additional file 4 — Folder containing SystmOne™ search algorithms. (ZIP 12.7 mb) [file 12875_2015_350_MOESM4_ESM.zip › Aspire S1 diagrams tw edired/9D3+5+7+9 (HTN monitoring #79).pdf]

9D3-5, 7, 9. Hypertension Register (upto 1.4.13)
ASPIRE Study / 9

Has a Read code in the DRHYP1 (Hypertension diagnosis codes) QOF cluster
Show read codes in cluster DRHYP1.

- Selecting only the most recent matching code
- Without a more recent Read code in the DRHYP2 (Codes for hypertension resolved) QOF cluster

Date of Read code before 01 Apr 2013
Registered before 01 Apr 2013

——	Mandatory In
- - - -	Optional In
....	Not In
