# Supplementary material for: Developing ‘high impact’ guideline-based quality indicators for UK primary care: a multi-stage consensus process
Source: BMC Fam Pract. 2015 Oct 28;16:156. doi: 10.1186/s12875-015-0350-6 (PMC4624600; doi:10.1186/s12875-015-0350-6)
Supplement: Additional file 4 — Folder containing SystmOne™ search algorithms. (ZIP 12.7 mb) [file 12875_2015_350_MOESM4_ESM.zip › Aspire S1 diagrams tw edired/9N4 (HTN monitoring #79).pdf]

——	Mandatory In
- - - -	Optional In
....	Not In

9N4. Hypertension Register AND BMI AND Dietary History AND Lifestyle Counselling
ASPIRE Study / 9

Registered before 01 Apr 2013
Where patient is registered at General Practice

IN

- **BMI, Dietary History and lifestyle counselling**
  ASPIRE Study / 9
  Where patient is registered at General Practice

  IN
  - **Lifestyle Advice or Exercise Advice**
    ASPIRE Study / 9
    Where patient is registered at General Practice

    IN
    - **Lifestyle Advice**
      ASPIRE Study / 9
      Has a Read code in...Exact Read Codes:
      Lifestyle counselling (XaEFY)
      Lifestyle advice regarding hypertension (XaQaV)
      Date of Read code between 01 Apr 2012 and 31 Mar 2013

    OR IN
    - **Exercise advice**
      ASPIRE Study / 9
      Has a Read code in...Exact Read Codes:
      Lifestyle advice regarding exercise (XaJIt)
      Education : Exercise (Y0305)
      Read Codes and Children:
      Advice about exercise (Xa9zF)
      Advice to undertake functional activity (Xa9zR)
      Excluding Exact Read Codes:
      Pelvic floor exercise advice given (XaNg2)
      Selecting only the most recent matching code
      Date of Read code between 01 Apr 2012 and 31 Mar 2013
      Where patient is registered at General Practice

  AND IN
  - **Dietary History or Exercise Advice**
    ASPIRE Study / 9
    Where patient is registered at General Practice

    IN
    - **Exercise advice**
      ASPIRE Study / 9
      Has a Read code in...Exact Read Codes:
      Lifestyle advice regarding exercise (XaJIt)
      Education : Exercise (Y0305)
      Read Codes and Children:
      Advice about exercise (Xa9zF)
      Advice to undertake functional activity (Xa9zR)
      Excluding Exact Read Codes:
      Pelvic floor exercise advice given (XaNg2)
      Selecting only the most recent matching code
      Date of Read code between 01 Apr 2012 and 31 Mar 2013
      Where patient is registered at General Practice

    OR IN
    - **Dietary History**
      ASPIRE Study / 9
      Has a Read code in...Read Codes and Children:
      Dietary history (1F...)
      Diets (Ub01y)
      Exercise history (XE0os)
      Date of Read code between 01 Apr 2012 and 31 Mar 2013
      Where patient is registered at General Practice

  AND IN
  - **BMI**
    ASPIRE Study / 9
    Has a BMI

Date of numeric reading between 01 Apr 2012 and 31 Mar 2013

Registered before 01 Apr 2013

AND IN

9D3-5, 7, 9. Hypertension Register (upto 1.4.13)

ASPIRE Study / 9

Has a Read code in the DRHYP1 (Hypertension diagnosis codes) QOF cluster
Show read codes in cluster DRHYP1.

- Selecting only the most recent matching code
- Without a more recent Read code in the DRHYP2 (Codes for hypertension resolved) QOF cluster

Date of Read code before 01 Apr 2013

Registered before 01 Apr 2013
