# Supplementary material for: Developing ‘high impact’ guideline-based quality indicators for UK primary care: a multi-stage consensus process
Source: BMC Fam Pract. 2015 Oct 28;16:156. doi: 10.1186/s12875-015-0350-6 (PMC4624600; doi:10.1186/s12875-015-0350-6)
Supplement: Additional file 4 — Folder containing SystmOne™ search algorithms. (ZIP 12.7 mb) [file 12875_2015_350_MOESM4_ESM.zip › Aspire S1 diagrams tw edired/9N5 (HTN monitoring #79).pdf]

____	Mandatory In
- - - -	Optional In
....	Not In

9N5. Hypertension Register with Alcohol intake recorded
ASPIRE Study / 9

Registered before 01 Apr 2013
Where patient is registered at General Practice

IN →

9D3-5, 7, 9. Hypertension Register (upto 1.4.13)
ASPIRE Study / 9

Has a Read code in the DRHYP1 (Hypertension diagnosis codes) QOF cluster
Show read codes in cluster DRHYP1.

- Selecting only the most recent matching code
- Without a more recent Read code in the DRHYP2 (Codes for hypertension resolved) QOF cluster

Date of Read code before 01 Apr 2013
Registered before 01 Apr 2013

AND IN →

ALC QoF Cluster
ASPIRE Study / 9

Has a Read code in the ALC (Alcohol consumption codes) QOF cluster
Show read codes in cluster ALC.
Date of Read code between 01 Apr 2012 and 31 Mar 2013
Where patient is registered at General Practice
