# Supplementary material for: Developing ‘high impact’ guideline-based quality indicators for UK primary care: a multi-stage consensus process
Source: BMC Fam Pract. 2015 Oct 28;16:156. doi: 10.1186/s12875-015-0350-6 (PMC4624600; doi:10.1186/s12875-015-0350-6)
Supplement: Additional file 4 — Folder containing SystmOne™ search algorithms. (ZIP 12.7 mb) [file 12875_2015_350_MOESM4_ESM.zip › Aspire S1 diagrams tw edired/9N8 (HTN monitoring #79).pdf]

| | |
|---|---|
| —— | Mandatory In |
| - - - - | Optional In |
| .......... | Not In |

**9N8. Numerator 1+2**
ASPIRE Study / 9
Registered before 01 Apr 2013
Where patient is registered at General Practice

- IN → **9N2. Hypertension and QRisk 2**
  ASPIRE Study / 9
  Registered before 01 Apr 2013
  Where patient is registered at General Practice
  - IN → **CVDASS Coding**
    ASPIRE Study / 9
    Has a Read code in the CVDASS (Cardio Vascular Risk Assessment codes) QOF cluster
    Show read codes in cluster CVDASS.
    Date of Read code between 01 Apr 2012 and 31 Mar 2013
  - AND IN → **9D1,2 + 8. Hypertension Register (qof year 12/13)**
    ASPIRE Study / 9
    Has a Read code in the DRHYP1 (Hypertension diagnosis codes) QOF cluster
    Show read codes in cluster DRHYP1.
    - Selecting only the most recent matching code
    - Without a more recent Read code in the DRHYP2 (Codes for hypertension resolved) QOF cluster

    Date of Read code between 01 Apr 2012 and 31 Mar 2013
    Registered before 01 Apr 2013
- AND IN → **9N1. Hypertension register and N1.1 or N1.2**
  ASPIRE Study / 9
  Registered before 01 Apr 2013
  Where patient is registered at General Practice
  - IN → **N1.1 OR N1.2 (NO ECG)**
    ASPIRE Study / 9
    Where patient is registered at General Practice
    - IN (optional) → **N1.1. HbA1c and Urine Albumin/ Creating ratio and Urine dipstick test**
      ASPIRE Study / 9
      Where patient is registered at General Practice
      - IN → **Urine dipstick test**
        ASPIRE Study / 9
        Has a Read code of Urine dipstick test (4618.) or one of its children
        Date of Read code between 01 Apr 2012 and 31 Mar 2013
      - AND IN → **Urine Albumin/ Creatinine ratio**
        ASPIRE Study / 9
        Has a Urine albumin/creatinine ratio
        Date of numeric reading between 01 Apr 2012 and 31 Mar 2013
      - AND IN → **HbA1c**
        ASPIRE Study / 9
        Has a Read code in the HBA (HbA1c codes) QOF cluster
        Show read codes in cluster HBA.
        Date of Read code between 01 Apr 2012 and 31 Mar 2013
    - OR IN (optional) → **N1.2. Plasma glucose level and serum electrolyte level and eGFR and serum cholesterol level and serum HDL cholesterol level NO ECG**
      ASPIRE Study / 9
      Where patient is registered at General Practice

Practice

IN

**Plasma glucose level**
ASPIRE Study / 9

Has a Plasma glucose level
Date of numeric reading between 01 Apr 2012 and 31 Mar 2013

AND IN

**Serum electrolyte level**
ASPIRE Study / 9

Has a Read code of Serum electrolyte levels (XE2mj)
Date of Read code between 01 Apr 2012 and 31 Mar 2013

AND IN

**eGFR cluster**
ASPIRE Study / 9

Has a Read code in the EGFR (Codes for estimated glomerular filtration rate) QOF cluster Show read codes in cluster EGFR.
Date of Read code between 01 Apr 2012 and 31 Mar 2013

AND IN

**Serum cholesterol level**
ASPIRE Study / 9

Has a Serum cholesterol level
Date of numeric reading between 01 Apr 2012 and 31 Mar 2013

AND IN

**Serum HDL cholesterol level**
ASPIRE Study / 9

Has a Serum HDL cholesterol level
Date of numeric reading between 01 Apr 2012 and 31 Mar 2013

AND IN

**9D1,2 + 8. Hypertension Register (qof year 12/13)**
ASPIRE Study / 9

Has a Read code in the DRHYP1 (Hypertension diagnosis codes) QOF cluster Show read codes in cluster DRHYP1.

- Selecting only the most recent matching code
- Without a more recent Read code in the DRHYP2 (Codes for hypertension resolved) QOF cluster

Date of Read code between 01 Apr 2012 and 31 Mar 2013
Registered before 01 Apr 2013
