# Supplementary material for: Developing ‘high impact’ guideline-based quality indicators for UK primary care: a multi-stage consensus process
Source: BMC Fam Pract. 2015 Oct 28;16:156. doi: 10.1186/s12875-015-0350-6 (PMC4624600; doi:10.1186/s12875-015-0350-6)
Supplement: Additional file 4 — Folder containing SystmOne™ search algorithms. (ZIP 12.7 mb) [file 12875_2015_350_MOESM4_ESM.zip › Aspire S1 diagrams tw edired/9N9 (HTN monitoring #79).pdf]

Mandatory In
Optional In
Not In

9N9. Numerators 3,4,5,7
ASPIRE Study / 9

Registered before 01 Apr 2013
Where patient is registered at General Practice

- IN → **9N7. Hypertension Register and Hypertension Review**
  ASPIRE Study / 9

  Registered before 01 Apr 2013
  Where patient is registered at General Practice

  - IN → **BP reading and Hypertension review**
    ASPIRE Study / 9

    Where patient is registered at General Practice

    - IN → **Hypertension Review**
      ASPIRE Study / 9

      Has a Read code in...Exact Read Codes:
      Lifestyle counselling (XaEFY)
      Hypertension six month review (XaIyD)
      Hypertension annual review (XaIyE)
      Lifestyle advice regarding hypertension (XaQaV)
      Hypertension 9 month review (XaXOi)
      Date of Read code between 01 Apr 2012 and 31 Mar 2013
      Registered before 01 Apr 2013

    - AND IN → **BP Exists in the last 15 months**
      ASPIRE Study / 9

      Where patient is registered at General Practice

      - IN → **BP exists (>30/30) in the last 15 months**
        ASPIRE Study / 9

        Has a BP reading > 30 / 30
        Date of BP reading between 01 Jan 2012 and 31 Mar 2013
        Where patient is registered at General Practice

      - OR IN → **BP Systolic and Diastolic valid in the last 15 months**
        ASPIRE Study / 9

        Where patient is registered at General Practice

        - IN → **BP Systolic =>30 in the last 15 months**
          ASPIRE Study / 9

          Has numeric reading in the BPSYS (BP systolic codes) nGMS cluster >= 30.0
          Date of numeric reading between 01 Jan 2012 and 31 Mar 2013
          Where patient is registered at General Practice

        - AND IN → **BP Diastolic =>30 in the last 15 months**
          ASPIRE Study / 9

          Has numeric reading in the BPDIA (BP diastolic codes) nGMS cluster >= 30.0
          Date of numeric reading between 01 Jan 2012 and 31 Mar 2013
          Where patient is registered at General Practice

  - AND IN → **9D3-5, 7, 9. Hypertension Register (upto 1.4.13)**
    ASPIRE Study / 9

    Has a Read code in the DRHYP1 (Hypertension diagnosis codes) QOF cluster
    Show read codes in cluster DRHYP1.
    - Selecting only the most recent matching code
    - Without a more recent Read code in the DRHYP2 (Codes for hypertension resolved) QOF cluster

    Date of Read code before 01 Apr 2013
    Registered before 01 Apr 2013

AND IN

9N5. Hypertension Register with Alcohol intake recorded
ASPIRE Study / 9

Registered before 01 Apr 2013
Where patient is registered at General Practice

IN

9D3-5, 7, 9. Hypertension Register (upto 1.4.13)
ASPIRE Study / 9

Has a Read code in the DRHYP1 (Hypertension diagnosis codes) QOF cluster
Show read codes in cluster DRHYP1.

- Selecting only the most recent matching code
- Without a more recent Read code in the DRHYP2 (Codes for hypertension resolved) QOF cluster

Date of Read code before 01 Apr 2013
Registered before 01 Apr 2013

AND IN

ALC QoF Cluster
ASPIRE Study / 9

Has a Read code in the ALC (Alcohol consumption codes) QOF cluster
Show read codes in cluster ALC.
Date of Read code between 01 Apr 2012 and 31 Mar 2013
Where patient is registered at General Practice

AND IN

9N4. Hypertension Register AND BMI AND Dietary History AND Lifestyle Counselling
ASPIRE Study / 9

Registered before 01 Apr 2013
Where patient is registered at General Practice

IN

BMI, Dietary History and lifestyle counselling
ASPIRE Study / 9

Where patient is registered at General Practice

IN

Lifestyle Advice or Exercise Advice
ASPIRE Study / 9

Where patient is registered at General Practice

IN

Lifestyle Advice
ASPIRE Study / 9

Has a Read code in...Exact Read Codes:
Lifestyle counselling (XaEFY)
Lifestyle advice regarding hypertension (XaQaV)
Date of Read code between 01 Apr 2012 and 31 Mar 2013

OR IN

Exercise advice
ASPIRE Study / 9

Has a Read code in...Exact Read Codes:
Lifestyle advice regarding exercise (XaJIt)
Education : Exercise (Y0305)
Read Codes and Children:
Advice about exercise (Xa9zF)
Advice to undertake functional activity (Xa9zR)
Excluding Exact Read Codes:
Pelvic floor exercise advice given (XaNg2)

- Selecting only the most recent matching code

Date of Read code between 01 Apr 2012 and 31 Mar 2013
Where patient is registered at General Practice

AND IN

Dietary History or Exercise Advice
ASPIRE Study / 9

Where patient is registered at General Practice

IN

Exercise advice
ASPIRE Study / 9

Has a Read code in...Exact Read Codes:
Lifestyle advice regarding exercise (XaJIt)
Education : Exercise (Y0305)
Read Codes and Children:
Advice about exercise (Xa9zF)
Advice to undertake functional activity (Xa9zR)
Excluding Exact Read Codes:
Pelvic floor exercise advice given (XaNg2)

- Selecting only the most recent matching code

Date of Read code between 01 Apr 2012 and 31 Mar 2013
Where patient is registered at General Practice

OR IN

Dietary History
ASPIRE Study / 9

Has a Read code in...Read Codes and Children:
Dietary history (1F...)
Diets (Ub01y)
Exercise history (XE0os)
Date of Read code between 01 Apr 2012 and 31 Mar 2013
Where patient is registered at General Practice

AND IN

BMI
ASPIRE Study / 9

Has a BMI
Date of numeric reading between 01 Apr 2012 and 31 Mar 2013
Registered before 01 Apr 2013

AND IN

9D3-5, 7, 9. Hypertension Register (upto 1.4.13)
ASPIRE Study / 9

Has a Read code in the DRHYP1 (Hypertension diagnosis codes) QOF cluster
Show read codes in cluster DRHYP1.

- Selecting only the most recent matching code
- Without a more recent Read code in the DRHYP2 (Codes for hypertension resolved) QOF cluster

Date of Read code before 01 Apr 2013
Registered before 01 Apr 2013

AND IN

9N3. Hypertension and Lifestyle advice
ASPIRE Study / 9

Registered before 01 Apr 2013
Where patient is registered at General Practice

IN

Lifestyle Advice
ASPIRE Study / 9

Has a Read code in...Exact Read Codes:
Lifestyle counselling (XaEFY)
Lifestyle advice regarding hypertension (XaQaV)
Date of Read code between 01 Apr 2012 and 31 Mar 2013

AND IN

9D3-5, 7, 9. Hypertension Register (upto 1.4.13)
ASPIRE Study / 9

Has a Read code in the DRHYP1 (Hypertension diagnosis codes) QOF cluster
Show read codes in cluster DRHYP1.

- Selecting only the most recent matching code
- Without a more recent Read code in the DRHYP2 (Codes for hypertension resolved) QOF cluster

Date of Read code before 01 Apr 2013
Registered before 01 Apr 2013
