# Supplementary figures and images for: Developing ‘high impact’ guideline-based quality indicators for UK primary care: a multi-stage consensus process
Source: BMC Fam Pract. 2015 Oct 28;16:156. doi: 10.1186/s12875-015-0350-6 (PMC4624600; doi:10.1186/s12875-015-0350-6)

|       |              |
|-------|--------------|
| ————  | Mandatory In |
| ----- | Optional In  |
| ..... | Not In       |

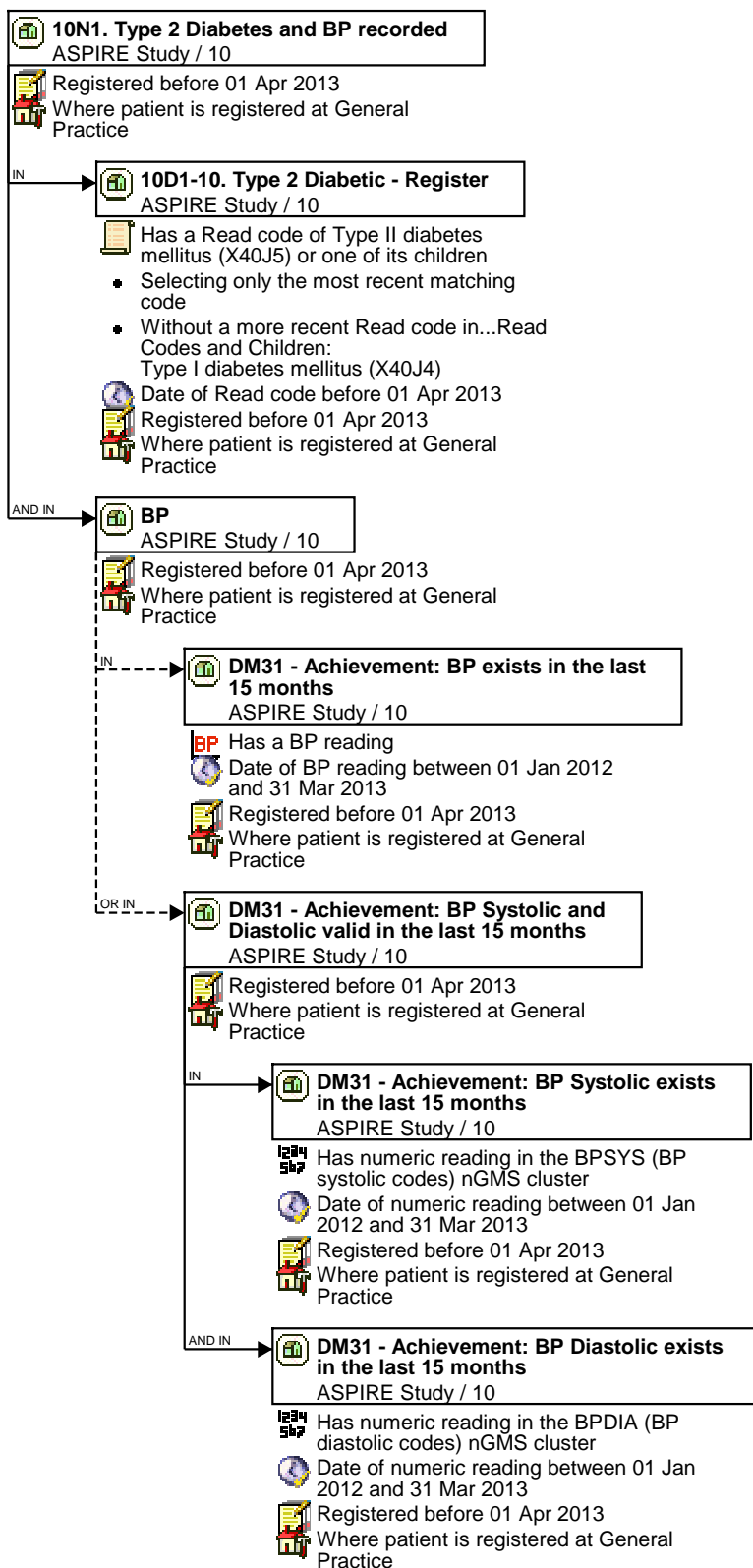

Supplement: Additional file 4 — Folder containing SystmOne™ search algorithms. (ZIP 12.7 mb) [file 12875_2015_350_MOESM4_ESM.zip › Aspire S1 diagrams tw edired/10N1 (DM processes #71).pdf]

|       |              |
|-------|--------------|
| ——    | Mandatory In |
| ----  | Optional In  |
| ..... | Not In       |

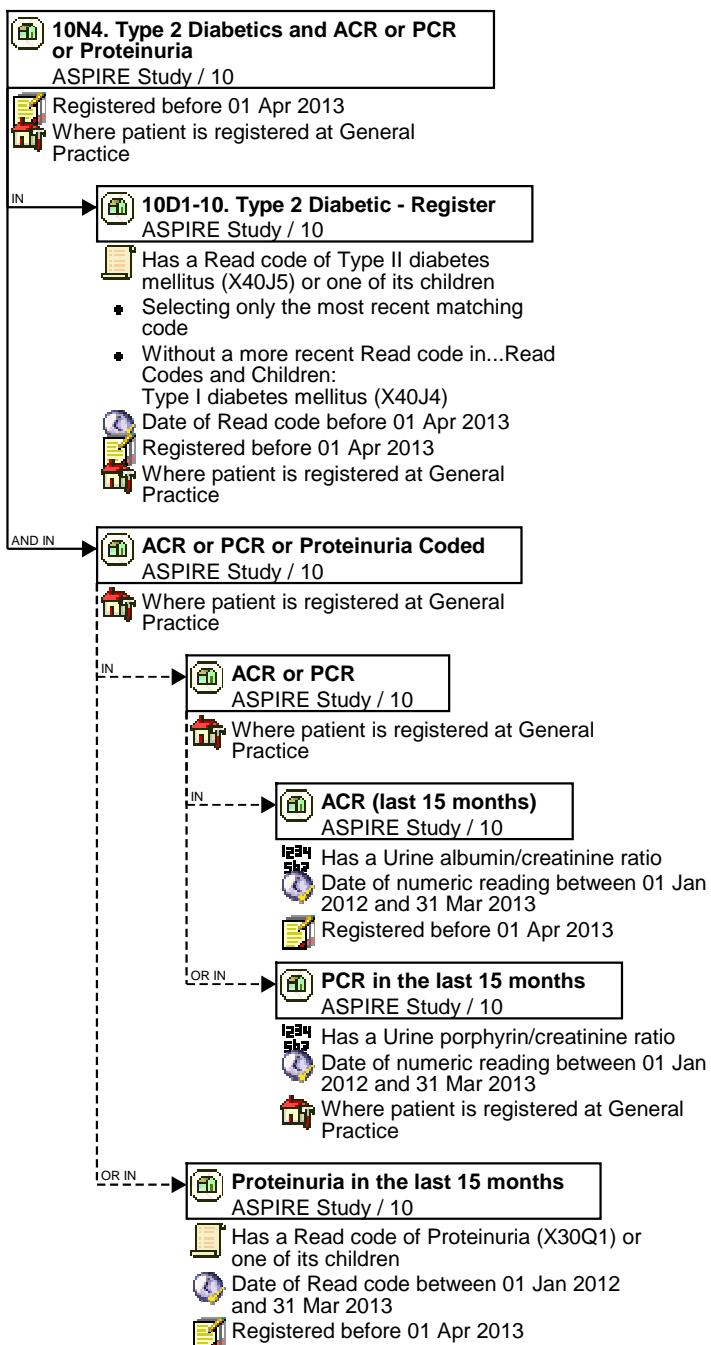

Supplement: Additional file 4 — Folder containing SystmOne™ search algorithms. (ZIP 12.7 mb) [file 12875_2015_350_MOESM4_ESM.zip › Aspire S1 diagrams tw edired/10N4 (DM processes #71).pdf]

|       |              |
|-------|--------------|
| ————  | Mandatory In |
| ----- | Optional In  |
| ..... | Not In       |

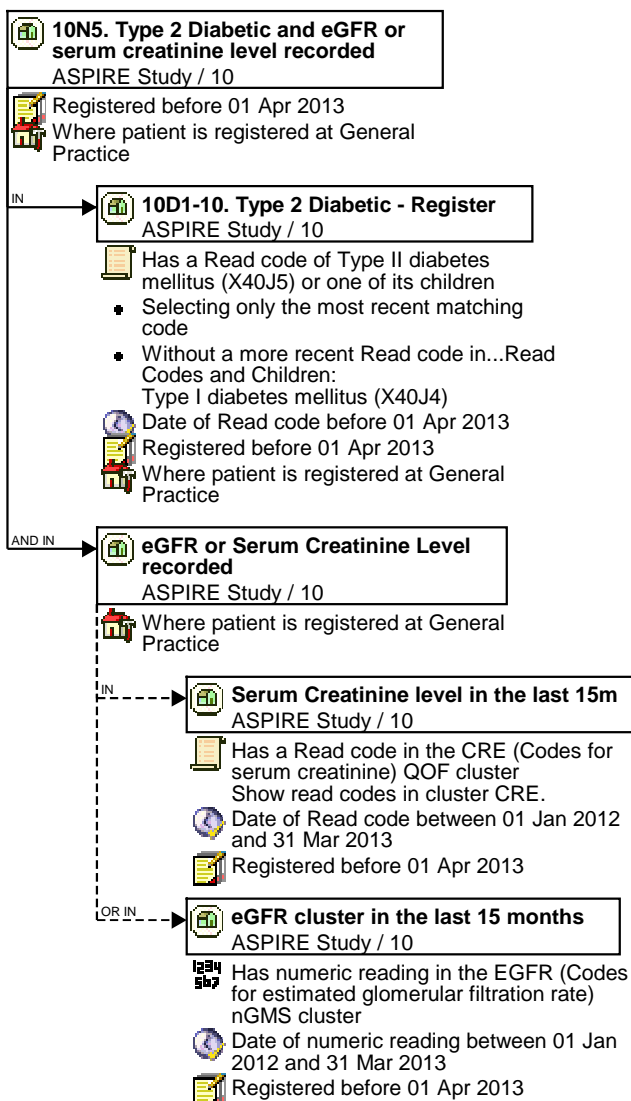

Supplement: Additional file 4 — Folder containing SystmOne™ search algorithms. (ZIP 12.7 mb) [file 12875_2015_350_MOESM4_ESM.zip › Aspire S1 diagrams tw edired/10N5 (DM processes #71).pdf]

|       |              |
|-------|--------------|
| ————  | Mandatory In |
| ----- | Optional In  |
| ..... | Not In       |

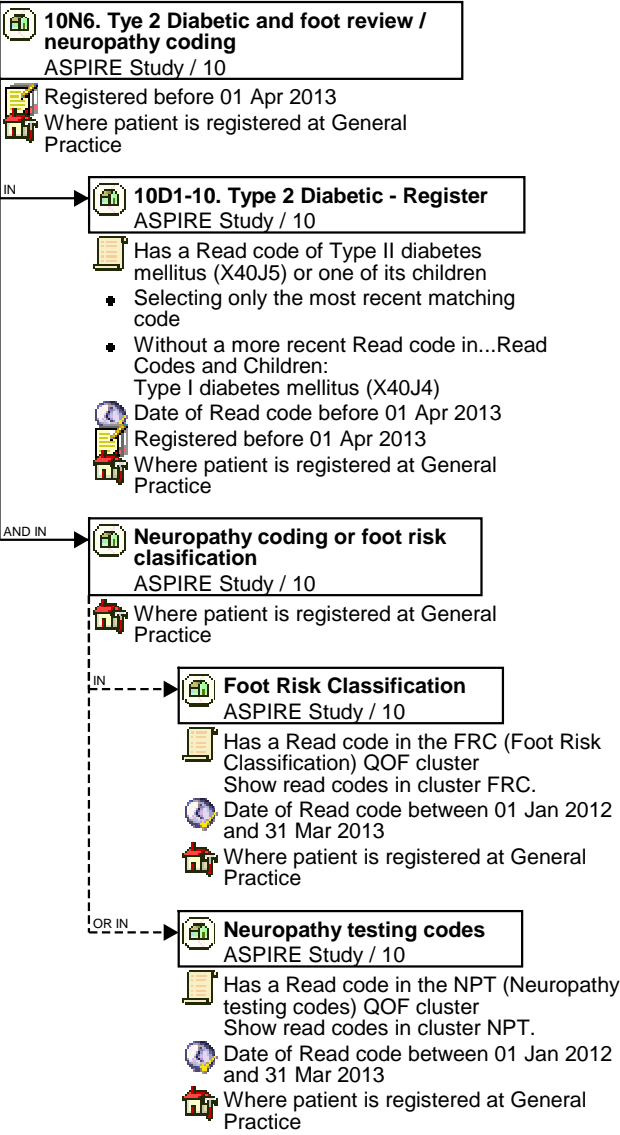

Supplement: Additional file 4 — Folder containing SystmOne™ search algorithms. (ZIP 12.7 mb) [file 12875_2015_350_MOESM4_ESM.zip › Aspire S1 diagrams tw edired/10N6 (DM processes #71).pdf]

|       |              |
|-------|--------------|
| ——    | Mandatory In |
| ----  | Optional In  |
| ..... | Not In       |

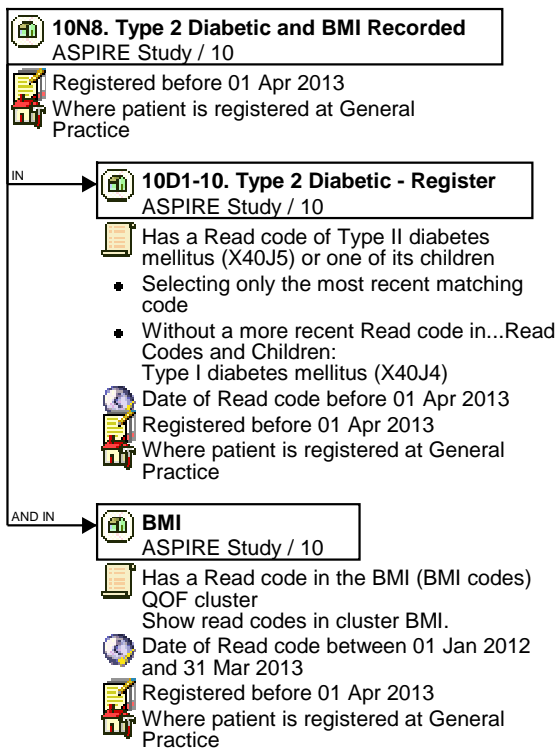

Supplement: Additional file 4 — Folder containing SystmOne™ search algorithms. (ZIP 12.7 mb) [file 12875_2015_350_MOESM4_ESM.zip › Aspire S1 diagrams tw edired/10N8 (DM processes #71).pdf]

|       |              |
|-------|--------------|
| ————  | Mandatory In |
| ----- | Optional In  |
| ..... | Not In       |

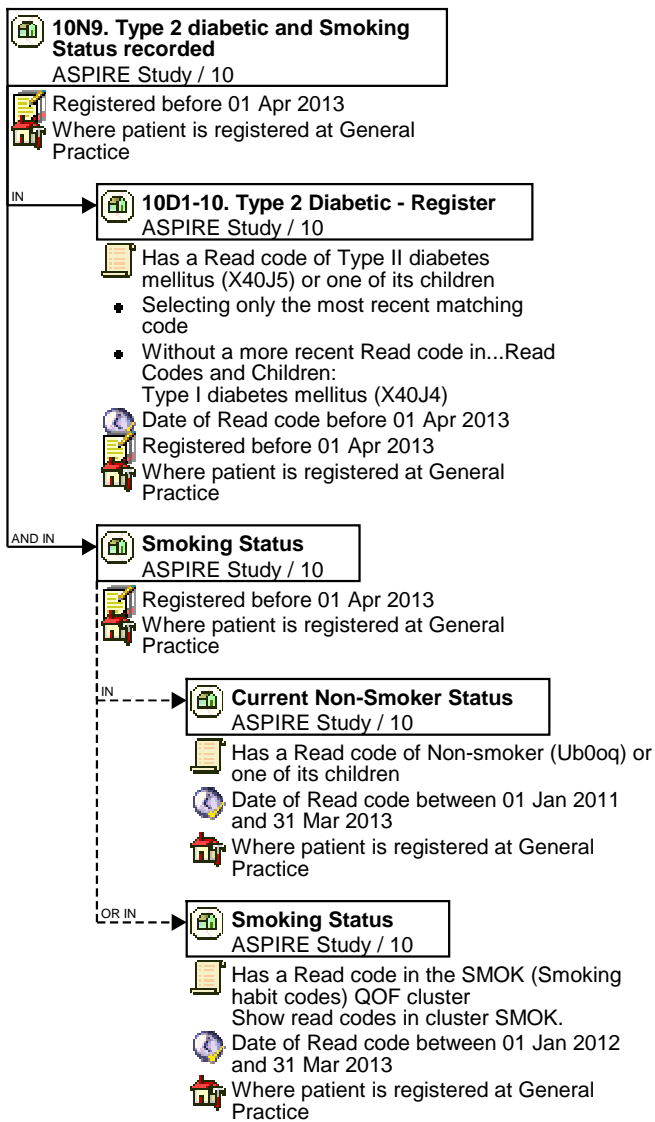

Supplement: Additional file 4 — Folder containing SystmOne™ search algorithms. (ZIP 12.7 mb) [file 12875_2015_350_MOESM4_ESM.zip › Aspire S1 diagrams tw edired/10N9 (DM processes #71).pdf]

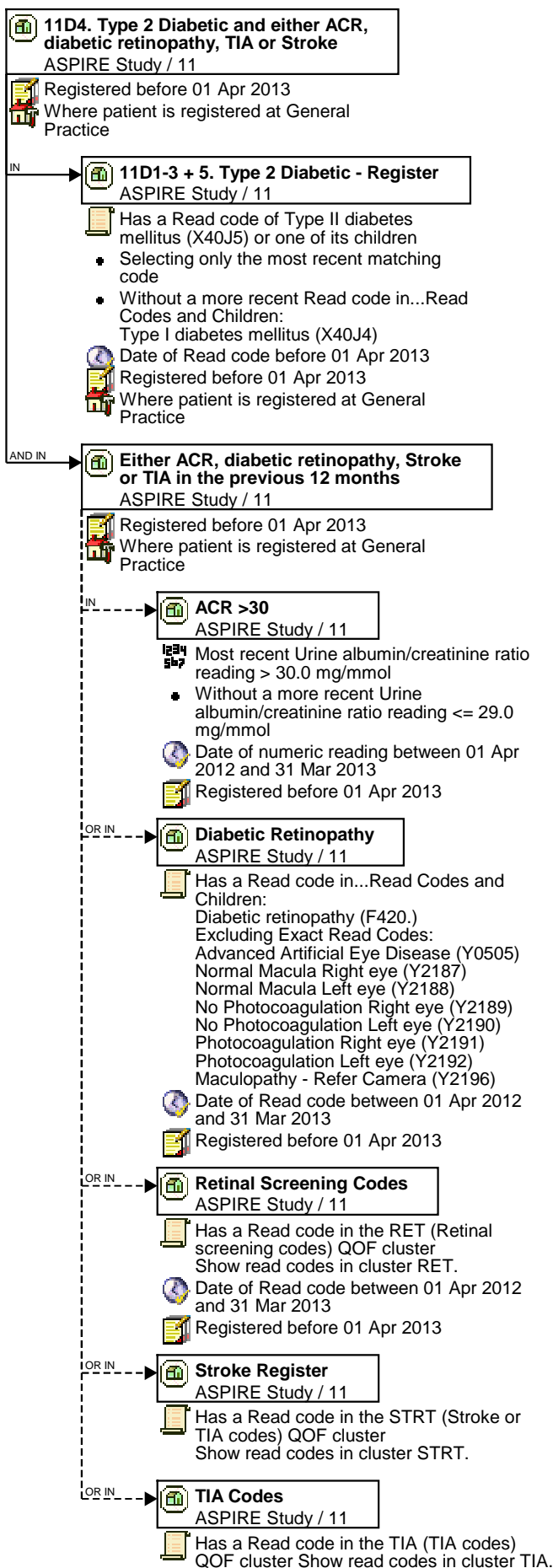

Supplement: Additional file 4 — Folder containing SystmOne™ search algorithms. (ZIP 12.7 mb) [file 12875_2015_350_MOESM4_ESM.zip › Aspire S1 diagrams tw edired/11D4 (DM outcomes #73).pdf]

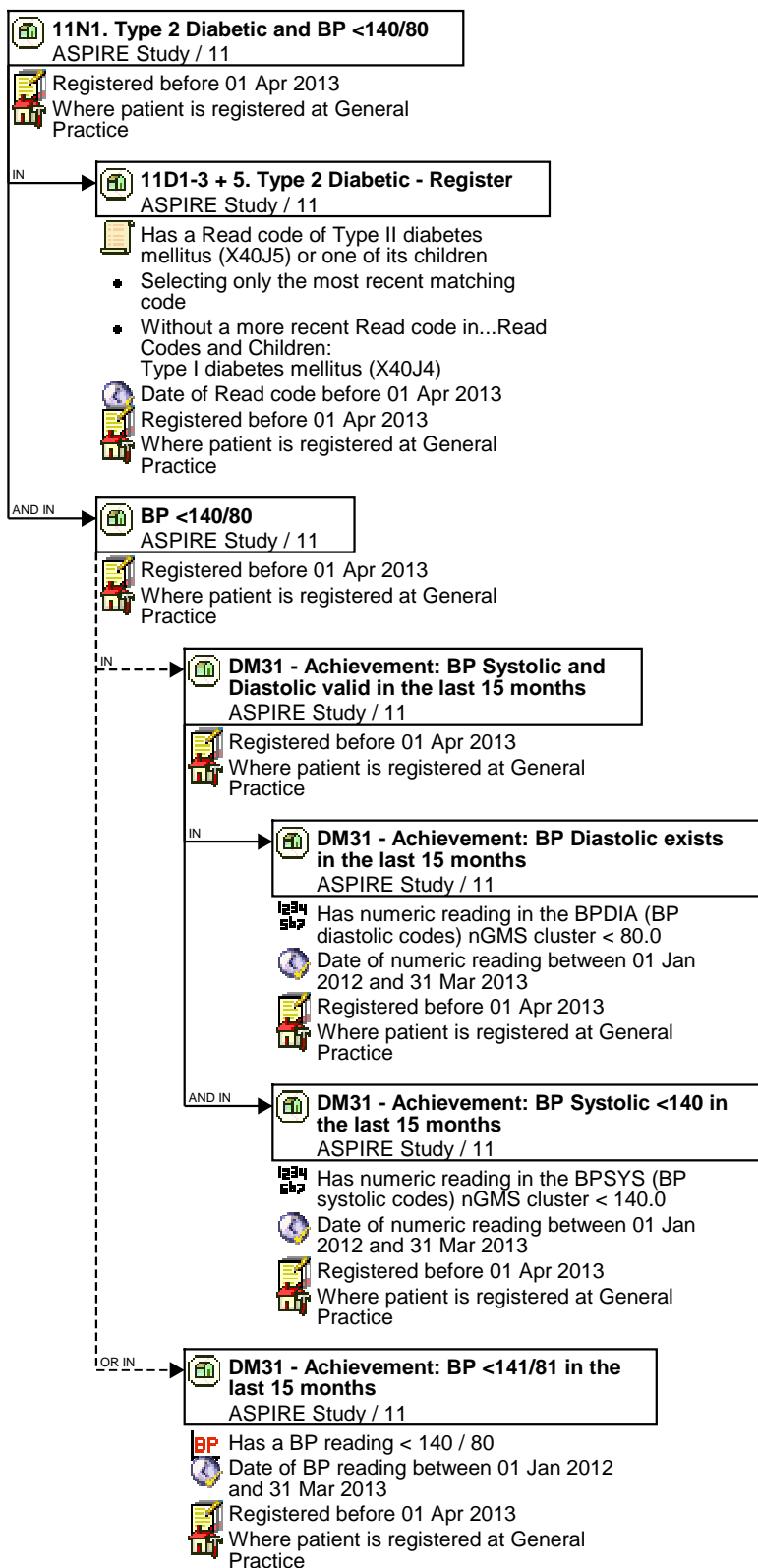

Supplement: Additional file 4 — Folder containing SystmOne™ search algorithms. (ZIP 12.7 mb) [file 12875_2015_350_MOESM4_ESM.zip › Aspire S1 diagrams tw edired/11N1 (DM outcomes #73).pdf]

|       |              |
|-------|--------------|
| ————  | Mandatory In |
| ----- | Optional In  |
| ..... | Not In       |

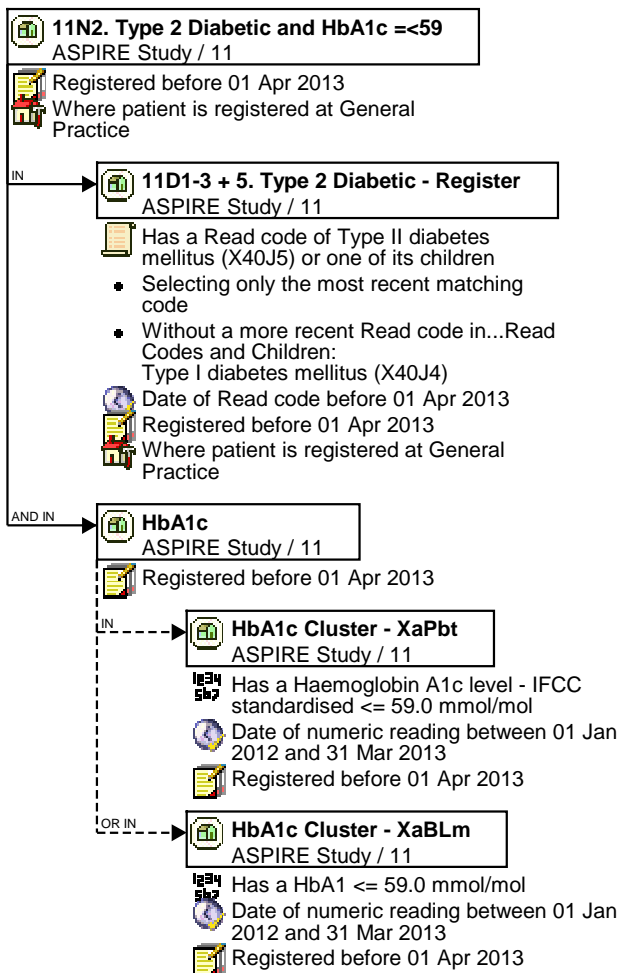

Supplement: Additional file 4 — Folder containing SystmOne™ search algorithms. (ZIP 12.7 mb) [file 12875_2015_350_MOESM4_ESM.zip › Aspire S1 diagrams tw edired/11N2 (DM outcomes #73).pdf]

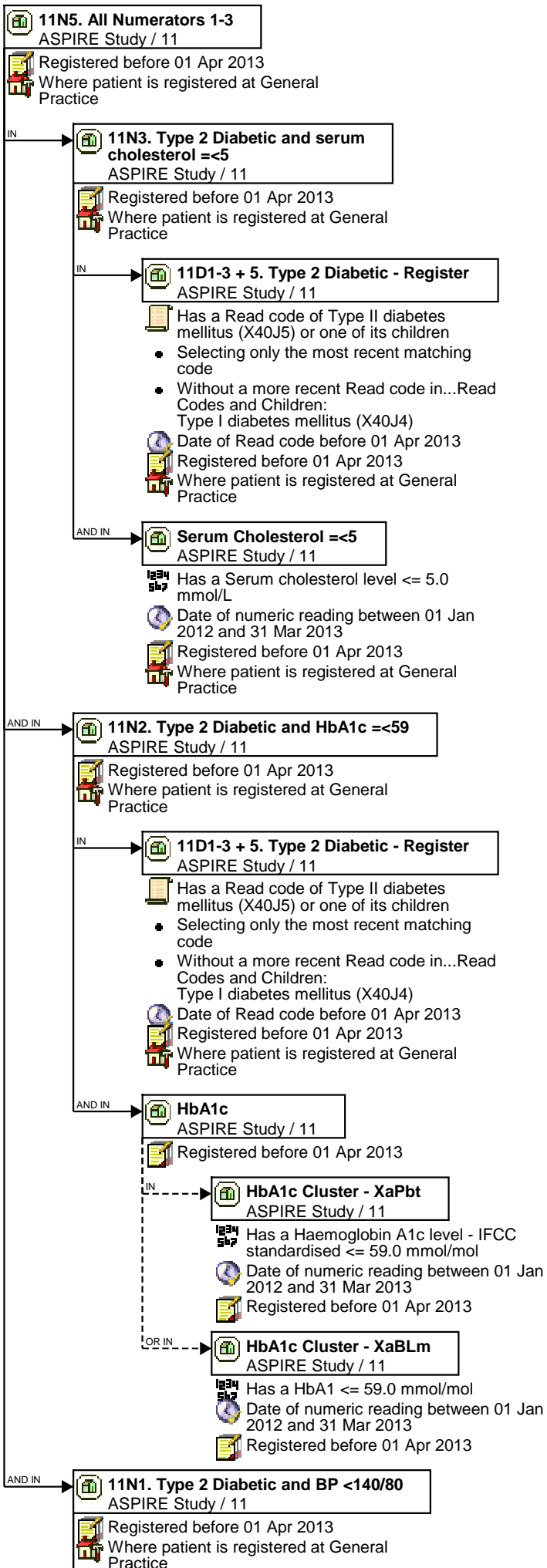

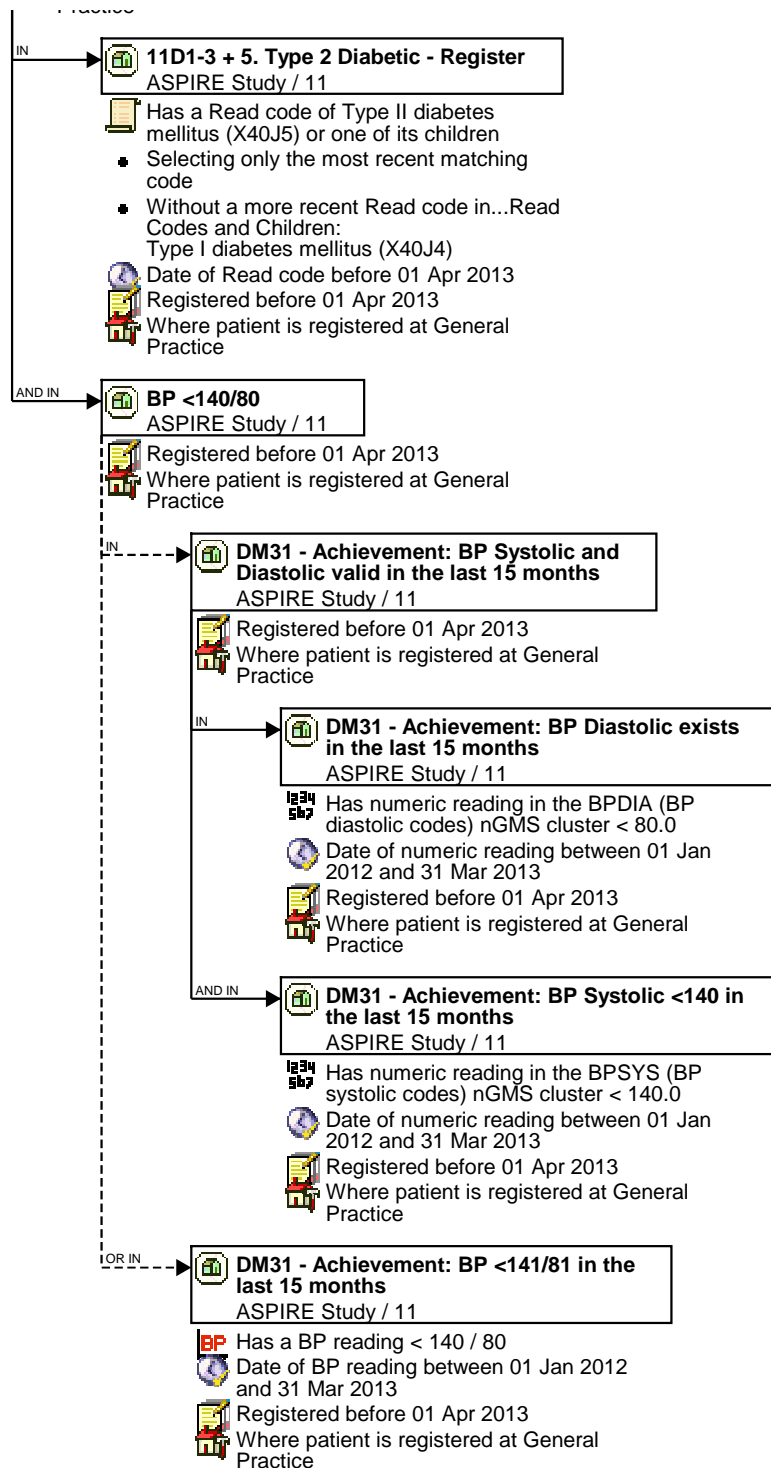

Supplement: Additional file 4 — Folder containing SystmOne™ search algorithms. (ZIP 12.7 mb) [file 12875_2015_350_MOESM4_ESM.zip › Aspire S1 diagrams tw edired/11N5 (DM outcomes #73).pdf]

|       |              |
|-------|--------------|
| ——    | Mandatory In |
| ----  | Optional In  |
| ..... | Not In       |

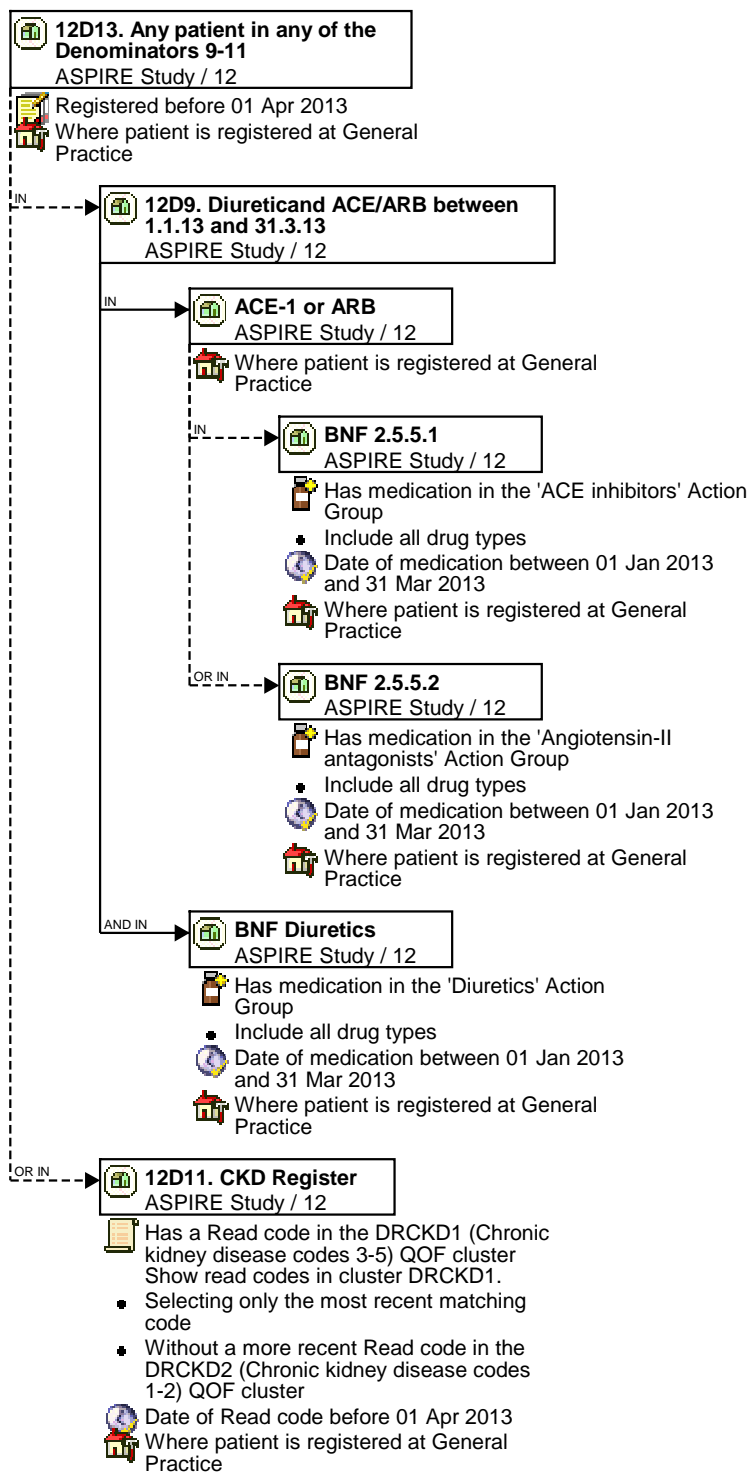

Supplement: Additional file 4 — Folder containing SystmOne™ search algorithms. (ZIP 12.7 mb) [file 12875_2015_350_MOESM4_ESM.zip › Aspire S1 diagrams tw edired/12D13 (Risky p).pdf]

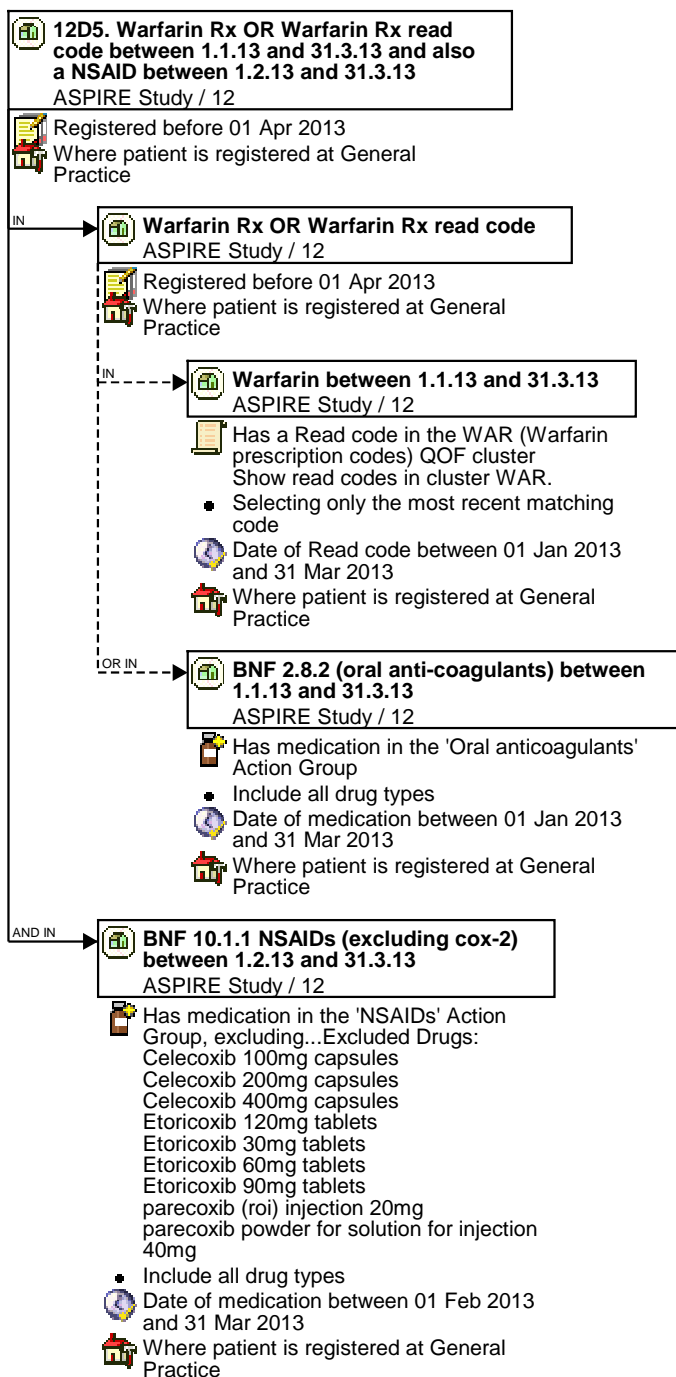

Supplement: Additional file 4 — Folder containing SystmOne™ search algorithms. (ZIP 12.7 mb) [file 12875_2015_350_MOESM4_ESM.zip › Aspire S1 diagrams tw edired/12D5 (Risky p).pdf]

|       |              |
|-------|--------------|
| ————  | Mandatory In |
| ----- | Optional In  |
| ..... | Not In       |

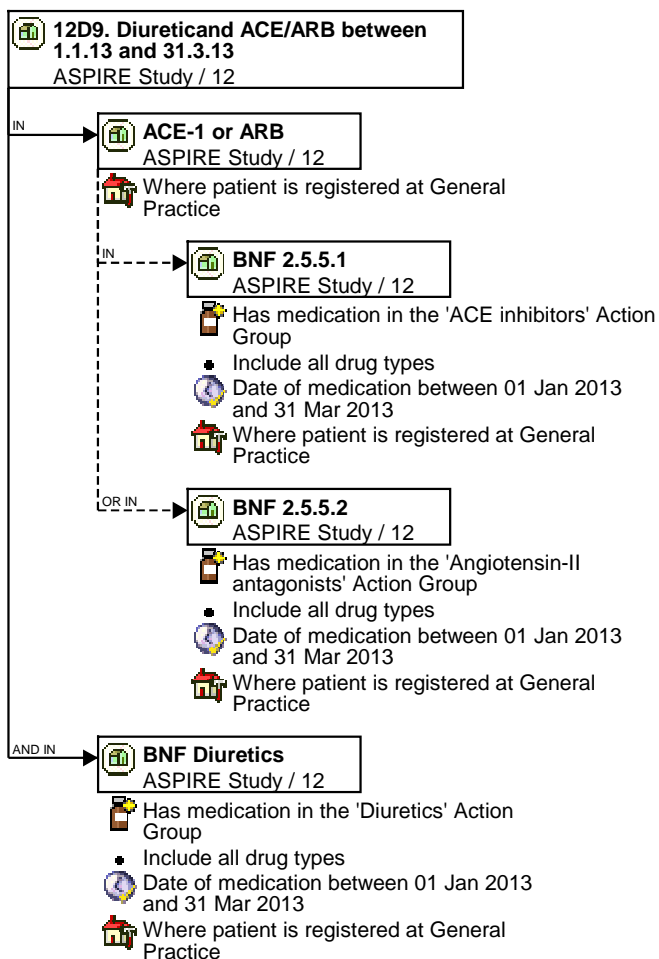

Supplement: Additional file 4 — Folder containing SystmOne™ search algorithms. (ZIP 12.7 mb) [file 12875_2015_350_MOESM4_ESM.zip › Aspire S1 diagrams tw edired/12D9 (Risky p).pdf]

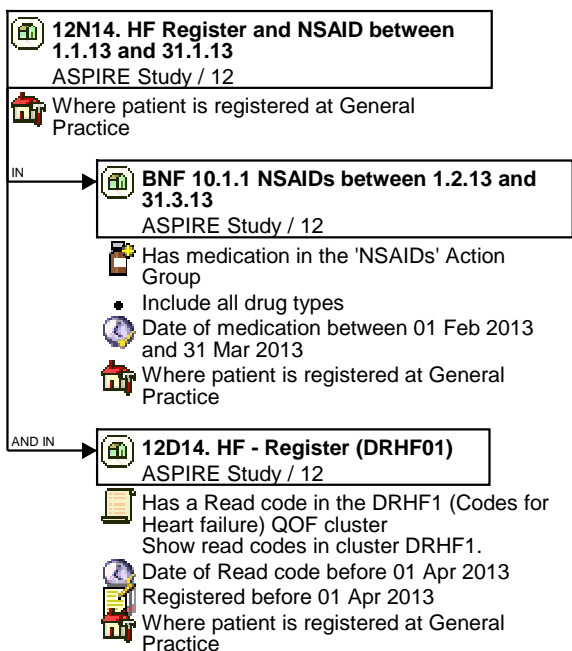

Supplement: Additional file 4 — Folder containing SystmOne™ search algorithms. (ZIP 12.7 mb) [file 12875_2015_350_MOESM4_ESM.zip › Aspire S1 diagrams tw edired/12N14 (Risky p).pdf]

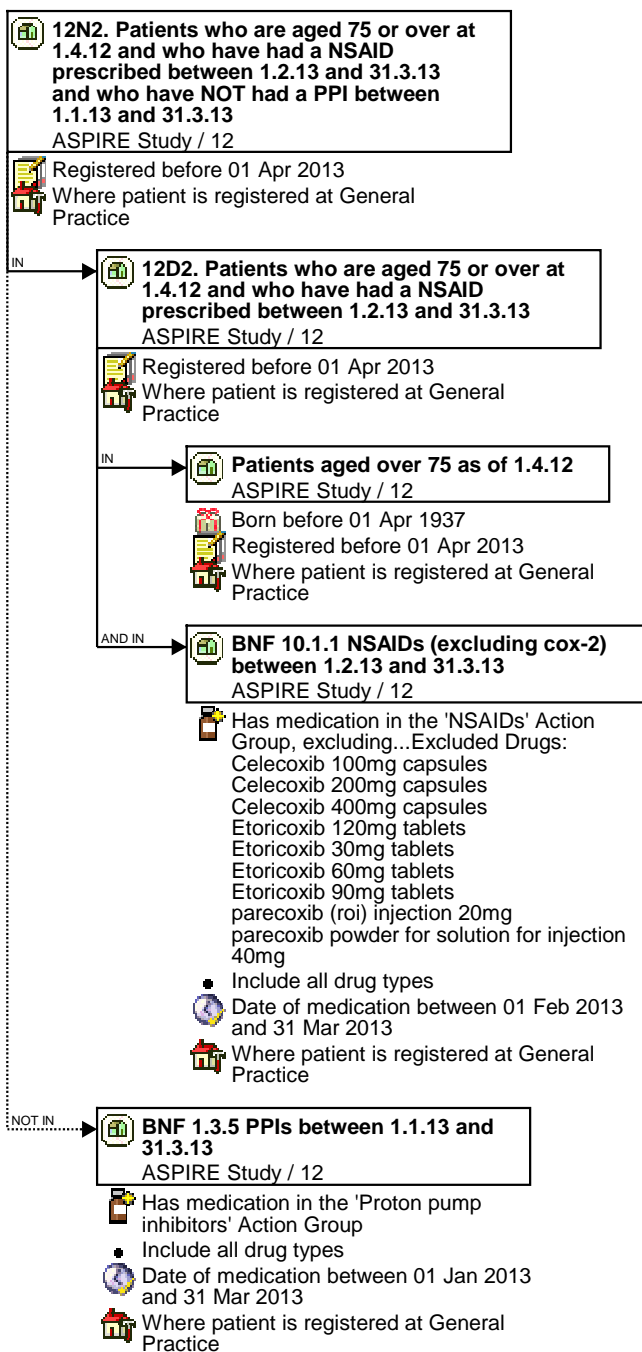

Supplement: Additional file 4 — Folder containing SystmOne™ search algorithms. (ZIP 12.7 mb) [file 12875_2015_350_MOESM4_ESM.zip › Aspire S1 diagrams tw edired/12N2 (Risky p).pdf]

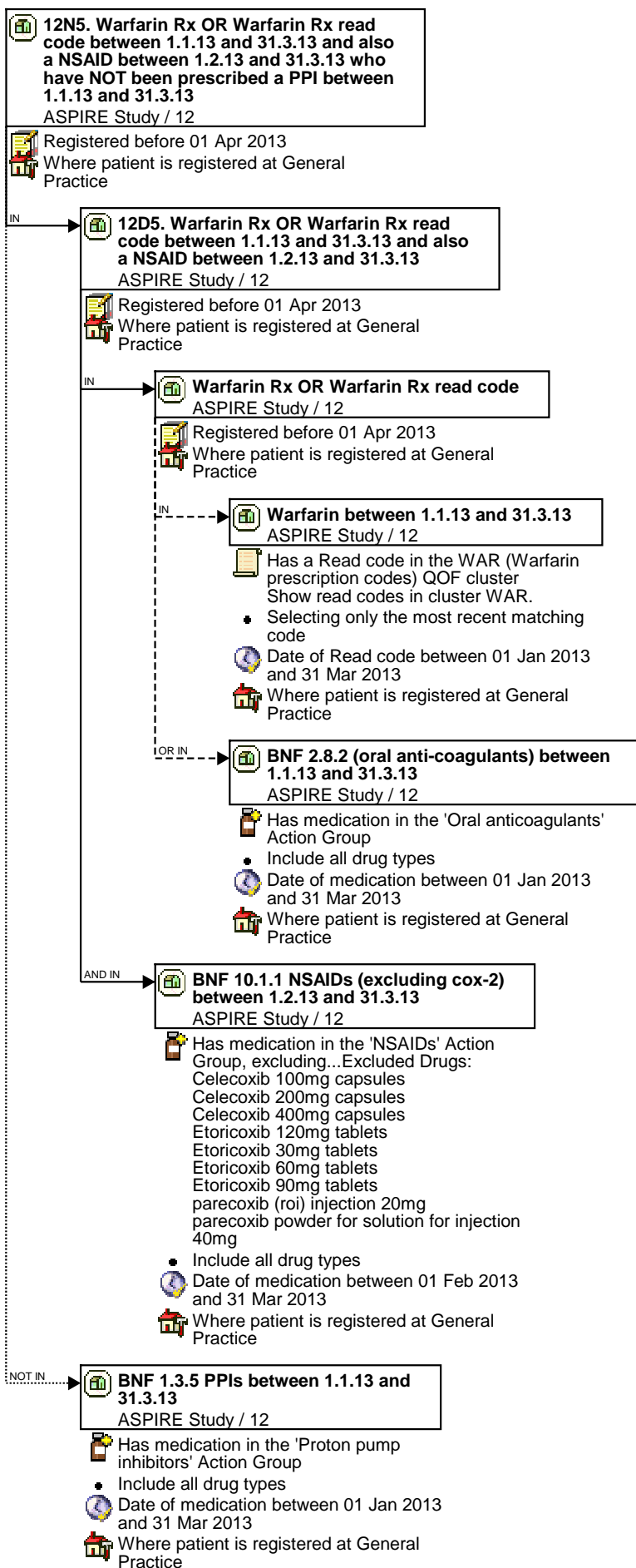

Supplement: Additional file 4 — Folder containing SystmOne™ search algorithms. (ZIP 12.7 mb) [file 12875_2015_350_MOESM4_ESM.zip › Aspire S1 diagrams tw edired/12N5 (Risky p).pdf]

|       |              |
|-------|--------------|
| —     | Mandatory In |
| ----  | Optional In  |
| ..... | Not In       |

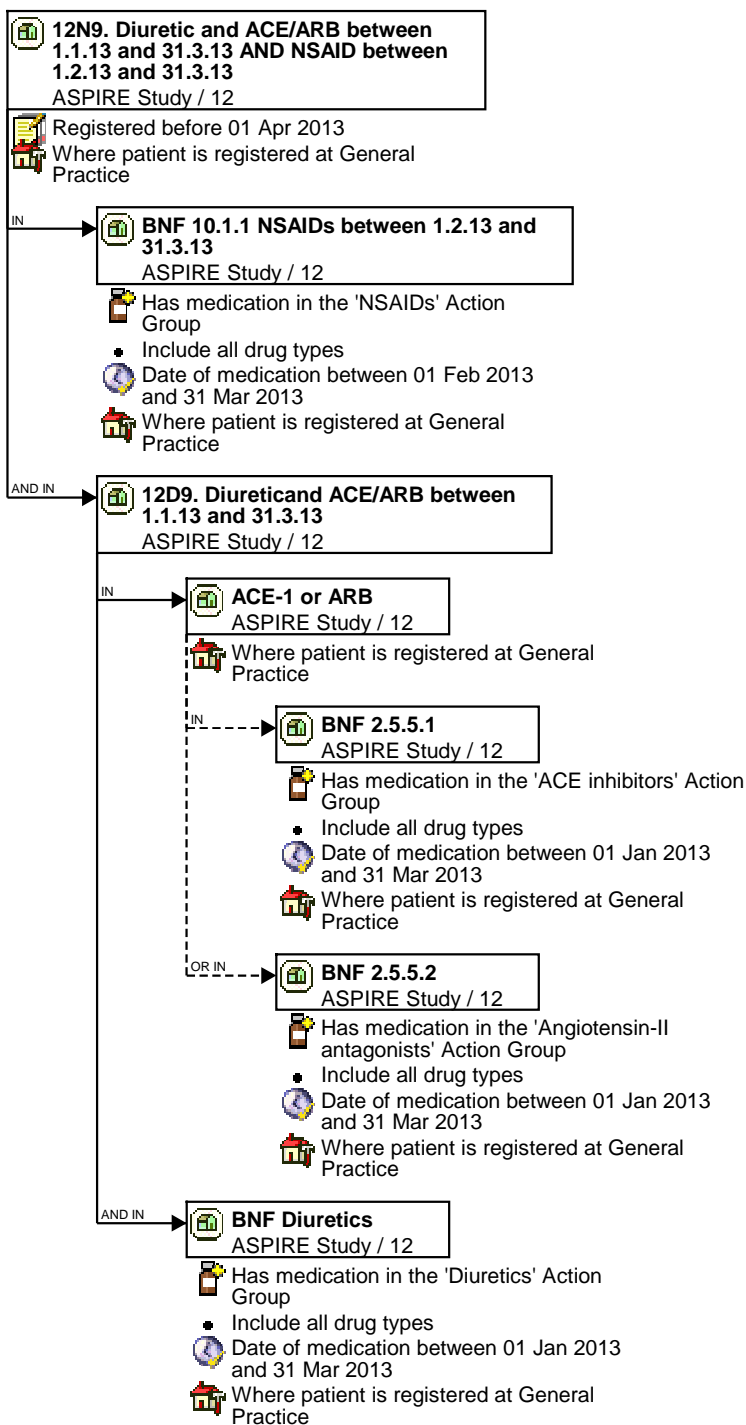

Supplement: Additional file 4 — Folder containing SystmOne™ search algorithms. (ZIP 12.7 mb) [file 12875_2015_350_MOESM4_ESM.zip › Aspire S1 diagrams tw edired/12N9 (Risky p).pdf]

|       |              |
|-------|--------------|
| ————  | Mandatory In |
| ----- | Optional In  |
| ..... | Not In       |

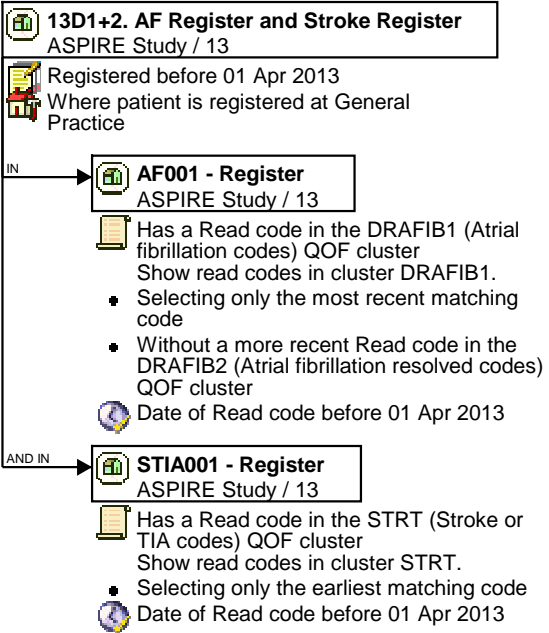

Supplement: Additional file 4 — Folder containing SystmOne™ search algorithms. (ZIP 12.7 mb) [file 12875_2015_350_MOESM4_ESM.zip › Aspire S1 diagrams tw edired/13D1+2 (AF #39).pdf]

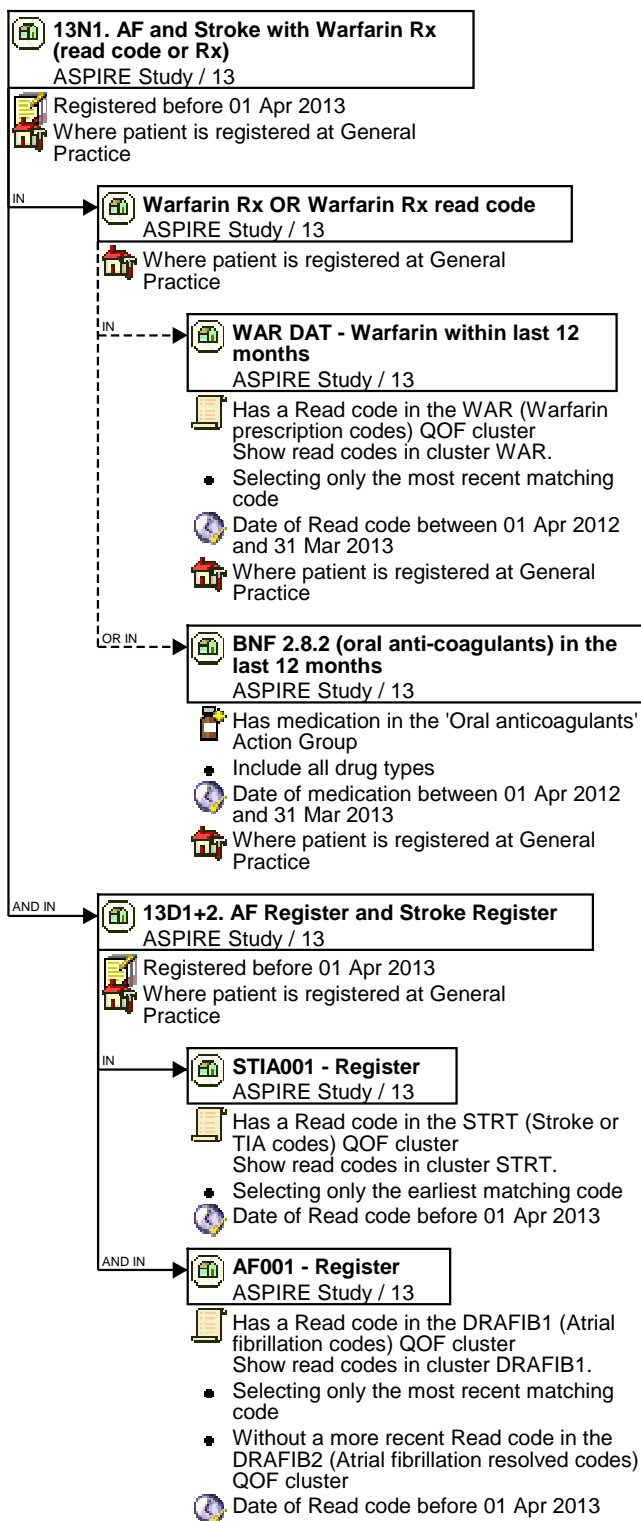

Supplement: Additional file 4 — Folder containing SystmOne™ search algorithms. (ZIP 12.7 mb) [file 12875_2015_350_MOESM4_ESM.zip › Aspire S1 diagrams tw edired/13N1 (AF #39).pdf]

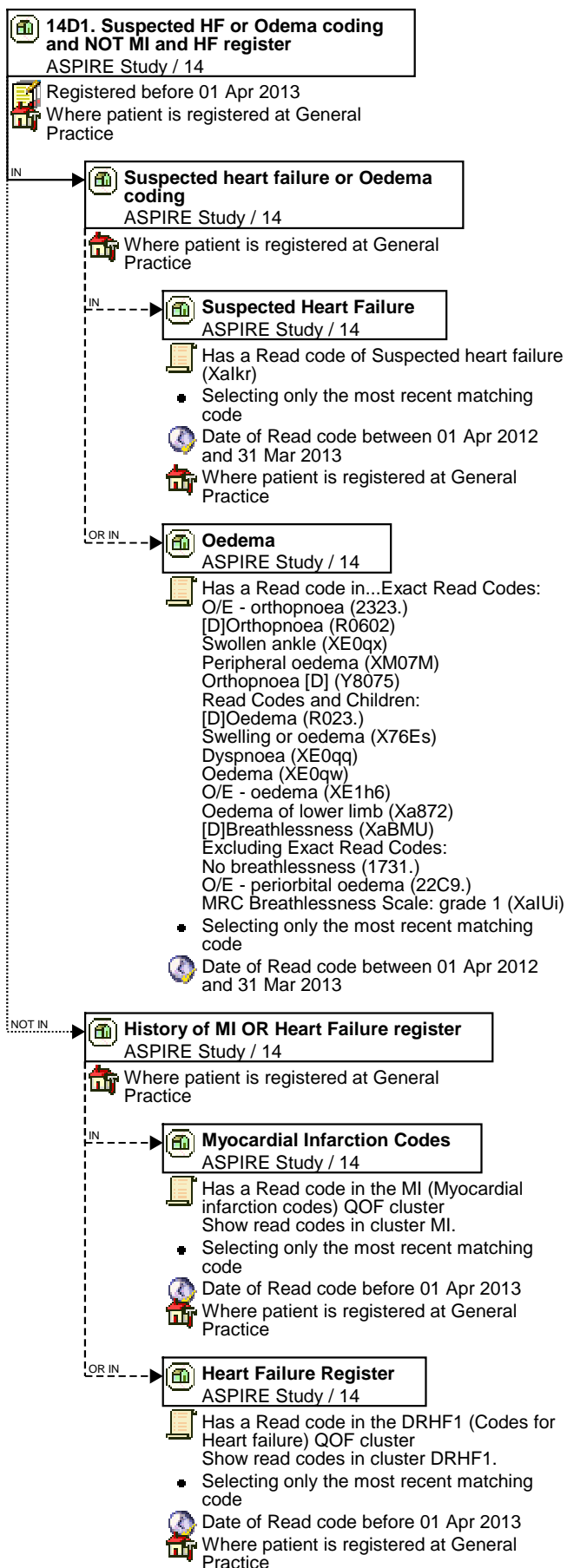

Supplement: Additional file 4 — Folder containing SystmOne™ search algorithms. (ZIP 12.7 mb) [file 12875_2015_350_MOESM4_ESM.zip › Aspire S1 diagrams tw edired/14D1 (CHF #42).pdf]

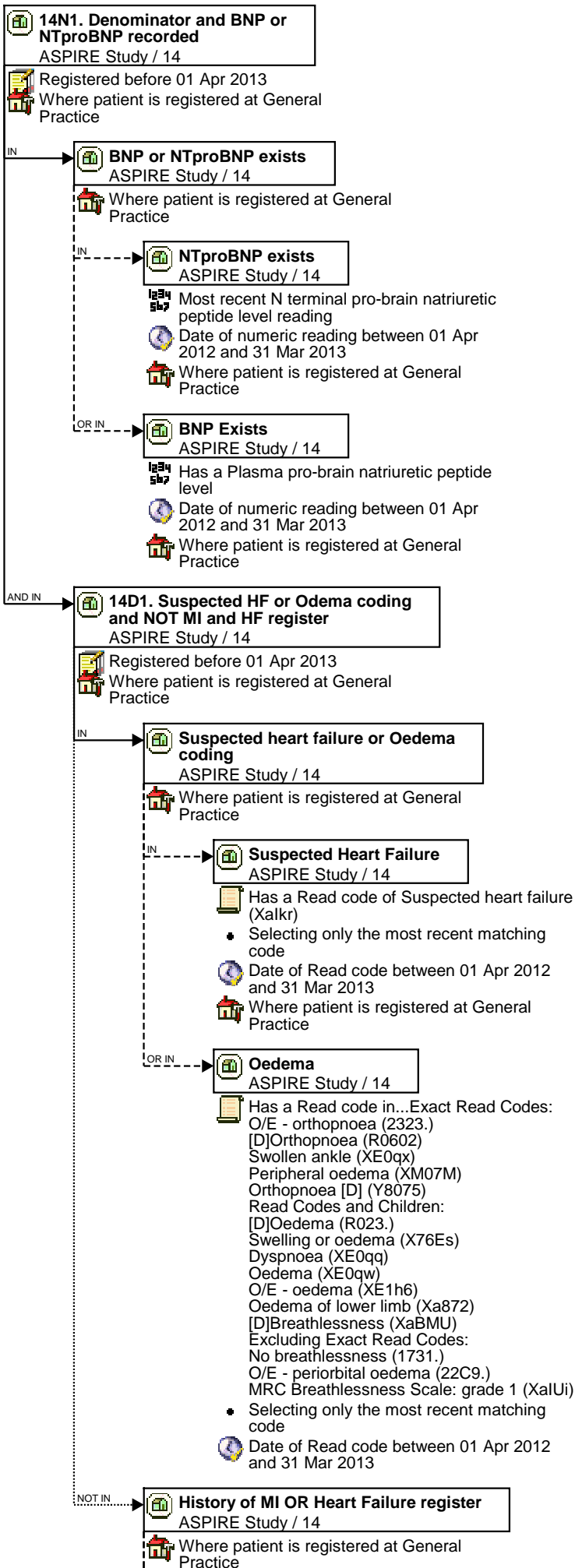

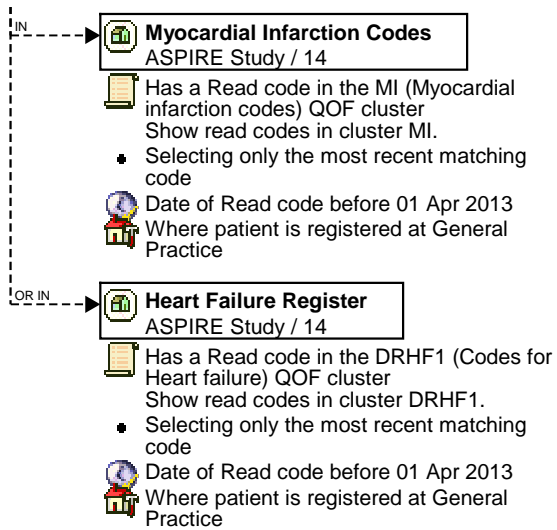

Supplement: Additional file 4 — Folder containing SystmOne™ search algorithms. (ZIP 12.7 mb) [file 12875_2015_350_MOESM4_ESM.zip › Aspire S1 diagrams tw edired/14N1 (CHF #42).pdf]

|       |              |
|-------|--------------|
| ————  | Mandatory In |
| ----- | Optional In  |
| ..... | Not In       |

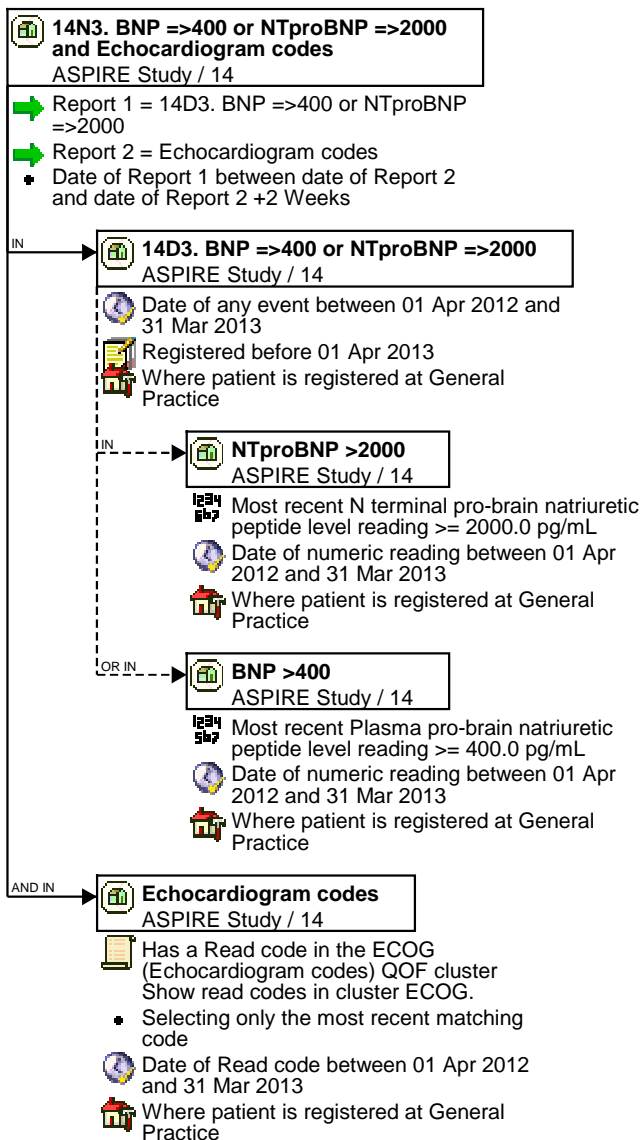

Supplement: Additional file 4 — Folder containing SystmOne™ search algorithms. (ZIP 12.7 mb) [file 12875_2015_350_MOESM4_ESM.zip › Aspire S1 diagrams tw edired/14N3 (CHF #42).pdf]

|       |              |
|-------|--------------|
| ————  | Mandatory In |
| ----- | Optional In  |
| ..... | Not In       |

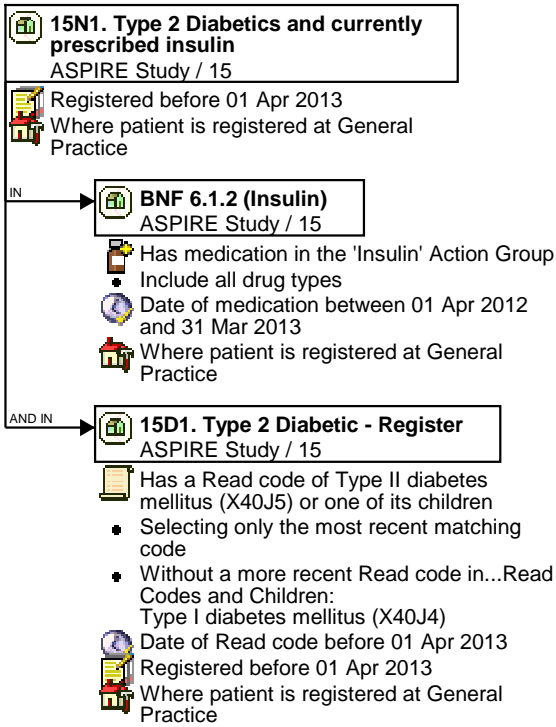

Supplement: Additional file 4 — Folder containing SystmOne™ search algorithms. (ZIP 12.7 mb) [file 12875_2015_350_MOESM4_ESM.zip › Aspire S1 diagrams tw edired/15N1 (DM #33).pdf]

|       |              |
|-------|--------------|
| ——    | Mandatory In |
| ----  | Optional In  |
| ..... | Not In       |

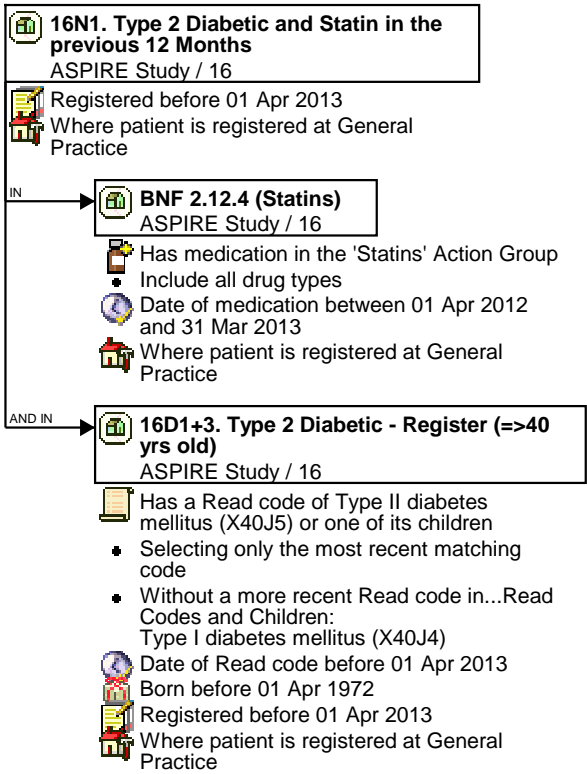

Supplement: Additional file 4 — Folder containing SystmOne™ search algorithms. (ZIP 12.7 mb) [file 12875_2015_350_MOESM4_ESM.zip › Aspire S1 diagrams tw edired/16N1 (DM #36).pdf]

|       |              |
|-------|--------------|
| ————  | Mandatory In |
| ----- | Optional In  |
| ..... | Not In       |

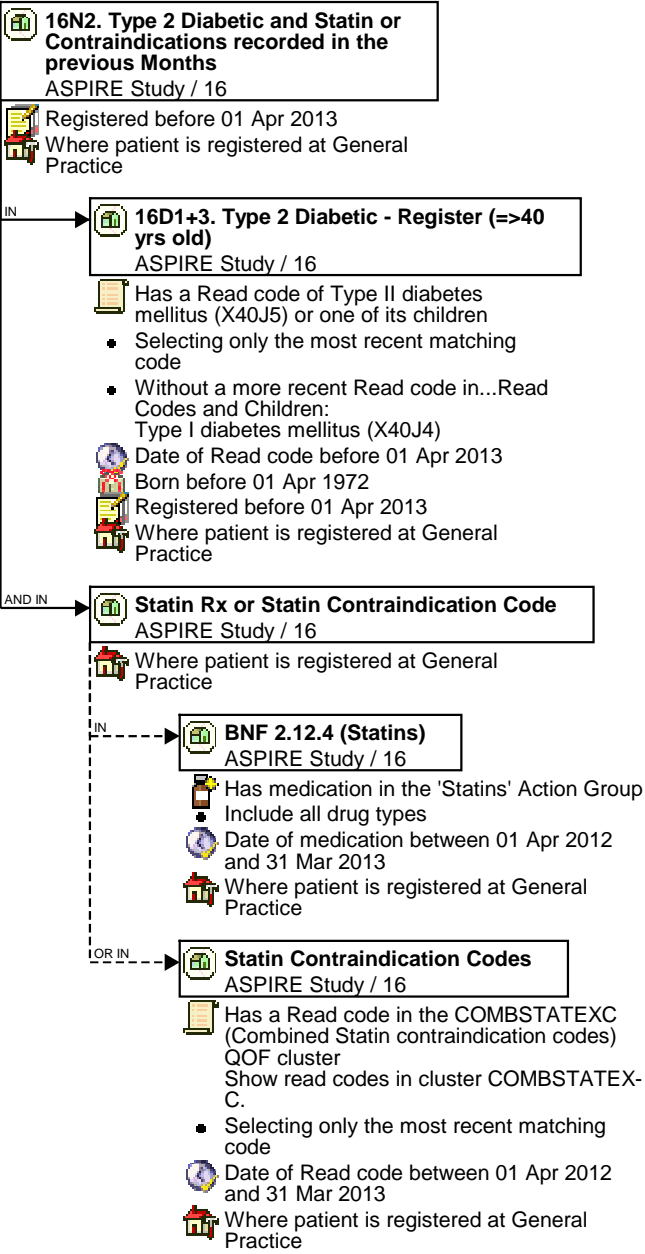

Supplement: Additional file 4 — Folder containing SystmOne™ search algorithms. (ZIP 12.7 mb) [file 12875_2015_350_MOESM4_ESM.zip › Aspire S1 diagrams tw edired/16N2 (DM #36).pdf]

|       |              |
|-------|--------------|
| ————  | Mandatory In |
| ----- | Optional In  |
| ..... | Not In       |

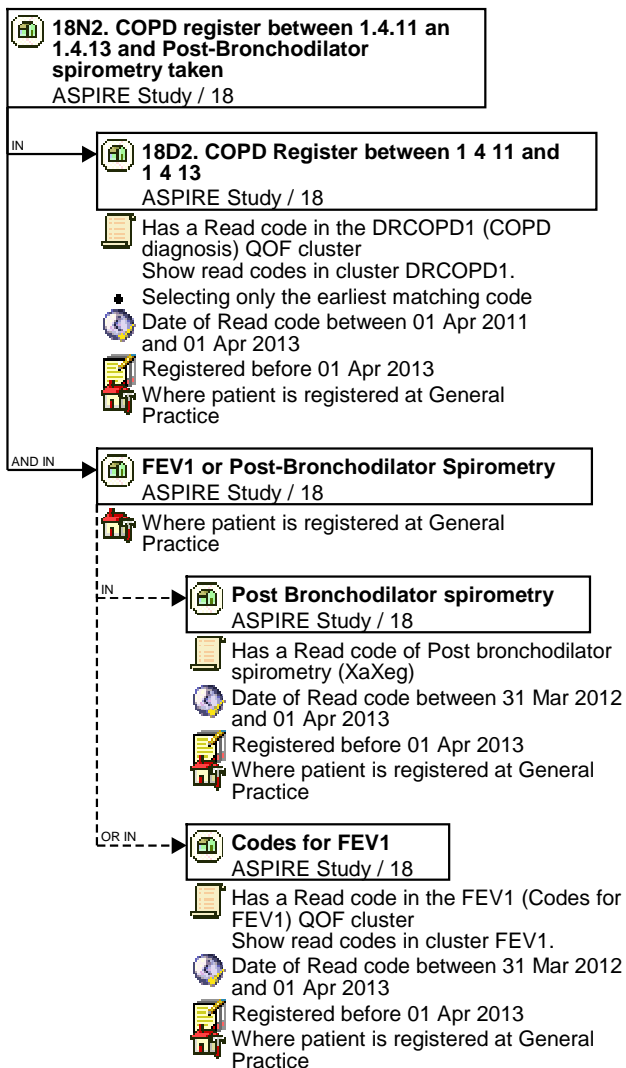

Supplement: Additional file 4 — Folder containing SystmOne™ search algorithms. (ZIP 12.7 mb) [file 12875_2015_350_MOESM4_ESM.zip › Aspire S1 diagrams tw edired/18N2 (COPD #55).pdf]

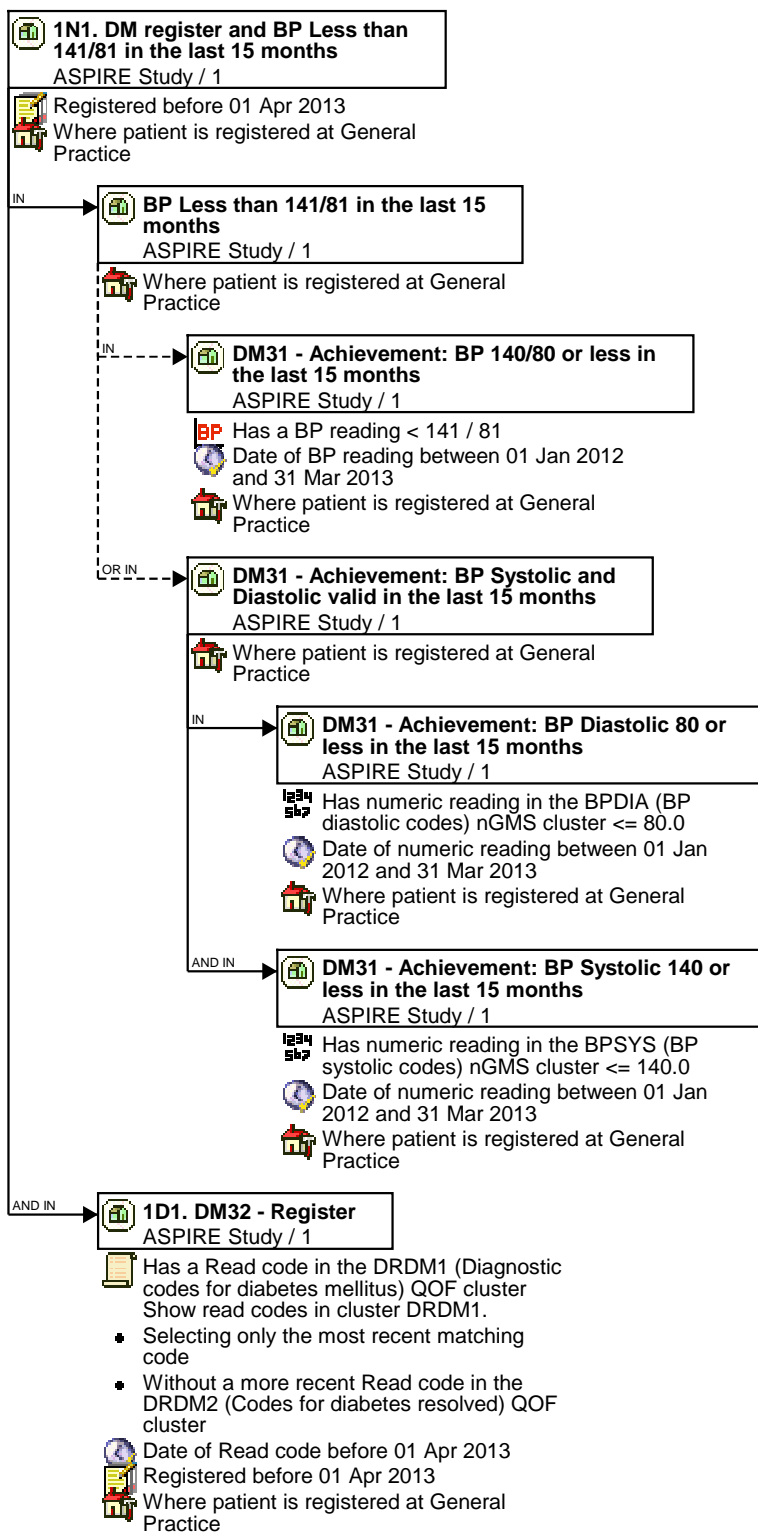

Supplement: Additional file 4 — Folder containing SystmOne™ search algorithms. (ZIP 12.7 mb) [file 12875_2015_350_MOESM4_ESM.zip › Aspire S1 diagrams tw edired/1N1 (Diabetes #37).pdf]

|       |              |
|-------|--------------|
| —     | Mandatory In |
| ----  | Optional In  |
| ..... | Not In       |

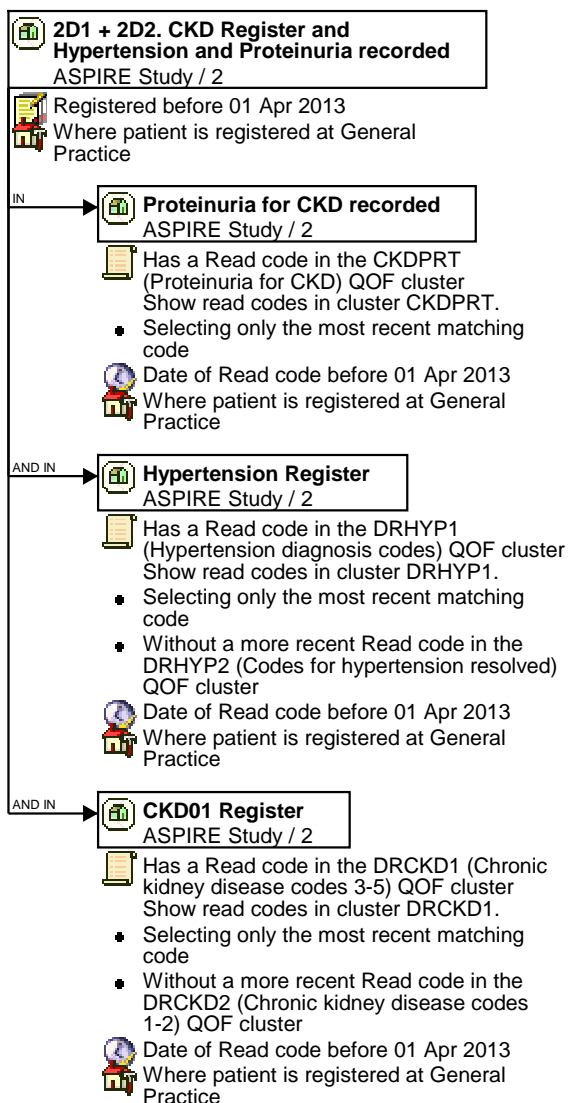

Supplement: Additional file 4 — Folder containing SystmOne™ search algorithms. (ZIP 12.7 mb) [file 12875_2015_350_MOESM4_ESM.zip › Aspire S1 diagrams tw edired/2D1+2 (CKD #17).pdf]

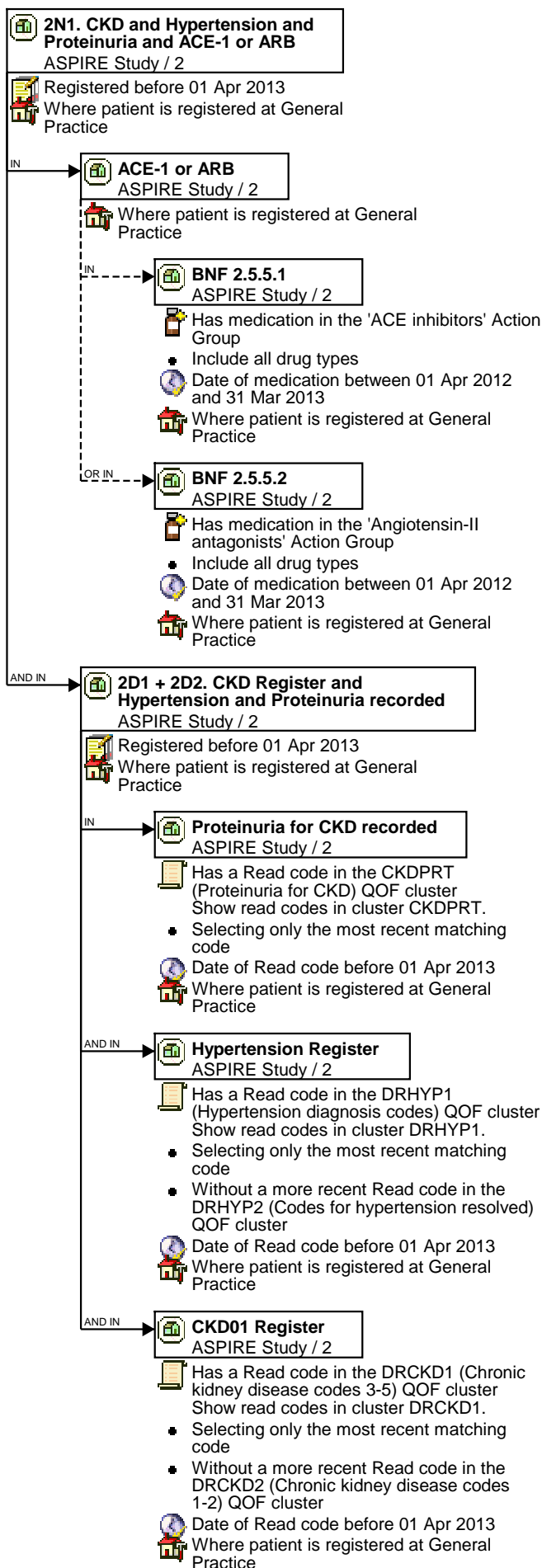

Supplement: Additional file 4 — Folder containing SystmOne™ search algorithms. (ZIP 12.7 mb) [file 12875_2015_350_MOESM4_ESM.zip › Aspire S1 diagrams tw edired/2N1 (CKD #17).pdf]

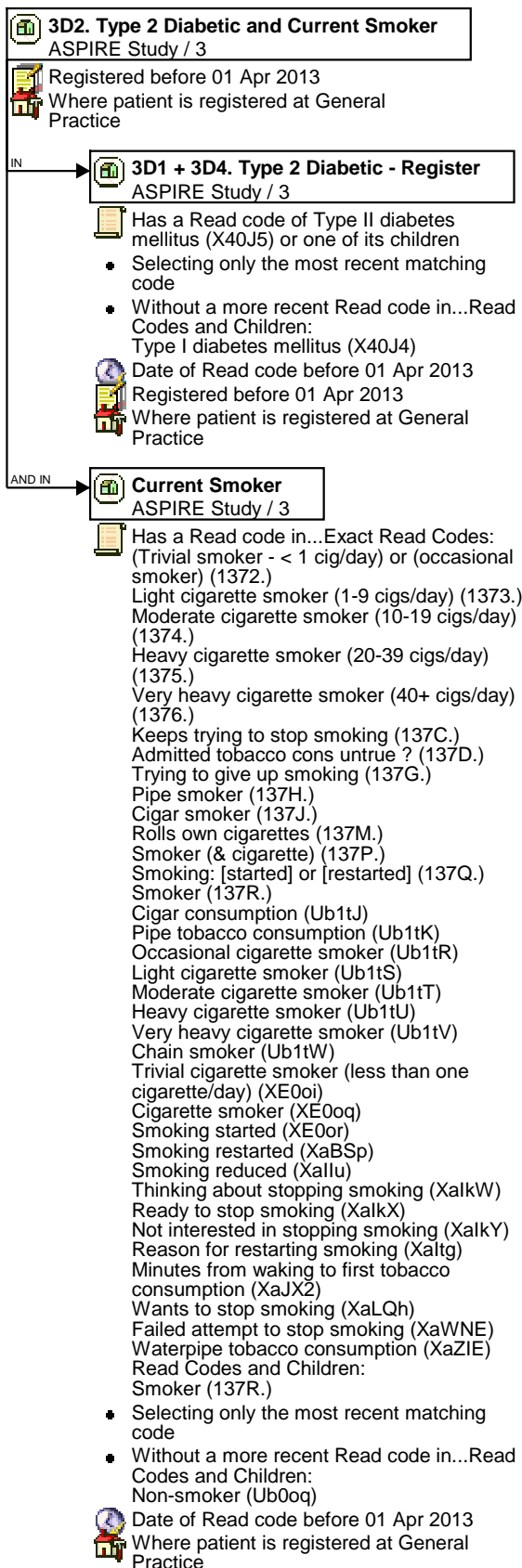

Supplement: Additional file 4 — Folder containing SystmOne™ search algorithms. (ZIP 12.7 mb) [file 12875_2015_350_MOESM4_ESM.zip › Aspire S1 diagrams tw edired/3D2 (Diabetes #34).pdf]

|       |              |
|-------|--------------|
| ————  | Mandatory In |
| ----- | Optional In  |
| ..... | Not In       |

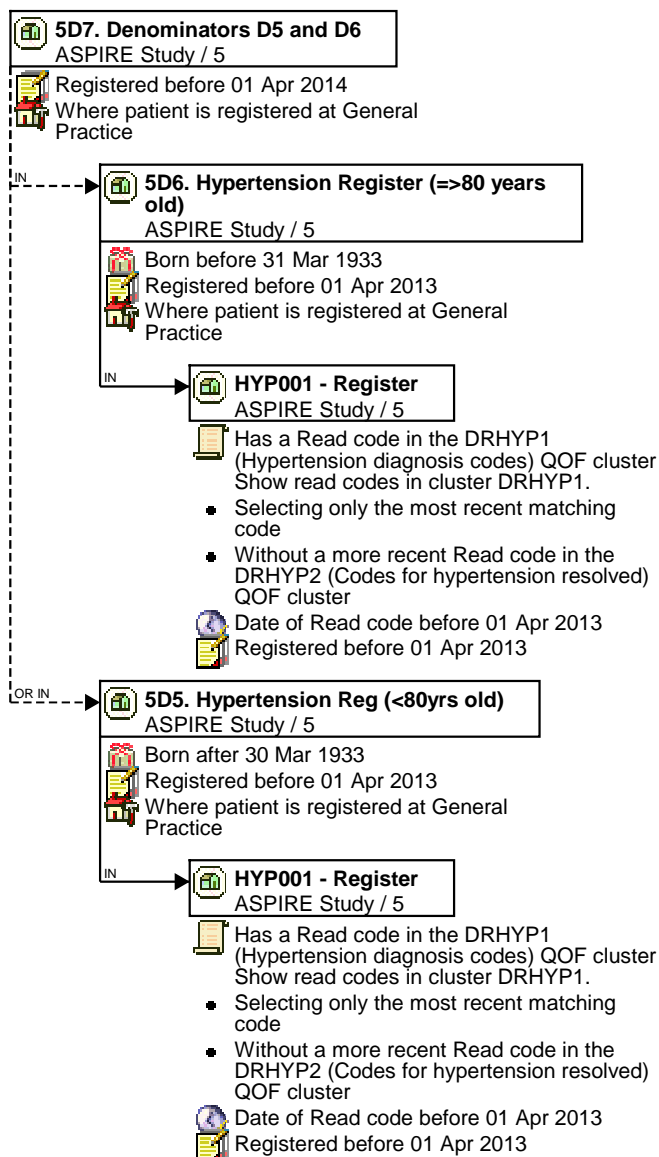

Supplement: Additional file 4 — Folder containing SystmOne™ search algorithms. (ZIP 12.7 mb) [file 12875_2015_350_MOESM4_ESM.zip › Aspire S1 diagrams tw edired/5D7 (HTN targets #77).pdf]

|       |              |
|-------|--------------|
| ————  | Mandatory In |
| ----- | Optional In  |
| ..... | Not In       |

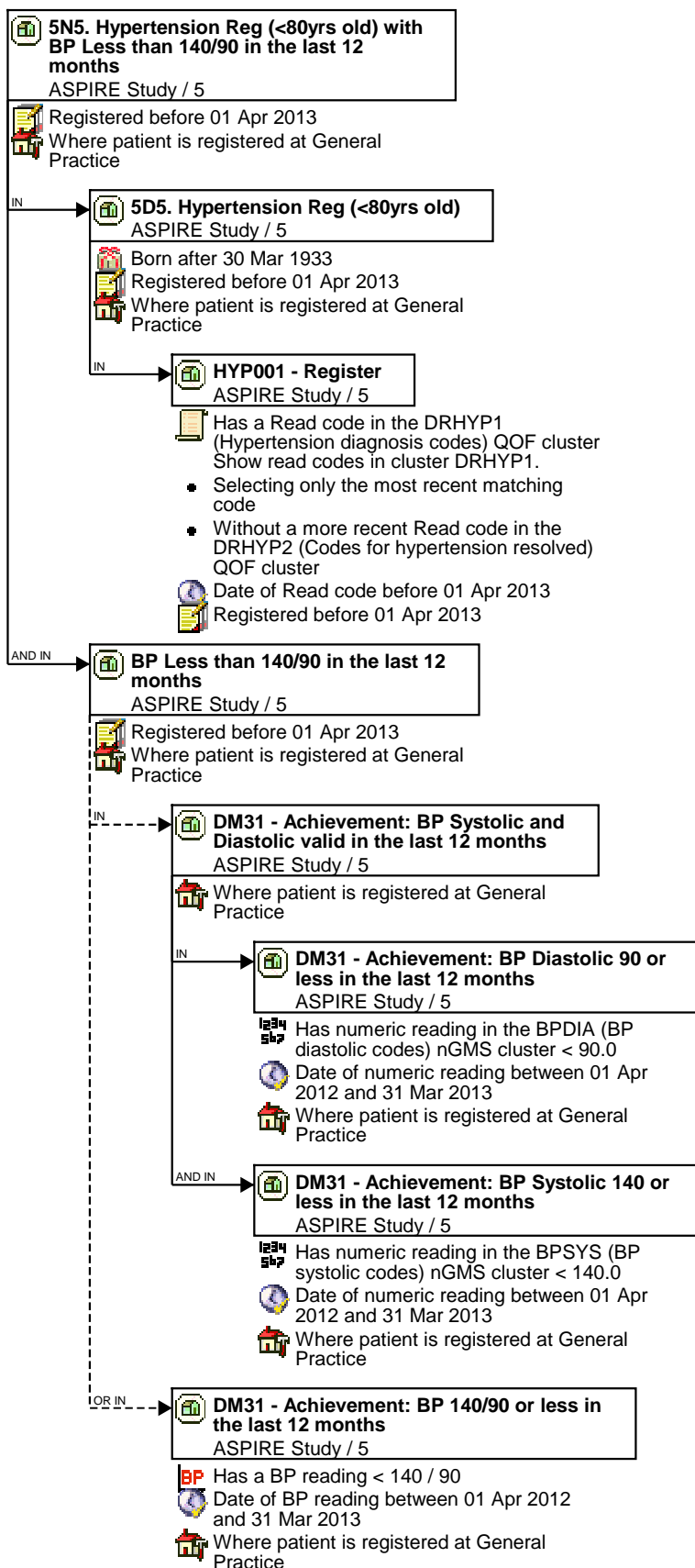

Supplement: Additional file 4 — Folder containing SystmOne™ search algorithms. (ZIP 12.7 mb) [file 12875_2015_350_MOESM4_ESM.zip › Aspire S1 diagrams tw edired/5N5 (HTN targets #77).pdf]

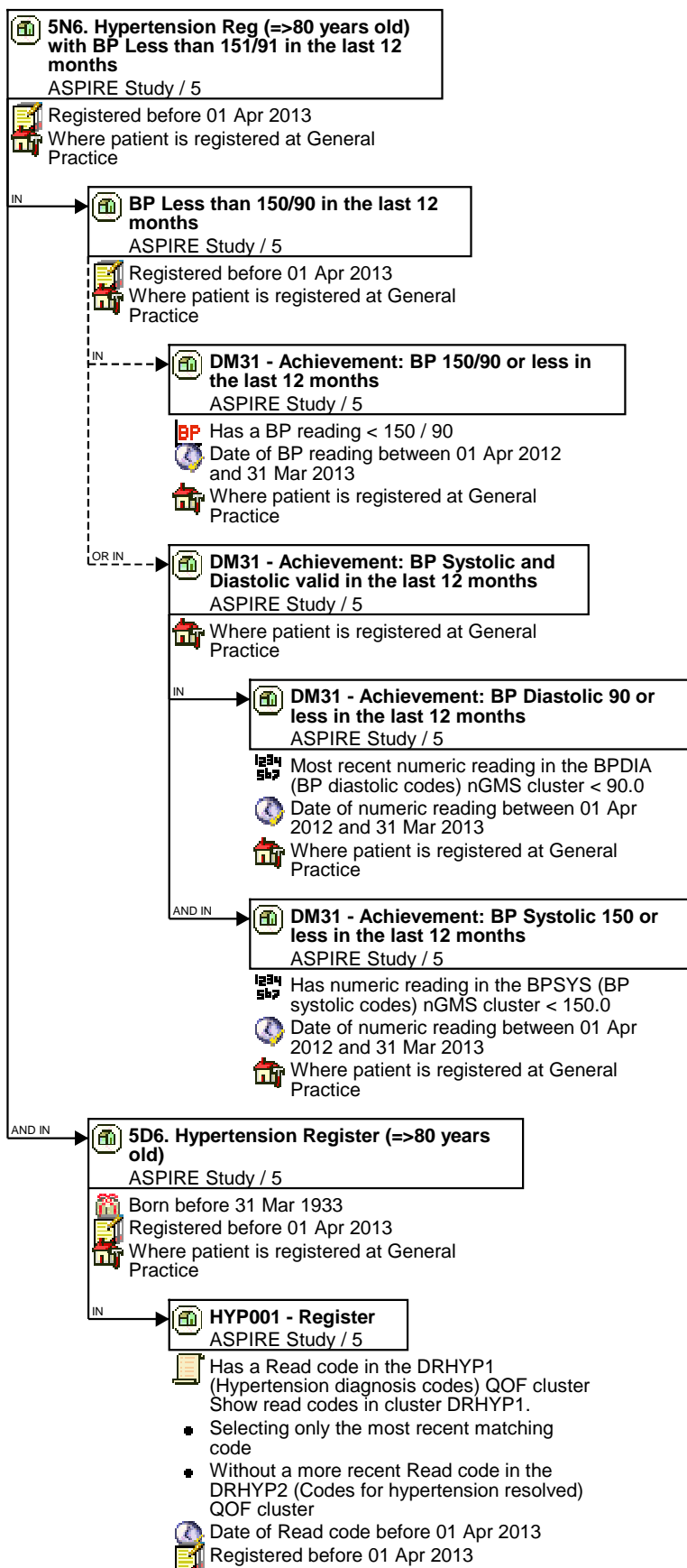

Supplement: Additional file 4 — Folder containing SystmOne™ search algorithms. (ZIP 12.7 mb) [file 12875_2015_350_MOESM4_ESM.zip › Aspire S1 diagrams tw edired/5N6 (HTN targets #77).pdf]

|       |              |
|-------|--------------|
| ————  | Mandatory In |
| ----- | Optional In  |
| ..... | Not In       |

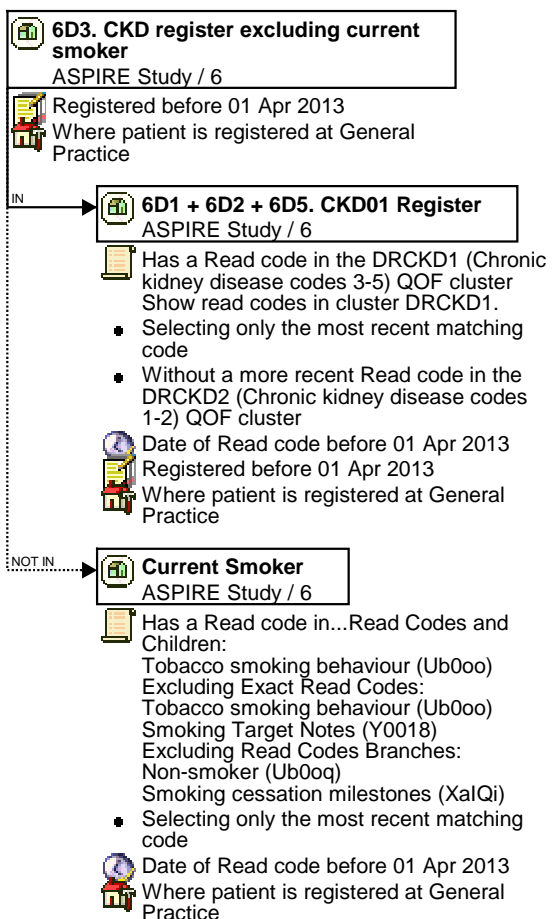

Supplement: Additional file 4 — Folder containing SystmOne™ search algorithms. (ZIP 12.7 mb) [file 12875_2015_350_MOESM4_ESM.zip › Aspire S1 diagrams tw edired/6D3 (CKD #46).pdf]

|       |              |
|-------|--------------|
| —     | Mandatory In |
| ----  | Optional In  |
| ..... | Not In       |

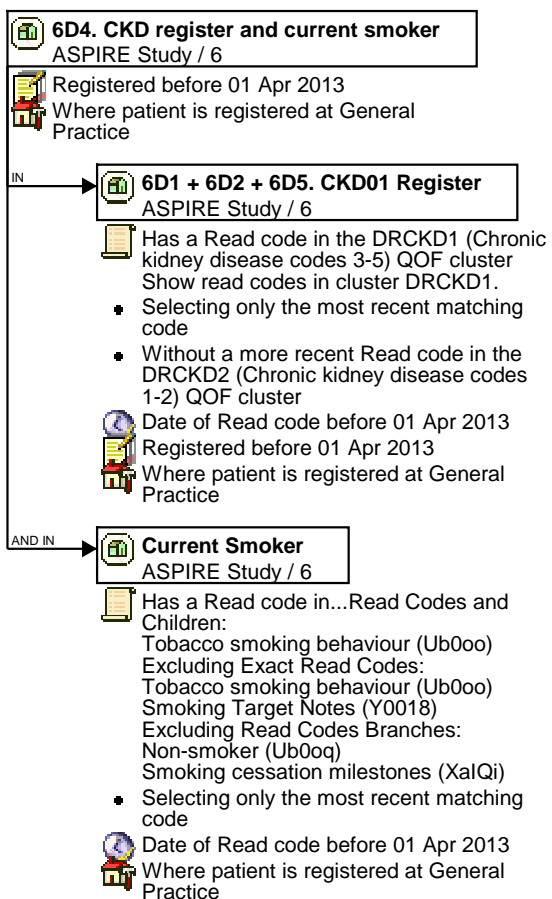

Supplement: Additional file 4 — Folder containing SystmOne™ search algorithms. (ZIP 12.7 mb) [file 12875_2015_350_MOESM4_ESM.zip › Aspire S1 diagrams tw edired/6D4 (CKD #46).pdf]

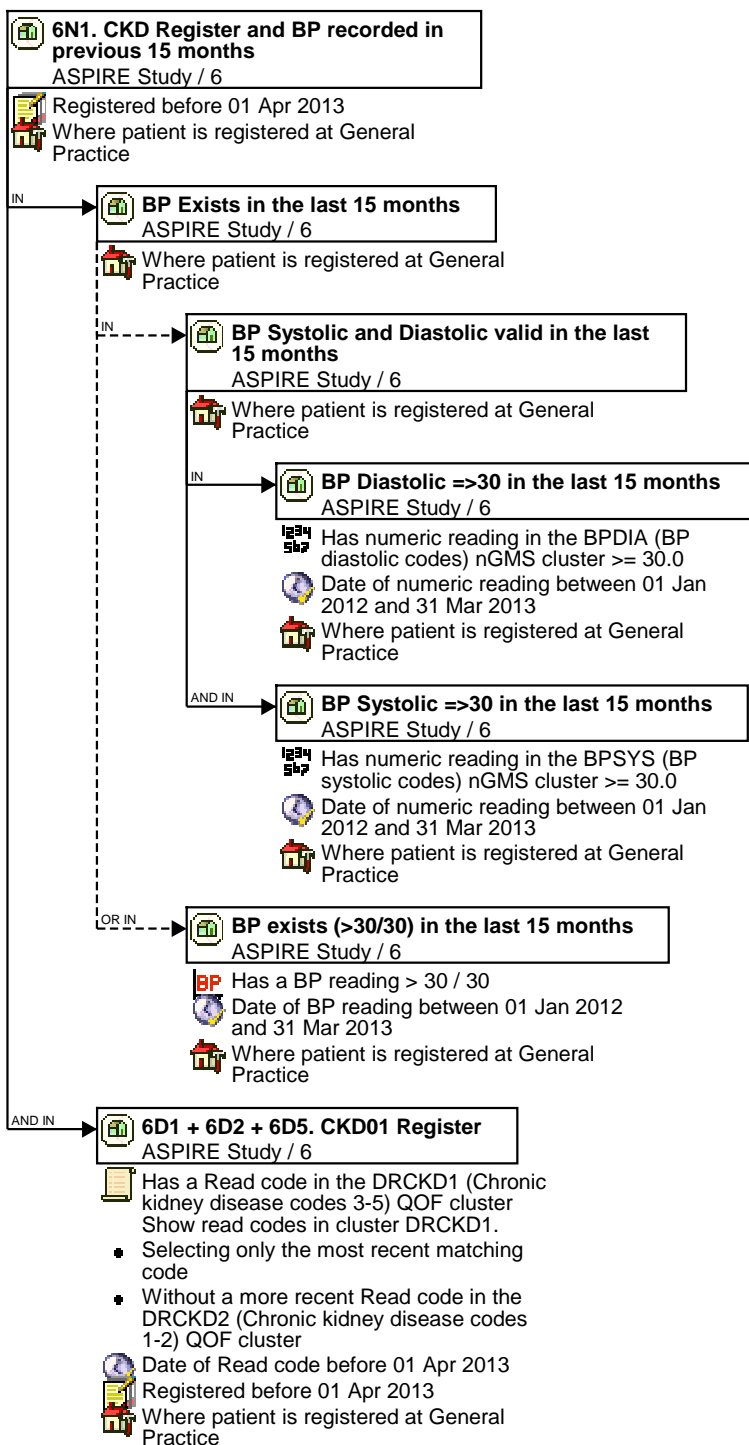

Supplement: Additional file 4 — Folder containing SystmOne™ search algorithms. (ZIP 12.7 mb) [file 12875_2015_350_MOESM4_ESM.zip › Aspire S1 diagrams tw edired/6N1 (CKD #46).pdf]

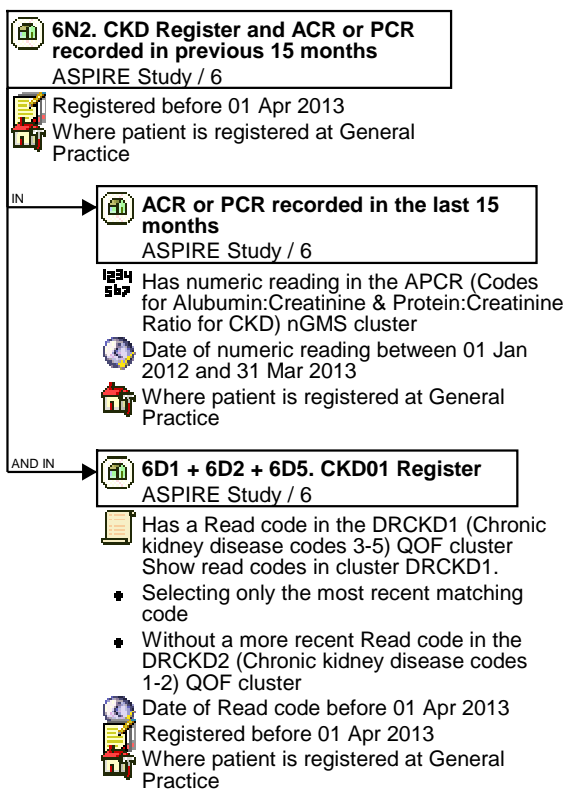

Supplement: Additional file 4 — Folder containing SystmOne™ search algorithms. (ZIP 12.7 mb) [file 12875_2015_350_MOESM4_ESM.zip › Aspire S1 diagrams tw edired/6N2 (CKD #46).pdf]

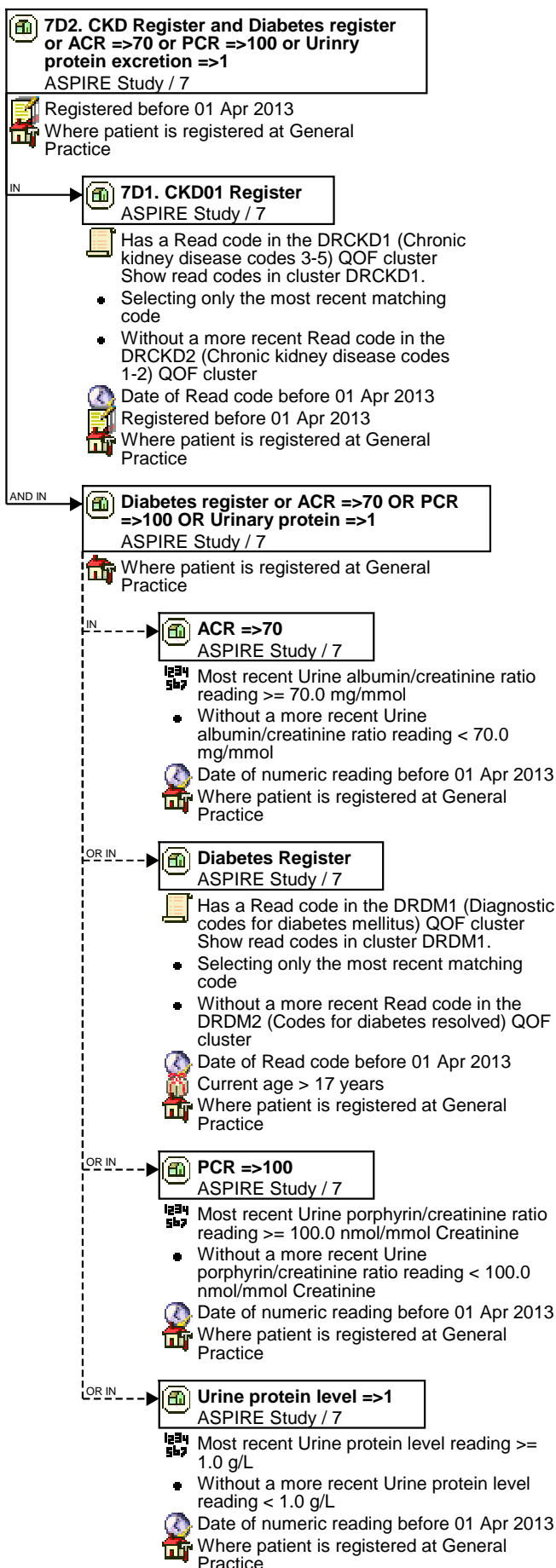

Supplement: Additional file 4 — Folder containing SystmOne™ search algorithms. (ZIP 12.7 mb) [file 12875_2015_350_MOESM4_ESM.zip › Aspire S1 diagrams tw edired/7D2 (CKD #47).pdf]

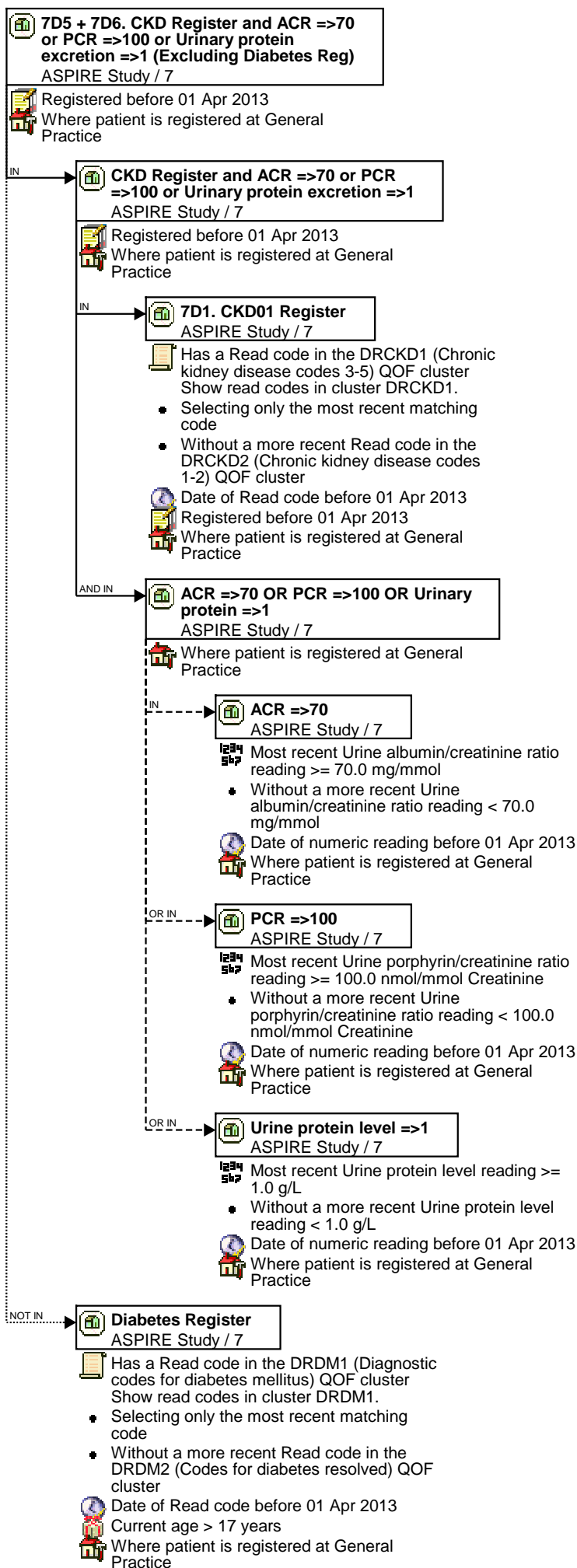

Supplement: Additional file 4 — Folder containing SystmOne™ search algorithms. (ZIP 12.7 mb) [file 12875_2015_350_MOESM4_ESM.zip › Aspire S1 diagrams tw edired/7D5+6 (CKD #47).pdf]

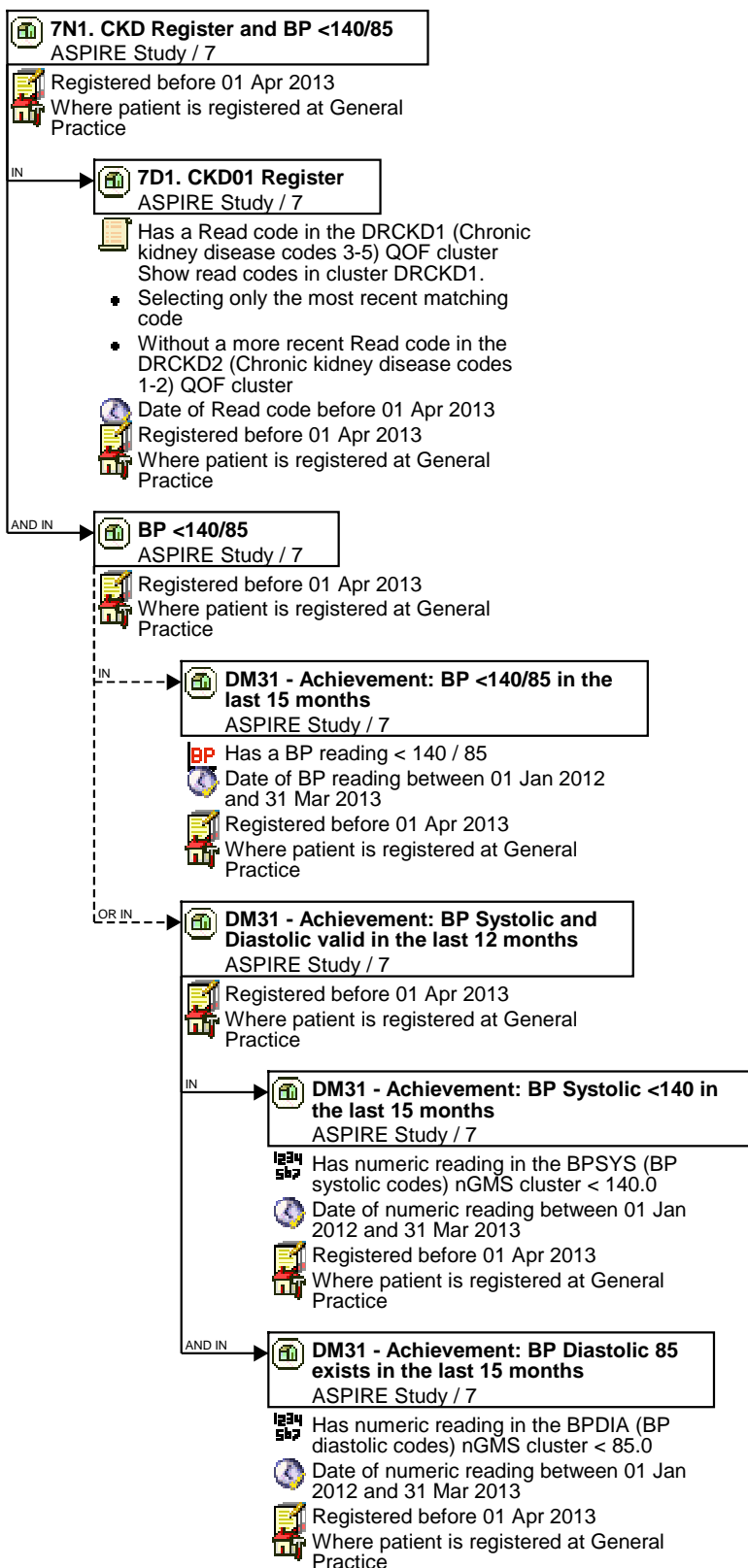

Supplement: Additional file 4 — Folder containing SystmOne™ search algorithms. (ZIP 12.7 mb) [file 12875_2015_350_MOESM4_ESM.zip › Aspire S1 diagrams tw edired/7N1 (CKD #47).pdf]

|       |              |
|-------|--------------|
| ————  | Mandatory In |
| ----- | Optional In  |
| ..... | Not In       |

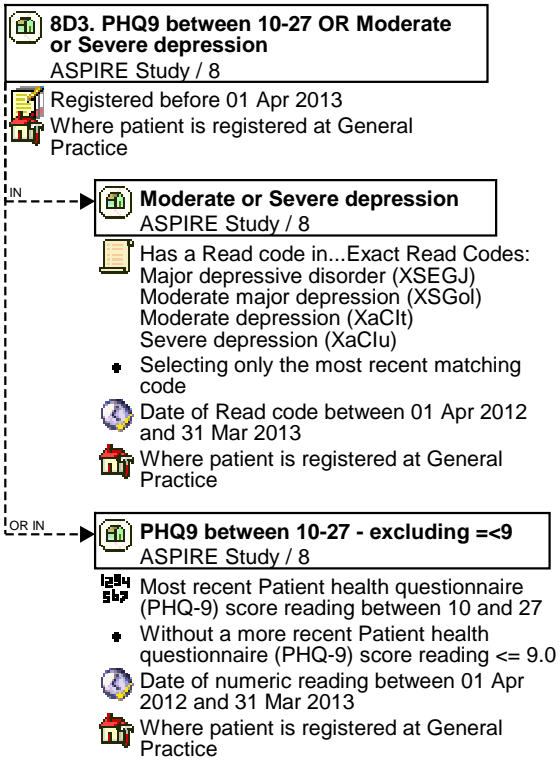

Supplement: Additional file 4 — Folder containing SystmOne™ search algorithms. (ZIP 12.7 mb) [file 12875_2015_350_MOESM4_ESM.zip › Aspire S1 diagrams tw edired/8B3 (Depression #69).pdf]

|       |              |
|-------|--------------|
| ————  | Mandatory In |
| ----- | Optional In  |
| ..... | Not In       |

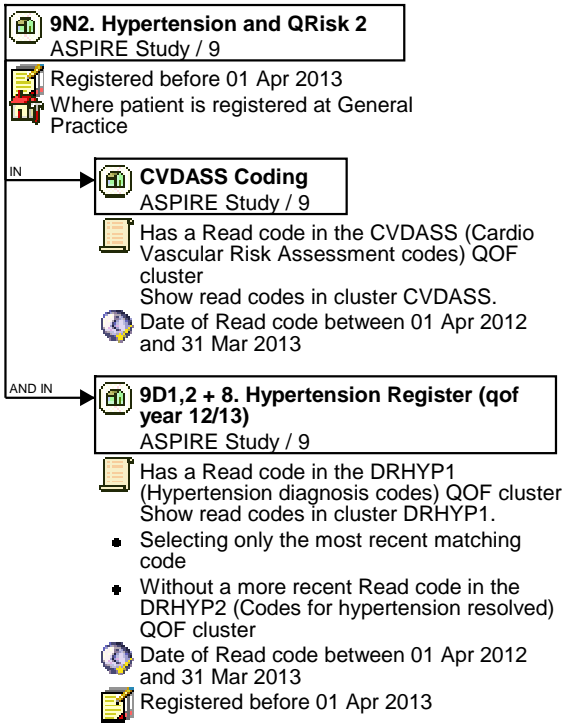

Supplement: Additional file 4 — Folder containing SystmOne™ search algorithms. (ZIP 12.7 mb) [file 12875_2015_350_MOESM4_ESM.zip › Aspire S1 diagrams tw edired/9N2 (HTN monitoring #79).pdf]

|       |              |
|-------|--------------|
| ——    | Mandatory In |
| ----  | Optional In  |
| ..... | Not In       |

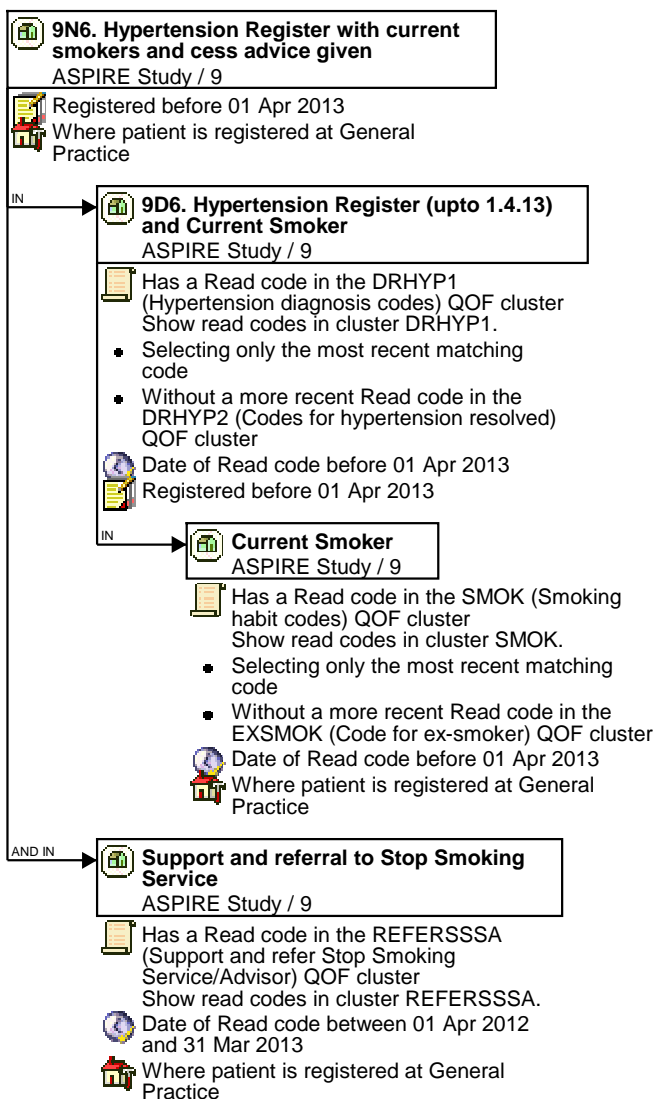

Supplement: Additional file 4 — Folder containing SystmOne™ search algorithms. (ZIP 12.7 mb) [file 12875_2015_350_MOESM4_ESM.zip › Aspire S1 diagrams tw edired/9N6 (HTN monitoring #79).pdf]

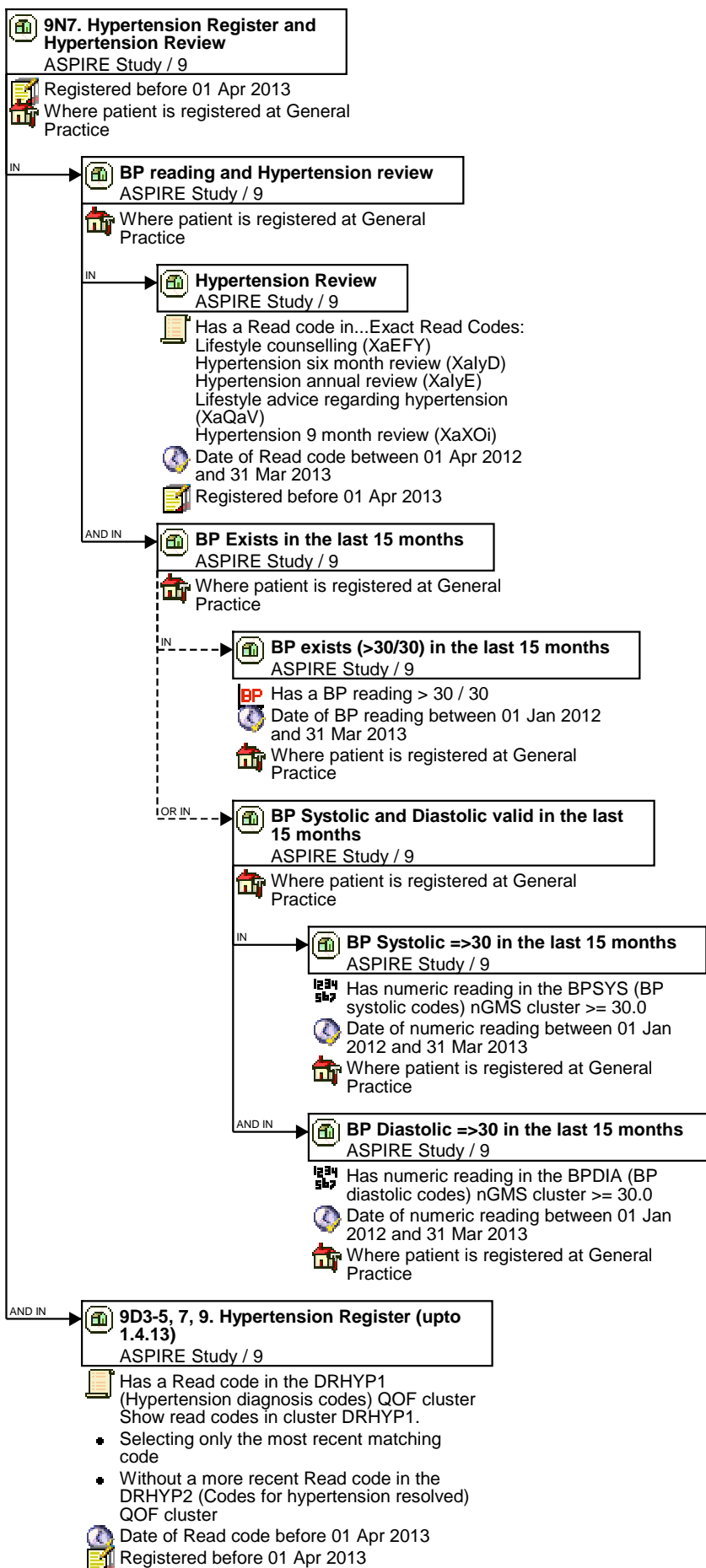

Supplement: Additional file 4 — Folder containing SystmOne™ search algorithms. (ZIP 12.7 mb) [file 12875_2015_350_MOESM4_ESM.zip › Aspire S1 diagrams tw edired/9N7 (HTN monitoring #79).pdf]
